# The structure of human motivation

**DOI:** 10.1186/s40359-023-01346-5

**Published:** 2023-10-06

**Authors:** J. David Pincus

**Affiliations:** 1 Employee Benefit Research Institute, Washington, DC, 20024 USA; 2 Research and Development Department, Leading Indicator Systems, One Franklin Street, Boston, MA 02110 USA

**Keywords:** Unified model of human motivation, Spiritual motivation, Material motivation, Social motivation, Egocentric motivation

## Abstract

**Background:**

A unified model of human motivation has been recently introduced that integrates all prior “mini-theories” of motivation into a single, symmetrical model based on first principles: four life domains crossed by three levels of attainment, resulting in 12 discrete motivations. Evidence from a series of studies using a novel image-based method is used to test structural hypotheses derived from a unified model of human motivation.

**Method:**

The studies employ large samples (810n to 986n) of working adults who conducted a time-constrained image-based exercise to measure the relative presence or absence of different emotional needs.

**Results:**

These studies provide support for the theoretical model, suggesting that there is substantial heuristic and practical value in a structured framework of motivating needs.

**Conclusions:**

Findings suggest that our theoretical model reflects deep interrelationships between discrete types of human motivation, and by linking specific measures to a comprehensive model of human motivation, researchers can have confidence that they have adequately measured the motivation construct.

**Supplementary Information:**

The online version contains supplementary material available at 10.1186/s40359-023-01346-5.

## Background

Motivation has been defined as an individual-level, unobservable state of striving, which drives and directs goal-pursuit behavior toward need fulfillment [1–3]. Motivations, then, represent unmet needs which become salient to the organism, directing the organism to pursue need fulfillment, which is experienced both affectively and cognitively, and can be expressed behaviorally. A unified model of human motivation has been recently introduced that integrates all prior “mini theories” of motivation into a single, symmetrical model based on first principles: four life domains (the domains of the Self, the Material, the Social, and the Spiritual) crossed by three levels of attainment (To Be, To Do, To Have), resulting in 12 discrete motivations (Table 1). The life domains are derived from a literature review identifying twelve distinct conceptual systems that present these four domains as representing the totality of human life drawn from the fields of philosophy, religion, and psychology. The model’s levels of attainment correspond to Aristotle’s three modes of existence, *potentiality*, *potentiality-as-such*, and *actuality*. Because there are no additional life domains or modes of existence, we can claim that this model is comprehensive [1].

**Table 1:** A unified model of human motivation (Pincus, 2023a)

Three Levels of Attainment	Four Life Domains			
	Self (A)	Material (B)	Social (C)	Spiritual (D)
Aspirational (3) (*Having)*	Fulfilling Potential (A3P) & Failure to Thrive (A3N)	Success (B3P) & Failure (B3N)	Recognition (C3P) & Scorn (C3N)	Higher Purpose (D3P) & Materialism (D3N)
Experiential (2) (*Doing*)	Authenticity (A2P) & Conformity (A2N)	Immersion (B2P) & Boredom (B2N)	Caring (C2P) & Uncaring (C2N)	Ethics (D2P) & Wrongdoing (D2N)
Foundational (1) (*Being*)	Safety (A1P) & Anxiety (A1N)	Autonomy (B1P) & Disempowerment (B1N)	Inclusion (C1P) & Exclusion (C1N)	Justice (D1P) & Injustice (D1N)

We believe that there is strong justification for introducing a new theoretical framework of human needs because past attempts at creating “unified” need models have had the unfortunate tendency to leave out large numbers of widely recognized fundamental motivations. Maslow’s need hierarchy, for example, includes the needs for safety, belonging, esteem, self-actualization, and transcendence, but leaves out the needs for autonomy, immersion, achievement, identity-formation, fairness, and morality [4]. Contemporary frameworks have been proposed that seek integration, such as Dweck’s self-coherence model of higher needs (formed by the conjunction of more basic needs) [3], but even this “unified” framework limits the range of needs to only seven: acceptance, competence, predictability, trust, control, self-esteem/status, and, the presumed root of them all, self-coherence; in a familiar pattern, this framework leaves out the needs for immersion, achievement, fairness, morality, and self-transcendent purpose. Recent structural analyses of need theories have been offered [5], but these serve as systems of cataloging dimensions such as biological vs. individual vs. social and hierarchical vs. independent vs. opponent-process, and never actually integrate all needs within a single framework. It is in response to the absence of a conceptual framework that can account for the totality of human needs that the unified pyramid of human motivation was developed.

**Fig. 1:**
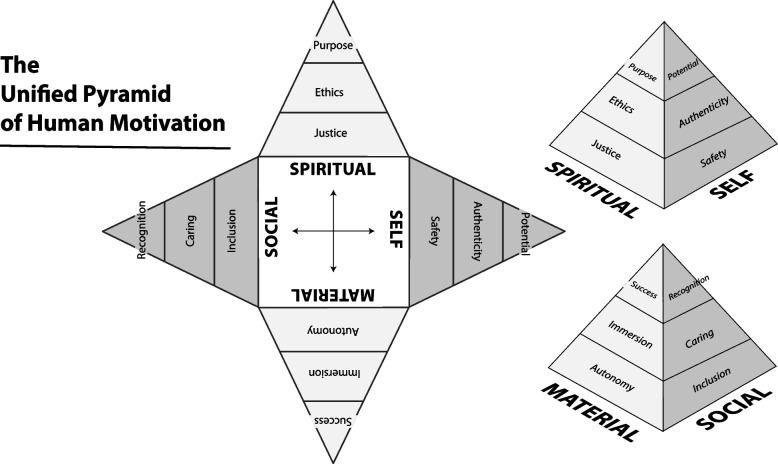
A unified pyramid of human motivation [1]

A large part of the value of this new model derives from the structural hypotheses that it suggests. From a structural perspective, the Spiritual domain is hypothesized as being closely linked with the Self and the Social domains, but less associated with, and even antipodal to, motives of the Material domain. Similarly, the domain of the Self and the Social domain are hypothesized to operate as antipodal sets of motives, linked strongly to their adjacent domains, the Material and the Spiritual. This placement of adjacencies and opposites suggests representation via a four-sided cube composed of three hierarchical layers. To reflect the hierarchical nature of the domains, i.e., greater numbers attain the foundational levels and fewer attain higher levels, the cube is reshaped to have a wider base and narrower top, resulting in a pentahedral (pyramidal) structure (Fig. 1). This paper aims to test the factor structure of this theoretical framework. Because the valence of positive and negative emotional needs is known to overwhelm subtle interrelationships when conducting factor analysis [6–8], we have conducted these analyses separately for promotion and prevention data.

The first set of hypotheses address the distinctiveness of the four life domains:

- Hypothesis 1.1. The three motivations of the Self domain (Safety, Authenticity, Potential) will form a distinct factor.
- Hypothesis 1.2. The three motivations of the Material domain (Autonomy, Immersion, Success) will form a distinct factor.
- Hypothesis 1.3. The three motivations of the Social domain (Inclusion, Caring, Recognition) will form a distinct factor.
- Hypothesis 1.4. The three motivations of the Spiritual domain (Justice, Ethics, Purpose) will form a distinct factor.

A second set of hypotheses address the distinctiveness of the three levels of attainment:

- Hypothesis 2.1. The four motivations of the Foundational level (Safety, Autonomy, Inclusion, Justice) will form a distinct factor.
- Hypothesis 2.2. The four motivations of the Experiential level (Authenticity, Immersion, Caring, and Ethics) will form a distinct factor.
- Hypothesis 2.3. The four motivations of the Aspirational domain (Potential, Success, Recognition, Purpose) will form a distinct factor.

A third set of hypotheses address the antipodal nature of the Self vs. Social and Material vs. Spiritual domains:

- Hypothesis 3 summary. Models composed of adjacent life domains will show better fit than models composed of antipodal life domains.
- Hypothesis 3.1: A factor combining the Self and Material domains will show relatively good fit.
- Hypothesis 3.2: A factor combining the Self and Spiritual domains will show relatively good fit.
- Hypothesis 3.3: A factor combining the Social and Material domains will show relatively good fit.
- Hypothesis 3.4: A factor combining the Social and Spiritual domains will show relatively good fit.
- Hypothesis 3.5: A factor combining the Self and Social domains will show relatively poor fit.
- Hypothesis 3.6: A factor combining the Material and Spiritual domains will show relatively poor fit.

### Existing support for the model

Support for the proposed structure comes from the body of cross-cultural work on subjective well-being, which has consistently identified two dimensions along which cultures can be distinguished. One of these dimensions pertains to self-orientation vs. social orientation. In communalistic cultures, fulfilling *other*-directed needs, as opposed to egocentric needs, is consistently more associated with higher levels of well-being. In individualistic cultures, the fulfillment of *self*-oriented needs is associated with enhanced well-being [9–12]. The other key recurring dimension is the degree to which cultures place value on materialism vs. idealism, which has been found to be associated with differing levels of industrialization. The most industrialized cultures are marked by consumerist materialism whereas less industrialized, traditional cultures tend to be far more idealistic and spiritual, and need fulfillment that aligns with these endpoints produces greater levels of subjective well-being in those respective cultures [13–15].

Across the many literature reviews of the cross-cultural subjective well-being literature, these are the two most reported dimensions [16]. We suggest that these dimensions are in no way arbitrary but instead represent the fundamental tradeoffs that define all higher order human needs: the degree to which we focus on one our own needs vs. those of others, and the degree to which we focus on materialism vs. higher ideals. The fact that these dimensions systematically determine levels of well-being in different cultures provides strong support for the fundamental structure of our model.

Going a level deeper, research on the sanctification of life goals [17–21] is strongly supportive of the proposed model, particularly regarding the position of the Spiritual domain relative to the other three domains. After explicitly religious goals, the personal strivings that are most likely to be sanctified (i.e., perceived to represent manifestations of God or exhibit other sacred qualities) tend to be those associated with the Social domain (i.e., helping others, family connections) and the Self domain (i.e., existential issues and other self-relevant issues). Within each domain they found sanctification associated with certain types of social goals, such as altruism, and with certain self-oriented goals, such as existential goals, but not others. Social goals that are more distal (e.g., staying in contact with friends regardless of distance) as well as Self-oriented self-improvement goals that blur into the Material domain (e.g., to keep on learning and pursuing my degree) were less likely to be sanctified. Purely instrumental goals, those related to the physical, material world (i.e., work, money, exercise, travel, home improvement, etc.) were *least* likely to be sanctified. These findings suggest strong linkage between the Spiritual domain and both the Social and Self domains, but not the Material domain.

### Rapid exposure image selection data

We sought to extend the generalizability of our theoretical model by employing an alternative method, removing it from the purview of traditional scalar approaches into the realm of affective measures. Because human motivation is inherently affective [22], we hoped that employing an affective measure would represent a better match for our research goals. To this end, we employed an image-based exercise with a rapid exposure, rapid response protocol as a means of bypassing rational filtering of responses. Our method substantially limited the opportunity for conscious thought, requiring split-second “gut-level” reactions, an important attribute for our purposes because of the close conceptual connection between motivation and emotion.

Because the unified pyramid model seeks to integrate the entirety of the logistical possibilities of human motivation, we sought to test the model’s hypothesized structure in as broad a manner as possible. The proposed model should be able to accommodate different settings, cultures, and measurement approaches. Accordingly, this novel approach to measurement via rapid image selection represents a good fit for that purpose because it does not rely on language-based scales which are prone to issues of response style.

## Method

### Measurements

There is a long history of using images to conduct implicit motive research [23–37]. These techniques generally involve exposing subjects to a series of standardized images and asking for their interpretations of scenes, which are then coded and scored. To avoid problems of reliability and validity associated with this open-ended and somewhat subjective approach, we have developed a set of images to represent each of the 12 motivational concepts of the proposed model, both in terms of their positive, promotional expression (i.e., those states that one might desire to feel *more* of) and their negative, preventative expression (i.e., those states that one might desire to feel *less* of).

### Image validation

The process of image development involved testing 320 stock photos taken from Shutterstock’s image library in live tests with 2,728 research participants, resulting in distinct sets of images corresponding to each motivational construct. Rules for image content were derived from a combination of image searching by keywords within Shutterstock’s library, with key distinguishing characteristics of images recorded, and interviews with artists and photographers to verify these characteristics as distinctively communicating each motivational concept. Using a rapid-exposure, rapid-response protocol, subjects were asked to complete a sentence by selecting those positive (promotion) images that represented the ways they wished to feel *more* and to not select those images that did not complete the sentence for them. The exercise was repeated using negative (prevention) images wherein subjects were asked to select images that represented ways they wished to feel *less* and to not select those images that did not complete the sentence for them. A final set of 72 images were selected for inclusion using the Kuder-Richardson (KR20) technique, with the minimal acceptable value of 0.8, for assessing the internal consistency of binary datal; 248 images with lower KR20 values, even if they met the 0.8 threshold, were not used. Six raters, who had been extensively trained in the definitions for each motivation, and tested for their ability to clearly differentiate them, categorized the resulting sets of images into the 12 promotion motives and the 12 prevention motives, achieving a Cohen’s kappa of 0.91, indicating almost perfect agreement. Separate sets of images were created using this method for each of the 12 promotion motivations (“ways I want to feel more…”) and for each of the 12 prevention motivations (“ways I want to feel less…”).

### Sentence completion task

To measure motivational states, participants were provided a context known to be associated with strong feelings, both promotion-focused and prevention-focused, one’s job or occupation: “Thinking about my job, I wish I could feel a little more…/less….” This approach to setting an emotional context is a form of priming, a well-established psychological procedure involving exposure to a stimulus in advance of a task to activate a schema (or frame of mind) within which the task can proceed. The effectiveness of priming to activate schemata has been well documented [38, 39].

### Testing protocol

Bringing all these elements together resulted in the following test protocol:

1. Each subject is asked to concentrate on how they feel about their job.
2. The protocol is presented as a sentence completion task.
3. A practice round of six images is offered to prepare subjects for the speed of image presentation.
4. Thirty-six promotion motivation images are shown in random order.
5. Instructions are presented for the prevention motivation task, and the process repeats for the 36-prevention motivation image set, also shown in random order.

Images are displayed within the designated time frame and are selected by subjects via clicks of the mouse, space bar, or touchscreen. The number of images selected defines individual-level scores for each motive. The resulting data represent individual-level profiles of promotion and prevention motivations associated with any given context, which can be aggregated and compared using any other variable in the study (e.g., experiences, attitudes, demographics, etc.).

### Timing of image presentation

The timing of image exposures is based on neurological research that details the time course of emotional visual perception [40–47]. Using magnetoencephalography, Rudrauf et al. (2008 [43], 2009 [44]) have delineated the sequence associated with the neural process of emotional and cognitive reactions to emotionally evocative images. These replicated findings show that from the time images are first processed in the visual cortex to the initiation of emotional response roughly 500 ms have elapsed. Damasio has provided a concise summary of the findings:

*These researchers' efforts indicate that this earliest stage of emotional reaction to a stimulus takes place in a sub-one-second time frame – about five hundred milliseconds… In ‘conscious mind time,’ [this] …sits between the couple of hundred milliseconds we require to be conscious of a pattern in perception and the seven or eight hundred milliseconds we need to process a concept.* (p. 122) [42].

Applying these findings, this technique uses a measurement period that begins at 500 ms and ends at 1500 ms; although the purely emotional response ends at approximately 750 ms, we needed to add the average motor reaction time for visual discrimination tasks (via mouse click) of 550 ms [48] and the 200-ms latency associated with 4G mobile networks [49], which are necessary to manifest responses that are initiated in the 500 to 750-ms window. By keeping responses within this window of time, we ensure that reactions stem primarily from emotional processes since recording ends before cognitive reprocessing (conscious reflection) can come into play [41], keeping them free from cognitive distortions such as demand characteristics or impression management. Through this protocol, we avoid the challenges associated with conscious, rational self-report methods to identify emotions, while providing richer motivational insights than can be provided by physiological measures such as electromyography, skin conductance, or functional magnetic resonance imaging.

### Instrument

Three large-scale surveys included the image protocol to assess employee emotional needs. These questionnaires also included a variety of indicators of employee well-being, including the Center for Epidemiologic Studies Depression Scale (10-item version) [50, 51]; the Perceived Stress Scale (PSS-10) [52]; and the Brief COPE scale (28-item version) [53]. These surveys also included measures that are conceptually related to individual motivations including the Big Five Personality Inventory (BFI-15) [54] and the Rokeach Terminal Value Survey (18 items) [55] for establishing points of comparison to establish discriminant, convergent, and concurrent validity. These measures serve as scalar independent variables used to predict two sets of dependent variables. For establishing convergent validity, the analyses employ the image-based motivational measures, these scalar measures, and the two combined as independent variables and two psychometrically validated measures as dependent variables:

- Self-Reported Burnout Scale (single item; respondents classify their level of burnout in one of five levels [56]
- Self-Reported Intention to Quit Scale (3 items) [57]

### Samples

This exercise was administered to three population-representative samples of US-based employees working full time who were recruited by InnovateMR, a professional research panel company, to participate in an 18-min anonymized survey, for which they were compensated using the panels’ compensation systems. Ethics approval and consent to participate: All subjects are members of commercial survey panel, InnovateMR, which is governed by their own ethical review processes and guidelines. Accordingly, there was no need for ethics approval. Waves were collected in March 2021, May 2022, and June 2023. Participants were drawn from population-representative samples of American employees who are currently working full time for companies with at least 20 employees, in proportion to the Bureau of Labor Statistics’ distributions of employment by employer size. This step ensures that the samples are representative of the US population of similarly situated workers. Each wave of this national survey has a statistical confidence level of 95 percent with margins of error ranging from ± 3 to 5 percent. The resulting samples are generally representative of US full-time workers as estimated by the U.S. Bureau of Labor Statistics Current Population Survey, corresponding to BLS distributions for sex, age, and race; an exception is the March 2021 wave, the sample of which skewed toward 35–44 year old employees, resulting in underrepresentation of older and younger age groups, as well as Hispanics, anomalies that were corrected in subsequent waves (Table 2). Because respondents were able to select more than one category of race, race distributions vary slightly from BLS estimates and sum to over 100%. Unless otherwise noted, results refer to analyses conducted using the May 2022 dataset.

**Table 2:** Sample characteristics

	Bureau of Labor Statistics^a^	March 2021	May 2022	June 2023
Sample size	60,000	932	810	986
Margin of error at 95% CI	± < 1%	± 3%	± 3%	± 3%
Response rate	71%	35%	38%	26%
Sex				
Male	56.3%	57.0%	55.4%	56.6%
Female	43.7%	43.0%	44.6%	43.4%
Age				
18–19	1.0%	0.4%	1.0%	0.8%
20–24	7.2%	4.7%	7.0%	7.4%
25–34	25.1%	23.9%	25.6%	23.9%
35–44	24.4%	39.8%	24.9%	24.4%
45–54	23.4%	19.0%	23.4%	23.8%
55–64	19.0%	12.0%	18.1%	19.6%
Race				
Asian (non-Hispanic)	6.6%	6.4%	6.9%	6.9%
Black (non-Hispanic)	11.8%	12.3%	12.9%	12.9%
Hispanic/Latino	18.5%	13.6%	17.8%	19.5%
White (non-Hispanic)	63.0%	70.6%	70.2%	68.5%

^a^U. S. Bureau of Labor Statistics (2023). Labor Force Statistics from the Current Population Survey. Demographic characteristics of U.S. workers, employed, usually work full-time, by age, sex, and race

## Method validation

### Data transformation

Past studies have demonstrated that stronger, more accessible emotional responses are reflected in faster response times [58–60]. Accordingly, all analyses of the image-based data were based on response latencies associated with image selections. Each latency was subtracted from the maximum exposure period (1,600 ms), effectively inverting the latencies into strength scores, such that larger scores indicate faster responses and presumably stronger emotional responses.

### Internal reliability

The images chosen to represent each motivation, both positive and negative, were evaluated for internal consistency using the Kuder-Richardson KR20 (a version of Cronbach’s alpha appropriate for binary data). KR20 provides a strong test for three reasons: Firstly, negative affective measures are known to show less variance than positive affective measures, which has been attributed to a general fear factor [6–8], and by including positive and negative measures together, alpha is suppressed. Secondly, coefficient alpha values are determined largely by the number of items assessed; since we are evaluating a mere six binary variables per cell, alpha values are necessarily suppressed. Thirdly, KR20 yields more conservative estimates than Cronbach’s alpha, which also contributes to the suppression of coefficient alpha. For these three reasons, we propose that the minimum adequacy of 0.60 should be adopted. When all image data is merged within theorized life domains (i.e., Self, Material, Social, and Spiritual), all alphas meet or exceed the 0.90 threshold (Table 3). At the level of the 12 distinct motives, all exceed the 0.80 threshold, exceeding the generally accepted level for unipolar, scalar affective measures [61], the nearest relevant standard.

**Table 3:** Coefficient alpha values using KR20 for life domains and cells of the model

	**Self** **0.93**	**Material** **0.93**	**Social** **0.93**	**Spiritual** **0.93**
**Aspirational**	Fulfilling Potential & Failure to Thrive 0.82	Success & Failure 0.81	Recognition & Scorn 0.83	Higher Purpose & Materialism 0.81
**Experiential**	Authenticity & Conformity 0.81	Immersion & Boredom 0.80	Caring & Uncaring 0.83	Ethics & Wrongdoing 0.83
**Foundational**	Safety & Anxiety 0.81	Autonomy & Disempowerment 0.82	Inclusion & Exclusion 0.80	Justice & Injustice 0.82

### Stability over time

In May 2022, research was conducted using 60 matched subjects who had previously completed the exercise in the March 2021 wave, a 14-month gap. Because the exercise is designed to measure motivation concerning a particular object, event, or situation (e.g., “my job,” “the direction my life is going,” “my most important relationships,” etc.) at a particular point in time, our expectations were modest regarding the strength of test–retest correlations because emotional needs can be expected to change over a year (e.g., they can be satisfied or become more intense in the interim). Nevertheless, test–retest reliabilities were calculated overall and found to be extremely durable, exceeding 0.65 for overall positive and negative scores, as well as each of the four domains (Self, Material, Social, Spiritual; Table 4).

**Table 4:** 14-Month test–retest correlations (*n* = 60)

Domains	*r*	*p*
Overall Positive	0.75	< .01
Overall Negative	0.67	< .01
Self	0.72	< .01
Material	0.71	< .01
Social	0.78	< .01
Spiritual	0.67	< .01

### Convergent validity

With satisfactory reliability established, the validity of the method was examined. In the absence of comparable measures of motivation, finding a similar metric against which convergent validity could be assessed presented a challenge. Fortunately, theorists have proposed that employee engagement can be viewed as a surrogate for motivation [62, 63]. The absence of engagement has been defined by Maslach and others as *job burnout* [64, 65], a condition that is strongly associated with intention to quit [66]. Accordingly, we regressed our scores against psychometrically validated scalar measures of burnout [56] and intent to quit [57] as dependent variables.

We sought to compare our method’s ability to predict these two motivational outcomes against three established measures of well-being: CESD-10 [50, 51]; PSS-10 [52]; Brief COPE-28 [53]; as well as two motivationally relevant measures, BFI-15 [54] and RVS-18 [55]. Because the two sets of measures (i.e., rapid image selection vs. slow analytic deliberation) represent fundamentally different modes of knowing, one that is relatively automatic and under weak voluntary control, and another that works more slowly and deliberatively [67], we expected the ability of each type of predictor to explain different amounts of variance in the dependent variables. These results are summarized in Table 5; the full output is available in Supplementary Material Table 5. In every case, the image-based method produced models that significantly explained the variance in both dependent variables. In every case, the image-based method explained substantially greater variance than any of the established measures, typically between two and four *times* the variance as measured by R-squared. Models were estimated using the combination of each of the established measures one at a time (in addition to the image-based measures), which increased the amount of variance explained marginally by 10 to 30 percent. Entering *all* variables simultaneously increased the amount of variable explained by a more substantial 50 to 60 percent over the image-based measures alone. Consistent with our expectations, results demonstrate that the image-based measures and established scalar measures perform very differently. The image-based method significantly explains variance in both motivational outcomes, self-reported burnout and intent to quit, at rates representing *multiples* of the amount of variance explained by alternative methods.

**Table 5:** Summary of regression models: Image-based and established measures as predictors of *burnout* and *intent to quit*

Dependent variable: Burnout	R	R-squared	Cohen’s f	Effect size	Dependent variable: Intent to Quit	R	R-squared	Cohen’s f	Effect size
*Independent variables*					*Independent variables*				
Image-based method	0.36	0.13	0.386	L	Image-based method	0.35	0.12	0.374	L
BFI-15	0.24	0.06	0.247	M	BFI-15	0.16	0.03 ns	0.162	M
Image-based method + BFI-15	0.41	0.17	0.450	L	Image-based method + BFI-15	0.38	0.14	0.411	L
Image-based method	0.36	0.13	0.386	L	Image-based method	0.35	0.12	0.374	L
PSS-10	0.10	0.01 ns	0.101	S	PSS-10	0.19	0.04	0.194	M
Image-based method + PSS-10	0.41	0.17	0.450	L	Image-based method + PSS-10	0.37	0.14	0.398	L
Image-based method	0.36	0.13	0.386	L	Image-based method	0.35	0.12	0.374	L
Brief COPE-28	0.22	0.05	0.226	M	Brief COPE-28	0.16	0.03 ns	0.162	M
Image-based method + Brief COPE-28	0.41	0.17	0.450	L	Image-based method + Brief COPE-28	0.39	0.15	0.424	L
Image-based method	0.36	0.13	0.386	L	Image-based method	0.35	0.12	0.374	L
RVS-18	0.17	0.03* ns*	0.173	M	RVS-18	0.16	0.03 ns	0.162	M
Image-based method + RVS-18	0.39	0.15	0.424	L	Image-based method + RVS-18	0.38	0.14	0.411	L
Image-based method	0.36	0.13	0.386	L	Image-based method	0.35	0.12	0.374	L
BFI-15	0.24	0.06	.0247	S	BFI-15	0.16	0.03	0.162	M
PSS-10	0.10	0.01* ns*	0.101	S	PSS-10	0.19	0.04	0.194	M
Brief COPE-28	0.22	0.05	0.226	M	Brief COPE-28	0.16	0.03* ns*	0.162	M
RVS-18	0.17	0.03* ns*	0.173	M	RVS-18	0.17	0.03* ns*	0.173	M
Image-based method + BFI-15 + PSS-10 + Brief COPE-28 + RVS-18	0.44	0.20	0.493	L	Image-based method + BFI-15 + PSS-10 + Brief COPE-28 + RVS-18	0.44	0.20	.493	L

All models are significant at < 0.01 unless noted *ns* (non-significant)

Cohen’s f interpretation of effect sizes: S (small) = .01-.14; M (medium) = .150-.34; L (large) = .35 or larger

Cohen J. E. (1988). Statistical Power Analysis for the Behavioral Sciences. Hillsdale, NJ: Lawrence Erlbaum Associates, Inc

Effect sizes were also calculated using the Cohen’s f statistic, a preferred method for estimating effect sizes for multiple regression [68]. Using Cohen’s guidelines, we observed that all of the 20 models that utilized image-based data were associated with large effect sizes. None of the sixteen models that excluded image-based data showed large effects. We take these findings of improved predictiveness of motivational outcomes as evidence that the image-based method demonstrates convergent validity.

### Discriminant validity

Discriminant validity is the degree to which a test or measure diverges from (i.e., does not correlate with) another measure whose underlying construct is conceptually unrelated to it. In support of a strong test of discriminant validity, we included a series of established measures of the related but distinct concepts of values, personality traits, and coping styles. To assess values, we administered the Rokeach Values Survey, an 18-item ranking exercise [55]. To assess the Big Five personality traits of Openness, Conscientiousness, Extraversion, Agreeableness, and Neuroticism, we employed the 15-item Big Five Inventory (BFI-S) short-form assessment [54]. To assess the tendency to rely on social support, we included four relevant items from the Brief COPE scale [53]. Regarding discriminant validity, we anticipated a nonsignificant or weak relationship between the RVS, BFI-S, and B-COPE and the motivational profiles produced by our assessment. These measures were collected alongside the assessment in two waves of large-scale survey research in March 2021 (*n* = 932) and May 2022 (*n* = 810). Descriptive analyses showed no missing data on these variables. Results show very few significant correlations between the new measure and the RVS, BFI-S, and B-COPE despite large sample sizes. The largest correlation between any of these measures and any of the 24 image-based variables tested concurrently is 0.18, a weak but significant negative correlation between striving to fulfill one’s potential and the tendency not to prioritize justice as a value (i.e., those who strive to be the very best tend to value fairness less). Other weak but significant correlations similarly demonstrate a pattern that fits the conceptual definitions of the relevant motivations, including those between striving to fulfill potential and conscientiousness (*r* = 0.11; i.e., the trait of being hardworking is associated with striving for self-actualization); striving for authenticity and openness to experience (*r* = 0.12; i.e., openness is associated with the striving to express one’s true identity); striving for success and agreeableness (*r* = 0.12; i.e., “yea saying” associated with the desire for success) (Table 6).

**Table 6:** Discriminant validity of image-based method vs. BFI, RVS, and social support items of B-COPE (November 2021)

Category	Measure	M (SD)	A1	A2	A3	B1	B2	B3	C1	C2	C3	D1	D2	D3
Personality trait	Conscientiousness	3.82 (0.71)	0.063	-0.058	.108^a^	0.057	-0.006	.102^a^	0.062	0.03	.084^b^	0.073	0.035	0.018
Personality trait	Extraversion	3.15 (0.78)	-0.071	-.078^b^	-0.065	-.078^b^	-0.033	-0.019	0.024	-0.02	0.028	-0.042	-0.008	-0.054
Personality trait	Neuroticism	2.98 (0.85)	0.026	0.068	0.041	0.029	.137^a^	0.01	0.033	0.072	0.067	-0.002	0.047	0.025
Personality trait	Openness	3.84 (0.76)	-0.006	.115^a^	-0.035	0.039	0.007	0.05	0.016	0.042	0.04	0.004	0.046	0.031
Personality trait	Agreeableness	3.77 (0.75)	0.07	0.021	0.044	0.056	0.016	.124^a^	.095^b^	0.05	0.062	0.061	0.003	.077^b^
Terminal value	True Friendship	4.50 (3.57)	0.038	0.042	0.061	0.065	-0.022	0.069	-0.011	-0.003	0.01	0.013	-0.062	0.021
Terminal value	Mature Love	4.69 (3.63)	-0.011	-0.031	0.036	0.023	0.041	0.029	0.025	0.012	0.039	0.012	-0.042	0.019
Terminal value	Self-respect	4.20 (2.89)	-0.071	-0.058	-0.01	0.025	-.121^a^	0.012	0.013	-0.047	0.026	-0.025	-.131^a^	-0.053
Terminal value	Happiness	4.13 (2.69)	0.004	0.014	-0.018	-0.011	0.016	0.002	-0.041	-0.049	0.034	-0.053	0.005	0.008
Terminal value	Inner Harmony	6.13 (2.97)	-0.016	-.097^b^	-0.013	0.016	-.081^b^	-0.05	-0.016	-0.024	-0.03	-0.044	-0.032	-0.035
Terminal value	Equality	7.03 (3.04)	.138^a^	.086^b^	**.182**^a^	.125^a^	0.057	.104^a^	.095^b^	0.063	0.073	0.017	0.051	0.041
Terminal value	Freedom	7.15 (3.18)	0.01	0.042	.084^b^	0.027	-0.043	.089^b^	0.005	-0.022	0.014	0.044	-0.025	0.03
Terminal value	Pleasure	8.99 (3.11)	0.022	0.035	0.043	0.023	-0.02	0.045	-0.008	-0.036	-0.029	0.027	-0.029	0.021
Terminal value	Social Recognition	11.00 (3.58)	0.035	-0.063	0.042	.082^b^	-0.001	-0.053	0.016	0.016	-0.048	-0.035	-0.03	-0.028
Terminal value	Wisdom	9.71 (3.39)	0.063	0.049	0.027	0.048	0.066	0.034	0.029	0.038	0.044	0.058	0.027	0.043
Terminal value	Salvation	11.86 (3.63)	0.034	0.032	0.022	0.043	0.031	0.051	0.066	0.038	0.019	.091^b^	-0.033	0.068
Terminal value	Family Security	9.16 (4.76)	-0.041	-0.013	-0.052	-0.041	0.016	-0.069	-0.002	-0.006	0.002	-0.012	0.067	-0.036
Terminal value	National Security	13.12 (3.27)	0.056	-0.023	0.039	0.032	-0.025	0.03	.094^b^	0.002	0.009	0.019	0.045	0.002
Terminal value	A Sense of Accomplishment	12.59 (3.59)	0.019	-0.028	-0.04	-0.068	0.047	-0.074	-0.011	0.046	0.004	-0.028	0.026	-0.006
Terminal value	A World of Beauty	14.87 (2.53)	0.06	0.07	0.046	0.07	0.061	0.068	.078^b^	.080^b^	.077^b^	0.073	.129^a^	0.074
Terminal value	A World at Peace	14.26 (3.90)	0	0.045	-0.056	-0.037	0.021	0.035	-0.062	0.003	-0.006	-0.011	0.024	-0.026
Terminal value	A Comfortable Life	12.46 (5.61)	-.092^b^	0.01	-.134^a^	-.104^a^	0.018	-.108^a^	-0.074	0.022	-0.034	-0.036	0.058	-0.023
Terminal value	An Exciting Life	15.15 (4.18)	-.125^a^	-.084^b^	-.093^b^	-.162^a^	-.078^b^	-0.072	-.095^b^	-.121^a^	-.122^a^	-0.064	-.078^b^	-0.062
Coping style	Social Support	9.84 (3.36)	-0.025	-0.001	-.143^a^	-.087^b^	0.039	-.134^a^	-0.056	-0.013	-0.069	-.112^a^	-0.031	-0.067

^a^Correlation is significant at the 0.01 level (2-tailed)

^b^Correlation is significant at the 0.05 level (2-tailed)

A replication using the RVS, BFI-S, and social support items of the B-COPE was fielded in March 2022. Again, very few significant correlations were observed among the identical 24 image-based variables tested concurrently. The largest correlation between any of our measures and any of the concurrent measures tested is 0.15, a weak but significant correlation between striving for caring/intimacy and the tendency *not* to prioritize social recognition as a value (i.e., those who strive for true intimacy tend not to care about being admired by others). Other weak but significant correlations observed in the prior wave of research were replicated including those between striving to fulfill potential and conscientiousness (0.12; i.e., being hardworking is associated with striving for self-actualization) (Table 7). In sum, the new measures were highly distinctive and non-duplicative of other measures.

**Table 7:** Discriminant validity of image-based method vs. BFI, RVS, and social support items of B-COPE (March 2022)

Category	Measure	M (SD)	A1	A2	A3	B1	B2	B3	C1	C2	C3	D1	D2	D3
Personality trait	Conscientiousness	3.85 (0.77)	0.041	-0.009	.115^a^	-0.014	.072^b^	-0.014	0.064	0.058	.092^a^	0.02	0.043	0.038
Personality trait	Extraversion	3.14 (0.84)	-0.041	0.067	-0.029	-0.02	-0.013	-0.02	0.025	0.036	0.019	0	0.023	-0.013
Personality trait	Neuroticism	2.86 (0.99)	-0.021	-.074^b^	-0.016	-0.031	-0.023	-0.031	-.099^a^	-0.067	-.083^b^	-0.012	-0.056	-0.043
Personality trait	Openness	3.84 (0.76)	0.003	0.069	0.013	-0.007	0.058	-0.007	0.057	-0.017	0.002	-0.002	0.011	0.027
Personality trait	Agreeableness	3.77 (0.75)	0.057	0.013	.114^a^	0.033	.105a	0.033	.134^a^	.104^a^	.095^a^	.105^a^	.077^b^	.080^b^
Terminal value	True Friendship	3.65 (3.22)	0.031	-.076^b^	.095^a^	-0.011	-0.036	-0.011	-0.051	0.049	-0.037	0.025	-0.04	-0.039
Terminal value	Mature Love	4.38 (3.47)	.081^b^	0.013	.117^a^	0.066	0.028	0.066	0.015	.111^a^	0.049	0.058	0.029	-0.021
Terminal value	Self-respect	3.87 (2.43)	-0.031	-.085^b^	0.008	-0.05	-0.009	-0.05	-0.045	-0.025	-0.013	-0.06	-0.059	0.011
Terminal value	Happiness	4.32 (2.40)	-0.057	-0.056	0.008	-0.061	-0.012	-0.061	-0.016	0.031	-0.02	-0.05	0	-0.028
Terminal value	Inner Harmony	6.08 (2.69)	-0.037	-0.028	-0.042	-0.03	-0.018	-0.03	0.008	-0.03	-0.026	-0.002	-0.059	-0.021
Terminal value	Equality	7.58 (3.35)	0.013	-.121^a^	.085^b^	-0.004	-0.035	-0.004	-0.06	0.038	-0.015	-0.044	-0.035	-0.056
Terminal value	Freedom	7.06 (2.91)	0.058	0.052	0.019	0.001	0.014	0.001	0.062	.101^a^	0.025	.090^b^	0.059	0.018
Terminal value	Pleasure	8.94 (2.89)	0.032	-0.032	.098^a^	0.033	0.042	0.033	0.029	.074^b^	0.018	-0.017	0.011	0.026
Terminal value	Social Recognition	11.16 (3.76)	.086^b^	-.072^b^	.133^a^	0.056	0.021	0.056	-0.009	**.148**^a^	0.034	0.02	0.018	0.029
Terminal value	Wisdom	9.23 (3.32)	0.012	0.027	-0.042	0.024	0	0.024	0.041	-0.03	0.021	0.039	-0.007	0.001
Terminal value	Salvation	10.95 (3.96)	-0.018	-0.044	-0.043	-0.026	-0.042	-0.026	-0.012	-0.06	0.013	0.03	-0.049	-0.002
Terminal value	Family Security	9.46 (4.45)	-.081^b^	0.05	-.107^a^	-0.004	-0.049	-0.004	-0.057	-.139^a^	-.070^b^	-.076^b^	-0.04	-0.057
Terminal value	National Security	13.17 (2.77)	-0.016	0.008	0.029	-0.002	-0.009	-0.002	-0.004	-0.034	-0.035	0.006	-0.035	0.016
Terminal value	A Sense of Accomplishment	12.87 (3.28)	0.021	0.058	-0.054	-0.021	.089^b^	-0.021	0.046	-0.005	0.016	0.022	0.063	0.064
Terminal value	A World of Beauty	14.71 (2.48)	-0.047	0.003	-0.021	-0.022	-0.004	-0.022	-0.06	-0.045	0.02	-0.047	-0.064	-0.012
Terminal value	A World at Peace	14.02 (4.12)	-0.033	0.046	-.073^b^	0.028	-0.025	0.028	-0.008	-0.067	0.011	-0.057	0.002	-0.026
Terminal value	A Comfortable Life	13.64 (4.94)	0.004	.111^a^	-0.057	0.011	0.042	0.011	.073^b^	-0.025	0.025	0.026	.107^a^	0.037
Terminal value	An Exciting Life	15.9 (3.57)	-0.027	0.038	-0.067	-0.04	0.005	-0.04	0.014	-0.025	-0.026	0.02	0.023	0.056
Coping style	Social Support	9.27 (3.19)	-0.033	0.038	-0.035	-0.052	0.019	-0.052	0.015	-0.006	-0.043	-0.014	0.004	0.006

^a^Correlation is significant at the 0.01 level (2-tailed)

^b^ Correlation is significant at the 0.05 level (2-tailed)

Discriminant validity has been defined in many ways, including discrimination of concepts within the same measurement framework. CI_CFA_(sys) is a new method [69] for measuring this kind of discriminant validity, and is based on the confidence intervals (CIs) generated by confirmatory factor analysis (CFA) as a means to assessing the degree to which a construct is truly distinct from other constructs. It does this by assessing the confidence interval of the correlations between the constructs with the square roots of the Average Variance Extracted (AVE) for each construct, looking for the presence of correlations between any two factors include the value 1.0 or if the correlation estimate is greater than the square roots of the AVEs of the factors involved. If either condition is met, discriminant validity could be an issue for those factors. In all cases, the correlations between all factors were below 1.0 but these correlations were larger than the corresponding AVEs, which were estimated at 0.394 (Self), 0.417 (Material), 0.437 (Social), and 0.421 (Spiritual), and the AVEs associated with pairing of these factors, which ranged from 0.627 (Self) to 0.661 (Social). The full output is available in Supplementary Material Table 6.7. The failure of the CI_CFA_ test is a notable deficiency of the method that will be explored in future work devoted to further differentiating the stimuli used to represent each construct.

### Criterion validity

Criterion validity is an indicator of how well a test correlates with an established standard of comparison. Criterion validity is generally divided into three types: retrospective validity (i.e., it can predict past outcomes), concurrent validity (i.e., it can predict current outcomes), and predictive validity (i.e., it can predict future outcomes). Because we did not have access to data about the past, we were restricted to concurrent and predictive validations.

### Concurrent validity

To estimate the concurrent validity of our resulting data, we proposed a set of seven hypotheses^1^:

- H-CV1. The personality trait of Openness to Experience will be significantly associated with the promotional need for Authenticity, the need to express one’s genuine identity, and Immersion, the need for optimal experiences.
- H-CV2. The personality trait of Extraversion will be significantly associated with the promotional need for Inclusion, the need to connect meaningfully with others.
- H-CV3. The personality trait of Agreeableness will be significantly associated with the promotional need for Ethics, the need to subjugate self-interest, and the promotional need for Caring.
- H-CV4. The personality trait of Conscientiousness will be significantly associated with the promotional need to fulfill one’s personal Potential.
- H-CV5. The personality trait of Neuroticism will be significantly associated with the prevention needs to feel less Unsafe or Anxious, and less Uncared for.
- H-CV6. Level of Depression will be significantly associated with prevention needs to feel less Unsafe or Anxious, and less Uncared for.
- H-CV7. Self-rated Work Performance will be significantly associated with the promotional need to fulfill one’s personal Potential.

### Concurrent validity results

Linear regression was conducted using image selections summed by discrete motivation, separately for promotion and prevention of each motive, as independent variables. All dependent variables are continuous variables named alongside each hypothesis number. The results of these analyses are summarized in Table 8; the full output is available in Supplementary Material Table 8.

**Table 8:** Summary of regression analyses employing image selection data to predict subject characteristics

Hypothesis	ANOVA	Significant predictors (linear regression)
CV-1. Openness to Experience ➔ Authenticity (A2P) ^a^ & Immersion (B2P) ^b^	F = 2.684, 22 df, *p* = .000	Authenticity (A2P; β = 2.096, *p* = 0.036) ^a^
		Success (B3P; β = 2.644, *p* = 0.008)
		Exclusion (C1N; β = -2.266, *p* = 0.024)
		Wrongdoing (D2N; β = 2.154, *p* = 0.032)
CV-2. Extraversion ➔ Inclusion (C1P) ^a^	F = 3.231, 22 df, *p* = .000	Inclusion (C1P; β = 3.428, *p* = 0.001) ^a^ Recognition (C3P; β = 4.044, *p* = 0.000) Uncaring (C2N; β = -3.910, *p* = 0.000) Anxiety (A1N; β = 2.379, *p* = 0.018)
CV-3. Agreeableness ➔ Caring (C2P) ^a^ & Ethics (D2P) ^a^	F = 1.716, 22 df, *p* = .021	Ethics (D2P; β = 2.561, *p* = 0.011) ^a^
		Caring (C2P; (β = -2.097, *p* = 0.036) ^a^
		Materialism (D3N; β = 2.860, *p* = 0.004)
CV-4. Conscientiousness ➔ Potential (A3P) ^a^	F = 2.451, 22 df, *p* = .000	Potential (A3P; β = 2.583, *p* = 0.010) ^a^
		Limitation (A3N; β = 1.988, *p* = 0.047)
		Stagnation (B2N; β = -1.968, *p* = 0.049) Inclusion (C1P; β = 2.420, *p* = 0.016) Recognition (C3P; β = 2.223, *p* = 0.026) Purpose (D3P; β = -2.174, *p* = 0.030)
CV-5. Neuroticism ➔ Anxiety (A1N) ^b^ & Uncaring (C2N) ^a^	F = 5.237, 22 df, *p* = .000	Uncaring (C2N; β = 4.759, *p* = 0.000) ^a^
		Caring (C2P; β = 2.875, *p* = 0.004)
		Inclusion (C1P; β = -2.044, *p *= 0.041)
		Recognition (C3P; β = -2.010, *p* = 0.045) Ethics (D2P; β = -2.868, *p* = 0.004)
		Stagnation (B2N; β = 2.370, *p* = 0.018)
		Scorn (C3N; β = 3.404, *p* = 0.001)
		Materialism (D3N; β = -2.021, *p* = 0.044)
CV-6. Depression ➔ Anxiety (A1N) ^a^ & Uncaring (C2N) ^a^	F = 9.904, 22 df, *p* = .000	Anxiety (A1N; β = 2.991, *p* = 0.003) ^a^
		Uncaring (C2N; β = 6.274, *p* = 0.000) ^a^
		Scorn (C3N; β = 3.313, *p* = 0.001)
		Materialism (D3N; β = -2.354, *p* = 0.019) Safety (A1P; β = -2.463, *p* = 0.014)
		Inclusion (C1P; β = -2.409, *p* = 0.016)
		Ethics (D2P; β = -3.267, *p* = 0.001)
		Purpose (D3P; β = 2.049, *p* = 0.041)
CV-7. Work Performance ➔ Potential (A3P) ^a^	F = 2.298, 22 df, *p* = .001	Potential (A3P; β = 2.432, *p* = 0.015) ^a^ Conformity (A2N; β = -2.360, *p* = 0.018) Exclusion (C1N; β = -2.111, *p* = 0.035)
		Scorn (C3N; β = -2.191, *p* = 0.029)
		Materialism (D3N; β = 1.976, *p* = 0.048)

^a^Findings support hypothesis

^b^Findings do not support hypothesis

#### Hypothesis CV-1: openness to experience

Linear regression predicting the level of Openness to Experience produced a significant ANOVA (F = 2.684, 22 *df*, *p* = 0.000). The promotional need for Authenticity (A2P) emerged as one of only four significant predictors (β = 2.096, *p* = 0.036). The other significant predictors included the promotional need for Success (B3P; β = 2.644, *p* = 0.008) and prevention needs for Inclusion (C1N; β = -2.266, *p* = 0.024) and Ethics (D2N; β = 2.154, *p* = 0.032).

The hypothesized relationship between Openness and Immersion was not supported (B2P; β = 0.296, *p* = 0.767), however, the hypothesized relationship between Openness and Authenticity was supported, providing partial support for the H CV-1. The finding of a significant *negative* relationship between prevention needs for Inclusion and Openness to Experience is intuitively sensible: Those who are more Open to Experience tend to be less concerned about social exclusion.

#### Hypothesis CV-2: extraversion

Linear regression predicting the level of Extraversion produced a significant ANOVA (F = 3.231, 22 *df*, *p* = 0.000). The promotional need for Inclusion (C1P) emerged as one of only four significant predictors (β = 3.428, *p* = 0.001). The other significant predictors included the promotional need for Recognition (C3P; β = 4.044, *p* = 0.000) and prevention needs for Caring (C2N; β = -3.910, *p* = 0.000) and Safety (A1N; β = 2.379, *p* = 0.018).

The hypothesized relationship (H CV-2) between Extraversion and Inclusion was supported. The finding of greater need for Recognition, another social need, among Extraverts is logically consistent with the need for Inclusion finding. The finding of a significant *negative* relationship between prevention needs for Caring and Extraversion is reasonable: Those who are more Extraverted tend to be less concerned about being uncared for presumably because of their greater social success.

#### Hypothesis CV-3: agreeableness

Linear regression predicting the level of Agreeableness produced a significant ANOVA (F = 1.716, 22 *df*, *p* = 0.021). The promotional need for Ethics (D2P) emerged as a significant predictor (β = 2.561, *p* = 0.011). The promotional need for Caring was also a significant predictor (C2P; (β = -2.097, *p* = 0.036), although this relationship is negative and therefore not predicted. The other significant predictor is the prevention need for Purpose (D3N; β = 2.860, *p* = 0.004).

The hypothesized relationship (H CV-3) between Agreeableness and Ethics was supported, although the hypothesized relationship with Caring was not, providing partial support overall. The finding of greater prevention needs for Purpose (i.e., relief from a sense of Materialism) among those high in Agreeableness is logically consistent with the need for Ethics finding, as both needs reflect an impulse toward selflessness.

#### Hypothesis CV-4: conscientiousness

Linear regression predicting the level of Conscientiousness produced a significant ANOVA (F = 2.451, 22 *df*, *p* = 0.000). The promotional need for Potential (A3P) emerged as a significant predictor (β = 2.583, *p* = 0.010), as did the prevention need for Potential (A3N; i.e., relief from limitation; β = 1.988, *p* = 0.047). Four other needs emerged as significant predictors: the prevention need for Immersion (B2N; i.e., relief from boredom; β = -1.968, *p* = 0.049), as well as promotional needs for Inclusion (C1P; β = 2.420, *p* = 0.016), Recognition (C3P; β = 2.223, *p* = 0.026), and Purpose (D3P; β = -2.174, *p* = 0.030).

The hypothesized relationship (H CV-4) between Conscientiousness and Potential was supported in terms of both promotion and prevention needs. The finding of an inverse relationship of Conscientiousness and the need for relief from boredom makes intuitive sense, as the conscientious presumably tend to stay busy. The finding of significant positive relationships between Conscientiousness and the social needs for Inclusion and Recognition similarly are sensible to the extent that Consciousness implies a commitment or duty to others.

#### Hypothesis CV-5: neuroticism

Linear regression predicting the level of Neuroticism produced a significant ANOVA (F = 5.237, 22 *df*, *p* = 0.000). Eight needs emerged as significant predictors of Neuroticism, including both promotion needs for Caring (C2P; β = 2.875, *p* = 0.004) and prevention needs for Caring (C2N; β = 4.759, *p* = 0.000), as stated in Hypothesis CV-5. The remaining significant predictors included *inverse* relations with promotion needs for social Inclusion (C1P; β = -2.044, *p* = 0.041), Recognition (C3P; β = -2.010, *p* = 0.045), and Ethics (D2P; β = -2.868, *p* = 0.004), and prevention needs for Immersion (B2N; i.e., relief from boredom; β = 2.370, *p* = 0.018), Recognition (C3N; β = 3.404, *p* = 0.001), and Purpose (D3N; β = -2.021, *p* = 0.044). The hypothesized relationship (H CV-5) between Neuroticism and Uncaring was supported in terms of both promotion and prevention needs, however, the relationship between Neuroticism and Anxiety was not supported; the remaining predictors were not hypothesized.

#### Hypothesis CV-6: depression

Linear regression predicting the level of Depression produced a significant ANOVA (F = 9.904, 22 *df*, *p* = 0.000). Similar to the related concept of Neuroticism, eight needs emerged as significant predictors, including prevention needs for less Anxiety (A1N; β = 2.991, *p* = 0.003) and Uncaring (C2N; β = 6.274, *p* = 0.000) in support of Hypothesis CV-6. Other significant prevention needs include the need for less Scorn (C3N; β = 3.313, *p* = 0.001) and Materialism (D3N; β = -2.354, *p* = 0.019). Promotion needs significantly predicting depression level included the *inverse* needs for Safety (A1P; β = -2.463, *p* = 0.014), Inclusion (C1P; β = -2.409, *p* = 0.016), and Ethics (D2P; β = -3.267, *p* = 0.001), as well as the need for Purpose (D3P; β = 2.049, *p* = 0.041).

#### Hypothesis CV-7: work performance

Linear regression predicting the level of self-rated Work Performance [70] produced a significant ANOVA (F = 2.298, 22 *df*, p = 0.001). Five needs emerged as significant predictors of performance including the hypothesized promotional need for Potential (A3P; β = 2.432, p = 0.015), the only significant promotion-facing predictor. Three of the four remaining significant predictors were inverse relationships with prevention needs, meaning that work performance is associated with the *absence* of relief needs for Authenticity (A2N; i.e., relief from conformity pressures; β = -2.360, *p* = 0.018), Inclusion (C1N; i.e., relief from exclusion; β = -2.111, *p* = 0.035), and Recognition (C3N; i.e., relief from scorn; β = -2.191, *p* = 0.029), a sensible finding. The final significant predictor is the prevention need for Purpose (D3N; i.e., relief from materialism; β = 1.976, *p* = 0.048).

In the case of each hypothesis, our approach demonstrated at least partial concurrent validity, demonstrating the ability to discriminate distinct motivations (Table 8):

- H CV-1: The hypothesized association of Openness to Experience with the need for Authenticity was supported, although the predicted association with need for Immersion was not.
- H CV-2: The hypothesized association of Extraversion with the need for Inclusion was supported.
- H CV-3: The hypothesized association of Agreeableness with the need for Ethics was supported, although the predicted association with the need for Caring was not.
- H CV-4: The hypothesized association of Conscientiousness with the need for Potential was supported.
- H CV-5: The hypothesized association of Neuroticism with the need to lessen feelings of Uncaring was supported, although the relationship with Anxiety was not supported.
- H CV-6: The hypothesized associations of Depression with the need to lessen feelings of Uncaring and Anxiety were both supported.
- H CV-7: The hypothesized association of Work Performance with the need to fulfill one’s Potential was supported.

In sum, of the eleven hypothesized relationships, eight were supported by the analyses.

## Assessing the model’s structure using image selection data

The structure of our theoretical model was tested using multiple methods using the latest data collected, which is also the largest dataset collected to date (June 2023, *n* = 986). CFA was used to determine the overall fit of the model’s assumptions about life domains (Hypotheses 1.1 – 1.4), levels of attainment (Hypotheses 2.1 – 2.3), and the antipodal nature of the Self vs. Social domain and the Material vs. Spiritual domain (Hypotheses 3.1 – 3.6). Canonical correlation analysis was used to test hypotheses regarding specific pairings of motives. Those pairs hypothesized to be relatively *stronger* are those falling in the same domain, e.g., the needs for inclusion and caring both appear in the Social domain, or level of attainment, e.g., the needs for safety and justice both appear in the Foundational level of attainment. Those pairs hypothesized to be relatively *weaker* are those that cross domains and levels of attainment, e.g., the needs for authenticity and success, or appear in antipodal domains, e.g., the needs for potential (Self) and recognition (Social).

## Confirmatory factor analysis results

We tested the hypothesized factor structure of the framework using confirmatory factor analysis (CFA), using image sets to represent each of the 12 cells of the model. CFA was chosen to test the model because the model is grounded in theory, in terms of the factors and the components that underlie them, as well as the hypothesized relationships between the factors. The purpose of the CFA was to test the goodness of fit of our model to the data. The determination of model fit was based on a comparison of several fit indices following generally accepted standards [71]. All analyses were conducted with JASP software, version 0.14.1 [72]. Because JASP requires the use of continuous variables for estimating CFA models, the latency of image selections (i.e., continuous) was used instead of binary selection variables.

Because of the known issues associated with combining positive and negative emotions in factor analyses, separate models were estimated for positive (promotion-focused) and negative (prevention-focused) motivations. To estimate the broadest model, all four domains (Self, Material, Social, Spiritual) were simultaneously modeled as first-order latent factors in overall models, separately for promotion and prevention motivation. Going a level deeper, separate models were estimated using second-order latent factors with the three corresponding motivations set as first order latent factors within each, with corresponding image variables set as indicators of each latent motivation. We have reported chi-square and a number of alternative fit indices including relative fit indices such as Tucker-Lewis fit index (TLI), and two absolute fit indices, the root mean-square error of approximation (RMSEA), and the standardized root mean square (SRMR), summarized in Tables 9, 10 and 11; the full output is available in Supplementary Material Tables 9.1, 9.2, 10.1, 10.2, 11.1, and 11.2.

**Table 9:** Confirmatory factor models for the four life domains

	Promotion motivation					Prevention motivation				
	Overall	Self	Material	Social	Spiritual	Overall	Self	Material	Social	Spiritual
Chi-square	1946.318, 588 *df*, *p* < .001	66.375, 24 *df*, *p* < .001	41.291, 24 *df*, *p* = 0.015	30.063, 24 *df*, *p* = 0.026	50.010, *df* 24, *p* < 0.001	1338.450, 588 *df*, *p* < .001	40.735, 24 *df*, *p* < .001	31.052, 24 *df*, *p* = 0.152	28.684, 24 *df*, *p* = 0.232	35.711, 24 *df*, *p* = 0.059
TLI	0.900	0.863	0.935	0.962	0.938	0.950	0.929	0.987	0.995	0.981
RMSEA	0.047	0.041	0.026	0.027	0.032	0.035	0.037	0.017	0.014	0.022
SRMR	0.040	0.036	0.027	0.024	0.031	0.027	0.033	0.021	0.019	0.023
Minimum acceptance fit thresholds exceeded	3	2	3	3	3	3	3	4	4	4

**Table 10:** Confirmatory factor models for the three levels of attainment

	Promotion motivation				Prevention motivation			
	Overall	Foundational	Experiential	Aspirational	Overall	Foundational	Experiential	Aspirational
Chi-square	1818.986, 557 *df*, *p* < .001	87.709, 50 *df*, *p* < .001	120.171, 50 *df*, *p* < 0.001	83.479, 50 *df*, *p* < .001	1330.930, 557 *df*, *p* < .001	74.505, 50 *df*, *p* = .014	97.702, 50 *df*, *p* < .001	100.733, 50 *df*, *p* < .001
TLI	0.900	0.925	0.889	0.926	0.950	0.976	0.946	0.952
RMSEA	0.047	0.027	0.037	0.032	0.037	0.022	0.030	0.031
SRMR	0.040	0.030	0.036	0.032	0.028	0.026	0.036	0.032
Minimum acceptance fit thresholds exceeded	3	3	2	3	3	3	3	3

**Table 11:** Confirmatory factor models for adjacent and antipodal life domains

	Promotion motivation						Prevention motivation					
	Self-Material	Material-Social	Social-Spiritual	Spiritual-Self	Self-Social	Material-Spiritual	Self-Material	Material-Social	Social-Spiritual	Spiritual-Self	Self-Social	Material-Spiritual
Chi-square (all 133 *df*, *p* < 0.001)	462.225	406.902	502.737	575.644	398.411	465.046	365.692	370.828	470.785	407.218	467.834	296.125
TLI	0.683	0.799	0.754	0.630	0.776	0.708	0.875	0.893	0.855	0.857	0.852	0.912
RMSEA	0.049	0.045	0.052	0.057	0.044	0.049	0.041	0.042	0.050	0.045	0.049	0.034
SRMR	0.049	0.045	0.049	0.054	0.046	0.047	0.040	0.039	0.046	0.041	0.044	0.035
Minimum acceptance fit thresholds exceeded	2	2	1	0	2	2	2	2	1	2	2	3

### *Promotion-oriented* models of life domains

The overall promotion-oriented life domain model produced a significant chi-square (1946.318, 588 *df*, *p* < 0.001) suggesting that the model did not perfectly fit the data. However, the model produced a TLI of 0.895, falling short of the minimum acceptance threshold for marginal fit [64, 73]. The model produced an RMSEA of 0.047 (0.045 LCI; 0.050 UCI), which fell below the standard of 0.05 as indicating good fit [66, 74]. The model produced an SRMRs of 0.040, which fell below the 0.08 cutoff [67, 75], again suggesting good fit. On this basis, with two of three fit indices having met minimum thresholds, we infer that the overall promotion model for life domains demonstrates moderate fit to the data, providing modest support for Hypotheses 1.1 – 1.4.

The four separate factor models produced significant chi-squares for each of the four domains, respectively (Self: 66.375, 24 *df*, *p* < 0.001; Material: 41.291, 24 *df*, *p* = 0.015; Social: 30.063, 24 *df*, *p* = 0.026; Spiritual: 50.010, 24 *df*, *p* < 0.001) suggesting that the model did not perfectly fit the data. However, three of the four models produced TLIs above 0.90, the minimum acceptance criterion for marginal fit [64, 73], and one, the Social domain, produced a TLI of 0.962, exceeding the accepted cutoff of 0.95 indicating good fit [65, 76]. The models produced RMSEAs of Self: 0.041 (0.030 LCI; 0.053 UCI); Material: 0.026 (0.012 LCI, 0.040 UCI); Social: 0.027 (0.009 LCI, 0.043 UCI); Spiritual: 0.032 (LCI 0.020, 0.045 UCI), each falling below the standard of 0.05 as indicating good fit [66, 74]. The models produced SRMRs of Self: 0.036; Material: 0.027; Social: 0.024; and Spiritual: 0.031, each falling below the 0.08 cutoff [67, 75], again suggesting good fit. On this basis, with all three fit indices having met minimum thresholds, we infer that the models for the Material, Social, and Spiritual domains demonstrate good fit to the data in support of Hypotheses 1.2, 1.3, and 1.4, respectively. The Self domain produced acceptance fit metrics for RMSEA and SRMR but not TLI (Table 9), which failed to support Hypothesis 1.1. Supplementary Material Table 9.1 provides the factor loadings for each indicator of each factor.

### *Prevention-oriented* models of life domains

The overall prevention-oriented life domain model produced a significant chi-square (1338.450, 588 *df*, *p* < 0.001) suggesting that the model did not perfectly fit the data. However, the model produced a TLI of 0.95, which meets the criterion for good fit [65, 76]. The model produced an RMSEA of 0.035 (0.033 LCI; 0.038 UCI), which fell below the standard of 0.05 as indicating good fit [66, 76]. The model produced an SRMRs of 0.027, which fell below the 0.08 cutoff [67, 75], again suggesting good fit. On this basis, with all three fit indices having met minimum thresholds, we infer that the overall prevention model for life domains demonstrates good fit to the data, providing overall support for Hypotheses 1.1 – 1.4.

The four separate factor models produced significant chi-squares for only one of the four domains (Self: 40.735, 24 *df*, *p* < 0.001; Material: 31.052, 24 *df*, *p* = 0.152; Social: 28.684, 24 *df*, *p* = 0.232; Spiritual: 35.711, 24 *df*, *p* = 0.059) suggesting that three of the four models fit the data extremely well. All four models produced TLIs above 0.90, the minimum acceptance criterion for marginal fit [64, 73], and two, the Social domain and the Spiritual domain, produced TLIs of 0.995 and 0.981, respectively, exceeding the accepted cutoff of 0.95 [65, 76], indicating good fit. The models produced RMSEAs of Self: 0.037 (0.022 LCI; 0.051 UCI); Material: 0.017 (0.000 LCI, 0.032 UCI); Social: 0.014 (0.000 LCI, 0.030 UCI); Spiritual: 0.022 (LCI 0.000, 0.036 UCI), each falling below the standard of 0.05 as indicating good fit [66, 74]. The models produced SRMRs of Self: 0.036; Material: 0.021; Social: 0.019; and Spiritual: 0.023, each falling below the 0.08 cutoff [67, 75], again suggesting good fit. On this basis, with all three fit indices having met minimum thresholds, we infer that the models for all four domains demonstrate good fit to the data (Table 9), providing support for Hypotheses 1.1 – 1.4. Supplementary Material Table 9.2 provides the factor loadings for each indicator of each factor.

### *Promotion* models of levels of attainment

The overall promotion-oriented level of attainment model produced a significant chi-square (1818.986, 587 *df*, *p* < 0.001) suggesting that the model did not perfectly fit the data. Additionally, the model produced a TLI of 0.899, just below the minimum acceptance criterion for marginal fit [64, 73]. The model produced an RMSEA of 0.047 (0.044 LCI; 0.049 UCI), which fell below the standard of 0.05 as indicating good fit [66, 74]. The model produced an SRMRs of 0.040, which fell below the 0.08 cutoff [67, 75], again suggesting good fit. On this basis, with two of three fit indices having met minimum thresholds, we infer that the overall promotion model for levels of attainment demonstrates moderate fit to the data, which provides modest support for Hypotheses 2.1 – 2.3.

The four separate factor models produced significant chi-squares for each of the three levels of attainment, respectively (Foundational: 87.709, 50 *df*, *p* < 0.001; Experiential: 120.171, 50 *df*, *p* < 0.001; Aspirational: 83.479, 50 *df*, *p* < 0.001) suggesting that the model did not perfectly fit the data. However, two of the three models produced TLIs above 0.90, the minimum acceptance criterion for marginal fit [64, 73]; the Experiential model’s TLI fell below 0.90 indicating poor fit. The models produced RMSEAs of Foundational: 0.027 (0.017 LCI; 0.036 UCI); Experiential: 0.037 (0.028 LCI, 0.045 UCI); Aspirational: 0.032 (0.023 LCI, 0.042 UCI); each falling below the standard of 0.05 as indicating good fit [66, 74]. The models produced SRMRs of Foundational: 0.030; Experiential: 0.036; and Aspirational: 0.032, each falling below the 0.08 cutoff, again suggesting good fit [67, 75]. On this basis, with all three fit indices having met minimum thresholds, we infer that the models for the Foundation and Aspirational levels of attainment demonstrate good fit to the data, providing support for Hypotheses 2.1 and 2.3. The Experiential level of attainment domain produced acceptance fit metrics for RMSEA and SRMR but not TLI (Table 10), which fails to support Hypothesis 2.2. Supplementary Material Table 10.1 provides the factor loadings for each indicator of each factor.

### *Prevention* models of levels of attainment

The overall prevention-oriented level of attainment model produced a significant chi-square (1330.930, 587 *df*, *p* < 0.001) suggesting that the model did not perfectly fit the data. However, the model produced a TLI of 0.95, which meets the criterion for good fit [65, 76]. The model produced an RMSEA of 0.037 (0.034 LCI; 0.039 UCI), which fell below the standard of 0.05 as indicating good fit [66, 74]. The model produced an SRMRs of 0.028, which fell below the 0.08 cutoff [67, 75], again suggesting good fit. On this basis, with all three fit indices having met minimum thresholds, we infer that the overall prevention model for levels of attainment demonstrates good fit to the data, providing overall support for Hypotheses 2.1 – 2.3.

The four separate factor models produced significant chi-squares for all three levels of attainment (Foundational: 74.505, 50 *df*, *p* = 0.014; Experiential: 97.702, 50 *df*, *p* < 0.001; Aspirational: 100.733, 50 *df*, *p* < 0.001) suggesting that the three models did not perfectly fit the data. All three of the models produced TLIs above 0.90, the minimum acceptance criterion for marginal fit [64, 73], and two, the Foundational and the Aspirational domain, produced TLIs of 0.976 and 0.952, respectively, exceeding the accepted cutoff of 0.95, indicating good fit [65, 76]. The models produced RMSEAs of Foundational: 0.022 (0.010 LCI; 0.032 UCI); Experiential: 0.030 (0.021 LCI, 0.039 UCI); Aspirational: 0.031 (0.022 LCI, 0.040 UCI), each falling below the standard of 0.05 as indicating good fit [66, 74]. The models produced SRMRs of Foundational: 0.026; Experiential: 0.036; and Aspirational: 0.032, each falling below the 0.08 cutoff, again suggesting good fit [67, 75]. On this basis, with all three fit indices having met fit thresholds, we infer that all models for the three levels of attainment demonstrate good fit to the data (Table 10), providing support for Hypotheses 2.1 – 2.3. Supplementary Material Table 10.2 provides the factor loadings for each indicator of each factor.

### Second order *promotion* models of adjacent vs. antipodal life domains

To test the antipodal nature of the Self vs. Social domain and the Material vs. Spiritual domain (Hypotheses 3.1 – 3.6), second order factors were defined by the integration of each of the four life domains in pairs, representing six possible combinations: Four are adjacent in the model: Self-Material, Material-Social, Social-Spiritual, Spiritual-Self; and two are antipodal: Self-Social, Material-Spiritual. The factor models produced significant chi-squares for each of the six pairings (Self-Material: 462.225, 133 *df*, *p* < 0.001; Material-Social: 406.902, 133 *df*, *p* < 0.001; Social-Spiritual: 502.737, 133 *df*, *p* < 0.001; Spiritual-Self: 575.644, *df* 133, *p* < 0.001; Self-Social: 398.411, *df* 133, *p* < 0.001; Material-Spiritual: 465.046, *df* 133, *p* < 0.001) suggesting that none of these models perfectly fit the data. Similarly, *none* of these models produced TLIs above 0.90, the minimum acceptance criterion for marginal fit [64, 73]. The models produced RMSEAs of Self-Material: 0.049 (0.044 LCI; 0.054 UCI); Material-Social: 0.045 (0.040 LCI, 0.050 UCI); Social-Spiritual: 0.052 (0.047 LCI, 0.057 UCI); Spiritual-Self: 0.057 (LCI 0.052, 0.062 UCI); Self-Social: 0.044 (LCI 0.039, 0.049 UCI); Material-Spiritual 0.049 (LCI 0.044, 0.054 UCI). Four of the six fall below the standard of 0.05 as indicating good fit [66, 74]. The models produced SRMRs of Self-Material: 0.049; Material-Social: 0.045; Social-Spiritual: 0.049; Spiritual-Self: 0.054; Self-Social: 0.046; Material-Spiritual: 0.047, each falling below the 0.08 cutoff, suggesting good fit [67, 75]. On this basis, with none of the models meeting the relative fit threshold and two of the six failing to meet the RMSEA threshold, we infer that these models do not demonstrate good fit to the data. Although demonstrating poor fit, the best fitting models were the antipodal Self-Social, a result counter to Hypothesis 3.6, and the adjacent Material-Social, providing support for Hypothesis 3.3 Because of the lack of consistency in these results, we do not feel that any of Hypotheses 3.1 – 3.6 are supported (Table 11). Supplementary Material Table 11.1 provides the factor loadings for each indicator of each factor.

### Second order *prevention* models of adjacent vs. antipodal life domains

The preceding analysis was repeated for prevention motivation factors. The factor models produced significant chi-squares for each of the six pairings (Self-Material: 365.692, 133 *df*, *p* < 0.001; Material-Social: 370.828, 133 *df*, *p* < 0.001; Social-Spiritual: 470.785, 133 *df*, *p* < 0.001; Spiritual-Self: 407.218, *df* 133, *p* < 0.001; Self-Social: 467.834, *df* 133, *p* < 0.001; Material-Spiritual: 296.125, *df* 133, *p* < 0.001) suggesting that none of these models perfectly fit the data. Similarly, five of these six models produced TLIs below 0.90, the minimum acceptance criterion for marginal fit [64, 73]; the one model exceeding this standard is the antipodal Material-Spiritual factor. The models produced RMSEAs of Self-Material: 0.041 (0.036 LCI; 0.046 UCI); Material-Social: 0.042 (0.037 LCI, 0.047 UCI); Social-Spiritual: 0.050 (0.045 LCI, 0.055 UCI); Spiritual-Self: 0.045 (LCI 0.040, 0.050 UCI); Self-Social: 0.049 (LCI 0.045, 0.054 UCI); Material-Spiritual 0.034 (LCI 0.029, 0.040 UCI). Five of the six fall below the standard of 0.05 as indicating good fit [66, 74], the lone exception being the adjacent Social-Spiritual. The models produced SRMRs of Self-Material: 0.040; Material-Social: 0.039; Social-Spiritual: 0.046; Spiritual-Self: 0.041; Self-Social: 0.044; Material-Spiritual: 0.035, each falling below the 0.08 cutoff, suggesting good fit [67, 75]. One of the models, the antipodal Material-Spiritual factor, meets all three standards; the remaining five do not demonstrate good fit to the data (Table 11), a result that runs counter to Hypothesis 3.6. Because of the lack of consistency in these results, we do not feel that any of Hypotheses 3.1 – 3.6 are supported. Supplementary Material Table 11.2 provides the factor loadings for each indicator of each factor.

## Canonical correlation results

Canonical correlations were conducted using the sets of image variables grouped by motive construct, to evaluate the multivariate shared relationship between variable sets (e.g., Inclusion and Caring) to test the model’s structural assumptions. Because there are 12 constructs, a total of 66 canonical correlations were possible. Pairs of constructs were coded as either predicted or non-predicted by the structure of the proposed theoretical model in terms of their horizontal (row) or vertical (column) adjacencies. Predictions are based on whether each pairing falls within the same domain, adjacent domains, or in antipodal domains (Table 12). The 24 predicted pairs (36 percent of total pairings) are composed of the three pairs that exist between the three motives within each of the four domains (for a subtotal of 12) and the three pairs that exist between the four motives at each of the three levels (for a subtotal of 12) when the six same-row antipodes are removed. The remaining 42 pairs (63 percent of total pairs) are predicted to share less variance. These specific hypotheses are presented above as Hypotheses 1.1 – 1.4, 2.1 – 2.3, and 3.1 – 3.6.

**Table 12:** Standardized canonical function coefficients for each construct pair

Set 1	Set 2	Canonical Correlation (all* p* < .000)	Average shared variance	Predicted (P), non-predicted (N)	Same domain (S), Adjacent (A), Antipodal (L)
Safety	Authenticity	0.576	13.5%	P	S
Safety	Potential	0.649	16.0%	P	S
Safety	Autonomy	0.654	17.5%	P	A
Safety	Immersion	0.569	13.0%	N	A
Safety	Success	0.633	16.5%	N	A
Safety	Inclusion	0.611	15.5%	N	L
Safety	Caring	0.635	16.0%	N	L
Safety	Recognition	0.627	16.5%	N	L
Safety	Justice	0.633	17.5%	P	A
Safety	Ethics	0.564	13.5%	N	A
Safety	Transcendence	0.628	15.5%	N	A
Authenticity	Potential	0.539	11.0%	P	S
Authenticity	Autonomy	0.526	11.5%	N	A
Authenticity	Immersion	0.484	9.5%	P	A
Authenticity	Success	0.52	11.5%	N	A
Authenticity	Inclusion	0.513	11.0%	N	L
Authenticity	Caring	0.534	11.5%	N	L
Authenticity	Recognition	0.501	10.5%	N	L
Authenticity	Justice	0.532	11.5%	N	A
Authenticity	Ethics	0.622	14.5%	P	A
Authenticity	Transcendence	0.626	14.5%	N	A
Potential	Autonomy	0.703	19.5%	N	A
Potential	Immersion	0.628	15.0%	N	A
Potential	Success	0.63	16.0%	P	A
Potential	Inclusion	0.567	13.0%	N	L
Potential	Caring	0.613	50.5%	N	L
Potential	Recognition	0.594	52.5%	N	L
Potential	Justice	0.562	41.0%	N	A
Potential	Ethics	0.507	43.5%	N	A
Potential	Transcendence	0.636	38.5%	P	A
Autonomy	Immersion	0.684	33.5%	P	S
Autonomy	Success	0.685	29.0%	P	S
Autonomy	Inclusion	0.625	43.0%	P	A
Autonomy	Caring	0.645	45.5%	N	A
Autonomy	Recognition	0.65	44.5%	N	A
Autonomy	Justice	0.582	33.0%	N	L
Autonomy	Ethics	0.54	39.5%	N	L
Autonomy	Transcendence	0.609	28.5%	N	L
Immersion	Success	0.682	40.0%	P	S
Immersion	Inclusion	0.643	29.5%	N	A
Immersion	Caring	0.628	26.0%	P	A
Immersion	Recognition	0.661	28.0%	N	A
Immersion	Justice	0.543	26.5%	N	L
Immersion	Ethics	0.564	27.0%	N	L
Immersion	Transcendence	0.549	27.0%	N	L
Success	Inclusion	0.64	38.5%	N	A
Success	Caring	0.634	28.0%	N	A
Success	Recognition	0.691	34.0%	P	A
Success	Justice	0.564	30.5%	N	L
Success	Ethics	0.572	27.0%	N	L
Success	Transcendence	0.571	36.5%	N	L
Inclusion	Caring	0.671	56.5%	P	S
Inclusion	Recognition	0.686	55.5%	P	S
Inclusion	Justice	0.612	45.0%	P	A
Inclusion	Ethics	0.558	44.5%	N	A
Inclusion	Transcendence	0.532	44.0%	N	A
Caring	Recognition	0.698	60.0%	P	S
Caring	Justice	0.595	42.0%	N	A
Caring	Ethics	0.619	49.0%	P	A
Caring	Transcendence	0.619	36.0%	N	A
Recognition	Justice	0.605	39.5%	N	A
Recognition	Ethics	0.641	44.5%	N	A
Recognition	Transcendence	0.574	37.5%	P	A
Justice	Ethics	0.571	54.0%	P	S
Justice	Transcendence	0.573	42.5%	P	S
Ethics	Transcendence	0.616	43.0%	P	S

In all comparisons, the first CCA functions yielded both statistical significance and a substantial amount of shared variance and were therefore included in subsequent analyses following best practices [55, 77]; all subsequent functions, which were created from residual variance after the extraction of the first functions, did not meet these requirements and will not be further explored. Examination of the standardized canonical function coefficients and structure coefficients for all functions revealed item coefficients that conform to the expected pattern, suggesting that their meanings were retained, as expected. Because the goal of this exercise is to evaluate the macro structure of the proposed model, we will not delve into the specifics of these coefficients but will rather focus on comparisons of the canonical correlations themselves, their significance and amount of shared variance explained for the relationships postulated to be stronger or weaker by the model.^2^ Table 12 presents the standardized canonical function coefficients for each first function.

The coefficients range from 0.484 to 0.703, all significant at *p* = 0.00002; using a standard interpretation of correlation strength [69, 78], all correlations are of at least *moderate* strength (± 0.4 to 0.6) and many are *strong* (± 0.6 to 0.8). Twenty-two pairs shared variance of at least 40 percent; these pairings show a pattern of connections that conforms to those predicted by the model: We see relatively strong connections between motives of the Self domain with both the Material and Spiritual domains, but not the Social domain, providing support for Hypotheses 3.2, 3.3, and 3.5, respectively. We also see relatively strong connections between motives of the Material domain with the domains of the Self and Social, but not the Spiritual domain, providing support for Hypotheses 3.1, 3.3, and 3.6.

These observations are borne out statistically. The correlation between the binary prediction variable (predicted = 1, not predicted = 0) and magnitude of coefficient is 0.25 (*p* = 0.0345), suggesting that predicted relationships have stronger interrelationships than non-predicted relationships. Indeed, pairs falling in the same life domain show the strongest average canonical correlation and percentage of variance explained (*r* = 0.64, 37.8% variance explained), pairs falling in adjacent life domains show middling results (*r* = 0.61, 28.8% variance explained), and pairs falling in antipodal domains show weakest association (*r* = 0.57, 26.2% variance explained). Demonstrated another way, of the ten strongest correlations (all *strong* correlations, 0.65 to 0.70), eight were predicted by our model, or 80 percent; of the fifteen weakest correlations (all of *moderate* strength, 0.48 to 0.56), none were predicted by our model (Table 13).

**Table 13:** Prevalence of predicted pairings by correlation strength bins

Correlation strength rank bins	% of observed correlations predicted by theoretical model
Top 10	80%
11 to 20	20%
21 to 30	50%
31 to 40	20%
41 to 50	10%
51 to 66	0%

## Discussion

CFA revealed that our model’s predictions regarding life domains, expressed as Hypotheses 1.1 through 1.4, are supported, with both *overall* promotion and prevention models tending to exceed minimum fit thresholds. A level of specificity lower, second order factors exceeded minimum thresholds for all four domains separately when prevention motivation is assessed, and for three of the four domains (all but the Self domain) when promotion motivation is assessed.

CFA also revealed that both *overall* promotion and prevention models for level of attainment, expressed in Hypotheses 2.1 through 2.3, are supported. A level of specificity lower, second order models are supported for all three levels when prevention motivation is assessed, and for two of the three levels (all but the Experiential level) when promotion motivation is assessed.

CFA results are more complicated when trying to test the structural assumptions of the model regarding adjacent and antipodal relationships between life domains, given in Hypotheses 3.1 through 3.6. In eleven cases out of twelve (the one exception being the Material-Spiritual second order factor when prevention motivation is assessed, a finding that is *counter* to our hypothesis), CFA models did not meet relative fit thresholds (TLI), suggesting that, in general, “hybrid factors” composed of more than one motivation, are not supported by the data. This finding, in itself, is supportive of our model as it demonstrates the distinctiveness of each of the four life domains.

To further investigate the structural assumptions of the model, we examined patterns among the motive-to-motive canonical correlation coefficients. Results demonstrate that pairings predicted by the model are substantially over-represented relative to chance among the most correlated pairs, as indicated by the significant relationship between prediction status and correlation strength. Of the 66 total pairs, 6 shared at least 28% variance suggesting a strong degree of association. Of these, 5 (83%) of the pairs represented predicted pairings (i.e., falling within the same column, or positioned in adjacent columns at the same row-level of striving); this rate can be compared with an expected chance rate of 36%, a finding that provides support for the model’s structure; additionally, every one (100%) of the fifteen smallest correlations observed were *not* predicted by the model, which can be compared with an expected chance rate of 64%.

Analysis of the image data reveals three important supports for the proposed model.

Firstly, the emergence of each of the theoretical motives proposed by the unified model (H1.1 – 1.4), partially with respect to the domain of the Self, provides empirical support for their independent existence. Secondly, the emergence of each of the theoretical levels of attainment (H2.1 – 2.3), partially with respect to the Experiential level, similarly provides empirical support for the fundamental structure of the model. Thirdly, although the structural assumptions of the model (H3.1 – 3.6) could not be supported through CFA, and even produced a contrary result in the Material-Spiritual association, analysis of the canonical correlations between motives provides support for the theoretical structure of the model; associations predicted by the model are greatly over-represented relative to chance among the most interrelated pairs (83% for the pairs showing highest correlations, more than double the chance level). The convergence of these findings suggest that the theoretical model is not just intuitively appealing as a heuristic but may reflect deeper interrelationships between different types of motivation, at least regarding the distinctiveness of the four life domains and three levels of attainment. Future research is needed to establish the adjacency-antipodal hypotheses stated as H3.1—3.6. The repeated finding of support for a Material-Spiritual factor bears closer investigation. We hypothesize that although this dimension should be antipodal according to past theory, these motives may function together in more of a complementary manner.

## General discussion

We note that our approach runs counter to prevailing trends in theoretical development in motivational science, which can be summarized as “really, (my construct) is the only thing that matters.” In the place of *my construct*, we can substitute any set of popular motivational concepts, e.g., autonomy-competence-relatedness, growth, willpower, grit, drive, identity coherence, mindset, etc. The prevailing approach rewards researchers who wish to distinguish themselves and perhaps publish a book named for their construct. Yet, from the broader view of the development of science, reductionistic overvaluation of “parsimony” over true theoretical integration or synthesis tends to inspire researchers to proliferate new motivational concepts, usually existing concepts with new names, into an already crowded field. What the field cries out for is not more “blind men describing elephants,” but rather integrated frameworks that can specify the full range of possible human motivations and their boundaries, to curtail further proliferation and organize the concepts already described.

### Practical implications

With a comprehensive taxonomy and framework of human motivation in hand, the study of motivation becomes greatly simplified. Firstly, by placing constructs and their measures within the structure of the matrix, it becomes obvious which have been included or omitted, as well as the degree of representation given to each, providing the researcher opportunity to adjust the scope of inquiry toward greater comprehensiveness, or to clearly state their reasons for construct exclusion. Secondly, because the model provides testable propositions about the relationships between discrete motives, theory can develop toward more nuanced understandings of the relationships between motivation constructs. Here are a few of the many possible resulting questions:

- How is the need for fulfilling one’s potential related to the need for autonomy? By gaining greater mastery, can one gain greater independence?
- What is the nature of the relationship between the needs for safety and justice? Can we ever feel safe if we live in an unjust system?
- What is the relationship between the needs for caring and ethics? Does truly ethical conduct come from adherence to rules or a sense of philanthropy?
- What is the connection between the needs for success and recognition? Like a tree falling in the woods, is achievement less rewarding if no one hears about it?

When distinct profiles of motivation can be measured, relating these profiles to different antecedent conditions and to different behavioral outcomes can provide a basis for evaluating potential interventions. By establishing, for example, that young drivers have a profound need for immersion/excitement and authenticity/self-expression, safe driving campaigns can be designed to channel these needs toward healthier outlets than their potential expression in reckless driving. By establishing that warehouse workers have a strong unmet need for justice/fairness, company policies can be adjusted specifically to improve equitable treatment. By establishing that parents with unmet needs for childcare feel psychologically unsafe, interventions can be directed to improving real and felt safety. In every case, pre- and post-intervention measurement can determine the extent of remaining unmet need, providing an opportunity to adjust interventions. The list of potential applications of this approach is as varied as human experience.

### Limitations and implications for future research

Although results were generally supportive of our model, particular findings diverged from our hypotheses. A consistent finding was that the Self life domain did not perform as well as the Material, Social, and Spiritual domains across both promotion and prevention motivation assessments. Another consistent finding was that the Experiential level of attainment did not perform as well as the Foundational and Aspirational levels across the promotion and prevention conditions. We hypothesize that both findings may represent the inherent character of egocentric needs, on the one hand, and intermediate, experiential needs on the other. Regarding the levels of attainment, it seems very likely that “starting points” (Foundational needs) and “ending points” (Aspirational needs) are more clearly definable as needs than the “broad middle” that extends between them. By the same token, all the non-Self life domains represent interactions with things or ideas *outside the self*, and, hence, needs related to these external “objects” may be more easily defined than needs that relate to the Self. A key element may simply be the observer’s vantage point: We are much “further away” from the Material, Social, and Spiritual domains than we are from ourselves; consequently, the gaps between our Self-oriented Foundational needs and Experiential needs and Aspirational needs probably appear much larger (and therefore more different) because we are living *in them.*

Methodologically, a large body of evidence now demonstrates the validity and reliability of our image-based technique, with one notable exception, the failure of the CI_CFA_ discriminant analysis, which necessitates future work to more clearly differentiate the stimuli used to represent each construct. We also anticipate the need for further replication of our findings using global samples and different measurement methods that broadly cover the same range of motivational constructs. We foresee a need to use multiple measurement approaches that go beyond the limitations of scaled instruments. Although we employed image-based measures in these studies, there are many more methods available, particularly in the domain of affective research, e.g., affective priming, implicit association test, brain imaging, vocal pitch analysis, etc. Although the generality of the current results must be established by future research, the present study has provided support for the basic structure of the model. Not only did all four life domains and all three levels of attainment emerge as distinct factors, but the predicted linkages were strongly over-represented among the most highly intercorrelated pairings. We hope that the current research will stimulate further investigation of this important area, namely, understanding human motivation.

### Supplementary Information

**Additional file 1:** **SM Table 5.** Regression models: Image-based and established measures as predictors of burnout and intent to quit: Full output.

**Additional file 2:** **SM Table 6.7.** Discriminant validity analysis using CI_CFA_(sys): Full output.

**Additional file 3:** **SM Table 8.** Regression models: Image-based measures to predict subject characteristics: Full output.

**Additional file 4:** **SM Table 9.** Confirmatory factor models for the four life domains: Full output.

**Additional file 5:** **SM Table 10.** Confirmatory factor models for the three levels of attainment: Full output.

**Additional file 6:** **SM Table 11.** Confirmatory factor models for adjacent and antipodal life domains: Full output.

## Data Availability

All data and material are available from the author.

## Abbreviations

CFA: Confirmatory factor analysis
TLI: Tucker-Lewis Index
RMSEA: The root mean-square error of approximation
SRMR: Standardized root mean square
CCA: Canonical correlation analysis

## Footnotes

1 Note that similar predictions could be made for all 12 motivations, both their promotion and prevention faces, and these are planned for inclusion in future waves of research.

2 The full output for each canonical function is available from the author.
