# Supplementary material for: The structure of human motivation
Source: BMC Psychol. 2023 Oct 6;11:308. doi: 10.1186/s40359-023-01346-5 (PMC10557177; doi:10.1186/s40359-023-01346-5)
Supplement: Supplementary file 1 — Additional file 1: SM Table 5. Regression models: Image-based and established measures as predictors of burnout and intent to quit: Full output. [file 40359_2023_1346_MOESM1_ESM.docx]

Supplementary Material Table 5. Regression models: Image-based and established measures as predictors of burnout and intent to quit: Full output

Burnout – Big 5 Alone

**Linear Regression**

| **Model Summary - burnout** | | | | | | | | | |
| --- | --- | --- | --- | --- | --- | --- | --- | --- | --- |
| **Model** | | **R** | | **R²** | | **Adjusted R²** | | **RMSE** | |
| H₀ |  | 0.000 |  | 0.000 |  | 0.000 |  | 0.466 |  |
| H₁ |  | 0.238 |  | 0.057 |  | 0.051 |  | 0.454 |  |
|  | | | | | | | | | |

| **ANOVA** | | | | | | | | | | | | | |
| --- | --- | --- | --- | --- | --- | --- | --- | --- | --- | --- | --- | --- | --- |
| **Model** | |  | | **Sum of Squares** | | **df** | | **Mean Square** | | **F** | | **p** | |
| H₁ |  | Regression |  | 9.986 |  | 5 |  | 1.997 |  | 9.674 |  | < .001 |  |
|  |  | Residual |  | 166.402 |  | 806 |  | 0.206 |  |  |  |  |  |
|  |  | Total |  | 176.388 |  | 811 |  |  |  |  |  |  |  |
|  | | | | | | | | | | | | | |
| *Note.*  The intercept model is omitted, as no meaningful information can be shown. | | | | | | | | | | | | | |

| **Coefficients** | | | | | | | | | | | | | |
| --- | --- | --- | --- | --- | --- | --- | --- | --- | --- | --- | --- | --- | --- |
| **Model** | |  | | **Unstandardized** | | **Standard Error** | | **Standardized** | | **t** | | **p** | |
| H₀ |  | (Intercept) |  | 0.319 |  | 0.016 |  |  |  | 19.489 |  | < .001 |  |
| H₁ |  | (Intercept) |  | -0.093 |  | 0.154 |  |  |  | -0.603 |  | 0.547 |  |
|  |  | Openness |  | -0.057 |  | 0.022 |  | -0.094 |  | -2.594 |  | 0.010 |  |
|  |  | Conscientiousness |  | 0.086 |  | 0.026 |  | 0.130 |  | 3.349 |  | < .001 |  |
|  |  | Extraversion |  | -0.013 |  | 0.021 |  | -0.022 |  | -0.617 |  | 0.537 |  |
|  |  | Agreeableness |  | 1.405×10^-5^ |  | 0.024 |  | 2.235×10^-5^ |  | 5.852×10^-4^ |  | 1.000 |  |
|  |  | Neuroticism |  | 0.114 |  | 0.019 |  | 0.219 |  | 6.007 |  | < .001 |  |
|  | | | | | | | | | | | | | |

Burnout –Image-based method Alone

## Linear Regression

| **Model Summary - burnout** | | | | | | | | | |
| --- | --- | --- | --- | --- | --- | --- | --- | --- | --- |
| **Model** | | **R** | | **R²** | | **Adjusted R²** | | **RMSE** | |
| H₀ |  | 0.000 |  | 0.000 |  | 0.000 |  | 0.466 |  |
| H₁ |  | 0.361 |  | 0.131 |  | 0.046 |  | 0.456 |  |
|  | | | | | | | | | |

| **ANOVA** | | | | | | | | | | | | | |
| --- | --- | --- | --- | --- | --- | --- | --- | --- | --- | --- | --- | --- | --- |
| **Model** | |  | | **Sum of Squares** | | **df** | | **Mean Square** | | **F** | | **p** | |
| H₁ |  | Regression |  | 23.024 |  | 72 |  | 0.320 |  | 1.541 |  | 0.004 |  |
|  |  | Residual |  | 153.364 |  | 739 |  | 0.208 |  |  |  |  |  |
|  |  | Total |  | 176.388 |  | 811 |  |  |  |  |  |  |  |
|  | | | | | | | | | | | | | |
| Note.  The intercept model is omitted, as no meaningful information can be shown. | | | | | | | | | | | | | |

| **Coefficients** | | | | | | | | | | | | | |
| --- | --- | --- | --- | --- | --- | --- | --- | --- | --- | --- | --- | --- | --- |
| **Model** | |  | | **Unstandardized** | | **Standard Error** | | **Standardized** | | **t** | | **p** | |
| H₀ |  | (Intercept) |  | 0.319 |  | 0.016 |  |  |  | 19.489 |  | < .001 |  |
| H₁ |  | (Intercept) |  | 0.262 |  | 0.025 |  |  |  | 10.303 |  | < .001 |  |
|  |  | A1Px |  | 1.077×10^-4^ |  | 6.300×10^-5^ |  | 0.080 |  | 1.710 |  | 0.088 |  |
|  |  | A1Py |  | 3.708×10^-5^ |  | 6.292×10^-5^ |  | 0.026 |  | 0.589 |  | 0.556 |  |
|  |  | A1Pz |  | 9.947×10^-5^ |  | 6.148×10^-5^ |  | 0.070 |  | 1.618 |  | 0.106 |  |
|  |  | A2Px |  | -1.472×10^-4^ |  | 7.533×10^-5^ |  | -0.095 |  | -1.954 |  | 0.051 |  |
|  |  | A2Py |  | -3.283×10^-5^ |  | 7.997×10^-5^ |  | -0.019 |  | -0.410 |  | 0.682 |  |
|  |  | A2Pz |  | 1.289×10^-5^ |  | 6.216×10^-5^ |  | 0.009 |  | 0.207 |  | 0.836 |  |
|  |  | A3Px |  | -5.897×10^-5^ |  | 5.756×10^-5^ |  | -0.047 |  | -1.025 |  | 0.306 |  |
|  |  | A3Py |  | 5.096×10^-5^ |  | 5.802×10^-5^ |  | 0.041 |  | 0.878 |  | 0.380 |  |
|  |  | A3Pz |  | -1.806×10^-5^ |  | 6.198×10^-5^ |  | -0.013 |  | -0.291 |  | 0.771 |  |
|  |  | B1Px |  | 1.243×10^-4^ |  | 7.083×10^-5^ |  | 0.080 |  | 1.754 |  | 0.080 |  |
|  |  | B1Py |  | 4.018×10^-5^ |  | 6.574×10^-5^ |  | 0.028 |  | 0.611 |  | 0.541 |  |
|  |  | B1Pz |  | 1.237×10^-5^ |  | 6.285×10^-5^ |  | 0.009 |  | 0.197 |  | 0.844 |  |
|  |  | B2Px |  | 9.389×10^-6^ |  | 6.641×10^-5^ |  | 0.006 |  | 0.141 |  | 0.888 |  |
|  |  | B2Py |  | -5.043×10^-5^ |  | 6.514×10^-5^ |  | -0.034 |  | -0.774 |  | 0.439 |  |
|  |  | B2Pz |  | 1.440×10^-5^ |  | 6.502×10^-5^ |  | 0.010 |  | 0.222 |  | 0.825 |  |
|  |  | B3Px |  | -1.859×10^-5^ |  | 5.936×10^-5^ |  | -0.015 |  | -0.313 |  | 0.754 |  |
|  |  | B3Py |  | -5.117×10^-5^ |  | 5.829×10^-5^ |  | -0.040 |  | -0.878 |  | 0.380 |  |
|  |  | B3Pz |  | -1.104×10^-4^ |  | 6.920×10^-5^ |  | -0.077 |  | -1.595 |  | 0.111 |  |
|  |  | C1Px |  | 1.362×10^-6^ |  | 5.584×10^-5^ |  | 0.001 |  | 0.024 |  | 0.981 |  |
|  |  | C1Py |  | -5.030×10^-5^ |  | 6.493×10^-5^ |  | -0.038 |  | -0.775 |  | 0.439 |  |
|  |  | C1Pz |  | 9.158×10^-5^ |  | 6.538×10^-5^ |  | 0.065 |  | 1.401 |  | 0.162 |  |
|  |  | C2Px |  | 8.975×10^-6^ |  | 6.815×10^-5^ |  | 0.006 |  | 0.132 |  | 0.895 |  |
|  |  | C2Py |  | -5.227×10^-5^ |  | 6.660×10^-5^ |  | -0.037 |  | -0.785 |  | 0.433 |  |
|  |  | C2Pz |  | -3.234×10^-5^ |  | 6.422×10^-5^ |  | -0.024 |  | -0.504 |  | 0.615 |  |
|  |  | C3Px |  | 2.816×10^-5^ |  | 6.010×10^-5^ |  | 0.022 |  | 0.468 |  | 0.640 |  |
|  |  | C3Py |  | 6.321×10^-6^ |  | 6.289×10^-5^ |  | 0.005 |  | 0.101 |  | 0.920 |  |
|  |  | C3Pz |  | -6.089×10^-6^ |  | 6.391×10^-5^ |  | -0.005 |  | -0.095 |  | 0.924 |  |
|  |  | D1Px |  | 5.941×10^-5^ |  | 6.281×10^-5^ |  | 0.046 |  | 0.946 |  | 0.345 |  |
|  |  | D1Py |  | -2.796×10^-5^ |  | 6.703×10^-5^ |  | -0.020 |  | -0.417 |  | 0.677 |  |
|  |  | D1Pz |  | 6.483×10^-5^ |  | 6.673×10^-5^ |  | 0.047 |  | 0.972 |  | 0.332 |  |
|  |  | D2Px |  | -7.078×10^-5^ |  | 6.858×10^-5^ |  | -0.047 |  | -1.032 |  | 0.302 |  |
|  |  | D2Py |  | -2.734×10^-5^ |  | 7.734×10^-5^ |  | -0.018 |  | -0.353 |  | 0.724 |  |
|  |  | D2Pz |  | -3.634×10^-5^ |  | 6.621×10^-5^ |  | -0.025 |  | -0.549 |  | 0.583 |  |
|  |  | D3Px |  | 1.319×10^-5^ |  | 7.072×10^-5^ |  | 0.009 |  | 0.187 |  | 0.852 |  |
|  |  | D3Py |  | -9.492×10^-5^ |  | 5.979×10^-5^ |  | -0.069 |  | -1.587 |  | 0.113 |  |
|  |  | D3Pz |  | -3.958×10^-5^ |  | 6.120×10^-5^ |  | -0.029 |  | -0.647 |  | 0.518 |  |
|  |  | A1Nx |  | 4.250×10^-5^ |  | 6.510×10^-5^ |  | 0.031 |  | 0.653 |  | 0.514 |  |
|  |  | A1Ny |  | -4.261×10^-5^ |  | 6.580×10^-5^ |  | -0.032 |  | -0.648 |  | 0.517 |  |
|  |  | A1Nz |  | 1.482×10^-4^ |  | 6.407×10^-5^ |  | 0.109 |  | 2.314 |  | 0.021 |  |
|  |  | A2Nx |  | -1.614×10^-4^ |  | 6.776×10^-5^ |  | -0.111 |  | -2.382 |  | 0.017 |  |
|  |  | A2Ny |  | -1.189×10^-4^ |  | 6.522×10^-5^ |  | -0.090 |  | -1.822 |  | 0.069 |  |
|  |  | A2Nz |  | 1.119×10^-5^ |  | 7.006×10^-5^ |  | 0.008 |  | 0.160 |  | 0.873 |  |
|  |  | A3Nx |  | -7.416×10^-5^ |  | 6.220×10^-5^ |  | -0.057 |  | -1.192 |  | 0.234 |  |
|  |  | A3Ny |  | -2.798×10^-6^ |  | 6.708×10^-5^ |  | -0.002 |  | -0.042 |  | 0.967 |  |
|  |  | A3Nz |  | 8.918×10^-5^ |  | 6.139×10^-5^ |  | 0.071 |  | 1.453 |  | 0.147 |  |
|  |  | B1Nx |  | 2.320×10^-5^ |  | 6.381×10^-5^ |  | 0.017 |  | 0.364 |  | 0.716 |  |
|  |  | B1Ny |  | 7.984×10^-5^ |  | 6.394×10^-5^ |  | 0.062 |  | 1.249 |  | 0.212 |  |
|  |  | B1Nz |  | 2.489×10^-4^ |  | 6.244×10^-5^ |  | 0.192 |  | 3.986 |  | < .001 |  |
|  |  | B2Nx |  | -3.859×10^-5^ |  | 5.700×10^-5^ |  | -0.030 |  | -0.677 |  | 0.499 |  |
|  |  | B2Ny |  | -4.294×10^-5^ |  | 6.940×10^-5^ |  | -0.031 |  | -0.619 |  | 0.536 |  |
|  |  | B2Nz |  | 1.152×10^-4^ |  | 6.007×10^-5^ |  | 0.091 |  | 1.919 |  | 0.055 |  |
|  |  | B3Nx |  | 2.245×10^-5^ |  | 6.898×10^-5^ |  | 0.016 |  | 0.325 |  | 0.745 |  |
|  |  | B3Ny |  | -4.394×10^-5^ |  | 6.753×10^-5^ |  | -0.030 |  | -0.651 |  | 0.515 |  |
|  |  | B3Nz |  | -2.673×10^-5^ |  | 7.052×10^-5^ |  | -0.019 |  | -0.379 |  | 0.705 |  |
|  |  | C1Nx |  | 7.771×10^-5^ |  | 7.380×10^-5^ |  | 0.055 |  | 1.053 |  | 0.293 |  |
|  |  | C1Ny |  | 1.446×10^-4^ |  | 6.709×10^-5^ |  | 0.097 |  | 2.155 |  | 0.031 |  |
|  |  | C1Nz |  | 3.162×10^-5^ |  | 7.642×10^-5^ |  | 0.019 |  | 0.414 |  | 0.679 |  |
|  |  | C2Nx |  | 5.836×10^-5^ |  | 6.155×10^-5^ |  | 0.046 |  | 0.948 |  | 0.343 |  |
|  |  | C2Ny |  | 3.009×10^-5^ |  | 6.058×10^-5^ |  | 0.024 |  | 0.497 |  | 0.619 |  |
|  |  | C2Nz |  | -1.595×10^-5^ |  | 6.276×10^-5^ |  | -0.012 |  | -0.254 |  | 0.799 |  |
|  |  | C3Nx |  | 6.457×10^-5^ |  | 6.618×10^-5^ |  | 0.050 |  | 0.976 |  | 0.330 |  |
|  |  | C3Ny |  | -4.764×10^-5^ |  | 6.827×10^-5^ |  | -0.035 |  | -0.698 |  | 0.486 |  |
|  |  | C3Nz |  | -7.180×10^-5^ |  | 6.352×10^-5^ |  | -0.056 |  | -1.130 |  | 0.259 |  |
|  |  | D1Nx |  | -7.666×10^-6^ |  | 6.601×10^-5^ |  | -0.006 |  | -0.116 |  | 0.908 |  |
|  |  | D1Ny |  | 8.668×10^-5^ |  | 6.532×10^-5^ |  | 0.064 |  | 1.327 |  | 0.185 |  |
|  |  | D1Nz |  | -5.048×10^-5^ |  | 6.649×10^-5^ |  | -0.038 |  | -0.759 |  | 0.448 |  |
|  |  | D2Nx |  | 2.633×10^-5^ |  | 5.996×10^-5^ |  | 0.020 |  | 0.439 |  | 0.661 |  |
|  |  | D2Ny |  | -1.014×10^-4^ |  | 6.337×10^-5^ |  | -0.076 |  | -1.600 |  | 0.110 |  |
|  |  | D2Nz |  | -1.144×10^-4^ |  | 6.739×10^-5^ |  | -0.085 |  | -1.697 |  | 0.090 |  |
|  |  | D3Nx |  | -5.066×10^-5^ |  | 6.396×10^-5^ |  | -0.038 |  | -0.792 |  | 0.429 |  |
|  |  | D3Ny |  | 5.330×10^-5^ |  | 6.754×10^-5^ |  | 0.037 |  | 0.789 |  | 0.430 |  |
|  |  | D3Nz |  | -1.106×10^-4^ |  | 6.451×10^-5^ |  | -0.083 |  | -1.715 |  | 0.087 |  |
|  | | | | | | | | | | | | | |

Burnout – Image-based method + Big 5

## Linear Regression

| **Model Summary - burnout** | | | | | | | | | |
| --- | --- | --- | --- | --- | --- | --- | --- | --- | --- |
| **Model** | | **R** | | **R²** | | **Adjusted R²** | | **RMSE** | |
| H₀ |  | 0.000 |  | 0.000 |  | 0.000 |  | 0.466 |  |
| H₁ |  | 0.411 |  | 0.169 |  | 0.082 |  | 0.447 |  |
|  | | | | | | | | | |

| **ANOVA** | | | | | | | | | | | | | |
| --- | --- | --- | --- | --- | --- | --- | --- | --- | --- | --- | --- | --- | --- |
| **Model** | |  | | **Sum of Squares** | | **df** | | **Mean Square** | | **F** | | **p** | |
| H₁ |  | Regression |  | 29.791 |  | 77 |  | 0.387 |  | 1.937 |  | < .001 |  |
|  |  | Residual |  | 146.597 |  | 734 |  | 0.200 |  |  |  |  |  |
|  |  | Total |  | 176.388 |  | 811 |  |  |  |  |  |  |  |
|  | | | | | | | | | | | | | |
| Note.  The intercept model is omitted, as no meaningful information can be shown. | | | | | | | | | | | | | |

| **Coefficients** | | | | | | | | | | | | | |
| --- | --- | --- | --- | --- | --- | --- | --- | --- | --- | --- | --- | --- | --- |
| **Model** | |  | | **Unstandardized** | | **Standard Error** | | **Standardized** | | **t** | | **p** | |
| H₀ |  | (Intercept) |  | 0.319 |  | 0.016 |  |  |  | 19.489 |  | < .001 |  |
| H₁ |  | (Intercept) |  | -0.063 |  | 0.158 |  |  |  | -0.399 |  | 0.690 |  |
|  |  | A1Px |  | 1.093×10^-4^ |  | 6.185×10^-5^ |  | 0.082 |  | 1.767 |  | 0.078 |  |
|  |  | A1Py |  | 5.038×10^-5^ |  | 6.182×10^-5^ |  | 0.036 |  | 0.815 |  | 0.415 |  |
|  |  | A1Pz |  | 7.327×10^-5^ |  | 6.103×10^-5^ |  | 0.052 |  | 1.200 |  | 0.230 |  |
|  |  | A2Px |  | -1.383×10^-4^ |  | 7.407×10^-5^ |  | -0.090 |  | -1.867 |  | 0.062 |  |
|  |  | A2Py |  | -1.740×10^-5^ |  | 7.862×10^-5^ |  | -0.010 |  | -0.221 |  | 0.825 |  |
|  |  | A2Pz |  | 2.373×10^-5^ |  | 6.127×10^-5^ |  | 0.017 |  | 0.387 |  | 0.699 |  |
|  |  | A3Px |  | -5.847×10^-5^ |  | 5.654×10^-5^ |  | -0.046 |  | -1.034 |  | 0.301 |  |
|  |  | A3Py |  | 4.901×10^-5^ |  | 5.697×10^-5^ |  | 0.039 |  | 0.860 |  | 0.390 |  |
|  |  | A3Pz |  | -2.887×10^-5^ |  | 6.096×10^-5^ |  | -0.021 |  | -0.474 |  | 0.636 |  |
|  |  | B1Px |  | 1.161×10^-4^ |  | 6.965×10^-5^ |  | 0.074 |  | 1.666 |  | 0.096 |  |
|  |  | B1Py |  | 1.569×10^-5^ |  | 6.466×10^-5^ |  | 0.011 |  | 0.243 |  | 0.808 |  |
|  |  | B1Pz |  | 4.134×10^-5^ |  | 6.198×10^-5^ |  | 0.030 |  | 0.667 |  | 0.505 |  |
|  |  | B2Px |  | 1.898×10^-5^ |  | 6.520×10^-5^ |  | 0.013 |  | 0.291 |  | 0.771 |  |
|  |  | B2Py |  | -4.243×10^-5^ |  | 6.414×10^-5^ |  | -0.029 |  | -0.662 |  | 0.508 |  |
|  |  | B2Pz |  | -1.580×10^-5^ |  | 6.420×10^-5^ |  | -0.011 |  | -0.246 |  | 0.806 |  |
|  |  | B3Px |  | -2.403×10^-5^ |  | 5.852×10^-5^ |  | -0.019 |  | -0.411 |  | 0.681 |  |
|  |  | B3Py |  | -4.651×10^-5^ |  | 5.752×10^-5^ |  | -0.036 |  | -0.809 |  | 0.419 |  |
|  |  | B3Pz |  | -1.034×10^-4^ |  | 6.798×10^-5^ |  | -0.072 |  | -1.520 |  | 0.129 |  |
|  |  | C1Px |  | 3.209×10^-6^ |  | 5.549×10^-5^ |  | 0.003 |  | 0.058 |  | 0.954 |  |
|  |  | C1Py |  | -3.457×10^-5^ |  | 6.417×10^-5^ |  | -0.026 |  | -0.539 |  | 0.590 |  |
|  |  | C1Pz |  | 7.869×10^-5^ |  | 6.433×10^-5^ |  | 0.056 |  | 1.223 |  | 0.222 |  |
|  |  | C2Px |  | 2.006×10^-5^ |  | 6.695×10^-5^ |  | 0.014 |  | 0.300 |  | 0.765 |  |
|  |  | C2Py |  | -6.080×10^-5^ |  | 6.541×10^-5^ |  | -0.042 |  | -0.929 |  | 0.353 |  |
|  |  | C2Pz |  | -3.383×10^-5^ |  | 6.309×10^-5^ |  | -0.025 |  | -0.536 |  | 0.592 |  |
|  |  | C3Px |  | 2.455×10^-5^ |  | 5.933×10^-5^ |  | 0.019 |  | 0.414 |  | 0.679 |  |
|  |  | C3Py |  | 8.465×10^-6^ |  | 6.187×10^-5^ |  | 0.006 |  | 0.137 |  | 0.891 |  |
|  |  | C3Pz |  | -9.099×10^-6^ |  | 6.296×10^-5^ |  | -0.007 |  | -0.145 |  | 0.885 |  |
|  |  | D1Px |  | 7.237×10^-5^ |  | 6.177×10^-5^ |  | 0.056 |  | 1.172 |  | 0.242 |  |
|  |  | D1Py |  | -3.284×10^-5^ |  | 6.595×10^-5^ |  | -0.024 |  | -0.498 |  | 0.619 |  |
|  |  | D1Pz |  | 3.035×10^-5^ |  | 6.599×10^-5^ |  | 0.022 |  | 0.460 |  | 0.646 |  |
|  |  | D2Px |  | -6.228×10^-5^ |  | 6.745×10^-5^ |  | -0.041 |  | -0.923 |  | 0.356 |  |
|  |  | D2Py |  | -3.698×10^-5^ |  | 7.592×10^-5^ |  | -0.025 |  | -0.487 |  | 0.626 |  |
|  |  | D2Pz |  | -1.250×10^-5^ |  | 6.521×10^-5^ |  | -0.009 |  | -0.192 |  | 0.848 |  |
|  |  | D3Px |  | 2.785×10^-5^ |  | 6.957×10^-5^ |  | 0.020 |  | 0.400 |  | 0.689 |  |
|  |  | D3Py |  | -7.535×10^-5^ |  | 5.889×10^-5^ |  | -0.055 |  | -1.280 |  | 0.201 |  |
|  |  | D3Pz |  | -3.139×10^-5^ |  | 6.030×10^-5^ |  | -0.023 |  | -0.521 |  | 0.603 |  |
|  |  | A1Nx |  | 4.483×10^-5^ |  | 6.408×10^-5^ |  | 0.033 |  | 0.699 |  | 0.484 |  |
|  |  | A1Ny |  | -3.040×10^-5^ |  | 6.478×10^-5^ |  | -0.023 |  | -0.469 |  | 0.639 |  |
|  |  | A1Nz |  | 1.261×10^-4^ |  | 6.308×10^-5^ |  | 0.093 |  | 1.999 |  | 0.046 |  |
|  |  | A2Nx |  | -1.464×10^-4^ |  | 6.679×10^-5^ |  | -0.101 |  | -2.192 |  | 0.029 |  |
|  |  | A2Ny |  | -1.157×10^-4^ |  | 6.404×10^-5^ |  | -0.088 |  | -1.806 |  | 0.071 |  |
|  |  | A2Nz |  | 3.100×10^-5^ |  | 6.899×10^-5^ |  | 0.022 |  | 0.449 |  | 0.653 |  |
|  |  | A3Nx |  | -1.002×10^-4^ |  | 6.126×10^-5^ |  | -0.077 |  | -1.636 |  | 0.102 |  |
|  |  | A3Ny |  | -4.988×10^-6^ |  | 6.602×10^-5^ |  | -0.004 |  | -0.076 |  | 0.940 |  |
|  |  | A3Nz |  | 6.855×10^-5^ |  | 6.063×10^-5^ |  | 0.055 |  | 1.131 |  | 0.259 |  |
|  |  | B1Nx |  | 2.686×10^-5^ |  | 6.267×10^-5^ |  | 0.020 |  | 0.429 |  | 0.668 |  |
|  |  | B1Ny |  | 6.956×10^-5^ |  | 6.287×10^-5^ |  | 0.054 |  | 1.106 |  | 0.269 |  |
|  |  | B1Nz |  | 2.406×10^-4^ |  | 6.133×10^-5^ |  | 0.185 |  | 3.923 |  | < .001 |  |
|  |  | B2Nx |  | -4.301×10^-5^ |  | 5.609×10^-5^ |  | -0.034 |  | -0.767 |  | 0.443 |  |
|  |  | B2Ny |  | -4.196×10^-5^ |  | 6.815×10^-5^ |  | -0.030 |  | -0.616 |  | 0.538 |  |
|  |  | B2Nz |  | 7.597×10^-5^ |  | 5.946×10^-5^ |  | 0.060 |  | 1.278 |  | 0.202 |  |
|  |  | B3Nx |  | 2.078×10^-5^ |  | 6.770×10^-5^ |  | 0.015 |  | 0.307 |  | 0.759 |  |
|  |  | B3Ny |  | -5.074×10^-5^ |  | 6.646×10^-5^ |  | -0.034 |  | -0.763 |  | 0.445 |  |
|  |  | B3Nz |  | -1.159×10^-5^ |  | 6.945×10^-5^ |  | -0.008 |  | -0.167 |  | 0.867 |  |
|  |  | C1Nx |  | 5.535×10^-5^ |  | 7.280×10^-5^ |  | 0.039 |  | 0.760 |  | 0.447 |  |
|  |  | C1Ny |  | 1.412×10^-4^ |  | 6.622×10^-5^ |  | 0.095 |  | 2.132 |  | 0.033 |  |
|  |  | C1Nz |  | 4.369×10^-5^ |  | 7.520×10^-5^ |  | 0.027 |  | 0.581 |  | 0.561 |  |
|  |  | C2Nx |  | 4.863×10^-5^ |  | 6.061×10^-5^ |  | 0.038 |  | 0.802 |  | 0.423 |  |
|  |  | C2Ny |  | 3.143×10^-5^ |  | 5.955×10^-5^ |  | 0.025 |  | 0.528 |  | 0.598 |  |
|  |  | C2Nz |  | -8.232×10^-6^ |  | 6.162×10^-5^ |  | -0.006 |  | -0.134 |  | 0.894 |  |
|  |  | C3Nx |  | 4.250×10^-5^ |  | 6.523×10^-5^ |  | 0.033 |  | 0.652 |  | 0.515 |  |
|  |  | C3Ny |  | -6.675×10^-5^ |  | 6.716×10^-5^ |  | -0.050 |  | -0.994 |  | 0.321 |  |
|  |  | C3Nz |  | -5.962×10^-5^ |  | 6.248×10^-5^ |  | -0.047 |  | -0.954 |  | 0.340 |  |
|  |  | D1Nx |  | 4.226×10^-5^ |  | 6.557×10^-5^ |  | 0.032 |  | 0.645 |  | 0.519 |  |
|  |  | D1Ny |  | 9.708×10^-5^ |  | 6.427×10^-5^ |  | 0.072 |  | 1.510 |  | 0.131 |  |
|  |  | D1Nz |  | -4.716×10^-5^ |  | 6.534×10^-5^ |  | -0.036 |  | -0.722 |  | 0.471 |  |
|  |  | D2Nx |  | 2.137×10^-5^ |  | 5.893×10^-5^ |  | 0.016 |  | 0.363 |  | 0.717 |  |
|  |  | D2Ny |  | -8.945×10^-5^ |  | 6.223×10^-5^ |  | -0.067 |  | -1.437 |  | 0.151 |  |
|  |  | D2Nz |  | -1.258×10^-4^ |  | 6.626×10^-5^ |  | -0.093 |  | -1.899 |  | 0.058 |  |
|  |  | D3Nx |  | -6.093×10^-5^ |  | 6.286×10^-5^ |  | -0.046 |  | -0.969 |  | 0.333 |  |
|  |  | D3Ny |  | 6.460×10^-5^ |  | 6.648×10^-5^ |  | 0.044 |  | 0.972 |  | 0.332 |  |
|  |  | D3Nz |  | -8.742×10^-5^ |  | 6.348×10^-5^ |  | -0.066 |  | -1.377 |  | 0.169 |  |
|  |  | Openness |  | -0.050 |  | 0.023 |  | -0.082 |  | -2.193 |  | 0.029 |  |
|  |  | Conscientiousness |  | 0.077 |  | 0.026 |  | 0.117 |  | 2.934 |  | 0.003 |  |
|  |  | Extraversion |  | -0.011 |  | 0.022 |  | -0.020 |  | -0.523 |  | 0.601 |  |
|  |  | Agreeableness |  | -0.009 |  | 0.025 |  | -0.014 |  | -0.362 |  | 0.717 |  |
|  |  | Neuroticism |  | 0.100 |  | 0.020 |  | 0.191 |  | 5.033 |  | < .001 |  |
|  | | | | | | | | | | | | | |

Quitting - Big 5 Alone

## Linear Regression

| **Model Summary - quitting** | | | | | | | | | |
| --- | --- | --- | --- | --- | --- | --- | --- | --- | --- |
| **Model** | | **R** | | **R²** | | **Adjusted R²** | | **RMSE** | |
| H₀ |  | 0.000 |  | 0.000 |  | 0.000 |  | 0.420 |  |
| H₁ |  | 0.166 |  | 0.028 |  | 0.021 |  | 0.415 |  |
|  | | | | | | | | | |

| **ANOVA** | | | | | | | | | | | | | |
| --- | --- | --- | --- | --- | --- | --- | --- | --- | --- | --- | --- | --- | --- |
| **Model** | |  | | **Sum of Squares** | | **df** | | **Mean Square** | | **F** | | **p** | |
| H₁ |  | Regression |  | 3.929 |  | 5 |  | 0.786 |  | 4.559 |  | < .001 |  |
|  |  | Residual |  | 138.922 |  | 806 |  | 0.172 |  |  |  |  |  |
|  |  | Total |  | 142.851 |  | 811 |  |  |  |  |  |  |  |
|  | | | | | | | | | | | | | |
| Note.  The intercept model is omitted, as no meaningful information can be shown. | | | | | | | | | | | | | |

| **Coefficients** | | | | | | | | | | | | | |
| --- | --- | --- | --- | --- | --- | --- | --- | --- | --- | --- | --- | --- | --- |
| **Model** | |  | | **Unstandardized** | | **Standard Error** | | **Standardized** | | **t** | | **p** | |
| H₀ |  | (Intercept) |  | 0.228 |  | 0.015 |  |  |  | 15.469 |  | < .001 |  |
| H₁ |  | (Intercept) |  | -0.064 |  | 0.141 |  |  |  | -0.451 |  | 0.652 |  |
|  |  | Openness |  | 0.072 |  | 0.020 |  | 0.132 |  | 3.600 |  | < .001 |  |
|  |  | Conscientiousness |  | -0.011 |  | 0.023 |  | -0.019 |  | -0.486 |  | 0.627 |  |
|  |  | Extraversion |  | -0.013 |  | 0.019 |  | -0.025 |  | -0.693 |  | 0.489 |  |
|  |  | Agreeableness |  | -0.009 |  | 0.022 |  | -0.015 |  | -0.394 |  | 0.694 |  |
|  |  | Neuroticism |  | 0.045 |  | 0.017 |  | 0.096 |  | 2.586 |  | 0.010 |  |
|  | | | | | | | | | | | | | |

Quitting - Image-based method Alone

## Linear Regression

| **Model Summary - quitting** | | | | | | | | | |
| --- | --- | --- | --- | --- | --- | --- | --- | --- | --- |
| **Model** | | **R** | | **R²** | | **Adjusted R²** | | **RMSE** | |
| H₀ |  | 0.000 |  | 0.000 |  | 0.000 |  | 0.420 |  |
| H₁ |  | 0.354 |  | 0.125 |  | 0.040 |  | 0.411 |  |
|  | | | | | | | | | |

| **ANOVA** | | | | | | | | | | | | | |
| --- | --- | --- | --- | --- | --- | --- | --- | --- | --- | --- | --- | --- | --- |
| **Model** | |  | | **Sum of Squares** | | **df** | | **Mean Square** | | **F** | | **p** | |
| H₁ |  | Regression |  | 17.900 |  | 72 |  | 0.249 |  | 1.470 |  | 0.009 |  |
|  |  | Residual |  | 124.951 |  | 739 |  | 0.169 |  |  |  |  |  |
|  |  | Total |  | 142.851 |  | 811 |  |  |  |  |  |  |  |
|  | | | | | | | | | | | | | |
| Note.  The intercept model is omitted, as no meaningful information can be shown. | | | | | | | | | | | | | |

| **Coefficients** | | | | | | | | | | | | | |
| --- | --- | --- | --- | --- | --- | --- | --- | --- | --- | --- | --- | --- | --- |
| **Model** | |  | | **Unstandardized** | | **Standard Error** | | **Standardized** | | **t** | | **p** | |
| H₀ |  | (Intercept) |  | 0.228 |  | 0.015 |  |  |  | 15.469 |  | < .001 |  |
| H₁ |  | (Intercept) |  | 0.202 |  | 0.023 |  |  |  | 8.800 |  | < .001 |  |
|  |  | A1Px |  | 1.171×10^-4^ |  | 5.686×10^-5^ |  | 0.097 |  | 2.058 |  | 0.040 |  |
|  |  | A1Py |  | 2.953×10^-5^ |  | 5.679×10^-5^ |  | 0.023 |  | 0.520 |  | 0.603 |  |
|  |  | A1Pz |  | -7.897×10^-5^ |  | 5.549×10^-5^ |  | -0.062 |  | -1.423 |  | 0.155 |  |
|  |  | A2Px |  | 7.312×10^-6^ |  | 6.800×10^-5^ |  | 0.005 |  | 0.108 |  | 0.914 |  |
|  |  | A2Py |  | -1.205×10^-4^ |  | 7.218×10^-5^ |  | -0.079 |  | -1.669 |  | 0.095 |  |
|  |  | A2Pz |  | -1.100×10^-5^ |  | 5.611×10^-5^ |  | -0.009 |  | -0.196 |  | 0.845 |  |
|  |  | A3Px |  | 2.108×10^-5^ |  | 5.195×10^-5^ |  | 0.019 |  | 0.406 |  | 0.685 |  |
|  |  | A3Py |  | 2.479×10^-5^ |  | 5.237×10^-5^ |  | 0.022 |  | 0.473 |  | 0.636 |  |
|  |  | A3Pz |  | 1.735×10^-5^ |  | 5.594×10^-5^ |  | 0.014 |  | 0.310 |  | 0.757 |  |
|  |  | B1Px |  | 7.142×10^-6^ |  | 6.394×10^-5^ |  | 0.005 |  | 0.112 |  | 0.911 |  |
|  |  | B1Py |  | 2.331×10^-5^ |  | 5.934×10^-5^ |  | 0.018 |  | 0.393 |  | 0.695 |  |
|  |  | B1Pz |  | -2.012×10^-5^ |  | 5.673×10^-5^ |  | -0.016 |  | -0.355 |  | 0.723 |  |
|  |  | B2Px |  | -6.431×10^-5^ |  | 5.994×10^-5^ |  | -0.049 |  | -1.073 |  | 0.284 |  |
|  |  | B2Py |  | -5.107×10^-5^ |  | 5.880×10^-5^ |  | -0.039 |  | -0.868 |  | 0.385 |  |
|  |  | B2Pz |  | -1.241×10^-4^ |  | 5.869×10^-5^ |  | -0.097 |  | -2.115 |  | 0.035 |  |
|  |  | B3Px |  | 2.289×10^-5^ |  | 5.358×10^-5^ |  | 0.020 |  | 0.427 |  | 0.669 |  |
|  |  | B3Py |  | 6.192×10^-5^ |  | 5.261×10^-5^ |  | 0.053 |  | 1.177 |  | 0.240 |  |
|  |  | B3Pz |  | -6.299×10^-5^ |  | 6.246×10^-5^ |  | -0.049 |  | -1.008 |  | 0.314 |  |
|  |  | C1Px |  | -7.259×10^-5^ |  | 5.040×10^-5^ |  | -0.065 |  | -1.440 |  | 0.150 |  |
|  |  | C1Py |  | -6.127×10^-5^ |  | 5.860×10^-5^ |  | -0.051 |  | -1.045 |  | 0.296 |  |
|  |  | C1Pz |  | -3.340×10^-5^ |  | 5.902×10^-5^ |  | -0.026 |  | -0.566 |  | 0.572 |  |
|  |  | C2Px |  | 1.092×10^-4^ |  | 6.152×10^-5^ |  | 0.087 |  | 1.774 |  | 0.076 |  |
|  |  | C2Py |  | 6.472×10^-5^ |  | 6.011×10^-5^ |  | 0.050 |  | 1.077 |  | 0.282 |  |
|  |  | C2Pz |  | -3.207×10^-5^ |  | 5.797×10^-5^ |  | -0.026 |  | -0.553 |  | 0.580 |  |
|  |  | C3Px |  | 7.167×10^-5^ |  | 5.424×10^-5^ |  | 0.062 |  | 1.321 |  | 0.187 |  |
|  |  | C3Py |  | 3.555×10^-7^ |  | 5.677×10^-5^ |  | 2.934×10^-4^ |  | 0.006 |  | 0.995 |  |
|  |  | C3Pz |  | 2.218×10^-5^ |  | 5.768×10^-5^ |  | 0.019 |  | 0.384 |  | 0.701 |  |
|  |  | D1Px |  | -1.800×10^-5^ |  | 5.670×10^-5^ |  | -0.015 |  | -0.317 |  | 0.751 |  |
|  |  | D1Py |  | -3.363×10^-5^ |  | 6.050×10^-5^ |  | -0.027 |  | -0.556 |  | 0.578 |  |
|  |  | D1Pz |  | -5.137×10^-5^ |  | 6.023×10^-5^ |  | -0.041 |  | -0.853 |  | 0.394 |  |
|  |  | D2Px |  | 6.547×10^-5^ |  | 6.190×10^-5^ |  | 0.048 |  | 1.058 |  | 0.291 |  |
|  |  | D2Py |  | -1.998×10^-5^ |  | 6.981×10^-5^ |  | -0.015 |  | -0.286 |  | 0.775 |  |
|  |  | D2Pz |  | 8.668×10^-5^ |  | 5.977×10^-5^ |  | 0.066 |  | 1.450 |  | 0.147 |  |
|  |  | D3Px |  | -3.361×10^-5^ |  | 6.383×10^-5^ |  | -0.027 |  | -0.526 |  | 0.599 |  |
|  |  | D3Py |  | -6.280×10^-6^ |  | 5.397×10^-5^ |  | -0.005 |  | -0.116 |  | 0.907 |  |
|  |  | D3Pz |  | -7.381×10^-5^ |  | 5.524×10^-5^ |  | -0.060 |  | -1.336 |  | 0.182 |  |
|  |  | A1Nx |  | 4.616×10^-5^ |  | 5.876×10^-5^ |  | 0.038 |  | 0.786 |  | 0.432 |  |
|  |  | A1Ny |  | 5.302×10^-5^ |  | 5.940×10^-5^ |  | 0.044 |  | 0.893 |  | 0.372 |  |
|  |  | A1Nz |  | -5.018×10^-5^ |  | 5.783×10^-5^ |  | -0.041 |  | -0.868 |  | 0.386 |  |
|  |  | A2Nx |  | 1.562×10^-4^ |  | 6.117×10^-5^ |  | 0.120 |  | 2.554 |  | 0.011 |  |
|  |  | A2Ny |  | -3.248×10^-6^ |  | 5.887×10^-5^ |  | -0.003 |  | -0.055 |  | 0.956 |  |
|  |  | A2Nz |  | -6.304×10^-6^ |  | 6.323×10^-5^ |  | -0.005 |  | -0.100 |  | 0.921 |  |
|  |  | A3Nx |  | 5.312×10^-5^ |  | 5.614×10^-5^ |  | 0.046 |  | 0.946 |  | 0.344 |  |
|  |  | A3Ny |  | -3.968×10^-5^ |  | 6.055×10^-5^ |  | -0.033 |  | -0.655 |  | 0.512 |  |
|  |  | A3Nz |  | 2.022×10^-5^ |  | 5.541×10^-5^ |  | 0.018 |  | 0.365 |  | 0.715 |  |
|  |  | B1Nx |  | 4.701×10^-5^ |  | 5.760×10^-5^ |  | 0.039 |  | 0.816 |  | 0.415 |  |
|  |  | B1Ny |  | -1.157×10^-6^ |  | 5.771×10^-5^ |  | -9.959×10^-4^ |  | -0.020 |  | 0.984 |  |
|  |  | B1Nz |  | 1.888×10^-5^ |  | 5.636×10^-5^ |  | 0.016 |  | 0.335 |  | 0.738 |  |
|  |  | B2Nx |  | -5.979×10^-5^ |  | 5.145×10^-5^ |  | -0.052 |  | -1.162 |  | 0.246 |  |
|  |  | B2Ny |  | -6.678×10^-5^ |  | 6.264×10^-5^ |  | -0.053 |  | -1.066 |  | 0.287 |  |
|  |  | B2Nz |  | -6.291×10^-5^ |  | 5.422×10^-5^ |  | -0.055 |  | -1.160 |  | 0.246 |  |
|  |  | B3Nx |  | -4.098×10^-5^ |  | 6.227×10^-5^ |  | -0.033 |  | -0.658 |  | 0.511 |  |
|  |  | B3Ny |  | -8.722×10^-5^ |  | 6.095×10^-5^ |  | -0.066 |  | -1.431 |  | 0.153 |  |
|  |  | B3Nz |  | -6.562×10^-6^ |  | 6.366×10^-5^ |  | -0.005 |  | -0.103 |  | 0.918 |  |
|  |  | C1Nx |  | -1.752×10^-4^ |  | 6.661×10^-5^ |  | -0.137 |  | -2.631 |  | 0.009 |  |
|  |  | C1Ny |  | 4.023×10^-5^ |  | 6.056×10^-5^ |  | 0.030 |  | 0.664 |  | 0.507 |  |
|  |  | C1Nz |  | 5.314×10^-5^ |  | 6.898×10^-5^ |  | 0.036 |  | 0.770 |  | 0.441 |  |
|  |  | C2Nx |  | 6.710×10^-5^ |  | 5.556×10^-5^ |  | 0.058 |  | 1.208 |  | 0.228 |  |
|  |  | C2Ny |  | 8.565×10^-5^ |  | 5.468×10^-5^ |  | 0.075 |  | 1.566 |  | 0.118 |  |
|  |  | C2Nz |  | 3.451×10^-5^ |  | 5.664×10^-5^ |  | 0.029 |  | 0.609 |  | 0.543 |  |
|  |  | C3Nx |  | 5.956×10^-5^ |  | 5.974×10^-5^ |  | 0.051 |  | 0.997 |  | 0.319 |  |
|  |  | C3Ny |  | -2.849×10^-6^ |  | 6.162×10^-5^ |  | -0.002 |  | -0.046 |  | 0.963 |  |
|  |  | C3Nz |  | -5.719×10^-5^ |  | 5.734×10^-5^ |  | -0.050 |  | -0.997 |  | 0.319 |  |
|  |  | D1Nx |  | -2.907×10^-5^ |  | 5.958×10^-5^ |  | -0.024 |  | -0.488 |  | 0.626 |  |
|  |  | D1Ny |  | -1.627×10^-5^ |  | 5.896×10^-5^ |  | -0.013 |  | -0.276 |  | 0.783 |  |
|  |  | D1Nz |  | 1.447×10^-4^ |  | 6.001×10^-5^ |  | 0.122 |  | 2.410 |  | 0.016 |  |
|  |  | D2Nx |  | -3.423×10^-6^ |  | 5.412×10^-5^ |  | -0.003 |  | -0.063 |  | 0.950 |  |
|  |  | D2Ny |  | 1.779×10^-4^ |  | 5.720×10^-5^ |  | 0.148 |  | 3.110 |  | 0.002 |  |
|  |  | D2Nz |  | 2.656×10^-5^ |  | 6.082×10^-5^ |  | 0.022 |  | 0.437 |  | 0.662 |  |
|  |  | D3Nx |  | -3.809×10^-5^ |  | 5.773×10^-5^ |  | -0.032 |  | -0.660 |  | 0.510 |  |
|  |  | D3Ny |  | -1.818×10^-4^ |  | 6.096×10^-5^ |  | -0.139 |  | -2.982 |  | 0.003 |  |
|  |  | D3Nz |  | 1.011×10^-4^ |  | 5.823×10^-5^ |  | 0.085 |  | 1.736 |  | 0.083 |  |
|  | | | | | | | | | | | | | |

Quitting - Image-based method + Big 5

## Linear Regression

| **Model Summary - quitting** | | | | | | | | | |
| --- | --- | --- | --- | --- | --- | --- | --- | --- | --- |
| **Model** | | **R** | | **R²** | | **Adjusted R²** | | **RMSE** | |
| H₀ |  | 0.000 |  | 0.000 |  | 0.000 |  | 0.420 |  |
| H₁ |  | 0.380 |  | 0.145 |  | 0.055 |  | 0.408 |  |
|  | | | | | | | | | |

| **ANOVA** | | | | | | | | | | | | | |
| --- | --- | --- | --- | --- | --- | --- | --- | --- | --- | --- | --- | --- | --- |
| **Model** | |  | | **Sum of Squares** | | **df** | | **Mean Square** | | **F** | | **p** | |
| H₁ |  | Regression |  | 20.657 |  | 77 |  | 0.268 |  | 1.611 |  | 0.001 |  |
|  |  | Residual |  | 122.194 |  | 734 |  | 0.166 |  |  |  |  |  |
|  |  | Total |  | 142.851 |  | 811 |  |  |  |  |  |  |  |
|  | | | | | | | | | | | | | |
| Note.  The intercept model is omitted, as no meaningful information can be shown. | | | | | | | | | | | | | |

| **Coefficients** | | | | | | | | | | | | | |
| --- | --- | --- | --- | --- | --- | --- | --- | --- | --- | --- | --- | --- | --- |
| **Model** | |  | | **Unstandardized** | | **Standard Error** | | **Standardized** | | **t** | | **p** | |
| H₀ |  | (Intercept) |  | 0.228 |  | 0.015 |  |  |  | 15.469 |  | < .001 |  |
| H₁ |  | (Intercept) |  | -0.067 |  | 0.145 |  |  |  | -0.464 |  | 0.643 |  |
|  |  | A1Px |  | 1.150×10^-4^ |  | 5.647×10^-5^ |  | 0.095 |  | 2.037 |  | 0.042 |  |
|  |  | A1Py |  | 2.975×10^-5^ |  | 5.644×10^-5^ |  | 0.023 |  | 0.527 |  | 0.598 |  |
|  |  | A1Pz |  | -6.694×10^-5^ |  | 5.572×10^-5^ |  | -0.053 |  | -1.201 |  | 0.230 |  |
|  |  | A2Px |  | -4.837×10^-6^ |  | 6.762×10^-5^ |  | -0.003 |  | -0.072 |  | 0.943 |  |
|  |  | A2Py |  | -1.310×10^-4^ |  | 7.178×10^-5^ |  | -0.086 |  | -1.825 |  | 0.068 |  |
|  |  | A2Pz |  | -1.005×10^-5^ |  | 5.594×10^-5^ |  | -0.008 |  | -0.180 |  | 0.857 |  |
|  |  | A3Px |  | 3.035×10^-5^ |  | 5.162×10^-5^ |  | 0.027 |  | 0.588 |  | 0.557 |  |
|  |  | A3Py |  | 2.800×10^-5^ |  | 5.202×10^-5^ |  | 0.025 |  | 0.538 |  | 0.591 |  |
|  |  | A3Pz |  | 2.254×10^-5^ |  | 5.566×10^-5^ |  | 0.019 |  | 0.405 |  | 0.686 |  |
|  |  | B1Px |  | -9.538×10^-6^ |  | 6.359×10^-5^ |  | -0.007 |  | -0.150 |  | 0.881 |  |
|  |  | B1Py |  | 1.958×10^-5^ |  | 5.903×10^-5^ |  | 0.015 |  | 0.332 |  | 0.740 |  |
|  |  | B1Pz |  | -1.283×10^-5^ |  | 5.659×10^-5^ |  | -0.010 |  | -0.227 |  | 0.821 |  |
|  |  | B2Px |  | -6.021×10^-5^ |  | 5.953×10^-5^ |  | -0.046 |  | -1.011 |  | 0.312 |  |
|  |  | B2Py |  | -5.552×10^-5^ |  | 5.856×10^-5^ |  | -0.042 |  | -0.948 |  | 0.343 |  |
|  |  | B2Pz |  | -1.221×10^-4^ |  | 5.861×10^-5^ |  | -0.095 |  | -2.083 |  | 0.038 |  |
|  |  | B3Px |  | 4.140×10^-5^ |  | 5.343×10^-5^ |  | 0.036 |  | 0.775 |  | 0.439 |  |
|  |  | B3Py |  | 6.423×10^-5^ |  | 5.251×10^-5^ |  | 0.055 |  | 1.223 |  | 0.222 |  |
|  |  | B3Pz |  | -5.882×10^-5^ |  | 6.207×10^-5^ |  | -0.046 |  | -0.948 |  | 0.344 |  |
|  |  | C1Px |  | -7.373×10^-5^ |  | 5.066×10^-5^ |  | -0.066 |  | -1.455 |  | 0.146 |  |
|  |  | C1Py |  | -6.614×10^-5^ |  | 5.859×10^-5^ |  | -0.055 |  | -1.129 |  | 0.259 |  |
|  |  | C1Pz |  | -2.636×10^-5^ |  | 5.873×10^-5^ |  | -0.021 |  | -0.449 |  | 0.654 |  |
|  |  | C2Px |  | 1.013×10^-4^ |  | 6.112×10^-5^ |  | 0.080 |  | 1.658 |  | 0.098 |  |
|  |  | C2Py |  | 5.994×10^-5^ |  | 5.972×10^-5^ |  | 0.047 |  | 1.004 |  | 0.316 |  |
|  |  | C2Pz |  | -3.240×10^-5^ |  | 5.760×10^-5^ |  | -0.026 |  | -0.562 |  | 0.574 |  |
|  |  | C3Px |  | 6.263×10^-5^ |  | 5.417×10^-5^ |  | 0.054 |  | 1.156 |  | 0.248 |  |
|  |  | C3Py |  | 1.994×10^-6^ |  | 5.649×10^-5^ |  | 0.002 |  | 0.035 |  | 0.972 |  |
|  |  | C3Pz |  | 2.811×10^-5^ |  | 5.748×10^-5^ |  | 0.024 |  | 0.489 |  | 0.625 |  |
|  |  | D1Px |  | -2.211×10^-5^ |  | 5.640×10^-5^ |  | -0.019 |  | -0.392 |  | 0.695 |  |
|  |  | D1Py |  | -3.697×10^-5^ |  | 6.021×10^-5^ |  | -0.030 |  | -0.614 |  | 0.539 |  |
|  |  | D1Pz |  | -4.398×10^-5^ |  | 6.025×10^-5^ |  | -0.035 |  | -0.730 |  | 0.466 |  |
|  |  | D2Px |  | 5.483×10^-5^ |  | 6.158×10^-5^ |  | 0.040 |  | 0.890 |  | 0.374 |  |
|  |  | D2Py |  | -1.917×10^-5^ |  | 6.931×10^-5^ |  | -0.014 |  | -0.277 |  | 0.782 |  |
|  |  | D2Pz |  | 8.378×10^-5^ |  | 5.953×10^-5^ |  | 0.064 |  | 1.407 |  | 0.160 |  |
|  |  | D3Px |  | -3.262×10^-5^ |  | 6.352×10^-5^ |  | -0.026 |  | -0.514 |  | 0.608 |  |
|  |  | D3Py |  | 4.009×10^-6^ |  | 5.377×10^-5^ |  | 0.003 |  | 0.075 |  | 0.941 |  |
|  |  | D3Pz |  | -6.886×10^-5^ |  | 5.505×10^-5^ |  | -0.056 |  | -1.251 |  | 0.211 |  |
|  |  | A1Nx |  | 4.689×10^-5^ |  | 5.851×10^-5^ |  | 0.038 |  | 0.801 |  | 0.423 |  |
|  |  | A1Ny |  | 4.063×10^-5^ |  | 5.914×10^-5^ |  | 0.034 |  | 0.687 |  | 0.492 |  |
|  |  | A1Nz |  | -4.218×10^-5^ |  | 5.759×10^-5^ |  | -0.034 |  | -0.732 |  | 0.464 |  |
|  |  | A2Nx |  | 1.552×10^-4^ |  | 6.097×10^-5^ |  | 0.119 |  | 2.545 |  | 0.011 |  |
|  |  | A2Ny |  | -5.375×10^-6^ |  | 5.846×10^-5^ |  | -0.005 |  | -0.092 |  | 0.927 |  |
|  |  | A2Nz |  | -1.609×10^-5^ |  | 6.299×10^-5^ |  | -0.013 |  | -0.255 |  | 0.798 |  |
|  |  | A3Nx |  | 4.426×10^-5^ |  | 5.593×10^-5^ |  | 0.038 |  | 0.791 |  | 0.429 |  |
|  |  | A3Ny |  | -2.995×10^-5^ |  | 6.028×10^-5^ |  | -0.025 |  | -0.497 |  | 0.619 |  |
|  |  | A3Nz |  | 1.910×10^-5^ |  | 5.535×10^-5^ |  | 0.017 |  | 0.345 |  | 0.730 |  |
|  |  | B1Nx |  | 3.959×10^-5^ |  | 5.721×10^-5^ |  | 0.032 |  | 0.692 |  | 0.489 |  |
|  |  | B1Ny |  | -5.091×10^-6^ |  | 5.740×10^-5^ |  | -0.004 |  | -0.089 |  | 0.929 |  |
|  |  | B1Nz |  | 1.504×10^-5^ |  | 5.600×10^-5^ |  | 0.013 |  | 0.269 |  | 0.788 |  |
|  |  | B2Nx |  | -6.310×10^-5^ |  | 5.121×10^-5^ |  | -0.055 |  | -1.232 |  | 0.218 |  |
|  |  | B2Ny |  | -7.340×10^-5^ |  | 6.222×10^-5^ |  | -0.058 |  | -1.180 |  | 0.238 |  |
|  |  | B2Nz |  | -7.067×10^-5^ |  | 5.429×10^-5^ |  | -0.062 |  | -1.302 |  | 0.193 |  |
|  |  | B3Nx |  | -4.223×10^-5^ |  | 6.181×10^-5^ |  | -0.034 |  | -0.683 |  | 0.495 |  |
|  |  | B3Ny |  | -9.190×10^-5^ |  | 6.067×10^-5^ |  | -0.069 |  | -1.515 |  | 0.130 |  |
|  |  | B3Nz |  | 2.023×10^-6^ |  | 6.341×10^-5^ |  | 0.002 |  | 0.032 |  | 0.975 |  |
|  |  | C1Nx |  | -1.709×10^-4^ |  | 6.647×10^-5^ |  | -0.133 |  | -2.572 |  | 0.010 |  |
|  |  | C1Ny |  | 4.680×10^-5^ |  | 6.046×10^-5^ |  | 0.035 |  | 0.774 |  | 0.439 |  |
|  |  | C1Nz |  | 5.881×10^-5^ |  | 6.866×10^-5^ |  | 0.040 |  | 0.857 |  | 0.392 |  |
|  |  | C2Nx |  | 5.526×10^-5^ |  | 5.533×10^-5^ |  | 0.048 |  | 0.999 |  | 0.318 |  |
|  |  | C2Ny |  | 7.919×10^-5^ |  | 5.437×10^-5^ |  | 0.069 |  | 1.457 |  | 0.146 |  |
|  |  | C2Nz |  | 3.745×10^-5^ |  | 5.625×10^-5^ |  | 0.031 |  | 0.666 |  | 0.506 |  |
|  |  | C3Nx |  | 4.977×10^-5^ |  | 5.955×10^-5^ |  | 0.042 |  | 0.836 |  | 0.404 |  |
|  |  | C3Ny |  | -9.962×10^-6^ |  | 6.132×10^-5^ |  | -0.008 |  | -0.162 |  | 0.871 |  |
|  |  | C3Nz |  | -5.814×10^-5^ |  | 5.705×10^-5^ |  | -0.051 |  | -1.019 |  | 0.308 |  |
|  |  | D1Nx |  | -1.064×10^-5^ |  | 5.987×10^-5^ |  | -0.009 |  | -0.178 |  | 0.859 |  |
|  |  | D1Ny |  | -6.604×10^-6^ |  | 5.868×10^-5^ |  | -0.005 |  | -0.113 |  | 0.910 |  |
|  |  | D1Nz |  | 1.450×10^-4^ |  | 5.966×10^-5^ |  | 0.122 |  | 2.430 |  | 0.015 |  |
|  |  | D2Nx |  | -8.010×10^-6^ |  | 5.380×10^-5^ |  | -0.007 |  | -0.149 |  | 0.882 |  |
|  |  | D2Ny |  | 1.767×10^-4^ |  | 5.681×10^-5^ |  | 0.147 |  | 3.110 |  | 0.002 |  |
|  |  | D2Nz |  | 2.347×10^-5^ |  | 6.049×10^-5^ |  | 0.019 |  | 0.388 |  | 0.698 |  |
|  |  | D3Nx |  | -3.911×10^-5^ |  | 5.739×10^-5^ |  | -0.033 |  | -0.681 |  | 0.496 |  |
|  |  | D3Ny |  | -1.785×10^-4^ |  | 6.070×10^-5^ |  | -0.136 |  | -2.941 |  | 0.003 |  |
|  |  | D3Nz |  | 1.078×10^-4^ |  | 5.796×10^-5^ |  | 0.090 |  | 1.861 |  | 0.063 |  |
|  |  | Openness |  | 0.058 |  | 0.021 |  | 0.106 |  | 2.787 |  | 0.005 |  |
|  |  | Conscientiousness |  | -0.005 |  | 0.024 |  | -0.009 |  | -0.225 |  | 0.822 |  |
|  |  | Extraversion |  | -0.004 |  | 0.020 |  | -0.008 |  | -0.217 |  | 0.828 |  |
|  |  | Agreeableness |  | -0.012 |  | 0.023 |  | -0.022 |  | -0.542 |  | 0.588 |  |
|  |  | Neuroticism |  | 0.044 |  | 0.018 |  | 0.094 |  | 2.438 |  | 0.015 |  |
|  | | | | | | | | | | | | | |

Burnout – PSS Only

## Linear Regression

| **Model Summary - burnout** | | | | | | | | | |
| --- | --- | --- | --- | --- | --- | --- | --- | --- | --- |
| **Model** | | **R** | | **R²** | | **Adjusted R²** | | **RMSE** | |
| H₀ |  | 0.000 |  | 0.000 |  | 0.000 |  | 0.466 |  |
| H₁ |  | 0.099 |  | 0.010 |  | 0.009 |  | 0.464 |  |
|  | | | | | | | | | |

| **ANOVA** | | | | | | | | | | | | | |
| --- | --- | --- | --- | --- | --- | --- | --- | --- | --- | --- | --- | --- | --- |
| **Model** | |  | | **Sum of Squares** | | **df** | | **Mean Square** | | **F** | | **p** | |
| H₁ |  | Regression |  | 1.738 |  | 1 |  | 1.738 |  | 8.058 |  | 0.005 |  |
|  |  | Residual |  | 174.650 |  | 810 |  | 0.216 |  |  |  |  |  |
|  |  | Total |  | 176.388 |  | 811 |  |  |  |  |  |  |  |
|  | | | | | | | | | | | | | |
| Note.  The intercept model is omitted, as no meaningful information can be shown. | | | | | | | | | | | | | |

| **Coefficients** | | | | | | | | | | | | | |
| --- | --- | --- | --- | --- | --- | --- | --- | --- | --- | --- | --- | --- | --- |
| **Model** | |  | | **Unstandardized** | | **Standard Error** | | **Standardized** | | **t** | | **p** | |
| H₀ |  | (Intercept) |  | 0.319 |  | 0.016 |  |  |  | 19.489 |  | < .001 |  |
| H₁ |  | (Intercept) |  | 0.088 |  | 0.083 |  |  |  | 1.053 |  | 0.293 |  |
|  |  | PSS |  | 0.008 |  | 0.003 |  | 0.099 |  | 2.839 |  | 0.005 |  |
|  | | | | | | | | | | | | | |

Burnout – IMAGE-BASED METHOD only (above)

Burnout PSS + Image-based method

## Linear Regression

| **Model Summary - burnout** | | | | | | | | | |
| --- | --- | --- | --- | --- | --- | --- | --- | --- | --- |
| **Model** | | **R** | | **R²** | | **Adjusted R²** | | **RMSE** | |
| H₀ |  | 0.000 |  | 0.000 |  | 0.000 |  | 0.466 |  |
| H₁ |  | 0.372 |  | 0.139 |  | 0.053 |  | 0.454 |  |
|  | | | | | | | | | |

| **ANOVA** | | | | | | | | | | | | | |
| --- | --- | --- | --- | --- | --- | --- | --- | --- | --- | --- | --- | --- | --- |
| **Model** | |  | | **Sum of Squares** | | **df** | | **Mean Square** | | **F** | | **p** | |
| H₁ |  | Regression |  | 24.448 |  | 73 |  | 0.335 |  | 1.627 |  | 0.001 |  |
|  |  | Residual |  | 151.940 |  | 738 |  | 0.206 |  |  |  |  |  |
|  |  | Total |  | 176.388 |  | 811 |  |  |  |  |  |  |  |
|  | | | | | | | | | | | | | |
| Note.  The intercept model is omitted, as no meaningful information can be shown. | | | | | | | | | | | | | |

| **Coefficients** | | | | | | | | | | | | | |
| --- | --- | --- | --- | --- | --- | --- | --- | --- | --- | --- | --- | --- | --- |
| **Model** | |  | | **Unstandardized** | | **Standard Error** | | **Standardized** | | **t** | | **p** | |
| H₀ |  | (Intercept) |  | 0.319 |  | 0.016 |  |  |  | 19.489 |  | < .001 |  |
| H₁ |  | (Intercept) |  | 0.042 |  | 0.087 |  |  |  | 0.486 |  | 0.627 |  |
|  |  | PSS |  | 0.008 |  | 0.003 |  | 0.095 |  | 2.630 |  | 0.009 |  |
|  |  | A1Px |  | 9.842×10^-5^ |  | 6.285×10^-5^ |  | 0.073 |  | 1.566 |  | 0.118 |  |
|  |  | A1Py |  | 3.651×10^-5^ |  | 6.267×10^-5^ |  | 0.026 |  | 0.583 |  | 0.560 |  |
|  |  | A1Pz |  | 1.004×10^-4^ |  | 6.124×10^-5^ |  | 0.071 |  | 1.639 |  | 0.102 |  |
|  |  | A2Px |  | -1.502×10^-4^ |  | 7.504×10^-5^ |  | -0.097 |  | -2.001 |  | 0.046 |  |
|  |  | A2Py |  | -3.583×10^-5^ |  | 7.966×10^-5^ |  | -0.021 |  | -0.450 |  | 0.653 |  |
|  |  | A2Pz |  | 1.667×10^-5^ |  | 6.193×10^-5^ |  | 0.012 |  | 0.269 |  | 0.788 |  |
|  |  | A3Px |  | -4.947×10^-5^ |  | 5.744×10^-5^ |  | -0.039 |  | -0.861 |  | 0.389 |  |
|  |  | A3Py |  | 4.682×10^-5^ |  | 5.781×10^-5^ |  | 0.038 |  | 0.810 |  | 0.418 |  |
|  |  | A3Pz |  | -2.086×10^-5^ |  | 6.174×10^-5^ |  | -0.015 |  | -0.338 |  | 0.736 |  |
|  |  | B1Px |  | 1.213×10^-4^ |  | 7.056×10^-5^ |  | 0.078 |  | 1.719 |  | 0.086 |  |
|  |  | B1Py |  | 3.537×10^-5^ |  | 6.550×10^-5^ |  | 0.025 |  | 0.540 |  | 0.589 |  |
|  |  | B1Pz |  | 8.511×10^-6^ |  | 6.262×10^-5^ |  | 0.006 |  | 0.136 |  | 0.892 |  |
|  |  | B2Px |  | 5.427×10^-6^ |  | 6.616×10^-5^ |  | 0.004 |  | 0.082 |  | 0.935 |  |
|  |  | B2Py |  | -5.422×10^-5^ |  | 6.490×10^-5^ |  | -0.037 |  | -0.835 |  | 0.404 |  |
|  |  | B2Pz |  | 1.442×10^-5^ |  | 6.476×10^-5^ |  | 0.010 |  | 0.223 |  | 0.824 |  |
|  |  | B3Px |  | -6.730×10^-6^ |  | 5.930×10^-5^ |  | -0.005 |  | -0.114 |  | 0.910 |  |
|  |  | B3Py |  | -4.679×10^-5^ |  | 5.808×10^-5^ |  | -0.036 |  | -0.806 |  | 0.421 |  |
|  |  | B3Pz |  | -1.102×10^-4^ |  | 6.892×10^-5^ |  | -0.077 |  | -1.598 |  | 0.110 |  |
|  |  | C1Px |  | 1.131×10^-5^ |  | 5.574×10^-5^ |  | 0.009 |  | 0.203 |  | 0.839 |  |
|  |  | C1Py |  | -5.436×10^-5^ |  | 6.469×10^-5^ |  | -0.041 |  | -0.840 |  | 0.401 |  |
|  |  | C1Pz |  | 9.861×10^-5^ |  | 6.518×10^-5^ |  | 0.070 |  | 1.513 |  | 0.131 |  |
|  |  | C2Px |  | 1.252×10^-5^ |  | 6.790×10^-5^ |  | 0.009 |  | 0.184 |  | 0.854 |  |
|  |  | C2Py |  | -5.040×10^-5^ |  | 6.633×10^-5^ |  | -0.035 |  | -0.760 |  | 0.448 |  |
|  |  | C2Pz |  | -3.524×10^-5^ |  | 6.398×10^-5^ |  | -0.026 |  | -0.551 |  | 0.582 |  |
|  |  | C3Px |  | 2.905×10^-5^ |  | 5.986×10^-5^ |  | 0.023 |  | 0.485 |  | 0.628 |  |
|  |  | C3Py |  | 8.634×10^-6^ |  | 6.265×10^-5^ |  | 0.006 |  | 0.138 |  | 0.890 |  |
|  |  | C3Pz |  | -3.151×10^-6^ |  | 6.366×10^-5^ |  | -0.002 |  | -0.049 |  | 0.961 |  |
|  |  | D1Px |  | 6.298×10^-5^ |  | 6.258×10^-5^ |  | 0.049 |  | 1.006 |  | 0.315 |  |
|  |  | D1Py |  | -3.600×10^-5^ |  | 6.683×10^-5^ |  | -0.026 |  | -0.539 |  | 0.590 |  |
|  |  | D1Pz |  | 6.807×10^-5^ |  | 6.647×10^-5^ |  | 0.049 |  | 1.024 |  | 0.306 |  |
|  |  | D2Px |  | -7.400×10^-5^ |  | 6.831×10^-5^ |  | -0.049 |  | -1.083 |  | 0.279 |  |
|  |  | D2Py |  | -2.686×10^-5^ |  | 7.703×10^-5^ |  | -0.018 |  | -0.349 |  | 0.727 |  |
|  |  | D2Pz |  | -3.383×10^-5^ |  | 6.596×10^-5^ |  | -0.023 |  | -0.513 |  | 0.608 |  |
|  |  | D3Px |  | 7.303×10^-6^ |  | 7.047×10^-5^ |  | 0.005 |  | 0.104 |  | 0.917 |  |
|  |  | D3Py |  | -8.813×10^-5^ |  | 5.961×10^-5^ |  | -0.064 |  | -1.478 |  | 0.140 |  |
|  |  | D3Pz |  | -3.623×10^-5^ |  | 6.097×10^-5^ |  | -0.026 |  | -0.594 |  | 0.553 |  |
|  |  | A1Nx |  | 3.407×10^-5^ |  | 6.492×10^-5^ |  | 0.025 |  | 0.525 |  | 0.600 |  |
|  |  | A1Ny |  | -4.799×10^-5^ |  | 6.557×10^-5^ |  | -0.036 |  | -0.732 |  | 0.465 |  |
|  |  | A1Nz |  | 1.433×10^-4^ |  | 6.384×10^-5^ |  | 0.105 |  | 2.244 |  | 0.025 |  |
|  |  | A2Nx |  | -1.581×10^-4^ |  | 6.751×10^-5^ |  | -0.109 |  | -2.343 |  | 0.019 |  |
|  |  | A2Ny |  | -1.153×10^-4^ |  | 6.498×10^-5^ |  | -0.087 |  | -1.774 |  | 0.076 |  |
|  |  | A2Nz |  | 2.836×10^-6^ |  | 6.985×10^-5^ |  | 0.002 |  | 0.041 |  | 0.968 |  |
|  |  | A3Nx |  | -7.622×10^-5^ |  | 6.195×10^-5^ |  | -0.059 |  | -1.230 |  | 0.219 |  |
|  |  | A3Ny |  | 1.088×10^-5^ |  | 6.702×10^-5^ |  | 0.008 |  | 0.162 |  | 0.871 |  |
|  |  | A3Nz |  | 9.188×10^-5^ |  | 6.116×10^-5^ |  | 0.074 |  | 1.502 |  | 0.133 |  |
|  |  | B1Nx |  | 2.225×10^-5^ |  | 6.356×10^-5^ |  | 0.016 |  | 0.350 |  | 0.726 |  |
|  |  | B1Ny |  | 8.513×10^-5^ |  | 6.372×10^-5^ |  | 0.066 |  | 1.336 |  | 0.182 |  |
|  |  | B1Nz |  | 2.528×10^-4^ |  | 6.221×10^-5^ |  | 0.195 |  | 4.063 |  | < .001 |  |
|  |  | B2Nx |  | -4.247×10^-5^ |  | 5.679×10^-5^ |  | -0.033 |  | -0.748 |  | 0.455 |  |
|  |  | B2Ny |  | -4.377×10^-5^ |  | 6.913×10^-5^ |  | -0.031 |  | -0.633 |  | 0.527 |  |
|  |  | B2Nz |  | 1.087×10^-4^ |  | 5.988×10^-5^ |  | 0.085 |  | 1.815 |  | 0.070 |  |
|  |  | B3Nx |  | 2.160×10^-5^ |  | 6.871×10^-5^ |  | 0.016 |  | 0.314 |  | 0.753 |  |
|  |  | B3Ny |  | -5.142×10^-5^ |  | 6.732×10^-5^ |  | -0.035 |  | -0.764 |  | 0.445 |  |
|  |  | B3Nz |  | -1.951×10^-5^ |  | 7.030×10^-5^ |  | -0.014 |  | -0.278 |  | 0.781 |  |
|  |  | C1Nx |  | 8.255×10^-5^ |  | 7.353×10^-5^ |  | 0.058 |  | 1.123 |  | 0.262 |  |
|  |  | C1Ny |  | 1.516×10^-4^ |  | 6.687×10^-5^ |  | 0.102 |  | 2.267 |  | 0.024 |  |
|  |  | C1Nz |  | 2.706×10^-5^ |  | 7.613×10^-5^ |  | 0.017 |  | 0.355 |  | 0.722 |  |
|  |  | C2Nx |  | 4.532×10^-5^ |  | 6.151×10^-5^ |  | 0.035 |  | 0.737 |  | 0.461 |  |
|  |  | C2Ny |  | 2.062×10^-5^ |  | 6.045×10^-5^ |  | 0.016 |  | 0.341 |  | 0.733 |  |
|  |  | C2Nz |  | -8.505×10^-6^ |  | 6.257×10^-5^ |  | -0.006 |  | -0.136 |  | 0.892 |  |
|  |  | C3Nx |  | 6.160×10^-5^ |  | 6.593×10^-5^ |  | 0.047 |  | 0.934 |  | 0.350 |  |
|  |  | C3Ny |  | -5.782×10^-5^ |  | 6.811×10^-5^ |  | -0.043 |  | -0.849 |  | 0.396 |  |
|  |  | C3Nz |  | -7.622×10^-5^ |  | 6.329×10^-5^ |  | -0.060 |  | -1.204 |  | 0.229 |  |
|  |  | D1Nx |  | -7.178×10^-6^ |  | 6.575×10^-5^ |  | -0.005 |  | -0.109 |  | 0.913 |  |
|  |  | D1Ny |  | 8.454×10^-5^ |  | 6.507×10^-5^ |  | 0.063 |  | 1.299 |  | 0.194 |  |
|  |  | D1Nz |  | -5.148×10^-5^ |  | 6.622×10^-5^ |  | -0.039 |  | -0.777 |  | 0.437 |  |
|  |  | D2Nx |  | 2.125×10^-5^ |  | 5.975×10^-5^ |  | 0.016 |  | 0.356 |  | 0.722 |  |
|  |  | D2Ny |  | -1.021×10^-4^ |  | 6.312×10^-5^ |  | -0.076 |  | -1.617 |  | 0.106 |  |
|  |  | D2Nz |  | -1.195×10^-4^ |  | 6.715×10^-5^ |  | -0.089 |  | -1.780 |  | 0.075 |  |
|  |  | D3Nx |  | -5.000×10^-5^ |  | 6.371×10^-5^ |  | -0.038 |  | -0.785 |  | 0.433 |  |
|  |  | D3Ny |  | 5.770×10^-5^ |  | 6.729×10^-5^ |  | 0.040 |  | 0.858 |  | 0.391 |  |
|  |  | D3Nz |  | -1.056×10^-4^ |  | 6.428×10^-5^ |  | -0.080 |  | -1.643 |  | 0.101 |  |
|  | | | | | | | | | | | | | |

Quitting – PSS

## Linear Regression

| **Model Summary - quitting** | | | | | | | | | |
| --- | --- | --- | --- | --- | --- | --- | --- | --- | --- |
| **Model** | | **R** | | **R²** | | **Adjusted R²** | | **RMSE** | |
| H₀ |  | 0.000 |  | 0.000 |  | 0.000 |  | 0.420 |  |
| H₁ |  | 0.189 |  | 0.036 |  | 0.034 |  | 0.412 |  |
|  | | | | | | | | | |

| **ANOVA** | | | | | | | | | | | | | |
| --- | --- | --- | --- | --- | --- | --- | --- | --- | --- | --- | --- | --- | --- |
| **Model** | |  | | **Sum of Squares** | | **df** | | **Mean Square** | | **F** | | **p** | |
| H₁ |  | Regression |  | 5.093 |  | 1 |  | 5.093 |  | 29.944 |  | < .001 |  |
|  |  | Residual |  | 137.758 |  | 810 |  | 0.170 |  |  |  |  |  |
|  |  | Total |  | 142.851 |  | 811 |  |  |  |  |  |  |  |
|  | | | | | | | | | | | | | |
| Note.  The intercept model is omitted, as no meaningful information can be shown. | | | | | | | | | | | | | |

| **Coefficients** | | | | | | | | | | | | | |
| --- | --- | --- | --- | --- | --- | --- | --- | --- | --- | --- | --- | --- | --- |
| **Model** | |  | | **Unstandardized** | | **Standard Error** | | **Standardized** | | **t** | | **p** | |
| H₀ |  | (Intercept) |  | 0.228 |  | 0.015 |  |  |  | 15.469 |  | < .001 |  |
| H₁ |  | (Intercept) |  | -0.168 |  | 0.074 |  |  |  | -2.280 |  | 0.023 |  |
|  |  | PSS |  | 0.014 |  | 0.003 |  | 0.189 |  | 5.472 |  | < .001 |  |
|  | | | | | | | | | | | | | |

Quitting IMAGE-BASED METHOD Alone (above)

Quitting PSS + Image-based method

## Linear Regression

| **Model Summary - quitting** | | | | | | | | | |
| --- | --- | --- | --- | --- | --- | --- | --- | --- | --- |
| **Model** | | **R** | | **R²** | | **Adjusted R²** | | **RMSE** | |
| H₀ |  | 0.000 |  | 0.000 |  | 0.000 |  | 0.420 |  |
| H₁ |  | 0.393 |  | 0.155 |  | 0.071 |  | 0.405 |  |
|  | | | | | | | | | |

| **ANOVA** | | | | | | | | | | | | | |
| --- | --- | --- | --- | --- | --- | --- | --- | --- | --- | --- | --- | --- | --- |
| **Model** | |  | | **Sum of Squares** | | **df** | | **Mean Square** | | **F** | | **p** | |
| H₁ |  | Regression |  | 22.079 |  | 73 |  | 0.302 |  | 1.848 |  | < .001 |  |
|  |  | Residual |  | 120.772 |  | 738 |  | 0.164 |  |  |  |  |  |
|  |  | Total |  | 142.851 |  | 811 |  |  |  |  |  |  |  |
|  | | | | | | | | | | | | | |
| Note.  The intercept model is omitted, as no meaningful information can be shown. | | | | | | | | | | | | | |

| **Coefficients** | | | | | | | | | | | | | |
| --- | --- | --- | --- | --- | --- | --- | --- | --- | --- | --- | --- | --- | --- |
| **Model** | |  | | **Unstandardized** | | **Standard Error** | | **Standardized** | | **t** | | **p** | |
| H₀ |  | (Intercept) |  | 0.228 |  | 0.015 |  |  |  | 15.469 |  | < .001 |  |
| H₁ |  | (Intercept) |  | -0.174 |  | 0.078 |  |  |  | -2.239 |  | 0.025 |  |
|  |  | A1Nx |  | 3.173×10^-5^ |  | 5.788×10^-5^ |  | 0.026 |  | 0.548 |  | 0.584 |  |
|  |  | A1Ny |  | 4.380×10^-5^ |  | 5.846×10^-5^ |  | 0.036 |  | 0.749 |  | 0.454 |  |
|  |  | A1Nz |  | -5.866×10^-5^ |  | 5.692×10^-5^ |  | -0.048 |  | -1.031 |  | 0.303 |  |
|  |  | A2Nx |  | 1.619×10^-4^ |  | 6.018×10^-5^ |  | 0.124 |  | 2.689 |  | 0.007 |  |
|  |  | A2Ny |  | 2.893×10^-6^ |  | 5.793×10^-5^ |  | 0.002 |  | 0.050 |  | 0.960 |  |
|  |  | A2Nz |  | -2.061×10^-5^ |  | 6.227×10^-5^ |  | -0.016 |  | -0.331 |  | 0.741 |  |
|  |  | A3Nx |  | 4.959×10^-5^ |  | 5.524×10^-5^ |  | 0.043 |  | 0.898 |  | 0.370 |  |
|  |  | A3Ny |  | -1.626×10^-5^ |  | 5.975×10^-5^ |  | -0.013 |  | -0.272 |  | 0.786 |  |
|  |  | A3Nz |  | 2.485×10^-5^ |  | 5.453×10^-5^ |  | 0.022 |  | 0.456 |  | 0.649 |  |
|  |  | B1Nx |  | 4.539×10^-5^ |  | 5.666×10^-5^ |  | 0.037 |  | 0.801 |  | 0.423 |  |
|  |  | B1Ny |  | 7.903×10^-6^ |  | 5.681×10^-5^ |  | 0.007 |  | 0.139 |  | 0.889 |  |
|  |  | B1Nz |  | 2.556×10^-5^ |  | 5.546×10^-5^ |  | 0.022 |  | 0.461 |  | 0.645 |  |
|  |  | B2Nx |  | -6.642×10^-5^ |  | 5.063×10^-5^ |  | -0.058 |  | -1.312 |  | 0.190 |  |
|  |  | B2Ny |  | -6.821×10^-5^ |  | 6.163×10^-5^ |  | -0.054 |  | -1.107 |  | 0.269 |  |
|  |  | B2Nz |  | -7.419×10^-5^ |  | 5.338×10^-5^ |  | -0.065 |  | -1.390 |  | 0.165 |  |
|  |  | B3Nx |  | -4.243×10^-5^ |  | 6.126×10^-5^ |  | -0.035 |  | -0.693 |  | 0.489 |  |
|  |  | B3Ny |  | -1.000×10^-4^ |  | 6.002×10^-5^ |  | -0.075 |  | -1.667 |  | 0.096 |  |
|  |  | B3Nz |  | 5.813×10^-6^ |  | 6.267×10^-5^ |  | 0.004 |  | 0.093 |  | 0.926 |  |
|  |  | C1Nx |  | -1.669×10^-4^ |  | 6.555×10^-5^ |  | -0.130 |  | -2.547 |  | 0.011 |  |
|  |  | C1Ny |  | 5.220×10^-5^ |  | 5.962×10^-5^ |  | 0.039 |  | 0.875 |  | 0.382 |  |
|  |  | C1Nz |  | 4.532×10^-5^ |  | 6.788×10^-5^ |  | 0.031 |  | 0.668 |  | 0.505 |  |
|  |  | C2Nx |  | 4.476×10^-5^ |  | 5.484×10^-5^ |  | 0.039 |  | 0.816 |  | 0.415 |  |
|  |  | C2Ny |  | 6.941×10^-5^ |  | 5.389×10^-5^ |  | 0.061 |  | 1.288 |  | 0.198 |  |
|  |  | C2Nz |  | 4.727×10^-5^ |  | 5.578×10^-5^ |  | 0.039 |  | 0.847 |  | 0.397 |  |
|  |  | C3Nx |  | 5.447×10^-5^ |  | 5.878×10^-5^ |  | 0.047 |  | 0.927 |  | 0.354 |  |
|  |  | C3Ny |  | -2.029×10^-5^ |  | 6.072×10^-5^ |  | -0.017 |  | -0.334 |  | 0.738 |  |
|  |  | C3Nz |  | -6.477×10^-5^ |  | 5.643×10^-5^ |  | -0.057 |  | -1.148 |  | 0.251 |  |
|  |  | D1Nx |  | -2.823×10^-5^ |  | 5.862×10^-5^ |  | -0.024 |  | -0.482 |  | 0.630 |  |
|  |  | D1Ny |  | -1.994×10^-5^ |  | 5.801×10^-5^ |  | -0.016 |  | -0.344 |  | 0.731 |  |
|  |  | D1Nz |  | 1.429×10^-4^ |  | 5.904×10^-5^ |  | 0.120 |  | 2.421 |  | 0.016 |  |
|  |  | D2Nx |  | -1.213×10^-5^ |  | 5.327×10^-5^ |  | -0.010 |  | -0.228 |  | 0.820 |  |
|  |  | D2Ny |  | 1.767×10^-4^ |  | 5.628×10^-5^ |  | 0.147 |  | 3.140 |  | 0.002 |  |
|  |  | D2Nz |  | 1.768×10^-5^ |  | 5.987×10^-5^ |  | 0.015 |  | 0.295 |  | 0.768 |  |
|  |  | D3Nx |  | -3.696×10^-5^ |  | 5.680×10^-5^ |  | -0.031 |  | -0.651 |  | 0.515 |  |
|  |  | D3Ny |  | -1.743×10^-4^ |  | 5.999×10^-5^ |  | -0.133 |  | -2.905 |  | 0.004 |  |
|  |  | D3Nz |  | 1.097×10^-4^ |  | 5.731×10^-5^ |  | 0.092 |  | 1.914 |  | 0.056 |  |
|  |  | A1Px |  | 1.011×10^-4^ |  | 5.603×10^-5^ |  | 0.084 |  | 1.805 |  | 0.072 |  |
|  |  | A1Py |  | 2.855×10^-5^ |  | 5.587×10^-5^ |  | 0.022 |  | 0.511 |  | 0.609 |  |
|  |  | A1Pz |  | -7.739×10^-5^ |  | 5.459×10^-5^ |  | -0.061 |  | -1.418 |  | 0.157 |  |
|  |  | A2Px |  | 2.187×10^-6^ |  | 6.690×10^-5^ |  | 0.002 |  | 0.033 |  | 0.974 |  |
|  |  | A2Py |  | -1.257×10^-4^ |  | 7.102×10^-5^ |  | -0.083 |  | -1.769 |  | 0.077 |  |
|  |  | A2Pz |  | -4.517×10^-6^ |  | 5.521×10^-5^ |  | -0.004 |  | -0.082 |  | 0.935 |  |
|  |  | A3Px |  | 3.735×10^-5^ |  | 5.121×10^-5^ |  | 0.033 |  | 0.729 |  | 0.466 |  |
|  |  | A3Py |  | 1.770×10^-5^ |  | 5.154×10^-5^ |  | 0.016 |  | 0.343 |  | 0.731 |  |
|  |  | A3Pz |  | 1.254×10^-5^ |  | 5.504×10^-5^ |  | 0.010 |  | 0.228 |  | 0.820 |  |
|  |  | B1Px |  | 2.073×10^-6^ |  | 6.291×10^-5^ |  | 0.001 |  | 0.033 |  | 0.974 |  |
|  |  | B1Py |  | 1.507×10^-5^ |  | 5.840×10^-5^ |  | 0.012 |  | 0.258 |  | 0.796 |  |
|  |  | B1Pz |  | -2.672×10^-5^ |  | 5.583×10^-5^ |  | -0.022 |  | -0.479 |  | 0.632 |  |
|  |  | B2Px |  | -7.110×10^-5^ |  | 5.899×10^-5^ |  | -0.054 |  | -1.205 |  | 0.228 |  |
|  |  | B2Py |  | -5.756×10^-5^ |  | 5.786×10^-5^ |  | -0.044 |  | -0.995 |  | 0.320 |  |
|  |  | B2Pz |  | -1.241×10^-4^ |  | 5.774×10^-5^ |  | -0.097 |  | -2.150 |  | 0.032 |  |
|  |  | B3Px |  | 4.321×10^-5^ |  | 5.287×10^-5^ |  | 0.038 |  | 0.817 |  | 0.414 |  |
|  |  | B3Py |  | 6.943×10^-5^ |  | 5.178×10^-5^ |  | 0.060 |  | 1.341 |  | 0.180 |  |
|  |  | B3Pz |  | -6.257×10^-5^ |  | 6.145×10^-5^ |  | -0.048 |  | -1.018 |  | 0.309 |  |
|  |  | C1Px |  | -5.555×10^-5^ |  | 4.970×10^-5^ |  | -0.050 |  | -1.118 |  | 0.264 |  |
|  |  | C1Py |  | -6.823×10^-5^ |  | 5.767×10^-5^ |  | -0.057 |  | -1.183 |  | 0.237 |  |
|  |  | C1Pz |  | -2.135×10^-5^ |  | 5.811×10^-5^ |  | -0.017 |  | -0.367 |  | 0.713 |  |
|  |  | C2Px |  | 1.152×10^-4^ |  | 6.053×10^-5^ |  | 0.091 |  | 1.903 |  | 0.057 |  |
|  |  | C2Py |  | 6.793×10^-5^ |  | 5.914×10^-5^ |  | 0.053 |  | 1.149 |  | 0.251 |  |
|  |  | C2Pz |  | -3.705×10^-5^ |  | 5.704×10^-5^ |  | -0.030 |  | -0.649 |  | 0.516 |  |
|  |  | C3Px |  | 7.321×10^-5^ |  | 5.337×10^-5^ |  | 0.063 |  | 1.372 |  | 0.171 |  |
|  |  | C3Py |  | 4.317×10^-6^ |  | 5.585×10^-5^ |  | 0.004 |  | 0.077 |  | 0.938 |  |
|  |  | C3Pz |  | 2.721×10^-5^ |  | 5.676×10^-5^ |  | 0.024 |  | 0.479 |  | 0.632 |  |
|  |  | D1Px |  | -1.190×10^-5^ |  | 5.579×10^-5^ |  | -0.010 |  | -0.213 |  | 0.831 |  |
|  |  | D1Py |  | -4.740×10^-5^ |  | 5.958×10^-5^ |  | -0.038 |  | -0.796 |  | 0.427 |  |
|  |  | D1Pz |  | -4.582×10^-5^ |  | 5.926×10^-5^ |  | -0.037 |  | -0.773 |  | 0.440 |  |
|  |  | D2Px |  | 5.996×10^-5^ |  | 6.091×10^-5^ |  | 0.044 |  | 0.984 |  | 0.325 |  |
|  |  | D2Py |  | -1.916×10^-5^ |  | 6.868×10^-5^ |  | -0.014 |  | -0.279 |  | 0.780 |  |
|  |  | D2Pz |  | 9.097×10^-5^ |  | 5.880×10^-5^ |  | 0.070 |  | 1.547 |  | 0.122 |  |
|  |  | D3Px |  | -4.370×10^-5^ |  | 6.283×10^-5^ |  | -0.035 |  | -0.695 |  | 0.487 |  |
|  |  | D3Py |  | 5.347×10^-6^ |  | 5.315×10^-5^ |  | 0.004 |  | 0.101 |  | 0.920 |  |
|  |  | D3Pz |  | -6.807×10^-5^ |  | 5.436×10^-5^ |  | -0.055 |  | -1.252 |  | 0.211 |  |
|  |  | PSS |  | 0.013 |  | 0.003 |  | 0.181 |  | 5.053 |  | < .001 |  |
|  | | | | | | | | | | | | | |

Burnout – Coping Alone

## Linear Regression

| **Model Summary - burnout** | | | | | | | | | |
| --- | --- | --- | --- | --- | --- | --- | --- | --- | --- |
| **Model** | | **R** | | **R²** | | **Adjusted R²** | | **RMSE** | |
| H₀ |  | 0.000 |  | 0.000 |  | 0.000 |  | 0.466 |  |
| H₁ |  | 0.217 |  | 0.047 |  | 0.030 |  | 0.459 |  |
|  | | | | | | | | | |

| **ANOVA** | | | | | | | | | | | | | |
| --- | --- | --- | --- | --- | --- | --- | --- | --- | --- | --- | --- | --- | --- |
| **Model** | |  | | **Sum of Squares** | | **df** | | **Mean Square** | | **F** | | **p** | |
| H₁ |  | Regression |  | 8.269 |  | 14 |  | 0.591 |  | 2.800 |  | < .001 |  |
|  |  | Residual |  | 168.119 |  | 797 |  | 0.211 |  |  |  |  |  |
|  |  | Total |  | 176.388 |  | 811 |  |  |  |  |  |  |  |
|  | | | | | | | | | | | | | |
| Note.  The intercept model is omitted, as no meaningful information can be shown. | | | | | | | | | | | | | |

| **Coefficients** | | | | | | | | | | | | | |
| --- | --- | --- | --- | --- | --- | --- | --- | --- | --- | --- | --- | --- | --- |
| **Model** | |  | | **Unstandardized** | | **Standard Error** | | **Standardized** | | **t** | | **p** | |
| H₀ |  | (Intercept) |  | 0.319 |  | 0.016 |  |  |  | 19.489 |  | < .001 |  |
| H₁ |  | (Intercept) |  | 0.403 |  | 0.070 |  |  |  | 5.780 |  | < .001 |  |
|  |  | selfDistraction |  | 0.021 |  | 0.013 |  | 0.075 |  | 1.622 |  | 0.105 |  |
|  |  | activeCoping |  | -0.016 |  | 0.014 |  | -0.057 |  | -1.168 |  | 0.243 |  |
|  |  | Denial |  | -0.010 |  | 0.013 |  | -0.040 |  | -0.765 |  | 0.445 |  |
|  |  | substanceUse |  | -0.017 |  | 0.012 |  | -0.073 |  | -1.454 |  | 0.146 |  |
|  |  | useEmotionalSupport |  | -0.006 |  | 0.014 |  | -0.023 |  | -0.434 |  | 0.664 |  |
|  |  | useInstrumentalSupport |  | 0.011 |  | 0.014 |  | 0.043 |  | 0.780 |  | 0.436 |  |
|  |  | behavioralDisengagement |  | -0.019 |  | 0.014 |  | -0.075 |  | -1.304 |  | 0.192 |  |
|  |  | venting |  | 0.004 |  | 0.014 |  | 0.014 |  | 0.276 |  | 0.783 |  |
|  |  | positiveReframing |  | -0.022 |  | 0.014 |  | -0.080 |  | -1.579 |  | 0.115 |  |
|  |  | planning |  | -0.019 |  | 0.017 |  | -0.072 |  | -1.120 |  | 0.263 |  |
|  |  | humor |  | -0.003 |  | 0.011 |  | -0.013 |  | -0.272 |  | 0.785 |  |
|  |  | acceptance |  | 0.006 |  | 0.012 |  | 0.020 |  | 0.443 |  | 0.658 |  |
|  |  | religion |  | -0.015 |  | 0.010 |  | -0.065 |  | -1.502 |  | 0.133 |  |
|  |  | selfBlame |  | 0.061 |  | 0.017 |  | 0.219 |  | 3.643 |  | < .001 |  |
|  | | | | | | | | | | | | | |

Burnout Coping + Image-based method

## Linear Regression

| **Model Summary - burnout** | | | | | | | | | |
| --- | --- | --- | --- | --- | --- | --- | --- | --- | --- |
| **Model** | | **R** | | **R²** | | **Adjusted R²** | | **RMSE** | |
| H₀ |  | 0.000 |  | 0.000 |  | 0.000 |  | 0.466 |  |
| H₁ |  | 0.406 |  | 0.165 |  | 0.066 |  | 0.451 |  |
|  | | | | | | | | | |

| **ANOVA** | | | | | | | | | | | | | |
| --- | --- | --- | --- | --- | --- | --- | --- | --- | --- | --- | --- | --- | --- |
| **Model** | |  | | **Sum of Squares** | | **df** | | **Mean Square** | | **F** | | **p** | |
| H₁ |  | Regression |  | 29.089 |  | 86 |  | 0.338 |  | 1.665 |  | < .001 |  |
|  |  | Residual |  | 147.299 |  | 725 |  | 0.203 |  |  |  |  |  |
|  |  | Total |  | 176.388 |  | 811 |  |  |  |  |  |  |  |
|  | | | | | | | | | | | | | |
| Note.  The intercept model is omitted, as no meaningful information can be shown. | | | | | | | | | | | | | |

| **Coefficients** | | | | | | | | | | | | | |
| --- | --- | --- | --- | --- | --- | --- | --- | --- | --- | --- | --- | --- | --- |
| **Model** | |  | | **Unstandardized** | | **Standard Error** | | **Standardized** | | **t** | | **p** | |
| H₀ |  | (Intercept) |  | 0.319 |  | 0.016 |  |  |  | 19.489 |  | < .001 |  |
| H₁ |  | (Intercept) |  | 0.374 |  | 0.074 |  |  |  | 5.060 |  | < .001 |  |
|  |  | A1Px |  | 1.227×10^-4^ |  | 6.288×10^-5^ |  | 0.092 |  | 1.951 |  | 0.051 |  |
|  |  | A1Py |  | 2.899×10^-5^ |  | 6.281×10^-5^ |  | 0.020 |  | 0.461 |  | 0.645 |  |
|  |  | A1Pz |  | 9.704×10^-5^ |  | 6.194×10^-5^ |  | 0.069 |  | 1.567 |  | 0.118 |  |
|  |  | A2Px |  | -1.371×10^-4^ |  | 7.535×10^-5^ |  | -0.089 |  | -1.819 |  | 0.069 |  |
|  |  | A2Py |  | -3.731×10^-5^ |  | 7.960×10^-5^ |  | -0.022 |  | -0.469 |  | 0.639 |  |
|  |  | A2Pz |  | 1.543×10^-5^ |  | 6.177×10^-5^ |  | 0.011 |  | 0.250 |  | 0.803 |  |
|  |  | A3Px |  | -5.930×10^-5^ |  | 5.766×10^-5^ |  | -0.047 |  | -1.029 |  | 0.304 |  |
|  |  | A3Py |  | 3.120×10^-5^ |  | 5.778×10^-5^ |  | 0.025 |  | 0.540 |  | 0.589 |  |
|  |  | A3Pz |  | -2.383×10^-5^ |  | 6.172×10^-5^ |  | -0.018 |  | -0.386 |  | 0.700 |  |
|  |  | B1Px |  | 1.146×10^-4^ |  | 7.094×10^-5^ |  | 0.073 |  | 1.615 |  | 0.107 |  |
|  |  | B1Py |  | 1.901×10^-5^ |  | 6.575×10^-5^ |  | 0.013 |  | 0.289 |  | 0.773 |  |
|  |  | B1Pz |  | 1.591×10^-5^ |  | 6.254×10^-5^ |  | 0.012 |  | 0.254 |  | 0.799 |  |
|  |  | B2Px |  | 2.541×10^-5^ |  | 6.635×10^-5^ |  | 0.017 |  | 0.383 |  | 0.702 |  |
|  |  | B2Py |  | -2.495×10^-5^ |  | 6.529×10^-5^ |  | -0.017 |  | -0.382 |  | 0.702 |  |
|  |  | B2Pz |  | 1.476×10^-5^ |  | 6.518×10^-5^ |  | 0.010 |  | 0.226 |  | 0.821 |  |
|  |  | B3Px |  | -3.003×10^-5^ |  | 5.920×10^-5^ |  | -0.024 |  | -0.507 |  | 0.612 |  |
|  |  | B3Py |  | -6.577×10^-5^ |  | 5.796×10^-5^ |  | -0.051 |  | -1.135 |  | 0.257 |  |
|  |  | B3Pz |  | -1.072×10^-4^ |  | 6.915×10^-5^ |  | -0.075 |  | -1.550 |  | 0.122 |  |
|  |  | C1Px |  | -2.367×10^-6^ |  | 5.598×10^-5^ |  | -0.002 |  | -0.042 |  | 0.966 |  |
|  |  | C1Py |  | -4.841×10^-5^ |  | 6.495×10^-5^ |  | -0.036 |  | -0.745 |  | 0.456 |  |
|  |  | C1Pz |  | 9.665×10^-5^ |  | 6.526×10^-5^ |  | 0.069 |  | 1.481 |  | 0.139 |  |
|  |  | C2Px |  | 6.949×10^-6^ |  | 6.801×10^-5^ |  | 0.005 |  | 0.102 |  | 0.919 |  |
|  |  | C2Py |  | -4.101×10^-5^ |  | 6.630×10^-5^ |  | -0.029 |  | -0.619 |  | 0.536 |  |
|  |  | C2Pz |  | -5.835×10^-5^ |  | 6.458×10^-5^ |  | -0.043 |  | -0.904 |  | 0.367 |  |
|  |  | C3Px |  | 3.037×10^-5^ |  | 5.970×10^-5^ |  | 0.024 |  | 0.509 |  | 0.611 |  |
|  |  | C3Py |  | 3.170×10^-5^ |  | 6.299×10^-5^ |  | 0.024 |  | 0.503 |  | 0.615 |  |
|  |  | C3Pz |  | -2.622×10^-5^ |  | 6.426×10^-5^ |  | -0.020 |  | -0.408 |  | 0.683 |  |
|  |  | D1Px |  | 6.238×10^-5^ |  | 6.264×10^-5^ |  | 0.048 |  | 0.996 |  | 0.320 |  |
|  |  | D1Py |  | -1.295×10^-5^ |  | 6.690×10^-5^ |  | -0.009 |  | -0.194 |  | 0.847 |  |
|  |  | D1Pz |  | 3.648×10^-5^ |  | 6.680×10^-5^ |  | 0.026 |  | 0.546 |  | 0.585 |  |
|  |  | D2Px |  | -5.739×10^-5^ |  | 6.827×10^-5^ |  | -0.038 |  | -0.841 |  | 0.401 |  |
|  |  | D2Py |  | -4.153×10^-6^ |  | 7.770×10^-5^ |  | -0.003 |  | -0.053 |  | 0.957 |  |
|  |  | D2Pz |  | -1.255×10^-5^ |  | 6.615×10^-5^ |  | -0.009 |  | -0.190 |  | 0.850 |  |
|  |  | D3Px |  | 1.571×10^-5^ |  | 7.122×10^-5^ |  | 0.011 |  | 0.221 |  | 0.825 |  |
|  |  | D3Py |  | -9.772×10^-5^ |  | 5.982×10^-5^ |  | -0.071 |  | -1.633 |  | 0.103 |  |
|  |  | D3Pz |  | -2.153×10^-5^ |  | 6.112×10^-5^ |  | -0.016 |  | -0.352 |  | 0.725 |  |
|  |  | A1Nx |  | 2.930×10^-5^ |  | 6.508×10^-5^ |  | 0.022 |  | 0.450 |  | 0.653 |  |
|  |  | A1Ny |  | -1.408×10^-6^ |  | 6.585×10^-5^ |  | -0.001 |  | -0.021 |  | 0.983 |  |
|  |  | A1Nz |  | 1.388×10^-4^ |  | 6.381×10^-5^ |  | 0.102 |  | 2.175 |  | 0.030 |  |
|  |  | A2Nx |  | -1.594×10^-4^ |  | 6.746×10^-5^ |  | -0.110 |  | -2.363 |  | 0.018 |  |
|  |  | A2Ny |  | -1.220×10^-4^ |  | 6.489×10^-5^ |  | -0.093 |  | -1.880 |  | 0.060 |  |
|  |  | A2Nz |  | 1.957×10^-5^ |  | 7.022×10^-5^ |  | 0.014 |  | 0.279 |  | 0.781 |  |
|  |  | A3Nx |  | -9.867×10^-5^ |  | 6.226×10^-5^ |  | -0.076 |  | -1.585 |  | 0.113 |  |
|  |  | A3Ny |  | 1.962×10^-5^ |  | 6.721×10^-5^ |  | 0.015 |  | 0.292 |  | 0.770 |  |
|  |  | A3Nz |  | 3.285×10^-5^ |  | 6.226×10^-5^ |  | 0.026 |  | 0.528 |  | 0.598 |  |
|  |  | B1Nx |  | 1.294×10^-5^ |  | 6.338×10^-5^ |  | 0.010 |  | 0.204 |  | 0.838 |  |
|  |  | B1Ny |  | 7.828×10^-5^ |  | 6.398×10^-5^ |  | 0.061 |  | 1.224 |  | 0.221 |  |
|  |  | B1Nz |  | 2.307×10^-4^ |  | 6.227×10^-5^ |  | 0.178 |  | 3.705 |  | < .001 |  |
|  |  | B2Nx |  | -2.876×10^-5^ |  | 5.721×10^-5^ |  | -0.022 |  | -0.503 |  | 0.615 |  |
|  |  | B2Ny |  | -4.442×10^-5^ |  | 6.927×10^-5^ |  | -0.032 |  | -0.641 |  | 0.522 |  |
|  |  | B2Nz |  | 1.090×10^-4^ |  | 6.026×10^-5^ |  | 0.086 |  | 1.810 |  | 0.071 |  |
|  |  | B3Nx |  | 2.730×10^-5^ |  | 6.890×10^-5^ |  | 0.020 |  | 0.396 |  | 0.692 |  |
|  |  | B3Ny |  | -3.720×10^-5^ |  | 6.796×10^-5^ |  | -0.025 |  | -0.547 |  | 0.584 |  |
|  |  | B3Nz |  | -2.799×10^-5^ |  | 7.021×10^-5^ |  | -0.019 |  | -0.399 |  | 0.690 |  |
|  |  | C1Nx |  | 7.757×10^-5^ |  | 7.364×10^-5^ |  | 0.054 |  | 1.053 |  | 0.293 |  |
|  |  | C1Ny |  | 1.229×10^-4^ |  | 6.705×10^-5^ |  | 0.082 |  | 1.833 |  | 0.067 |  |
|  |  | C1Nz |  | 3.754×10^-5^ |  | 7.648×10^-5^ |  | 0.023 |  | 0.491 |  | 0.624 |  |
|  |  | C2Nx |  | 5.101×10^-5^ |  | 6.154×10^-5^ |  | 0.040 |  | 0.829 |  | 0.407 |  |
|  |  | C2Ny |  | 4.168×10^-5^ |  | 6.039×10^-5^ |  | 0.033 |  | 0.690 |  | 0.490 |  |
|  |  | C2Nz |  | -2.635×10^-5^ |  | 6.245×10^-5^ |  | -0.020 |  | -0.422 |  | 0.673 |  |
|  |  | C3Nx |  | 7.655×10^-5^ |  | 6.624×10^-5^ |  | 0.059 |  | 1.156 |  | 0.248 |  |
|  |  | C3Ny |  | -4.962×10^-5^ |  | 6.784×10^-5^ |  | -0.037 |  | -0.731 |  | 0.465 |  |
|  |  | C3Nz |  | -8.686×10^-5^ |  | 6.349×10^-5^ |  | -0.068 |  | -1.368 |  | 0.172 |  |
|  |  | D1Nx |  | -1.084×10^-5^ |  | 6.561×10^-5^ |  | -0.008 |  | -0.165 |  | 0.869 |  |
|  |  | D1Ny |  | 1.193×10^-4^ |  | 6.598×10^-5^ |  | 0.089 |  | 1.808 |  | 0.071 |  |
|  |  | D1Nz |  | -6.189×10^-5^ |  | 6.686×10^-5^ |  | -0.047 |  | -0.926 |  | 0.355 |  |
|  |  | D2Nx |  | 2.307×10^-5^ |  | 5.967×10^-5^ |  | 0.018 |  | 0.387 |  | 0.699 |  |
|  |  | D2Ny |  | -9.323×10^-5^ |  | 6.307×10^-5^ |  | -0.070 |  | -1.478 |  | 0.140 |  |
|  |  | D2Nz |  | -8.422×10^-5^ |  | 6.778×10^-5^ |  | -0.062 |  | -1.243 |  | 0.214 |  |
|  |  | D3Nx |  | -6.522×10^-5^ |  | 6.382×10^-5^ |  | -0.049 |  | -1.022 |  | 0.307 |  |
|  |  | D3Ny |  | 5.194×10^-5^ |  | 6.732×10^-5^ |  | 0.036 |  | 0.772 |  | 0.441 |  |
|  |  | D3Nz |  | -8.685×10^-5^ |  | 6.417×10^-5^ |  | -0.065 |  | -1.353 |  | 0.176 |  |
|  |  | selfDistraction |  | 0.016 |  | 0.013 |  | 0.055 |  | 1.157 |  | 0.247 |  |
|  |  | activeCoping |  | -0.010 |  | 0.015 |  | -0.035 |  | -0.679 |  | 0.497 |  |
|  |  | Denial |  | -0.011 |  | 0.014 |  | -0.047 |  | -0.843 |  | 0.399 |  |
|  |  | substanceUse |  | -0.012 |  | 0.012 |  | -0.054 |  | -1.043 |  | 0.297 |  |
|  |  | useEmotionalSupport |  | -0.005 |  | 0.014 |  | -0.018 |  | -0.324 |  | 0.746 |  |
|  |  | useInstrumentalSupport |  | 0.006 |  | 0.014 |  | 0.025 |  | 0.432 |  | 0.666 |  |
|  |  | behavioralDisengagement |  | -0.019 |  | 0.015 |  | -0.075 |  | -1.272 |  | 0.204 |  |
|  |  | venting |  | 0.009 |  | 0.014 |  | 0.033 |  | 0.615 |  | 0.539 |  |
|  |  | positiveReframing |  | -0.020 |  | 0.014 |  | -0.075 |  | -1.437 |  | 0.151 |  |
|  |  | planning |  | -0.021 |  | 0.018 |  | -0.078 |  | -1.201 |  | 0.230 |  |
|  |  | humor |  | -0.007 |  | 0.011 |  | -0.027 |  | -0.585 |  | 0.558 |  |
|  |  | acceptance |  | -2.395×10^-4^ |  | 0.013 |  | -8.880×10^-4^ |  | -0.019 |  | 0.985 |  |
|  |  | religion |  | -0.011 |  | 0.010 |  | -0.049 |  | -1.095 |  | 0.274 |  |
|  |  | selfBlame |  | 0.060 |  | 0.017 |  | 0.215 |  | 3.459 |  | < .001 |  |
|  | | | | | | | | | | | | | |

Quitting – Coping

## Linear Regression

| **Model Summary - quitting** | | | | | | | | | |
| --- | --- | --- | --- | --- | --- | --- | --- | --- | --- |
| **Model** | | **R** | | **R²** | | **Adjusted R²** | | **RMSE** | |
| H₀ |  | 0.000 |  | 0.000 |  | 0.000 |  | 0.420 |  |
| H₁ |  | 0.159 |  | 0.025 |  | 0.008 |  | 0.418 |  |
|  | | | | | | | | | |

| **ANOVA** | | | | | | | | | | | | | |
| --- | --- | --- | --- | --- | --- | --- | --- | --- | --- | --- | --- | --- | --- |
| **Model** | |  | | **Sum of Squares** | | **df** | | **Mean Square** | | **F** | | **p** | |
| H₁ |  | Regression |  | 3.616 |  | 14 |  | 0.258 |  | 1.479 |  | 0.113 |  |
|  |  | Residual |  | 139.235 |  | 797 |  | 0.175 |  |  |  |  |  |
|  |  | Total |  | 142.851 |  | 811 |  |  |  |  |  |  |  |
|  | | | | | | | | | | | | | |
| Note.  The intercept model is omitted, as no meaningful information can be shown. | | | | | | | | | | | | | |

| **Coefficients** | | | | | | | | | | | | | |
| --- | --- | --- | --- | --- | --- | --- | --- | --- | --- | --- | --- | --- | --- |
| **Model** | |  | | **Unstandardized** | | **Standard Error** | | **Standardized** | | **t** | | **p** | |
| H₀ |  | (Intercept) |  | 0.228 |  | 0.015 |  |  |  | 15.469 |  | < .001 |  |
| H₁ |  | (Intercept) |  | 0.071 |  | 0.064 |  |  |  | 1.111 |  | 0.267 |  |
|  |  | selfDistraction |  | 0.006 |  | 0.012 |  | 0.023 |  | 0.497 |  | 0.620 |  |
|  |  | activeCoping |  | -0.003 |  | 0.013 |  | -0.012 |  | -0.240 |  | 0.810 |  |
|  |  | Denial |  | -0.015 |  | 0.012 |  | -0.068 |  | -1.269 |  | 0.205 |  |
|  |  | substanceUse |  | 0.003 |  | 0.010 |  | 0.017 |  | 0.331 |  | 0.741 |  |
|  |  | useEmotionalSupport |  | -0.011 |  | 0.013 |  | -0.047 |  | -0.883 |  | 0.377 |  |
|  |  | useInstrumentalSupport |  | -0.012 |  | 0.013 |  | -0.052 |  | -0.923 |  | 0.356 |  |
|  |  | behavioralDisengagement |  | 0.018 |  | 0.013 |  | 0.081 |  | 1.398 |  | 0.163 |  |
|  |  | venting |  | 0.015 |  | 0.012 |  | 0.063 |  | 1.223 |  | 0.222 |  |
|  |  | positiveReframing |  | -0.010 |  | 0.013 |  | -0.041 |  | -0.801 |  | 0.423 |  |
|  |  | planning |  | 0.022 |  | 0.016 |  | 0.088 |  | 1.367 |  | 0.172 |  |
|  |  | humor |  | 0.011 |  | 0.010 |  | 0.048 |  | 1.036 |  | 0.301 |  |
|  |  | acceptance |  | -0.004 |  | 0.011 |  | -0.017 |  | -0.369 |  | 0.713 |  |
|  |  | religion |  | 0.008 |  | 0.009 |  | 0.038 |  | 0.876 |  | 0.381 |  |
|  |  | selfBlame |  | 0.006 |  | 0.015 |  | 0.022 |  | 0.360 |  | 0.719 |  |
|  | | | | | | | | | | | | | |

Quitting - IMAGE-BASED METHOD + Coping

## Linear Regression

| **Model Summary - quitting** | | | | | | | | | |
| --- | --- | --- | --- | --- | --- | --- | --- | --- | --- |
| **Model** | | **R** | | **R²** | | **Adjusted R²** | | **RMSE** | |
| H₀ |  | 0.000 |  | 0.000 |  | 0.000 |  | 0.420 |  |
| H₁ |  | 0.387 |  | 0.150 |  | 0.049 |  | 0.409 |  |
|  | | | | | | | | | |

| **ANOVA** | | | | | | | | | | | | | |
| --- | --- | --- | --- | --- | --- | --- | --- | --- | --- | --- | --- | --- | --- |
| **Model** | |  | | **Sum of Squares** | | **df** | | **Mean Square** | | **F** | | **p** | |
| H₁ |  | Regression |  | 21.391 |  | 86 |  | 0.249 |  | 1.485 |  | 0.004 |  |
|  |  | Residual |  | 121.460 |  | 725 |  | 0.168 |  |  |  |  |  |
|  |  | Total |  | 142.851 |  | 811 |  |  |  |  |  |  |  |
|  | | | | | | | | | | | | | |
| Note.  The intercept model is omitted, as no meaningful information can be shown. | | | | | | | | | | | | | |

| **Coefficients** | | | | | | | | | | | | | |
| --- | --- | --- | --- | --- | --- | --- | --- | --- | --- | --- | --- | --- | --- |
| **Model** | |  | | **Unstandardized** | | **Standard Error** | | **Standardized** | | **t** | | **p** | |
| H₀ |  | (Intercept) |  | 0.228 |  | 0.015 |  |  |  | 15.469 |  | < .001 |  |
| H₁ |  | (Intercept) |  | 0.047 |  | 0.067 |  |  |  | 0.706 |  | 0.480 |  |
|  |  | A1Px |  | 1.252×10^-4^ |  | 5.710×10^-5^ |  | 0.104 |  | 2.193 |  | 0.029 |  |
|  |  | A1Py |  | 3.424×10^-5^ |  | 5.704×10^-5^ |  | 0.027 |  | 0.600 |  | 0.548 |  |
|  |  | A1Pz |  | -7.403×10^-5^ |  | 5.624×10^-5^ |  | -0.058 |  | -1.316 |  | 0.189 |  |
|  |  | A2Px |  | -1.524×10^-6^ |  | 6.842×10^-5^ |  | -0.001 |  | -0.022 |  | 0.982 |  |
|  |  | A2Py |  | -1.268×10^-4^ |  | 7.228×10^-5^ |  | -0.084 |  | -1.754 |  | 0.080 |  |
|  |  | A2Pz |  | -1.670×10^-5^ |  | 5.609×10^-5^ |  | -0.013 |  | -0.298 |  | 0.766 |  |
|  |  | A3Px |  | 3.927×10^-5^ |  | 5.236×10^-5^ |  | 0.035 |  | 0.750 |  | 0.453 |  |
|  |  | A3Py |  | 2.631×10^-5^ |  | 5.247×10^-5^ |  | 0.023 |  | 0.501 |  | 0.616 |  |
|  |  | A3Pz |  | 2.228×10^-5^ |  | 5.605×10^-5^ |  | 0.018 |  | 0.397 |  | 0.691 |  |
|  |  | B1Px |  | -9.546×10^-6^ |  | 6.441×10^-5^ |  | -0.007 |  | -0.148 |  | 0.882 |  |
|  |  | B1Py |  | 2.536×10^-5^ |  | 5.971×10^-5^ |  | 0.020 |  | 0.425 |  | 0.671 |  |
|  |  | B1Pz |  | -3.905×10^-5^ |  | 5.679×10^-5^ |  | -0.032 |  | -0.688 |  | 0.492 |  |
|  |  | B2Px |  | -6.851×10^-5^ |  | 6.025×10^-5^ |  | -0.052 |  | -1.137 |  | 0.256 |  |
|  |  | B2Py |  | -6.249×10^-5^ |  | 5.929×10^-5^ |  | -0.047 |  | -1.054 |  | 0.292 |  |
|  |  | B2Pz |  | -1.293×10^-4^ |  | 5.919×10^-5^ |  | -0.101 |  | -2.185 |  | 0.029 |  |
|  |  | B3Px |  | 1.956×10^-5^ |  | 5.376×10^-5^ |  | 0.017 |  | 0.364 |  | 0.716 |  |
|  |  | B3Py |  | 6.095×10^-5^ |  | 5.263×10^-5^ |  | 0.053 |  | 1.158 |  | 0.247 |  |
|  |  | B3Pz |  | -5.479×10^-5^ |  | 6.279×10^-5^ |  | -0.042 |  | -0.873 |  | 0.383 |  |
|  |  | C1Px |  | -6.466×10^-5^ |  | 5.084×10^-5^ |  | -0.058 |  | -1.272 |  | 0.204 |  |
|  |  | C1Py |  | -8.035×10^-5^ |  | 5.897×10^-5^ |  | -0.067 |  | -1.363 |  | 0.173 |  |
|  |  | C1Pz |  | -4.337×10^-5^ |  | 5.926×10^-5^ |  | -0.034 |  | -0.732 |  | 0.465 |  |
|  |  | C2Px |  | 1.111×10^-4^ |  | 6.176×10^-5^ |  | 0.088 |  | 1.798 |  | 0.073 |  |
|  |  | C2Py |  | 6.208×10^-5^ |  | 6.020×10^-5^ |  | 0.048 |  | 1.031 |  | 0.303 |  |
|  |  | C2Pz |  | -2.169×10^-5^ |  | 5.864×10^-5^ |  | -0.018 |  | -0.370 |  | 0.712 |  |
|  |  | C3Px |  | 6.778×10^-5^ |  | 5.421×10^-5^ |  | 0.059 |  | 1.250 |  | 0.212 |  |
|  |  | C3Py |  | 9.903×10^-6^ |  | 5.720×10^-5^ |  | 0.008 |  | 0.173 |  | 0.863 |  |
|  |  | C3Pz |  | 4.182×10^-5^ |  | 5.835×10^-5^ |  | 0.036 |  | 0.717 |  | 0.474 |  |
|  |  | D1Px |  | -1.493×10^-5^ |  | 5.688×10^-5^ |  | -0.013 |  | -0.262 |  | 0.793 |  |
|  |  | D1Py |  | -3.180×10^-5^ |  | 6.075×10^-5^ |  | -0.026 |  | -0.523 |  | 0.601 |  |
|  |  | D1Pz |  | -4.745×10^-5^ |  | 6.066×10^-5^ |  | -0.038 |  | -0.782 |  | 0.434 |  |
|  |  | D2Px |  | 6.673×10^-5^ |  | 6.199×10^-5^ |  | 0.049 |  | 1.076 |  | 0.282 |  |
|  |  | D2Py |  | -1.184×10^-5^ |  | 7.056×10^-5^ |  | -0.009 |  | -0.168 |  | 0.867 |  |
|  |  | D2Pz |  | 9.567×10^-5^ |  | 6.007×10^-5^ |  | 0.073 |  | 1.593 |  | 0.112 |  |
|  |  | D3Px |  | -2.449×10^-5^ |  | 6.467×10^-5^ |  | -0.019 |  | -0.379 |  | 0.705 |  |
|  |  | D3Py |  | -8.542×10^-6^ |  | 5.432×10^-5^ |  | -0.007 |  | -0.157 |  | 0.875 |  |
|  |  | D3Pz |  | -6.611×10^-5^ |  | 5.550×10^-5^ |  | -0.054 |  | -1.191 |  | 0.234 |  |
|  |  | A1Nx |  | 4.846×10^-5^ |  | 5.910×10^-5^ |  | 0.040 |  | 0.820 |  | 0.413 |  |
|  |  | A1Ny |  | 4.569×10^-5^ |  | 5.979×10^-5^ |  | 0.038 |  | 0.764 |  | 0.445 |  |
|  |  | A1Nz |  | -5.601×10^-5^ |  | 5.795×10^-5^ |  | -0.046 |  | -0.967 |  | 0.334 |  |
|  |  | A2Nx |  | 1.491×10^-4^ |  | 6.126×10^-5^ |  | 0.114 |  | 2.434 |  | 0.015 |  |
|  |  | A2Ny |  | 9.098×10^-6^ |  | 5.893×10^-5^ |  | 0.008 |  | 0.154 |  | 0.877 |  |
|  |  | A2Nz |  | -1.892×10^-5^ |  | 6.377×10^-5^ |  | -0.015 |  | -0.297 |  | 0.767 |  |
|  |  | A3Nx |  | 4.985×10^-5^ |  | 5.653×10^-5^ |  | 0.043 |  | 0.882 |  | 0.378 |  |
|  |  | A3Ny |  | -2.418×10^-5^ |  | 6.103×10^-5^ |  | -0.020 |  | -0.396 |  | 0.692 |  |
|  |  | A3Nz |  | 2.429×10^-5^ |  | 5.653×10^-5^ |  | 0.022 |  | 0.430 |  | 0.668 |  |
|  |  | B1Nx |  | 4.740×10^-5^ |  | 5.755×10^-5^ |  | 0.039 |  | 0.824 |  | 0.410 |  |
|  |  | B1Ny |  | 2.495×10^-6^ |  | 5.810×10^-5^ |  | 0.002 |  | 0.043 |  | 0.966 |  |
|  |  | B1Nz |  | 2.381×10^-5^ |  | 5.655×10^-5^ |  | 0.020 |  | 0.421 |  | 0.674 |  |
|  |  | B2Nx |  | -6.429×10^-5^ |  | 5.195×10^-5^ |  | -0.056 |  | -1.238 |  | 0.216 |  |
|  |  | B2Ny |  | -5.565×10^-5^ |  | 6.290×10^-5^ |  | -0.044 |  | -0.885 |  | 0.377 |  |
|  |  | B2Nz |  | -5.861×10^-5^ |  | 5.472×10^-5^ |  | -0.051 |  | -1.071 |  | 0.284 |  |
|  |  | B3Nx |  | -3.436×10^-5^ |  | 6.256×10^-5^ |  | -0.028 |  | -0.549 |  | 0.583 |  |
|  |  | B3Ny |  | -8.927×10^-5^ |  | 6.171×10^-5^ |  | -0.067 |  | -1.447 |  | 0.148 |  |
|  |  | B3Nz |  | -9.318×10^-6^ |  | 6.376×10^-5^ |  | -0.007 |  | -0.146 |  | 0.884 |  |
|  |  | C1Nx |  | -1.770×10^-4^ |  | 6.687×10^-5^ |  | -0.138 |  | -2.647 |  | 0.008 |  |
|  |  | C1Ny |  | 5.571×10^-5^ |  | 6.088×10^-5^ |  | 0.042 |  | 0.915 |  | 0.360 |  |
|  |  | C1Nz |  | 5.236×10^-5^ |  | 6.945×10^-5^ |  | 0.036 |  | 0.754 |  | 0.451 |  |
|  |  | C2Nx |  | 4.703×10^-5^ |  | 5.588×10^-5^ |  | 0.041 |  | 0.842 |  | 0.400 |  |
|  |  | C2Ny |  | 8.734×10^-5^ |  | 5.483×10^-5^ |  | 0.076 |  | 1.593 |  | 0.112 |  |
|  |  | C2Nz |  | 2.679×10^-5^ |  | 5.671×10^-5^ |  | 0.022 |  | 0.472 |  | 0.637 |  |
|  |  | C3Nx |  | 7.787×10^-5^ |  | 6.015×10^-5^ |  | 0.066 |  | 1.295 |  | 0.196 |  |
|  |  | C3Ny |  | -1.502×10^-5^ |  | 6.160×10^-5^ |  | -0.012 |  | -0.244 |  | 0.807 |  |
|  |  | C3Nz |  | -6.453×10^-5^ |  | 5.765×10^-5^ |  | -0.056 |  | -1.119 |  | 0.263 |  |
|  |  | D1Nx |  | -3.841×10^-5^ |  | 5.958×10^-5^ |  | -0.032 |  | -0.645 |  | 0.519 |  |
|  |  | D1Ny |  | -2.325×10^-5^ |  | 5.992×10^-5^ |  | -0.019 |  | -0.388 |  | 0.698 |  |
|  |  | D1Nz |  | 1.312×10^-4^ |  | 6.071×10^-5^ |  | 0.110 |  | 2.161 |  | 0.031 |  |
|  |  | D2Nx |  | -1.697×10^-5^ |  | 5.419×10^-5^ |  | -0.015 |  | -0.313 |  | 0.754 |  |
|  |  | D2Ny |  | 1.751×10^-4^ |  | 5.727×10^-5^ |  | 0.146 |  | 3.058 |  | 0.002 |  |
|  |  | D2Nz |  | 2.362×10^-5^ |  | 6.155×10^-5^ |  | 0.019 |  | 0.384 |  | 0.701 |  |
|  |  | D3Nx |  | -2.734×10^-5^ |  | 5.795×10^-5^ |  | -0.023 |  | -0.472 |  | 0.637 |  |
|  |  | D3Ny |  | -1.798×10^-4^ |  | 6.113×10^-5^ |  | -0.137 |  | -2.942 |  | 0.003 |  |
|  |  | D3Nz |  | 9.365×10^-5^ |  | 5.827×10^-5^ |  | 0.078 |  | 1.607 |  | 0.108 |  |
|  |  | selfDistraction |  | 0.004 |  | 0.012 |  | 0.016 |  | 0.343 |  | 0.732 |  |
|  |  | activeCoping |  | -0.005 |  | 0.013 |  | -0.019 |  | -0.373 |  | 0.709 |  |
|  |  | Denial |  | -0.019 |  | 0.012 |  | -0.087 |  | -1.538 |  | 0.125 |  |
|  |  | substanceUse |  | 0.013 |  | 0.011 |  | 0.061 |  | 1.159 |  | 0.247 |  |
|  |  | useEmotionalSupport |  | -0.009 |  | 0.013 |  | -0.037 |  | -0.673 |  | 0.501 |  |
|  |  | useInstrumentalSupport |  | -0.008 |  | 0.013 |  | -0.036 |  | -0.628 |  | 0.530 |  |
|  |  | behavioralDisengagement |  | 0.016 |  | 0.013 |  | 0.070 |  | 1.175 |  | 0.240 |  |
|  |  | venting |  | 0.017 |  | 0.013 |  | 0.071 |  | 1.316 |  | 0.189 |  |
|  |  | positiveReframing |  | -0.013 |  | 0.013 |  | -0.054 |  | -1.022 |  | 0.307 |  |
|  |  | planning |  | 0.029 |  | 0.016 |  | 0.120 |  | 1.824 |  | 0.069 |  |
|  |  | humor |  | 0.007 |  | 0.010 |  | 0.031 |  | 0.650 |  | 0.516 |  |
|  |  | acceptance |  | -0.005 |  | 0.012 |  | -0.020 |  | -0.408 |  | 0.683 |  |
|  |  | religion |  | 0.007 |  | 0.009 |  | 0.034 |  | 0.762 |  | 0.446 |  |
|  |  | selfBlame |  | -0.001 |  | 0.016 |  | -0.005 |  | -0.073 |  | 0.942 |  |
|  | | | | | | | | | | | | | |

Burnout - Rokeach

## Linear Regression

| **Model Summary - burnout** | | | | | | | | | |
| --- | --- | --- | --- | --- | --- | --- | --- | --- | --- |
| **Model** | | **R** | | **R²** | | **Adjusted R²** | | **RMSE** | |
| H₀ |  | 0.000 |  | 0.000 |  | 0.000 |  | 0.465 |  |
| H₁ |  | 0.165 |  | 0.027 |  | 0.006 |  | 0.464 |  |
|  | | | | | | | | | |

| **ANOVA** | | | | | | | | | | | | | |
| --- | --- | --- | --- | --- | --- | --- | --- | --- | --- | --- | --- | --- | --- |
| **Model** | |  | | **Sum of Squares** | | **df** | | **Mean Square** | | **F** | | **p** | |
| H₁ |  | Regression |  | 4.659 |  | 17 |  | 0.274 |  | 1.274 |  | 0.202 |  |
|  |  | Residual |  | 165.659 |  | 770 |  | 0.215 |  |  |  |  |  |
|  |  | Total |  | 170.319 |  | 787 |  |  |  |  |  |  |  |
|  | | | | | | | | | | | | | |
| Note.  The intercept model is omitted, as no meaningful information can be shown. | | | | | | | | | | | | | |

| **Coefficients** | | | | | | | | | | | | | |
| --- | --- | --- | --- | --- | --- | --- | --- | --- | --- | --- | --- | --- | --- |
| **Model** | |  | | **Unstandardized** | | **Standard Error** | | **Standardized** | | **t** | | **p** | |
| H₀ |  | (Intercept) |  | 0.316 |  | 0.017 |  |  |  | 19.067 |  | < .001 |  |
| H₁ |  | (Intercept) |  | 0.617 |  | 0.534 |  |  |  | 1.154 |  | 0.249 |  |
|  |  | Q13_aFriendship |  | -0.012 |  | 0.110 |  | -0.005 |  | -0.112 |  | 0.911 |  |
|  |  | Q13_bLove |  | -0.020 |  | 0.105 |  | -0.009 |  | -0.193 |  | 0.847 |  |
|  |  | Q13_cSelfR |  | -0.109 |  | 0.123 |  | -0.036 |  | -0.885 |  | 0.376 |  |
|  |  | Q13_dHappiness |  | -0.023 |  | 0.140 |  | -0.007 |  | -0.162 |  | 0.872 |  |
|  |  | Q13_eInnerH |  | 0.021 |  | 0.117 |  | 0.008 |  | 0.179 |  | 0.858 |  |
|  |  | Q13_fEquality |  | -0.111 |  | 0.110 |  | -0.045 |  | -1.017 |  | 0.310 |  |
|  |  | Q13_gFreedom |  | 0.023 |  | 0.110 |  | 0.009 |  | 0.206 |  | 0.836 |  |
|  |  | Q13_hPleasure |  | 0.079 |  | 0.122 |  | 0.030 |  | 0.647 |  | 0.518 |  |
|  |  | Q13_iRecog |  | -0.308 |  | 0.117 |  | -0.134 |  | -2.631 |  | 0.009 |  |
|  |  | Q13_jWisdom |  | -0.041 |  | 0.111 |  | -0.016 |  | -0.370 |  | 0.711 |  |
|  |  | Q13_kSalvation |  | -0.106 |  | 0.095 |  | -0.051 |  | -1.112 |  | 0.266 |  |
|  |  | Q13_lFamSec |  | -0.043 |  | 0.103 |  | -0.024 |  | -0.415 |  | 0.678 |  |
|  |  | Q13_mNatSec |  | -0.029 |  | 0.112 |  | -0.011 |  | -0.259 |  | 0.796 |  |
|  |  | Q13_nAccomp |  | 0.034 |  | 0.113 |  | 0.015 |  | 0.302 |  | 0.763 |  |
|  |  | Q13_oBeauty |  | 0.123 |  | 0.135 |  | 0.038 |  | 0.910 |  | 0.363 |  |
|  |  | Q13_pPeace |  | -0.011 |  | 0.106 |  | -0.005 |  | -0.102 |  | 0.919 |  |
|  |  | Q13_rExcite |  | -0.059 |  | 0.115 |  | -0.029 |  | -0.511 |  | 0.610 |  |
|  | | | | | | | | | | | | | |

Burnout IMAGE-BASED METHOD + Rokeach

## Linear Regression

| **Model Summary - burnout** | | | | | | | | | |
| --- | --- | --- | --- | --- | --- | --- | --- | --- | --- |
| **Model** | | **R** | | **R²** | | **Adjusted R²** | | **RMSE** | |
| H₀ |  | 0.000 |  | 0.000 |  | 0.000 |  | 0.465 |  |
| H₁ |  | 0.388 |  | 0.150 |  | 0.042 |  | 0.455 |  |
|  | | | | | | | | | |

| **ANOVA** | | | | | | | | | | | | | |
| --- | --- | --- | --- | --- | --- | --- | --- | --- | --- | --- | --- | --- | --- |
| **Model** | |  | | **Sum of Squares** | | **df** | | **Mean Square** | | **F** | | **p** | |
| H₁ |  | Regression |  | 25.596 |  | 89 |  | 0.288 |  | 1.387 |  | 0.015 |  |
|  |  | Residual |  | 144.723 |  | 698 |  | 0.207 |  |  |  |  |  |
|  |  | Total |  | 170.319 |  | 787 |  |  |  |  |  |  |  |
|  | | | | | | | | | | | | | |
| Note.  The intercept model is omitted, as no meaningful information can be shown. | | | | | | | | | | | | | |

| **Coefficients** | | | | | | | | | | | | | |
| --- | --- | --- | --- | --- | --- | --- | --- | --- | --- | --- | --- | --- | --- |
| **Model** | |  | | **Unstandardized** | | **Standard Error** | | **Standardized** | | **t** | | **p** | |
| H₀ |  | (Intercept) |  | 0.316 |  | 0.017 |  |  |  | 19.067 |  | < .001 |  |
| H₁ |  | (Intercept) |  | 0.355 |  | 0.563 |  |  |  | 0.630 |  | 0.529 |  |
|  |  | A1Px |  | 1.067×10^-4^ |  | 6.409×10^-5^ |  | 0.080 |  | 1.664 |  | 0.097 |  |
|  |  | A1Py |  | 5.423×10^-5^ |  | 6.461×10^-5^ |  | 0.039 |  | 0.839 |  | 0.402 |  |
|  |  | A1Pz |  | 1.082×10^-4^ |  | 6.323×10^-5^ |  | 0.077 |  | 1.712 |  | 0.087 |  |
|  |  | A2Px |  | -1.314×10^-4^ |  | 7.636×10^-5^ |  | -0.086 |  | -1.720 |  | 0.086 |  |
|  |  | A2Py |  | -2.925×10^-5^ |  | 8.374×10^-5^ |  | -0.017 |  | -0.349 |  | 0.727 |  |
|  |  | A2Pz |  | 1.734×10^-5^ |  | 6.365×10^-5^ |  | 0.012 |  | 0.272 |  | 0.785 |  |
|  |  | A3Px |  | -3.792×10^-5^ |  | 5.918×10^-5^ |  | -0.030 |  | -0.641 |  | 0.522 |  |
|  |  | A3Py |  | 5.302×10^-5^ |  | 6.010×10^-5^ |  | 0.043 |  | 0.882 |  | 0.378 |  |
|  |  | A3Pz |  | -4.667×10^-6^ |  | 6.324×10^-5^ |  | -0.003 |  | -0.074 |  | 0.941 |  |
|  |  | B1Px |  | 1.105×10^-4^ |  | 7.221×10^-5^ |  | 0.071 |  | 1.531 |  | 0.126 |  |
|  |  | B1Py |  | 6.667×10^-5^ |  | 6.721×10^-5^ |  | 0.047 |  | 0.992 |  | 0.322 |  |
|  |  | B1Pz |  | -3.015×10^-5^ |  | 6.521×10^-5^ |  | -0.022 |  | -0.462 |  | 0.644 |  |
|  |  | B2Px |  | -1.009×10^-6^ |  | 6.809×10^-5^ |  | -6.950×10^-4^ |  | -0.015 |  | 0.988 |  |
|  |  | B2Py |  | -2.368×10^-5^ |  | 6.610×10^-5^ |  | -0.016 |  | -0.358 |  | 0.720 |  |
|  |  | B2Pz |  | 1.157×10^-5^ |  | 6.674×10^-5^ |  | 0.008 |  | 0.173 |  | 0.862 |  |
|  |  | B3Px |  | -4.183×10^-5^ |  | 6.098×10^-5^ |  | -0.033 |  | -0.686 |  | 0.493 |  |
|  |  | B3Py |  | -3.699×10^-5^ |  | 5.975×10^-5^ |  | -0.029 |  | -0.619 |  | 0.536 |  |
|  |  | B3Pz |  | -1.022×10^-4^ |  | 7.059×10^-5^ |  | -0.072 |  | -1.448 |  | 0.148 |  |
|  |  | C1Px |  | 1.895×10^-5^ |  | 5.741×10^-5^ |  | 0.015 |  | 0.330 |  | 0.741 |  |
|  |  | C1Py |  | -8.538×10^-5^ |  | 6.652×10^-5^ |  | -0.064 |  | -1.284 |  | 0.200 |  |
|  |  | C1Pz |  | 8.269×10^-5^ |  | 6.709×10^-5^ |  | 0.059 |  | 1.232 |  | 0.218 |  |
|  |  | C2Px |  | 1.867×10^-5^ |  | 6.969×10^-5^ |  | 0.013 |  | 0.268 |  | 0.789 |  |
|  |  | C2Py |  | -6.444×10^-5^ |  | 6.813×10^-5^ |  | -0.045 |  | -0.946 |  | 0.345 |  |
|  |  | C2Pz |  | -4.222×10^-5^ |  | 6.687×10^-5^ |  | -0.031 |  | -0.631 |  | 0.528 |  |
|  |  | C3Px |  | 4.175×10^-5^ |  | 6.112×10^-5^ |  | 0.032 |  | 0.683 |  | 0.495 |  |
|  |  | C3Py |  | -2.059×10^-6^ |  | 6.476×10^-5^ |  | -0.002 |  | -0.032 |  | 0.975 |  |
|  |  | C3Pz |  | 2.300×10^-6^ |  | 6.498×10^-5^ |  | 0.002 |  | 0.035 |  | 0.972 |  |
|  |  | D1Px |  | 4.450×10^-5^ |  | 6.402×10^-5^ |  | 0.034 |  | 0.695 |  | 0.487 |  |
|  |  | D1Py |  | 9.142×10^-6^ |  | 6.877×10^-5^ |  | 0.007 |  | 0.133 |  | 0.894 |  |
|  |  | D1Pz |  | 3.581×10^-5^ |  | 6.789×10^-5^ |  | 0.026 |  | 0.527 |  | 0.598 |  |
|  |  | D2Px |  | -8.368×10^-5^ |  | 7.160×10^-5^ |  | -0.055 |  | -1.169 |  | 0.243 |  |
|  |  | D2Py |  | -2.543×10^-5^ |  | 7.924×10^-5^ |  | -0.017 |  | -0.321 |  | 0.748 |  |
|  |  | D2Pz |  | -1.926×10^-5^ |  | 6.749×10^-5^ |  | -0.013 |  | -0.285 |  | 0.775 |  |
|  |  | D3Px |  | 1.491×10^-5^ |  | 7.192×10^-5^ |  | 0.011 |  | 0.207 |  | 0.836 |  |
|  |  | D3Py |  | -9.367×10^-5^ |  | 6.120×10^-5^ |  | -0.068 |  | -1.531 |  | 0.126 |  |
|  |  | D3Pz |  | -3.842×10^-5^ |  | 6.212×10^-5^ |  | -0.028 |  | -0.619 |  | 0.536 |  |
|  |  | A1Nx |  | 1.366×10^-5^ |  | 6.699×10^-5^ |  | 0.010 |  | 0.204 |  | 0.838 |  |
|  |  | A1Ny |  | -2.858×10^-5^ |  | 6.721×10^-5^ |  | -0.022 |  | -0.425 |  | 0.671 |  |
|  |  | A1Nz |  | 1.495×10^-4^ |  | 6.679×10^-5^ |  | 0.111 |  | 2.238 |  | 0.026 |  |
|  |  | A2Nx |  | -1.813×10^-4^ |  | 7.135×10^-5^ |  | -0.123 |  | -2.542 |  | 0.011 |  |
|  |  | A2Ny |  | -1.058×10^-4^ |  | 6.687×10^-5^ |  | -0.081 |  | -1.582 |  | 0.114 |  |
|  |  | A2Nz |  | 1.597×10^-5^ |  | 7.130×10^-5^ |  | 0.011 |  | 0.224 |  | 0.823 |  |
|  |  | A3Nx |  | -8.056×10^-5^ |  | 6.300×10^-5^ |  | -0.062 |  | -1.279 |  | 0.201 |  |
|  |  | A3Ny |  | 2.575×10^-5^ |  | 6.945×10^-5^ |  | 0.019 |  | 0.371 |  | 0.711 |  |
|  |  | A3Nz |  | 6.844×10^-5^ |  | 6.404×10^-5^ |  | 0.055 |  | 1.069 |  | 0.286 |  |
|  |  | B1Nx |  | 3.318×10^-5^ |  | 6.615×10^-5^ |  | 0.025 |  | 0.502 |  | 0.616 |  |
|  |  | B1Ny |  | 8.360×10^-5^ |  | 6.617×10^-5^ |  | 0.065 |  | 1.264 |  | 0.207 |  |
|  |  | B1Nz |  | 2.383×10^-4^ |  | 6.389×10^-5^ |  | 0.184 |  | 3.730 |  | < .001 |  |
|  |  | B2Nx |  | -3.326×10^-5^ |  | 5.827×10^-5^ |  | -0.026 |  | -0.571 |  | 0.568 |  |
|  |  | B2Ny |  | -5.316×10^-5^ |  | 7.214×10^-5^ |  | -0.038 |  | -0.737 |  | 0.461 |  |
|  |  | B2Nz |  | 1.100×10^-4^ |  | 6.187×10^-5^ |  | 0.087 |  | 1.778 |  | 0.076 |  |
|  |  | B3Nx |  | 2.765×10^-5^ |  | 7.115×10^-5^ |  | 0.020 |  | 0.389 |  | 0.698 |  |
|  |  | B3Ny |  | -2.409×10^-5^ |  | 6.918×10^-5^ |  | -0.016 |  | -0.348 |  | 0.728 |  |
|  |  | B3Nz |  | -2.155×10^-5^ |  | 7.182×10^-5^ |  | -0.015 |  | -0.300 |  | 0.764 |  |
|  |  | C1Nx |  | 4.837×10^-5^ |  | 7.503×10^-5^ |  | 0.034 |  | 0.645 |  | 0.519 |  |
|  |  | C1Ny |  | 1.311×10^-4^ |  | 6.883×10^-5^ |  | 0.088 |  | 1.904 |  | 0.057 |  |
|  |  | C1Nz |  | 3.394×10^-5^ |  | 7.821×10^-5^ |  | 0.021 |  | 0.434 |  | 0.664 |  |
|  |  | C2Nx |  | 6.434×10^-5^ |  | 6.338×10^-5^ |  | 0.050 |  | 1.015 |  | 0.310 |  |
|  |  | C2Ny |  | 3.838×10^-5^ |  | 6.281×10^-5^ |  | 0.030 |  | 0.611 |  | 0.541 |  |
|  |  | C2Nz |  | -2.329×10^-5^ |  | 6.415×10^-5^ |  | -0.018 |  | -0.363 |  | 0.717 |  |
|  |  | C3Nx |  | 6.979×10^-5^ |  | 6.899×10^-5^ |  | 0.054 |  | 1.012 |  | 0.312 |  |
|  |  | C3Ny |  | -5.529×10^-5^ |  | 7.164×10^-5^ |  | -0.041 |  | -0.772 |  | 0.441 |  |
|  |  | C3Nz |  | -9.482×10^-5^ |  | 6.537×10^-5^ |  | -0.074 |  | -1.451 |  | 0.147 |  |
|  |  | D1Nx |  | 1.323×10^-5^ |  | 6.799×10^-5^ |  | 0.010 |  | 0.195 |  | 0.846 |  |
|  |  | D1Ny |  | 1.005×10^-4^ |  | 6.689×10^-5^ |  | 0.075 |  | 1.503 |  | 0.133 |  |
|  |  | D1Nz |  | -5.290×10^-5^ |  | 6.766×10^-5^ |  | -0.040 |  | -0.782 |  | 0.435 |  |
|  |  | D2Nx |  | 1.485×10^-5^ |  | 6.161×10^-5^ |  | 0.011 |  | 0.241 |  | 0.810 |  |
|  |  | D2Ny |  | -1.038×10^-4^ |  | 6.539×10^-5^ |  | -0.077 |  | -1.587 |  | 0.113 |  |
|  |  | D2Nz |  | -1.167×10^-4^ |  | 6.981×10^-5^ |  | -0.087 |  | -1.671 |  | 0.095 |  |
|  |  | D3Nx |  | -7.661×10^-5^ |  | 6.560×10^-5^ |  | -0.058 |  | -1.168 |  | 0.243 |  |
|  |  | D3Ny |  | 5.788×10^-5^ |  | 6.976×10^-5^ |  | 0.040 |  | 0.830 |  | 0.407 |  |
|  |  | D3Nz |  | -1.091×10^-4^ |  | 6.573×10^-5^ |  | -0.083 |  | -1.659 |  | 0.097 |  |
|  |  | Q13_aFriendship |  | -0.030 |  | 0.113 |  | -0.013 |  | -0.264 |  | 0.792 |  |
|  |  | Q13_bLove |  | 0.010 |  | 0.109 |  | 0.005 |  | 0.092 |  | 0.927 |  |
|  |  | Q13_cSelfR |  | -0.068 |  | 0.126 |  | -0.023 |  | -0.538 |  | 0.591 |  |
|  |  | Q13_dHappiness |  | 0.025 |  | 0.147 |  | 0.008 |  | 0.168 |  | 0.866 |  |
|  |  | Q13_eInnerH |  | 0.072 |  | 0.121 |  | 0.026 |  | 0.596 |  | 0.552 |  |
|  |  | Q13_fEquality |  | -0.104 |  | 0.113 |  | -0.042 |  | -0.917 |  | 0.359 |  |
|  |  | Q13_gFreedom |  | 0.053 |  | 0.115 |  | 0.020 |  | 0.464 |  | 0.643 |  |
|  |  | Q13_hPleasure |  | 0.069 |  | 0.126 |  | 0.026 |  | 0.550 |  | 0.582 |  |
|  |  | Q13_iRecog |  | -0.203 |  | 0.122 |  | -0.089 |  | -1.674 |  | 0.095 |  |
|  |  | Q13_jWisdom |  | -0.035 |  | 0.115 |  | -0.014 |  | -0.304 |  | 0.761 |  |
|  |  | Q13_kSalvation |  | -0.062 |  | 0.100 |  | -0.030 |  | -0.615 |  | 0.539 |  |
|  |  | Q13_lFamSec |  | -0.063 |  | 0.106 |  | -0.035 |  | -0.591 |  | 0.555 |  |
|  |  | Q13_mNatSec |  | 0.036 |  | 0.116 |  | 0.013 |  | 0.308 |  | 0.759 |  |
|  |  | Q13_nAccomp |  | 0.081 |  | 0.116 |  | 0.034 |  | 0.695 |  | 0.487 |  |
|  |  | Q13_oBeauty |  | 0.114 |  | 0.140 |  | 0.036 |  | 0.812 |  | 0.417 |  |
|  |  | Q13_pPeace |  | 0.019 |  | 0.109 |  | 0.009 |  | 0.173 |  | 0.863 |  |
|  |  | Q13_rExcite |  | -0.103 |  | 0.120 |  | -0.052 |  | -0.859 |  | 0.390 |  |
|  | | | | | | | | | | | | | |

Quitting -Rokeach

## Linear Regression

| **Model Summary - quitting** | | | | | | | | | |
| --- | --- | --- | --- | --- | --- | --- | --- | --- | --- |
| **Model** | | **R** | | **R²** | | **Adjusted R²** | | **RMSE** | |
| H₀ |  | 0.000 |  | 0.000 |  | 0.000 |  | 0.419 |  |
| H₁ |  | 0.157 |  | 0.025 |  | 0.003 |  | 0.419 |  |
|  | | | | | | | | | |

| **ANOVA** | | | | | | | | | | | | | |
| --- | --- | --- | --- | --- | --- | --- | --- | --- | --- | --- | --- | --- | --- |
| **Model** | |  | | **Sum of Squares** | | **df** | | **Mean Square** | | **F** | | **p** | |
| H₁ |  | Regression |  | 3.402 |  | 17 |  | 0.200 |  | 1.142 |  | 0.309 |  |
|  |  | Residual |  | 134.937 |  | 770 |  | 0.175 |  |  |  |  |  |
|  |  | Total |  | 138.339 |  | 787 |  |  |  |  |  |  |  |
|  | | | | | | | | | | | | | |
| Note.  The intercept model is omitted, as no meaningful information can be shown. | | | | | | | | | | | | | |

| **Coefficients** | | | | | | | | | | | | | |
| --- | --- | --- | --- | --- | --- | --- | --- | --- | --- | --- | --- | --- | --- |
| **Model** | |  | | **Unstandardized** | | **Standard Error** | | **Standardized** | | **t** | | **p** | |
| H₀ |  | (Intercept) |  | 0.227 |  | 0.015 |  |  |  | 15.209 |  | < .001 |  |
| H₁ |  | (Intercept) |  | -0.074 |  | 0.482 |  |  |  | -0.153 |  | 0.878 |  |
|  |  | Q13_aFriendship |  | -0.078 |  | 0.099 |  | -0.037 |  | -0.791 |  | 0.429 |  |
|  |  | Q13_bLove |  | 0.014 |  | 0.095 |  | 0.007 |  | 0.150 |  | 0.881 |  |
|  |  | Q13_cSelfR |  | 0.203 |  | 0.111 |  | 0.075 |  | 1.828 |  | 0.068 |  |
|  |  | Q13_dHappiness |  | -0.026 |  | 0.126 |  | -0.009 |  | -0.207 |  | 0.836 |  |
|  |  | Q13_eInnerH |  | -0.026 |  | 0.105 |  | -0.010 |  | -0.243 |  | 0.808 |  |
|  |  | Q13_fEquality |  | -0.110 |  | 0.099 |  | -0.049 |  | -1.116 |  | 0.265 |  |
|  |  | Q13_gFreedom |  | 0.083 |  | 0.099 |  | 0.035 |  | 0.832 |  | 0.406 |  |
|  |  | Q13_hPleasure |  | 0.203 |  | 0.110 |  | 0.084 |  | 1.846 |  | 0.065 |  |
|  |  | Q13_iRecog |  | 0.182 |  | 0.106 |  | 0.088 |  | 1.722 |  | 0.085 |  |
|  |  | Q13_jWisdom |  | 0.106 |  | 0.100 |  | 0.046 |  | 1.060 |  | 0.289 |  |
|  |  | Q13_kSalvation |  | 0.007 |  | 0.086 |  | 0.004 |  | 0.084 |  | 0.933 |  |
|  |  | Q13_lFamSec |  | 0.090 |  | 0.093 |  | 0.056 |  | 0.971 |  | 0.332 |  |
|  |  | Q13_mNatSec |  | -0.096 |  | 0.102 |  | -0.040 |  | -0.944 |  | 0.345 |  |
|  |  | Q13_nAccomp |  | -0.004 |  | 0.102 |  | -0.002 |  | -0.036 |  | 0.971 |  |
|  |  | Q13_oBeauty |  | -0.078 |  | 0.121 |  | -0.027 |  | -0.643 |  | 0.520 |  |
|  |  | Q13_pPeace |  | 0.099 |  | 0.096 |  | 0.053 |  | 1.030 |  | 0.303 |  |
|  |  | Q13_rExcite |  | 0.021 |  | 0.104 |  | 0.012 |  | 0.201 |  | 0.841 |  |
|  | | | | | | | | | | | | | |

Quitting – Rokeach + IMAGE-BASED METHOD

## Linear Regression

| **Model Summary - quitting** | | | | | | | | | |
| --- | --- | --- | --- | --- | --- | --- | --- | --- | --- |
| **Model** | | **R** | | **R²** | | **Adjusted R²** | | **RMSE** | |
| H₀ |  | 0.000 |  | 0.000 |  | 0.000 |  | 0.419 |  |
| H₁ |  | 0.382 |  | 0.146 |  | 0.037 |  | 0.411 |  |
|  | | | | | | | | | |

| **ANOVA** | | | | | | | | | | | | | |
| --- | --- | --- | --- | --- | --- | --- | --- | --- | --- | --- | --- | --- | --- |
| **Model** | |  | | **Sum of Squares** | | **df** | | **Mean Square** | | **F** | | **p** | |
| H₁ |  | Regression |  | 20.157 |  | 89 |  | 0.226 |  | 1.338 |  | 0.026 |  |
|  |  | Residual |  | 118.181 |  | 698 |  | 0.169 |  |  |  |  |  |
|  |  | Total |  | 138.339 |  | 787 |  |  |  |  |  |  |  |
|  | | | | | | | | | | | | | |
| Note.  The intercept model is omitted, as no meaningful information can be shown. | | | | | | | | | | | | | |

| **Coefficients** | | | | | | | | | | | | | |
| --- | --- | --- | --- | --- | --- | --- | --- | --- | --- | --- | --- | --- | --- |
| **Model** | |  | | **Unstandardized** | | **Standard Error** | | **Standardized** | | **t** | | **p** | |
| H₀ |  | (Intercept) |  | 0.227 |  | 0.015 |  |  |  | 15.209 |  | < .001 |  |
| H₁ |  | (Intercept) |  | -0.004 |  | 0.628 |  |  |  | -0.007 |  | 0.995 |  |
|  |  | A1Px |  | 1.210×10^-4^ |  | 5.791×10^-5^ |  | 0.101 |  | 2.090 |  | 0.037 |  |
|  |  | A1Py |  | 5.096×10^-5^ |  | 5.839×10^-5^ |  | 0.041 |  | 0.873 |  | 0.383 |  |
|  |  | A1Pz |  | -6.304×10^-5^ |  | 5.714×10^-5^ |  | -0.050 |  | -1.103 |  | 0.270 |  |
|  |  | A2Px |  | -1.784×10^-6^ |  | 6.900×10^-5^ |  | -0.001 |  | -0.026 |  | 0.979 |  |
|  |  | A2Py |  | -1.227×10^-4^ |  | 7.567×10^-5^ |  | -0.080 |  | -1.622 |  | 0.105 |  |
|  |  | A2Pz |  | 6.725×10^-7^ |  | 5.752×10^-5^ |  | 5.378×10^-4^ |  | 0.012 |  | 0.991 |  |
|  |  | A3Px |  | 3.649×10^-5^ |  | 5.348×10^-5^ |  | 0.032 |  | 0.682 |  | 0.495 |  |
|  |  | A3Py |  | 2.654×10^-5^ |  | 5.431×10^-5^ |  | 0.024 |  | 0.489 |  | 0.625 |  |
|  |  | A3Pz |  | 1.447×10^-5^ |  | 5.715×10^-5^ |  | 0.012 |  | 0.253 |  | 0.800 |  |
|  |  | B1Px |  | 3.195×10^-6^ |  | 6.525×10^-5^ |  | 0.002 |  | 0.049 |  | 0.961 |  |
|  |  | B1Py |  | 9.043×10^-6^ |  | 6.073×10^-5^ |  | 0.007 |  | 0.149 |  | 0.882 |  |
|  |  | B1Pz |  | -1.480×10^-5^ |  | 5.893×10^-5^ |  | -0.012 |  | -0.251 |  | 0.802 |  |
|  |  | B2Px |  | -7.017×10^-5^ |  | 6.153×10^-5^ |  | -0.054 |  | -1.140 |  | 0.254 |  |
|  |  | B2Py |  | -4.124×10^-5^ |  | 5.973×10^-5^ |  | -0.032 |  | -0.690 |  | 0.490 |  |
|  |  | B2Pz |  | -1.119×10^-4^ |  | 6.031×10^-5^ |  | -0.088 |  | -1.856 |  | 0.064 |  |
|  |  | B3Px |  | -4.613×10^-6^ |  | 5.511×10^-5^ |  | -0.004 |  | -0.084 |  | 0.933 |  |
|  |  | B3Py |  | 6.254×10^-5^ |  | 5.399×10^-5^ |  | 0.054 |  | 1.158 |  | 0.247 |  |
|  |  | B3Pz |  | -6.110×10^-5^ |  | 6.379×10^-5^ |  | -0.048 |  | -0.958 |  | 0.338 |  |
|  |  | C1Px |  | -6.816×10^-5^ |  | 5.188×10^-5^ |  | -0.062 |  | -1.314 |  | 0.189 |  |
|  |  | C1Py |  | -8.993×10^-5^ |  | 6.011×10^-5^ |  | -0.074 |  | -1.496 |  | 0.135 |  |
|  |  | C1Pz |  | -3.027×10^-5^ |  | 6.063×10^-5^ |  | -0.024 |  | -0.499 |  | 0.618 |  |
|  |  | C2Px |  | 1.141×10^-4^ |  | 6.297×10^-5^ |  | 0.091 |  | 1.811 |  | 0.071 |  |
|  |  | C2Py |  | 5.916×10^-5^ |  | 6.156×10^-5^ |  | 0.046 |  | 0.961 |  | 0.337 |  |
|  |  | C2Pz |  | -1.951×10^-5^ |  | 6.042×10^-5^ |  | -0.016 |  | -0.323 |  | 0.747 |  |
|  |  | C3Px |  | 7.080×10^-5^ |  | 5.523×10^-5^ |  | 0.061 |  | 1.282 |  | 0.200 |  |
|  |  | C3Py |  | 1.263×10^-5^ |  | 5.852×10^-5^ |  | 0.010 |  | 0.216 |  | 0.829 |  |
|  |  | C3Pz |  | 2.479×10^-5^ |  | 5.872×10^-5^ |  | 0.021 |  | 0.422 |  | 0.673 |  |
|  |  | D1Px |  | -7.220×10^-6^ |  | 5.785×10^-5^ |  | -0.006 |  | -0.125 |  | 0.901 |  |
|  |  | D1Py |  | -2.575×10^-5^ |  | 6.214×10^-5^ |  | -0.021 |  | -0.414 |  | 0.679 |  |
|  |  | D1Pz |  | -6.861×10^-5^ |  | 6.135×10^-5^ |  | -0.055 |  | -1.118 |  | 0.264 |  |
|  |  | D2Px |  | 5.225×10^-5^ |  | 6.471×10^-5^ |  | 0.038 |  | 0.807 |  | 0.420 |  |
|  |  | D2Py |  | -3.594×10^-5^ |  | 7.160×10^-5^ |  | -0.027 |  | -0.502 |  | 0.616 |  |
|  |  | D2Pz |  | 8.358×10^-5^ |  | 6.099×10^-5^ |  | 0.064 |  | 1.370 |  | 0.171 |  |
|  |  | D3Px |  | -3.993×10^-5^ |  | 6.499×10^-5^ |  | -0.032 |  | -0.614 |  | 0.539 |  |
|  |  | D3Py |  | -1.715×10^-5^ |  | 5.530×10^-5^ |  | -0.014 |  | -0.310 |  | 0.757 |  |
|  |  | D3Pz |  | -5.221×10^-5^ |  | 5.614×10^-5^ |  | -0.043 |  | -0.930 |  | 0.353 |  |
|  |  | A1Nx |  | 3.135×10^-5^ |  | 6.054×10^-5^ |  | 0.026 |  | 0.518 |  | 0.605 |  |
|  |  | A1Ny |  | 6.202×10^-5^ |  | 6.074×10^-5^ |  | 0.052 |  | 1.021 |  | 0.308 |  |
|  |  | A1Nz |  | -2.843×10^-5^ |  | 6.036×10^-5^ |  | -0.023 |  | -0.471 |  | 0.638 |  |
|  |  | A2Nx |  | 1.393×10^-4^ |  | 6.447×10^-5^ |  | 0.105 |  | 2.160 |  | 0.031 |  |
|  |  | A2Ny |  | 1.140×10^-5^ |  | 6.043×10^-5^ |  | 0.010 |  | 0.189 |  | 0.850 |  |
|  |  | A2Nz |  | 4.471×10^-6^ |  | 6.443×10^-5^ |  | 0.004 |  | 0.069 |  | 0.945 |  |
|  |  | A3Nx |  | 5.898×10^-5^ |  | 5.693×10^-5^ |  | 0.051 |  | 1.036 |  | 0.301 |  |
|  |  | A3Ny |  | -4.961×10^-5^ |  | 6.276×10^-5^ |  | -0.041 |  | -0.790 |  | 0.430 |  |
|  |  | A3Nz |  | 1.772×10^-5^ |  | 5.787×10^-5^ |  | 0.016 |  | 0.306 |  | 0.760 |  |
|  |  | B1Nx |  | 7.682×10^-5^ |  | 5.978×10^-5^ |  | 0.063 |  | 1.285 |  | 0.199 |  |
|  |  | B1Ny |  | 6.640×10^-6^ |  | 5.979×10^-5^ |  | 0.006 |  | 0.111 |  | 0.912 |  |
|  |  | B1Nz |  | 1.321×10^-5^ |  | 5.773×10^-5^ |  | 0.011 |  | 0.229 |  | 0.819 |  |
|  |  | B2Nx |  | -8.643×10^-5^ |  | 5.265×10^-5^ |  | -0.075 |  | -1.642 |  | 0.101 |  |
|  |  | B2Ny |  | -6.120×10^-5^ |  | 6.519×10^-5^ |  | -0.048 |  | -0.939 |  | 0.348 |  |
|  |  | B2Nz |  | -4.508×10^-5^ |  | 5.591×10^-5^ |  | -0.040 |  | -0.806 |  | 0.420 |  |
|  |  | B3Nx |  | -3.735×10^-5^ |  | 6.429×10^-5^ |  | -0.031 |  | -0.581 |  | 0.561 |  |
|  |  | B3Ny |  | -9.728×10^-5^ |  | 6.251×10^-5^ |  | -0.074 |  | -1.556 |  | 0.120 |  |
|  |  | B3Nz |  | -2.995×10^-5^ |  | 6.490×10^-5^ |  | -0.023 |  | -0.462 |  | 0.645 |  |
|  |  | C1Nx |  | -1.930×10^-4^ |  | 6.780×10^-5^ |  | -0.151 |  | -2.847 |  | 0.005 |  |
|  |  | C1Ny |  | 2.019×10^-5^ |  | 6.220×10^-5^ |  | 0.015 |  | 0.325 |  | 0.746 |  |
|  |  | C1Nz |  | 3.130×10^-5^ |  | 7.068×10^-5^ |  | 0.022 |  | 0.443 |  | 0.658 |  |
|  |  | C2Nx |  | 8.615×10^-5^ |  | 5.727×10^-5^ |  | 0.075 |  | 1.504 |  | 0.133 |  |
|  |  | C2Ny |  | 9.505×10^-5^ |  | 5.676×10^-5^ |  | 0.083 |  | 1.675 |  | 0.094 |  |
|  |  | C2Nz |  | 2.033×10^-5^ |  | 5.797×10^-5^ |  | 0.017 |  | 0.351 |  | 0.726 |  |
|  |  | C3Nx |  | 5.391×10^-5^ |  | 6.234×10^-5^ |  | 0.046 |  | 0.865 |  | 0.387 |  |
|  |  | C3Ny |  | -1.219×10^-5^ |  | 6.474×10^-5^ |  | -0.010 |  | -0.188 |  | 0.851 |  |
|  |  | C3Nz |  | -7.496×10^-5^ |  | 5.907×10^-5^ |  | -0.065 |  | -1.269 |  | 0.205 |  |
|  |  | D1Nx |  | -2.834×10^-5^ |  | 6.144×10^-5^ |  | -0.024 |  | -0.461 |  | 0.645 |  |
|  |  | D1Ny |  | -2.831×10^-5^ |  | 6.044×10^-5^ |  | -0.023 |  | -0.468 |  | 0.640 |  |
|  |  | D1Nz |  | 1.663×10^-4^ |  | 6.114×10^-5^ |  | 0.140 |  | 2.720 |  | 0.007 |  |
|  |  | D2Nx |  | -2.648×10^-5^ |  | 5.567×10^-5^ |  | -0.023 |  | -0.476 |  | 0.635 |  |
|  |  | D2Ny |  | 1.763×10^-4^ |  | 5.909×10^-5^ |  | 0.145 |  | 2.983 |  | 0.003 |  |
|  |  | D2Nz |  | 1.198×10^-6^ |  | 6.309×10^-5^ |  | 9.860×10^-4^ |  | 0.019 |  | 0.985 |  |
|  |  | D3Nx |  | -2.834×10^-5^ |  | 5.928×10^-5^ |  | -0.024 |  | -0.478 |  | 0.633 |  |
|  |  | D3Ny |  | -1.620×10^-4^ |  | 6.304×10^-5^ |  | -0.124 |  | -2.570 |  | 0.010 |  |
|  |  | D3Nz |  | 9.664×10^-5^ |  | 5.940×10^-5^ |  | 0.082 |  | 1.627 |  | 0.104 |  |
|  |  | Q13_aFriendship |  | -0.089 |  | 0.116 |  | -0.042 |  | -0.767 |  | 0.443 |  |
|  |  | Q13_bLove |  | 8.581×10^-4^ |  | 0.113 |  | 4.290×10^-4^ |  | 0.008 |  | 0.994 |  |
|  |  | Q13_cSelfR |  | 0.155 |  | 0.125 |  | 0.057 |  | 1.240 |  | 0.215 |  |
|  |  | Q13_dHappiness |  | -0.003 |  | 0.131 |  | -0.001 |  | -0.023 |  | 0.981 |  |
|  |  | Q13_eInnerH |  | -0.052 |  | 0.114 |  | -0.021 |  | -0.454 |  | 0.650 |  |
|  |  | Q13_fEquality |  | -0.112 |  | 0.115 |  | -0.050 |  | -0.976 |  | 0.329 |  |
|  |  | Q13_gFreedom |  | 0.080 |  | 0.117 |  | 0.034 |  | 0.679 |  | 0.497 |  |
|  |  | Q13_hPleasure |  | 0.204 |  | 0.120 |  | 0.085 |  | 1.704 |  | 0.089 |  |
|  |  | Q13_iRecog |  | 0.208 |  | 0.114 |  | 0.101 |  | 1.821 |  | 0.069 |  |
|  |  | Q13_jWisdom |  | 0.081 |  | 0.110 |  | 0.035 |  | 0.735 |  | 0.462 |  |
|  |  | Q13_kSalvation |  | 0.035 |  | 0.097 |  | 0.019 |  | 0.363 |  | 0.717 |  |
|  |  | Q13_lFamSec |  | 0.039 |  | 0.096 |  | 0.024 |  | 0.404 |  | 0.686 |  |
|  |  | Q13_mNatSec |  | -0.162 |  | 0.113 |  | -0.067 |  | -1.440 |  | 0.150 |  |
|  |  | Q13_nAccomp |  | 0.045 |  | 0.117 |  | 0.021 |  | 0.382 |  | 0.703 |  |
|  |  | Q13_oBeauty |  | -0.089 |  | 0.134 |  | -0.031 |  | -0.666 |  | 0.505 |  |
|  |  | Q13_pPeace |  | 0.073 |  | 0.106 |  | 0.039 |  | 0.691 |  | 0.490 |  |
|  |  | Q13_qComfort |  | -0.013 |  | 0.109 |  | -0.009 |  | -0.116 |  | 0.907 |  |
|  | | | | | | | | | | | | | |
