# Supplementary material for: The structure of human motivation
Source: BMC Psychol. 2023 Oct 6;11:308. doi: 10.1186/s40359-023-01346-5 (PMC10557177; doi:10.1186/s40359-023-01346-5)
Supplement: Supplementary file 2 — Additional file 2: SM Table 6.7. Discriminant validity analysis using CICFA(sys): Full output. [file 40359_2023_1346_MOESM2_ESM.docx]

| CFA - Domain - Promotional | |  |  |  |  |  |  | |  |  |  |  |  |  |  |  |  |  |
| --- | --- | --- | --- | --- | --- | --- | --- | --- | --- | --- | --- | --- | --- | --- | --- | --- | --- | --- |
|  |  |  |  |  |  |  |  | |  |  |  |  |  |  |  |  |  |  |
| **Factor variances** | | | | | | | | | | | | | | |  |  |  |  |
|  | | | | | | | | | | | **95% Confidence Interval** | | | |  |  |  |  |
| **Factor** | | **Estimate** | | **Std. Error** | | **z-value** | | | **p** | | **Lower** | | **Upper** | |  |  |  |  |
| Self |  | 1 |  | 0 |  |  |  | |  |  | 1 |  | 1 |  |  |  |  |  |
| Material |  | 1 |  | 0 |  |  |  | |  |  | 1 |  | 1 |  |  |  |  |  |
| Social |  | 1 |  | 0 |  |  |  | |  |  | 1 |  | 1 |  |  |  |  |  |
| Spiritual |  | 1 |  | 0 |  |  |  | |  |  | 1 |  | 1 |  |  |  |  |  |
|  | | | | | | | | | | | | | | |  |  |  |  |
|  |  |  |  |  |  |  |  | |  |  |  |  |  |  |  |  |  |  |
|  |  |  |  |  |  |  |  | |  |  |  |  |  |  |  |  |  |  |
| **Factor Covariances** | | | | | | | | | | | | | | | | | | |
|  | | | | | | | | | | | | | | | **95% Confidence Interval** | | | |
|  | |  | |  | | **Estimate** | | | **Std. Error** | | **z-value** | | **p** | | **Lower** | | **Upper** | |
| Self |  | ↔ |  | Material |  | 0.989 | |  | 0.011 |  | 92.566 |  | < .001 |  | 0.968 |  | 1.009 |  |
| Self |  | ↔ |  | Social |  | 0.921 | |  | 0.013 |  | 70.003 |  | < .001 |  | 0.895 |  | 0.947 |  |
| Self |  | ↔ |  | Spiritual |  | 0.967 | |  | 0.011 |  | 85.582 |  | < .001 |  | 0.945 |  | 0.989 |  |
| Material |  | ↔ |  | Social |  | 0.951 | |  | 0.011 |  | 87.756 |  | < .001 |  | 0.93 |  | 0.972 |  |
| Material |  | ↔ |  | Spiritual |  | 0.966 | |  | 0.01 |  | 92.913 |  | < .001 |  | 0.945 |  | 0.986 |  |
| Social |  | ↔ |  | Spiritual |  | 0.925 |  | | 0.012 |  | 77.983 |  | < .001 |  | 0.901 |  | 0.948 |  |
|  | | | | | | | | | | | | | | | | | | |
|  |  |  |  |  |  |  |  | |  |  |  |  |  |  |  |  |  |  |
|  |  |  |  |  |  |  |  | |  |  |  |  |  |  |  |  |  |  |
| **Average variance extracted** | | | |  |  |  |  | |  |  |  |  |  |  |  |  |  |  |
| **Factor** |  | **AVE** | **SQRT AVE** |  | AVE of .5 acceptable |  |  | |  |  |  |  |  |  |  |  |  |  |
| Self |  | 0.301 | 0.548634669 |  |  |  |  | |  |  |  |  |  |  |  |  |  |  |
| Material |  | 0.35 | 0.591607978 |  |  |  |  | |  |  |  |  |  |  |  |  |  |  |
| Social |  | 0.382 | 0.618061486 |  |  |  |  | |  |  |  |  |  |  |  |  |  |  |
| Spiritual |  | 0.368 | 0.606630036 |  |  |  |  | |  |  |  |  |  |  |  |  |  |  |
|  | | | |  |  |  |  | |  |  |  |  |  |  |  |  |  |  |
|  |  |  |  |  |  |  |  | |  |  |  |  |  |  |  |  |  |  |
|  |  | AVE Self | AVE Material | AVE Social | AVE Spiritual | | Correlation | |  |  |  |  |  |  |  |  |  |  |
| Self | Material | 0.5486347 | 0.591607978 |  |  |  | 0.99 | |  |  |  |  |  |  |  |  |  |  |
| Self | Social | 0.5486347 |  | 0.618061486 |  |  | 0.92 | |  |  |  |  |  |  |  |  |  |  |
| Self | Spiritual | 0.5486347 |  |  | 0.60663 |  | 0.97 | |  |  |  |  |  |  |  |  |  |  |
| Material | Social |  | 0.591607978 | 0.618061486 |  |  | 0.95 | |  |  |  |  |  |  |  |  |  |  |
| Material | Spiritual |  | 0.591607978 |  | 0.60663 |  | 0.97 | |  |  |  |  |  |  |  |  |  |  |
| Social | Spiritual |  |  | 0.618061486 | 0.60663 |  | 0.93 | |  |  |  |  |  |  |  |  |  |  |
|  |  |  |  |  |  |  |  | |  |  |  |  |  |  |  |  |  |  |
|  |  |  |  |  |  |  |  | |  |  |  |  |  |  |  |  |  |  |
| CFA - Domain - Prevention | |  |  |  |  |  |  | |  |  |  |  |  |  |  |  |  |  |
|  |  |  |  |  |  |  |  | |  |  |  |  |  |  |  |  |  |  |
| **Factor variances** | | | | | | | | | | | | | | |  |  |  |  |
|  | | | | | | | | | | | **95% Confidence Interval** | | | |  |  |  |  |
| **Factor** | | **Estimate** | | **Std. Error** | | **z-value** | | | **p** | | **Lower** | | **Upper** | |  |  |  |  |
| Self |  | 1 |  | 0 |  |  |  | |  |  | 1 |  | 1 |  |  |  |  |  |
| Material |  | 1 |  | 0 |  |  |  | |  |  | 1 |  | 1 |  |  |  |  |  |
| Social |  | 1 |  | 0 |  |  |  | |  |  | 1 |  | 1 |  |  |  |  |  |
| Spiritual |  | 1 |  | 0 |  |  |  | |  |  | 1 |  | 1 |  |  |  |  |  |
|  | | | | | | | | | | | | | | |  |  |  |  |
|  |  |  |  |  |  |  |  | |  |  |  |  |  |  |  |  |  |  |
|  |  |  |  |  |  |  |  | |  |  |  |  |  |  |  |  |  |  |
| **Factor Covariances** | | | | | | | | | | | | | | | | | | |
|  | | | | | | | | | | | | | | | **95% Confidence Interval** | | | |
|  | |  | |  | | **Estimate** | | | **Std. Error** | | **z-value** | | **p** | | **Lower** | | **Upper** | |
| Self |  | ↔ |  | Material |  | 0.986 |  | | 0.008 |  | 123.697 |  | < .001 |  | 0.97 |  | 1.001 |  |
| Self |  | ↔ |  | Social |  | 0.962 |  | | 0.009 |  | 109.227 |  | < .001 |  | 0.945 |  | 0.98 |  |
| Self |  | ↔ |  | Spiritual |  | 0.979 |  | | 0.008 |  | 119.445 |  | < .001 |  | 0.963 |  | 0.995 |  |
| Material |  | ↔ |  | Social |  | 0.946 |  | | 0.009 |  | 103.026 |  | < .001 |  | 0.928 |  | 0.964 |  |
| Material |  | ↔ |  | Spiritual |  | 0.954 |  | | 0.009 |  | 105.59 |  | < .001 |  | 0.936 |  | 0.972 |  |
| Social |  | ↔ |  | Spiritual |  | 0.937 |  | | 0.01 |  | 96.706 |  | < .001 |  | 0.918 |  | 0.956 |  |
|  | | | | | | | | | | | | | | | | | | |
|  |  |  |  |  |  |  |  | |  |  |  |  |  |  |  |  |  |  |
|  |  |  |  |  |  |  |  | |  |  |  |  |  |  |  |  |  |  |
| **Average variance extracted** | | | |  |  |  |  | |  |  |  |  |  |  |  |  |  |  |
| **Factor** |  | **AVE** | **AVE SQRT** |  |  |  |  | |  |  |  |  |  |  |  |  |  |  |
| Self |  | 0.394 | 0.627694193 |  |  |  |  | |  |  |  |  |  |  |  |  |  |  |
| Material |  | 0.417 | 0.645755372 |  |  |  |  | |  |  |  |  |  |  |  |  |  |  |
| Social |  | 0.437 | 0.661059755 |  |  |  |  | |  |  |  |  |  |  |  |  |  |  |
| Spiritual |  | 0.421 | 0.648845128 |  |  |  |  | |  |  |  |  |  |  |  |  |  |  |
|  | | | |  |  |  |  | |  |  |  |  |  |  |  |  |  |  |
|  |  |  |  |  |  |  |  | |  |  |  |  |  |  |  |  |  |  |
|  |  | AVE Self | AVE Material | AVE Social | AVE Spiritual | | Correlation | |  |  |  |  |  |  |  |  |  |  |
| Self | Material | 0.6276942 | 0.645755372 |  |  |  | 0.986 | |  |  |  |  |  |  |  |  |  |  |
| Self | Social | 0.6276942 |  | 0.661059755 |  |  | 0.962 | |  |  |  |  |  |  |  |  |  |  |
| Self | Spiritual | 0.6276942 |  |  | 0.648845 |  | 0.979 | |  |  |  |  |  |  |  |  |  |  |
| Material | Social |  | 0.645755372 | 0.661059755 |  |  | 0.946 | |  |  |  |  |  |  |  |  |  |  |
| Material | Spiritual |  | 0.645755372 |  | 0.648845 |  | 0.954 | |  |  |  |  |  |  |  |  |  |  |
| Social | Spiritual |  |  | 0.661059755 | 0.648845 |  | 0.937 | |  |  |  |  |  |  |  |  |  |  |
|  |  |  |  |  |  |  |  | |  |  |  |  |  |  |  |  |  |  |
|  |  |  |  |  |  |  |  | |  |  |  |  |  |  |  |  |  |  |
|  |  |  |  |  |  |  |  | |  |  |  |  |  |  |  |  |  |  |
| CFA - Levels Promotion | |  |  |  |  |  |  | |  |  |  |  |  |  |  |  |  |  |
|  |  |  |  |  |  |  |  | |  |  |  |  |  |  |  |  |  |  |
| **Factor variances** | | | | | | | | | | | | | | |  |  |  |  |
|  | | | | | | | | | | | **95% Confidence Interval** | | | |  |  |  |  |
| **Factor** | | **Estimate** | | **Std. Error** | | **z-value** | | | **p** | | **Lower** | | **Upper** | |  |  |  |  |
| Foundational |  | 1 |  | 0 |  |  |  | |  |  | 1 |  | 1 |  |  |  |  |  |
| Experiential |  | 1 |  | 0 |  |  |  | |  |  | 1 |  | 1 |  |  |  |  |  |
| Aspirational |  | 1 |  | 0 |  |  |  | |  |  | 1 |  | 1 |  |  |  |  |  |
|  | | | | | | | | | | | | | | |  |  |  |  |
|  |  |  |  |  |  |  |  | |  |  |  |  |  |  |  |  |  |  |
|  |  |  |  |  |  |  |  | |  |  |  |  |  |  |  |  |  |  |
| **Factor Covariances** | | | | | | | | | | | | | | | | | | |
|  | | | | | | | | | | | | | | | **95% Confidence Interval** | | | |
|  | |  | |  | | **Estimate** | | | **Std. Error** | | **z-value** | | **p** | | **Lower** | | **Upper** | |
| Foundational |  | ↔ |  | Experiential |  | 0.97 |  | | 0.008 |  | 120.94 |  | < .001 |  | 0.954 |  | 0.986 |  |
| Foundational |  | ↔ |  | Aspirational |  | 0.935 |  | | 0.011 |  | 87.114 |  | < .001 |  | 0.914 |  | 0.956 |  |
| Experiential |  | ↔ |  | Aspirational |  | 0.893 |  | | 0.012 |  | 73.569 |  | < .001 |  | 0.87 |  | 0.917 |  |
|  | | | | | | | | | | | | | | | | | | |
|  |  |  |  |  |  |  |  | |  |  |  |  |  |  |  |  |  |  |
|  |  |  |  |  |  |  |  | |  |  |  |  |  |  |  |  |  |  |
| **Average variance extracted** | | | |  |  |  |  | |  |  |  |  |  |  |  |  |  |  |
| **Factor** |  | **AVE** | **SQRT AVE** |  |  |  |  | |  |  |  |  |  |  |  |  |  |  |
| Foundational |  | 0.354 | 0.594978991 |  |  |  |  | |  |  |  |  |  |  |  |  |  |  |
| Experiential |  | 0.388 | 0.62289646 |  |  |  |  | |  |  |  |  |  |  |  |  |  |  |
| Aspirational |  | 0.322 | 0.567450438 |  |  |  |  | |  |  |  |  |  |  |  |  |  |  |
|  | | | |  |  |  |  | |  |  |  |  |  |  |  |  |  |  |
|  |  |  |  |  |  |  |  | |  |  |  |  |  |  |  |  |  |  |
|  |  | AVE Foundational | AVE Experiential | AVE Aspirational | Correlation | |  | |  |  |  |  |  |  |  |  |  |  |
| Foundational | Experiential | 0.594979 | 0.62289646 |  | 0.97 |  |  | |  |  |  |  |  |  |  |  |  |  |
| Foundational | Aspirational | 0.594979 |  | 0.567450438 | 0.94 |  |  | |  |  |  |  |  |  |  |  |  |  |
| Experiential | Aspirational | | 0.62289646 | 0.567450438 | 0.89 |  |  | |  |  |  |  |  |  |  |  |  |  |
|  |  |  |  |  |  |  |  | |  |  |  |  |  |  |  |  |  |  |
|  |  |  |  |  |  |  |  | |  |  |  |  |  |  |  |  |  |  |
| CFA - Levels Prevention | |  |  |  |  |  |  | |  |  |  |  |  |  |  |  |  |  |
|  |  |  |  |  |  |  |  | |  |  |  |  |  |  |  |  |  |  |
| **Factor variances** | | | | | | | | | | | | | | |  |  |  |  |
|  | | | | | | | | | | | **95% Confidence Interval** | | | |  |  |  |  |
| **Factor** | | **Estimate** | | **Std. Error** | | **z-value** | | | **p** | | **Lower** | | **Upper** | |  |  |  |  |
| Foundational |  | 1 |  | 0 |  |  |  | |  |  | 1 |  | 1 |  |  |  |  |  |
| Experiential |  | 1 |  | 0 |  |  |  | |  |  | 1 |  | 1 |  |  |  |  |  |
| Aspirational |  | 1 |  | 0 |  |  |  | |  |  | 1 |  | 1 |  |  |  |  |  |
|  | | | | | | | | | | | | | | |  |  |  |  |
|  |  |  |  |  |  |  |  | |  |  |  |  |  |  |  |  |  |  |
|  |  |  |  |  |  |  |  | |  |  |  |  |  |  |  |  |  |  |
| **Factor Covariances** | | | | | | | | | | | | | | | | | | |
|  | | | | | | | | | | | | | | | **95% Confidence Interval** | | | |
|  | |  | |  | | **Estimate** | | | **Std. Error** | | **z-value** | | **p** | | **Lower** | | **Upper** | |
| Foundational |  | ↔ |  | Experiential |  | 0.984 |  | | 0.006 |  | 160.088 |  | < .001 |  | 0.972 |  | 0.996 |  |
| Foundational |  | ↔ |  | Aspirational |  | 0.995 |  | | 0.006 |  | 169.624 |  | < .001 |  | 0.984 |  | 1.007 |  |
| Experiential |  | ↔ |  | Aspirational |  | 0.995 |  | | 0.006 |  | 166.602 |  | < .001 |  | 0.983 |  | 1.006 |  |
|  | | | | | | | | | | | | | | | | | | |
|  |  |  |  |  |  |  |  | |  |  |  |  |  |  |  |  |  |  |
|  |  |  |  |  |  |  |  | |  |  |  |  |  |  |  |  |  |  |
| **Average variance extracted** | | | |  |  |  |  | |  |  |  |  |  |  |  |  |  |  |
| **Factor** |  | **AVE** | **SQRT AVE** |  |  |  |  | |  |  |  |  |  |  |  |  |  |  |
| Foundational |  | 0.411 | 0.641092817 |  |  |  |  | |  |  |  |  |  |  |  |  |  |  |
| Experiential |  | 0.402 | 0.634034699 |  |  |  |  | |  |  |  |  |  |  |  |  |  |  |
| Aspirational |  | 0.407 | 0.637965516 |  |  |  |  | |  |  |  |  |  |  |  |  |  |  |
|  | | | |  |  |  |  | |  |  |  |  |  |  |  |  |  |  |
|  |  |  |  |  |  |  |  | |  |  |  |  |  |  |  |  |  |  |
|  |  | AVE Foundational | AVE Experiential | AVE Aspirational | Correlation | |  | |  |  |  |  |  |  |  |  |  |  |
| Foundational | Experiential | 0.6410928 | 0.634034699 |  | 0.97 |  |  | |  |  |  |  |  |  |  |  |  |  |
| Foundational | Aspirational | 0.6410928 |  | 0.637965516 | 0.94 |  |  | |  |  |  |  |  |  |  |  |  |  |
| Experiential | Aspirational | | 0.634034699 | 0.637965516 | 0.89 |  |  | |  |  |  |  |  |  |  |  |  |  |
