# Supplementary material for: The structure of human motivation
Source: BMC Psychol. 2023 Oct 6;11:308. doi: 10.1186/s40359-023-01346-5 (PMC10557177; doi:10.1186/s40359-023-01346-5)
Supplement: Supplementary file 3 — Additional file 3: SM Table 8. Regression models: Image-based measures to predict subject characteristics: Full output. [file 40359_2023_1346_MOESM3_ESM.zip › Supplemental Material Table 8R5.docx]

Supplementary Material Table 8.1 Summary of regression analyses employing image selection data to predict subject characteristics: Full output

**Regression**

**DV: Openness (Big 5)**

**IV: Image-based measures (summed by motive)**

| **Notes** | | |
| --- | --- | --- |
| Output Created | | 11-AUG-2023 09:59:54 |
| Comments | |  |
| Input | Data | C:\Users\jpincus\Desktop\Usefuls\LIS\LIS Perspectives\Workforce Listening Surveys\Wave 6\WFL6.merged.sav |
|  | Active Dataset | DataSet2 |
|  | Filter | <none> |
|  | Weight | weight1 |
|  | Split File | <none> |
|  | N of Rows in Working Data File | 976 |
| Missing Value Handling | Definition of Missing | User-defined missing values are treated as missing. |
|  | Cases Used | Statistics are based on cases with no missing values for any variable used. |
| Syntax | | REGRESSION  /MISSING LISTWISE  /STATISTICS COEFF OUTS R ANOVA  /CRITERIA=PIN(.05) POUT(.10)  /NOORIGIN  /DEPENDENT OPENNESS  /METHOD=ENTER a1n_sum a2n_sum a3n_sum b1n_sum b2n_sum b3n_sum c1n_sum c2n_sum c3n_sum d1n_sum  d2n_sum d3n_sum a1p_sum a2p_sum a3p_sum b1p_sum b2p_sum b3p_sum c1p_sum c2p_sum c3p_sum d1p_sum  d2p_sum d3p_sum. |
| Resources | Processor Time | 00:00:00.13 |
|  | Elapsed Time | 00:00:00.10 |
|  | Memory Required | 61072 bytes |
|  | Additional Memory Required for Residual Plots | 0 bytes |

| **Variables Entered/Removed^a^** | | | |
| --- | --- | --- | --- |
| Model | Variables Entered | Variables Removed | Method |
| 1 | d3p_sum, b2n_sum, d2p_sum, d3n_sum, a3p_sum, c3p_sum, a1p_sum, c1n_sum, d1p_sum, a2p_sum, b2p_sum, b3n_sum, c1p_sum, c2n_sum, a2n_sum, b3p_sum, c2p_sum, a3n_sum, c3n_sum, d1n_sum, d2n_sum, a1n_sum^b^ | . | Enter |
| a. Dependent Variable: OPENNESS | | | |
| b. Tolerance = .000 limit reached. | | | |

| **Model Summary** | | | | |
| --- | --- | --- | --- | --- |
| Model | R | R Square | Adjusted R Square | Std. Error of the Estimate |
| 1 | .240^a^ | .058 | .036 | .82162 |
| a. Predictors: (Constant), d3p_sum, b2n_sum, d2p_sum, d3n_sum, a3p_sum, c3p_sum, a1p_sum, c1n_sum, d1p_sum, a2p_sum, b2p_sum, b3n_sum, c1p_sum, c2n_sum, a2n_sum, b3p_sum, c2p_sum, a3n_sum, c3n_sum, d1n_sum, d2n_sum, a1n_sum | | | | |

| **ANOVA^a^** | | | | | | |
| --- | --- | --- | --- | --- | --- | --- |
| Model | | Sum of Squares | df | Mean Square | F | Sig. |
| 1 | Regression | 39.856 | 22 | 1.812 | 2.684 | .000^b^ |
|  | Residual | 650.225 | 963 | .675 |  |  |
|  | Total | 690.081 | 985 |  |  |  |
| a. Dependent Variable: OPENNESS | | | | | | |
| b. Predictors: (Constant), d3p_sum, b2n_sum, d2p_sum, d3n_sum, a3p_sum, c3p_sum, a1p_sum, c1n_sum, d1p_sum, a2p_sum, b2p_sum, b3n_sum, c1p_sum, c2n_sum, a2n_sum, b3p_sum, c2p_sum, a3n_sum, c3n_sum, d1n_sum, d2n_sum, a1n_sum | | | | | | |

| **Coefficients^a^** | | | | | | |
| --- | --- | --- | --- | --- | --- | --- |
| Model | | Unstandardized Coefficients | | Standardized Coefficients | t | Sig. |
|  |  | B | Std. Error | Beta |  |  |
| 1 | (Constant) | 3.577 | .068 |  | 52.623 | .000 |
|  | a1n_sum | .059 | .035 | .074 | 1.668 | .096 |
|  | a2n_sum | .026 | .033 | .030 | .773 | .440 |
|  | a3n_sum | .010 | .033 | .012 | .295 | .768 |
|  | b2n_sum | -.013 | .029 | -.017 | -.452 | .652 |
|  | b3n_sum | -.031 | .031 | -.040 | -.987 | .324 |
|  | c1n_sum | -.073 | .032 | -.093 | -2.266 | .024 |
|  | c2n_sum | -.004 | .030 | -.005 | -.118 | .906 |
|  | c3n_sum | -.019 | .029 | -.027 | -.655 | .512 |
|  | d1n_sum | .008 | .032 | .011 | .268 | .789 |
|  | d2n_sum | .072 | .034 | .093 | 2.154 | .032 |
|  | d3n_sum | -.030 | .028 | -.039 | -1.052 | .293 |
|  | a1p_sum | -.050 | .031 | -.060 | -1.610 | .108 |
|  | a2p_sum | .072 | .034 | .081 | 2.096 | .036 |
|  | a3p_sum | -.038 | .027 | -.049 | -1.383 | .167 |
|  | b2p_sum | .009 | .032 | .011 | .296 | .767 |
|  | b3p_sum | .086 | .033 | .104 | 2.644 | .008 |
|  | c1p_sum | .061 | .031 | .076 | 1.947 | .052 |
|  | c2p_sum | -.019 | .032 | -.022 | -.573 | .567 |
|  | c3p_sum | .020 | .028 | .026 | .704 | .482 |
|  | d1p_sum | .007 | .031 | .008 | .210 | .834 |
|  | d2p_sum | .005 | .028 | .007 | .179 | .858 |
|  | d3p_sum | .024 | .030 | .031 | .810 | .418 |
| a. Dependent Variable: OPENNESS | | | | | | |

| **Excluded Variables^a^** | | | | | | |
| --- | --- | --- | --- | --- | --- | --- |
| Model | | Beta In | t | Sig. | Partial Correlation | Collinearity Statistics |
|  |  |  |  |  |  | Tolerance |
| 1 | b1n_sum | .^b^ | . | . | . | .000 |
|  | b1p_sum | .^b^ | . | . | . | .000 |
| a. Dependent Variable: OPENNESS | | | | | | |
| b. Predictors in the Model: (Constant), d3p_sum, b2n_sum, d2p_sum, d3n_sum, a3p_sum, c3p_sum, a1p_sum, c1n_sum, d1p_sum, a2p_sum, b2p_sum, b3n_sum, c1p_sum, c2n_sum, a2n_sum, b3p_sum, c2p_sum, a3n_sum, c3n_sum, d1n_sum, d2n_sum, a1n_sum | | | | | | |

**Supplementary Material Table 8.2**

**Regression**

**DV: Extraversion (Big 5)**

**IV: Image-based measures (summed by motive)**

| **Notes** | | |
| --- | --- | --- |
| Output Created | | 11-AUG-2023 10:01:06 |
| Comments | |  |
| Input | Data | C:\Users\jpincus\Desktop\Usefuls\LIS\LIS Perspectives\Workforce Listening Surveys\Wave 6\WFL6.merged.sav |
|  | Active Dataset | DataSet2 |
|  | Filter | <none> |
|  | Weight | weight1 |
|  | Split File | <none> |
|  | N of Rows in Working Data File | 976 |
| Missing Value Handling | Definition of Missing | User-defined missing values are treated as missing. |
|  | Cases Used | Statistics are based on cases with no missing values for any variable used. |
| Syntax | | REGRESSION  /MISSING LISTWISE  /STATISTICS COEFF OUTS R ANOVA  /CRITERIA=PIN(.05) POUT(.10)  /NOORIGIN  /DEPENDENT EXTRAVERSION  /METHOD=ENTER a1n_sum a2n_sum a3n_sum b1n_sum b2n_sum b3n_sum c1n_sum c2n_sum c3n_sum d1n_sum  d2n_sum d3n_sum a1p_sum a2p_sum a3p_sum b1p_sum b2p_sum b3p_sum c1p_sum c2p_sum c3p_sum d1p_sum  d2p_sum d3p_sum. |
| Resources | Processor Time | 00:00:00.08 |
|  | Elapsed Time | 00:00:00.09 |
|  | Memory Required | 61072 bytes |
|  | Additional Memory Required for Residual Plots | 0 bytes |

| **Variables Entered/Removed^a^** | | | |
| --- | --- | --- | --- |
| Model | Variables Entered | Variables Removed | Method |
| 1 | d3p_sum, b2n_sum, d2p_sum, d3n_sum, a3p_sum, c3p_sum, a1p_sum, c1n_sum, d1p_sum, a2p_sum, b2p_sum, b3n_sum, c1p_sum, c2n_sum, a2n_sum, b3p_sum, c2p_sum, a3n_sum, c3n_sum, d1n_sum, d2n_sum, a1n_sum^b^ | . | Enter |
| a. Dependent Variable: EXTRAVERSION | | | |
| b. Tolerance = .000 limit reached. | | | |

| **Model Summary** | | | | |
| --- | --- | --- | --- | --- |
| Model | R | R Square | Adjusted R Square | Std. Error of the Estimate |
| 1 | .262^a^ | .069 | .047 | .87094 |
| a. Predictors: (Constant), d3p_sum, b2n_sum, d2p_sum, d3n_sum, a3p_sum, c3p_sum, a1p_sum, c1n_sum, d1p_sum, a2p_sum, b2p_sum, b3n_sum, c1p_sum, c2n_sum, a2n_sum, b3p_sum, c2p_sum, a3n_sum, c3n_sum, d1n_sum, d2n_sum, a1n_sum | | | | |

| **ANOVA^a^** | | | | | | |
| --- | --- | --- | --- | --- | --- | --- |
| Model | | Sum of Squares | df | Mean Square | F | Sig. |
| 1 | Regression | 53.913 | 22 | 2.451 | 3.231 | .000^b^ |
|  | Residual | 730.623 | 963 | .759 |  |  |
|  | Total | 784.536 | 985 |  |  |  |
| a. Dependent Variable: EXTRAVERSION | | | | | | |
| b. Predictors: (Constant), d3p_sum, b2n_sum, d2p_sum, d3n_sum, a3p_sum, c3p_sum, a1p_sum, c1n_sum, d1p_sum, a2p_sum, b2p_sum, b3n_sum, c1p_sum, c2n_sum, a2n_sum, b3p_sum, c2p_sum, a3n_sum, c3n_sum, d1n_sum, d2n_sum, a1n_sum | | | | | | |

| **Coefficients^a^** | | | | | | |
| --- | --- | --- | --- | --- | --- | --- |
| Model | | Unstandardized Coefficients | | Standardized Coefficients | t | Sig. |
|  |  | B | Std. Error | Beta |  |  |
| 1 | (Constant) | 3.149 | .072 |  | 43.696 | .000 |
|  | a1n_sum | .089 | .037 | .106 | 2.379 | .018 |
|  | a2n_sum | -.053 | .035 | -.058 | -1.492 | .136 |
|  | a3n_sum | -.001 | .035 | -.002 | -.041 | .967 |
|  | b2n_sum | -.037 | .031 | -.046 | -1.198 | .231 |
|  | b3n_sum | -.033 | .033 | -.040 | -.991 | .322 |
|  | c1n_sum | -.042 | .034 | -.051 | -1.244 | .214 |
|  | c2n_sum | -.124 | .032 | -.157 | -3.910 | .000 |
|  | c3n_sum | -.026 | .031 | -.035 | -.850 | .396 |
|  | d1n_sum | .017 | .033 | .021 | .513 | .608 |
|  | d2n_sum | .024 | .036 | .029 | .680 | .496 |
|  | d3n_sum | .035 | .030 | .044 | 1.172 | .242 |
|  | a1p_sum | -.034 | .033 | -.037 | -1.012 | .312 |
|  | a2p_sum | .068 | .036 | .072 | 1.870 | .062 |
|  | a3p_sum | .006 | .029 | .008 | .217 | .828 |
|  | b2p_sum | -.034 | .033 | -.039 | -1.026 | .305 |
|  | b3p_sum | .006 | .035 | .007 | .185 | .853 |
|  | c1p_sum | .113 | .033 | .133 | 3.428 | .001 |
|  | c2p_sum | -.011 | .034 | -.013 | -.332 | .740 |
|  | c3p_sum | .122 | .030 | .150 | 4.044 | .000 |
|  | d1p_sum | -.049 | .033 | -.057 | -1.500 | .134 |
|  | d2p_sum | .009 | .030 | .011 | .304 | .761 |
|  | d3p_sum | -.040 | .032 | -.047 | -1.260 | .208 |
| a. Dependent Variable: EXTRAVERSION | | | | | | |

| **Excluded Variables^a^** | | | | | | |
| --- | --- | --- | --- | --- | --- | --- |
| Model | | Beta In | t | Sig. | Partial Correlation | Collinearity Statistics |
|  |  |  |  |  |  | Tolerance |
| 1 | b1n_sum | .^b^ | . | . | . | .000 |
|  | b1p_sum | .^b^ | . | . | . | .000 |
| a. Dependent Variable: EXTRAVERSION | | | | | | |
| b. Predictors in the Model: (Constant), d3p_sum, b2n_sum, d2p_sum, d3n_sum, a3p_sum, c3p_sum, a1p_sum, c1n_sum, d1p_sum, a2p_sum, b2p_sum, b3n_sum, c1p_sum, c2n_sum, a2n_sum, b3p_sum, c2p_sum, a3n_sum, c3n_sum, d1n_sum, d2n_sum, a1n_sum | | | | | | |

**Supplementary Material Table 8.3**

**Regression**

**DV: Agreeableness (Big 5)**

**IV: Image-based measures (summed by motive)**

| **Notes** | | |
| --- | --- | --- |
| Output Created | | 11-AUG-2023 10:01:47 |
| Comments | |  |
| Input | Data | C:\Users\jpincus\Desktop\Usefuls\LIS\LIS Perspectives\Workforce Listening Surveys\Wave 6\WFL6.merged.sav |
|  | Active Dataset | DataSet2 |
|  | Filter | <none> |
|  | Weight | weight1 |
|  | Split File | <none> |
|  | N of Rows in Working Data File | 976 |
| Missing Value Handling | Definition of Missing | User-defined missing values are treated as missing. |
|  | Cases Used | Statistics are based on cases with no missing values for any variable used. |
| Syntax | | REGRESSION  /MISSING LISTWISE  /STATISTICS COEFF OUTS R ANOVA  /CRITERIA=PIN(.05) POUT(.10)  /NOORIGIN  /DEPENDENT AGREEABLE  /METHOD=ENTER a1n_sum a2n_sum a3n_sum b1n_sum b2n_sum b3n_sum c1n_sum c2n_sum c3n_sum d1n_sum  d2n_sum d3n_sum a1p_sum a2p_sum a3p_sum b1p_sum b2p_sum b3p_sum c1p_sum c2p_sum c3p_sum d1p_sum  d2p_sum d3p_sum. |
| Resources | Processor Time | 00:00:00.11 |
|  | Elapsed Time | 00:00:00.10 |
|  | Memory Required | 61072 bytes |
|  | Additional Memory Required for Residual Plots | 0 bytes |

| **Variables Entered/Removed^a^** | | | |
| --- | --- | --- | --- |
| Model | Variables Entered | Variables Removed | Method |
| 1 | d3p_sum, b2n_sum, d2p_sum, d3n_sum, a3p_sum, c3p_sum, a1p_sum, c1n_sum, d1p_sum, a2p_sum, b2p_sum, b3n_sum, c1p_sum, c2n_sum, a2n_sum, b3p_sum, c2p_sum, a3n_sum, c3n_sum, d1n_sum, d2n_sum, a1n_sum^b^ | . | Enter |
| a. Dependent Variable: AGREEABLE | | | |
| b. Tolerance = .000 limit reached. | | | |

| **Model Summary** | | | | |
| --- | --- | --- | --- | --- |
| Model | R | R Square | Adjusted R Square | Std. Error of the Estimate |
| 1 | .194^a^ | .038 | .016 | .79489 |
| a. Predictors: (Constant), d3p_sum, b2n_sum, d2p_sum, d3n_sum, a3p_sum, c3p_sum, a1p_sum, c1n_sum, d1p_sum, a2p_sum, b2p_sum, b3n_sum, c1p_sum, c2n_sum, a2n_sum, b3p_sum, c2p_sum, a3n_sum, c3n_sum, d1n_sum, d2n_sum, a1n_sum | | | | |

| **ANOVA^a^** | | | | | | |
| --- | --- | --- | --- | --- | --- | --- |
| Model | | Sum of Squares | df | Mean Square | F | Sig. |
| 1 | Regression | 23.858 | 22 | 1.084 | 1.716 | .021^b^ |
|  | Residual | 608.607 | 963 | .632 |  |  |
|  | Total | 632.465 | 985 |  |  |  |
| a. Dependent Variable: AGREEABLE | | | | | | |
| b. Predictors: (Constant), d3p_sum, b2n_sum, d2p_sum, d3n_sum, a3p_sum, c3p_sum, a1p_sum, c1n_sum, d1p_sum, a2p_sum, b2p_sum, b3n_sum, c1p_sum, c2n_sum, a2n_sum, b3p_sum, c2p_sum, a3n_sum, c3n_sum, d1n_sum, d2n_sum, a1n_sum | | | | | | |

| **Coefficients^a^** | | | | | | |
| --- | --- | --- | --- | --- | --- | --- |
| Model | | Unstandardized Coefficients | | Standardized Coefficients | t | Sig. |
|  |  | B | Std. Error | Beta |  |  |
| 1 | (Constant) | 3.711 | .066 |  | 56.423 | .000 |
|  | a1n_sum | -.002 | .034 | -.002 | -.044 | .965 |
|  | a2n_sum | -.031 | .032 | -.038 | -.956 | .339 |
|  | a3n_sum | -.026 | .032 | -.034 | -.827 | .408 |
|  | b2n_sum | -.012 | .028 | -.017 | -.435 | .664 |
|  | b3n_sum | .010 | .030 | .014 | .342 | .732 |
|  | c1n_sum | -.008 | .031 | -.011 | -.263 | .792 |
|  | c2n_sum | .028 | .029 | .040 | .986 | .325 |
|  | c3n_sum | .033 | .028 | .048 | 1.166 | .244 |
|  | d1n_sum | -.007 | .031 | -.009 | -.217 | .828 |
|  | d2n_sum | -.063 | .032 | -.085 | -1.953 | .051 |
|  | d3n_sum | .078 | .027 | .108 | 2.860 | .004 |
|  | a1p_sum | .022 | .030 | .027 | .711 | .477 |
|  | a2p_sum | .000 | .033 | .000 | -.007 | .994 |
|  | a3p_sum | .038 | .026 | .052 | 1.438 | .151 |
|  | b2p_sum | -.041 | .031 | -.052 | -1.335 | .182 |
|  | b3p_sum | -.037 | .032 | -.046 | -1.167 | .243 |
|  | c1p_sum | .050 | .030 | .065 | 1.654 | .099 |
|  | c2p_sum | -.066 | .031 | -.083 | -2.097 | .036 |
|  | c3p_sum | .053 | .027 | .072 | 1.921 | .055 |
|  | d1p_sum | -.009 | .030 | -.012 | -.306 | .759 |
|  | d2p_sum | .070 | .027 | .095 | 2.561 | .011 |
|  | d3p_sum | -.016 | .029 | -.021 | -.558 | .577 |
| a. Dependent Variable: AGREEABLE | | | | | | |

| **Excluded Variables^a^** | | | | | | |
| --- | --- | --- | --- | --- | --- | --- |
| Model | | Beta In | t | Sig. | Partial Correlation | Collinearity Statistics |
|  |  |  |  |  |  | Tolerance |
| 1 | b1n_sum | .^b^ | . | . | . | .000 |
|  | b1p_sum | .^b^ | . | . | . | .000 |
| a. Dependent Variable: AGREEABLE | | | | | | |
| b. Predictors in the Model: (Constant), d3p_sum, b2n_sum, d2p_sum, d3n_sum, a3p_sum, c3p_sum, a1p_sum, c1n_sum, d1p_sum, a2p_sum, b2p_sum, b3n_sum, c1p_sum, c2n_sum, a2n_sum, b3p_sum, c2p_sum, a3n_sum, c3n_sum, d1n_sum, d2n_sum, a1n_sum | | | | | | |

**Supplementary Material Table 8.4**

**Regression**

**DV: Conscientiousness (Big 5)**

**IV: Image-based measures (summed by motive)**

| **Notes** | | |
| --- | --- | --- |
| Output Created | | 11-AUG-2023 10:02:47 |
| Comments | |  |
| Input | Data | C:\Users\jpincus\Desktop\Usefuls\LIS\LIS Perspectives\Workforce Listening Surveys\Wave 6\WFL6.merged.sav |
|  | Active Dataset | DataSet2 |
|  | Filter | <none> |
|  | Weight | weight1 |
|  | Split File | <none> |
|  | N of Rows in Working Data File | 976 |
| Missing Value Handling | Definition of Missing | User-defined missing values are treated as missing. |
|  | Cases Used | Statistics are based on cases with no missing values for any variable used. |
| Syntax | | REGRESSION  /MISSING LISTWISE  /STATISTICS COEFF OUTS R ANOVA  /CRITERIA=PIN(.05) POUT(.10)  /NOORIGIN  /DEPENDENT CONSCIENTIOUS  /METHOD=ENTER a1n_sum a2n_sum a3n_sum b1n_sum b2n_sum b3n_sum c1n_sum c2n_sum c3n_sum d1n_sum  d2n_sum d3n_sum a1p_sum a2p_sum a3p_sum b1p_sum b2p_sum b3p_sum c1p_sum c2p_sum c3p_sum d1p_sum  d2p_sum d3p_sum. |
| Resources | Processor Time | 00:00:00.12 |
|  | Elapsed Time | 00:00:00.10 |
|  | Memory Required | 61072 bytes |
|  | Additional Memory Required for Residual Plots | 0 bytes |

| **Variables Entered/Removed^a^** | | | |
| --- | --- | --- | --- |
| Model | Variables Entered | Variables Removed | Method |
| 1 | d3p_sum, b2n_sum, d2p_sum, d3n_sum, a3p_sum, c3p_sum, a1p_sum, c1n_sum, d1p_sum, a2p_sum, b2p_sum, b3n_sum, c1p_sum, c2n_sum, a2n_sum, b3p_sum, c2p_sum, a3n_sum, c3n_sum, d1n_sum, d2n_sum, a1n_sum^b^ | . | Enter |
| a. Dependent Variable: CONSCIENTIOUS | | | |
| b. Tolerance = .000 limit reached. | | | |

| **Model Summary** | | | | |
| --- | --- | --- | --- | --- |
| Model | R | R Square | Adjusted R Square | Std. Error of the Estimate |
| 1 | .230^a^ | .053 | .031 | .73346 |
| a. Predictors: (Constant), d3p_sum, b2n_sum, d2p_sum, d3n_sum, a3p_sum, c3p_sum, a1p_sum, c1n_sum, d1p_sum, a2p_sum, b2p_sum, b3n_sum, c1p_sum, c2n_sum, a2n_sum, b3p_sum, c2p_sum, a3n_sum, c3n_sum, d1n_sum, d2n_sum, a1n_sum | | | | |

| **ANOVA^a^** | | | | | | |
| --- | --- | --- | --- | --- | --- | --- |
| Model | | Sum of Squares | df | Mean Square | F | Sig. |
| 1 | Regression | 29.007 | 22 | 1.318 | 2.451 | .000^b^ |
|  | Residual | 518.171 | 963 | .538 |  |  |
|  | Total | 547.178 | 985 |  |  |  |
| a. Dependent Variable: CONSCIENTIOUS | | | | | | |
| b. Predictors: (Constant), d3p_sum, b2n_sum, d2p_sum, d3n_sum, a3p_sum, c3p_sum, a1p_sum, c1n_sum, d1p_sum, a2p_sum, b2p_sum, b3n_sum, c1p_sum, c2n_sum, a2n_sum, b3p_sum, c2p_sum, a3n_sum, c3n_sum, d1n_sum, d2n_sum, a1n_sum | | | | | | |

| **Coefficients^a^** | | | | | | |
| --- | --- | --- | --- | --- | --- | --- |
| Model | | Unstandardized Coefficients | | Standardized Coefficients | t | Sig. |
|  |  | B | Std. Error | Beta |  |  |
| 1 | (Constant) | 3.923 | .061 |  | 64.647 | .000 |
|  | a1n_sum | -.044 | .032 | -.062 | -1.384 | .167 |
|  | a2n_sum | .014 | .030 | .019 | .474 | .636 |
|  | a3n_sum | .058 | .029 | .081 | 1.988 | .047 |
|  | b2n_sum | -.051 | .026 | -.076 | -1.968 | .049 |
|  | b3n_sum | .011 | .028 | .016 | .394 | .694 |
|  | c1n_sum | -.048 | .029 | -.068 | -1.665 | .096 |
|  | c2n_sum | -.025 | .027 | -.038 | -.944 | .345 |
|  | c3n_sum | -.025 | .026 | -.039 | -.948 | .343 |
|  | d1n_sum | .043 | .028 | .064 | 1.545 | .123 |
|  | d2n_sum | .006 | .030 | .009 | .210 | .834 |
|  | d3n_sum | .027 | .025 | .041 | 1.089 | .276 |
|  | a1p_sum | -.039 | .028 | -.052 | -1.399 | .162 |
|  | a2p_sum | -.038 | .031 | -.048 | -1.234 | .217 |
|  | a3p_sum | .063 | .024 | .092 | 2.583 | .010 |
|  | b2p_sum | -.040 | .028 | -.054 | -1.409 | .159 |
|  | b3p_sum | .032 | .029 | .043 | 1.106 | .269 |
|  | c1p_sum | .067 | .028 | .095 | 2.420 | .016 |
|  | c2p_sum | -.048 | .029 | -.066 | -1.679 | .093 |
|  | c3p_sum | .056 | .025 | .083 | 2.223 | .026 |
|  | d1p_sum | .009 | .028 | .012 | .312 | .755 |
|  | d2p_sum | -.003 | .025 | -.004 | -.115 | .908 |
|  | d3p_sum | -.058 | .027 | -.083 | -2.174 | .030 |
| a. Dependent Variable: CONSCIENTIOUS | | | | | | |

| **Excluded Variables^a^** | | | | | | |
| --- | --- | --- | --- | --- | --- | --- |
| Model | | Beta In | t | Sig. | Partial Correlation | Collinearity Statistics |
|  |  |  |  |  |  | Tolerance |
| 1 | b1n_sum | .^b^ | . | . | . | .000 |
|  | b1p_sum | .^b^ | . | . | . | .000 |
| a. Dependent Variable: CONSCIENTIOUS | | | | | | |
| b. Predictors in the Model: (Constant), d3p_sum, b2n_sum, d2p_sum, d3n_sum, a3p_sum, c3p_sum, a1p_sum, c1n_sum, d1p_sum, a2p_sum, b2p_sum, b3n_sum, c1p_sum, c2n_sum, a2n_sum, b3p_sum, c2p_sum, a3n_sum, c3n_sum, d1n_sum, d2n_sum, a1n_sum | | | | | | |

**Supplementary Material Table 8.5**

**Regression**

**DV: Neuroticism (Big 5)**

**IV: Image-based measures (summed by motive)**

| **Notes** | | |
| --- | --- | --- |
| Output Created | | 11-AUG-2023 10:03:56 |
| Comments | |  |
| Input | Data | C:\Users\jpincus\Desktop\Usefuls\LIS\LIS Perspectives\Workforce Listening Surveys\Wave 6\WFL6.merged.sav |
|  | Active Dataset | DataSet2 |
|  | Filter | <none> |
|  | Weight | weight1 |
|  | Split File | <none> |
|  | N of Rows in Working Data File | 976 |
| Missing Value Handling | Definition of Missing | User-defined missing values are treated as missing. |
|  | Cases Used | Statistics are based on cases with no missing values for any variable used. |
| Syntax | | REGRESSION  /MISSING LISTWISE  /STATISTICS COEFF OUTS R ANOVA  /CRITERIA=PIN(.05) POUT(.10)  /NOORIGIN  /DEPENDENT NEUROTICISM  /METHOD=ENTER a1n_sum a2n_sum a3n_sum b1n_sum b2n_sum b3n_sum c1n_sum c2n_sum c3n_sum d1n_sum  d2n_sum d3n_sum a1p_sum a2p_sum a3p_sum b1p_sum b2p_sum b3p_sum c1p_sum c2p_sum c3p_sum d1p_sum  d2p_sum d3p_sum. |
| Resources | Processor Time | 00:00:00.06 |
|  | Elapsed Time | 00:00:00.09 |
|  | Memory Required | 61072 bytes |
|  | Additional Memory Required for Residual Plots | 0 bytes |

| **Variables Entered/Removed^a^** | | | |
| --- | --- | --- | --- |
| Model | Variables Entered | Variables Removed | Method |
| 1 | d3p_sum, b2n_sum, d2p_sum, d3n_sum, a3p_sum, c3p_sum, a1p_sum, c1n_sum, d1p_sum, a2p_sum, b2p_sum, b3n_sum, c1p_sum, c2n_sum, a2n_sum, b3p_sum, c2p_sum, a3n_sum, c3n_sum, d1n_sum, d2n_sum, a1n_sum^b^ | . | Enter |
| a. Dependent Variable: NEUROTICISM | | | |
| b. Tolerance = .000 limit reached. | | | |

| **Model Summary** | | | | |
| --- | --- | --- | --- | --- |
| Model | R | R Square | Adjusted R Square | Std. Error of the Estimate |
| 1 | .327^a^ | .107 | .086 | .93299 |
| a. Predictors: (Constant), d3p_sum, b2n_sum, d2p_sum, d3n_sum, a3p_sum, c3p_sum, a1p_sum, c1n_sum, d1p_sum, a2p_sum, b2p_sum, b3n_sum, c1p_sum, c2n_sum, a2n_sum, b3p_sum, c2p_sum, a3n_sum, c3n_sum, d1n_sum, d2n_sum, a1n_sum | | | | |

| **ANOVA^a^** | | | | | | |
| --- | --- | --- | --- | --- | --- | --- |
| Model | | Sum of Squares | df | Mean Square | F | Sig. |
| 1 | Regression | 100.300 | 22 | 4.559 | 5.237 | .000^b^ |
|  | Residual | 838.454 | 963 | .870 |  |  |
|  | Total | 938.754 | 985 |  |  |  |
| a. Dependent Variable: NEUROTICISM | | | | | | |
| b. Predictors: (Constant), d3p_sum, b2n_sum, d2p_sum, d3n_sum, a3p_sum, c3p_sum, a1p_sum, c1n_sum, d1p_sum, a2p_sum, b2p_sum, b3n_sum, c1p_sum, c2n_sum, a2n_sum, b3p_sum, c2p_sum, a3n_sum, c3n_sum, d1n_sum, d2n_sum, a1n_sum | | | | | | |

| **Coefficients^a^** | | | | | | |
| --- | --- | --- | --- | --- | --- | --- |
| Model | | Unstandardized Coefficients | | Standardized Coefficients | t | Sig. |
|  |  | B | Std. Error | Beta |  |  |
| 1 | (Constant) | 2.617 | .077 |  | 33.901 | .000 |
|  | a1n_sum | .047 | .040 | .051 | 1.170 | .242 |
|  | a2n_sum | -.043 | .038 | -.043 | -1.140 | .255 |
|  | a3n_sum | -.038 | .037 | -.041 | -1.034 | .301 |
|  | b2n_sum | .077 | .033 | .089 | 2.370 | .018 |
|  | b3n_sum | .027 | .036 | .031 | .767 | .443 |
|  | c1n_sum | .001 | .036 | .001 | .015 | .988 |
|  | c2n_sum | .161 | .034 | .187 | 4.759 | .000 |
|  | c3n_sum | .113 | .033 | .136 | 3.404 | .001 |
|  | d1n_sum | .022 | .036 | .025 | .620 | .535 |
|  | d2n_sum | -.007 | .038 | -.008 | -.193 | .847 |
|  | d3n_sum | -.065 | .032 | -.074 | -2.021 | .044 |
|  | a1p_sum | -.004 | .036 | -.005 | -.125 | .901 |
|  | a2p_sum | .025 | .039 | .024 | .636 | .525 |
|  | a3p_sum | -.047 | .031 | -.053 | -1.515 | .130 |
|  | b2p_sum | .030 | .036 | .031 | .835 | .404 |
|  | b3p_sum | -.002 | .037 | -.003 | -.068 | .946 |
|  | c1p_sum | -.072 | .035 | -.078 | -2.044 | .041 |
|  | c2p_sum | .105 | .037 | .110 | 2.875 | .004 |
|  | c3p_sum | -.065 | .032 | -.073 | -2.010 | .045 |
|  | d1p_sum | .025 | .035 | .026 | .702 | .483 |
|  | d2p_sum | -.092 | .032 | -.102 | -2.868 | .004 |
|  | d3p_sum | -.001 | .034 | -.001 | -.024 | .981 |
| a. Dependent Variable: NEUROTICISM | | | | | | |

| **Excluded Variables^a^** | | | | | | |
| --- | --- | --- | --- | --- | --- | --- |
| Model | | Beta In | t | Sig. | Partial Correlation | Collinearity Statistics |
|  |  |  |  |  |  | Tolerance |
| 1 | b1n_sum | .^b^ | . | . | . | .000 |
|  | b1p_sum | .^b^ | . | . | . | .000 |
| a. Dependent Variable: NEUROTICISM | | | | | | |
| b. Predictors in the Model: (Constant), d3p_sum, b2n_sum, d2p_sum, d3n_sum, a3p_sum, c3p_sum, a1p_sum, c1n_sum, d1p_sum, a2p_sum, b2p_sum, b3n_sum, c1p_sum, c2n_sum, a2n_sum, b3p_sum, c2p_sum, a3n_sum, c3n_sum, d1n_sum, d2n_sum, a1n_sum | | | | | | |

**Supplementary Material Table 8.6**

**Regression**

**DV: Depression (Big 5)**

**IV: Image-based measures (summed by motive)**

| **Notes** | | |
| --- | --- | --- |
| Output Created | | 11-AUG-2023 10:17:14 |
| Comments | |  |
| Input | Data | C:\Users\jpincus\Desktop\Usefuls\LIS\LIS Perspectives\Workforce Listening Surveys\Wave 6\WFL6.merged.sav |
|  | Active Dataset | DataSet2 |
|  | Filter | <none> |
|  | Weight | weight1 |
|  | Split File | <none> |
|  | N of Rows in Working Data File | 976 |
| Missing Value Handling | Definition of Missing | User-defined missing values are treated as missing. |
|  | Cases Used | Statistics are based on cases with no missing values for any variable used. |
| Syntax | | REGRESSION  /MISSING LISTWISE  /STATISTICS COEFF OUTS R ANOVA  /CRITERIA=PIN(.05) POUT(.10)  /NOORIGIN  /DEPENDENT Depression  /METHOD=ENTER a1n_sum a2n_sum a3n_sum b1n_sum b2n_sum b3n_sum c1n_sum c2n_sum c3n_sum d1n_sum  d2n_sum d3n_sum a1p_sum a2p_sum a3p_sum b1p_sum b2p_sum b3p_sum c1p_sum c2p_sum c3p_sum d1p_sum  d2p_sum d3p_sum. |
| Resources | Processor Time | 00:00:00.11 |
|  | Elapsed Time | 00:00:00.09 |
|  | Memory Required | 61072 bytes |
|  | Additional Memory Required for Residual Plots | 0 bytes |

| **Variables Entered/Removed^a^** | | | |
| --- | --- | --- | --- |
| Model | Variables Entered | Variables Removed | Method |
| 1 | d3p_sum, b2n_sum, d2p_sum, d3n_sum, a3p_sum, c3p_sum, a1p_sum, c1n_sum, d1p_sum, a2p_sum, b2p_sum, b3n_sum, c1p_sum, c2n_sum, a2n_sum, b3p_sum, c2p_sum, a3n_sum, c3n_sum, d1n_sum, d2n_sum, a1n_sum^b^ | . | Enter |
| a. Dependent Variable: Depression | | | |
| b. Tolerance = .000 limit reached. | | | |

| **Model Summary** | | | | |
| --- | --- | --- | --- | --- |
| Model | R | R Square | Adjusted R Square | Std. Error of the Estimate |
| 1 | .430^a^ | .184 | .166 | 6.03115 |
| a. Predictors: (Constant), d3p_sum, b2n_sum, d2p_sum, d3n_sum, a3p_sum, c3p_sum, a1p_sum, c1n_sum, d1p_sum, a2p_sum, b2p_sum, b3n_sum, c1p_sum, c2n_sum, a2n_sum, b3p_sum, c2p_sum, a3n_sum, c3n_sum, d1n_sum, d2n_sum, a1n_sum | | | | |

| **ANOVA^a^** | | | | | | |
| --- | --- | --- | --- | --- | --- | --- |
| Model | | Sum of Squares | df | Mean Square | F | Sig. |
| 1 | Regression | 7925.987 | 22 | 360.272 | 9.904 | .000^b^ |
|  | Residual | 35036.582 | 963 | 36.375 |  |  |
|  | Total | 42962.569 | 985 |  |  |  |
| a. Dependent Variable: Depression | | | | | | |
| b. Predictors: (Constant), d3p_sum, b2n_sum, d2p_sum, d3n_sum, a3p_sum, c3p_sum, a1p_sum, c1n_sum, d1p_sum, a2p_sum, b2p_sum, b3n_sum, c1p_sum, c2n_sum, a2n_sum, b3p_sum, c2p_sum, a3n_sum, c3n_sum, d1n_sum, d2n_sum, a1n_sum | | | | | | |

| **Coefficients^a^** | | | | | | |
| --- | --- | --- | --- | --- | --- | --- |
| Model | | Unstandardized Coefficients | | Standardized Coefficients | t | Sig. |
|  |  | B | Std. Error | Beta |  |  |
| 1 | (Constant) | 9.288 | .499 |  | 18.612 | .000 |
|  | a1n_sum | .775 | .259 | .124 | 2.991 | .003 |
|  | a2n_sum | -.039 | .244 | -.006 | -.159 | .874 |
|  | a3n_sum | .193 | .240 | .031 | .803 | .422 |
|  | b2n_sum | .406 | .211 | .069 | 1.923 | .055 |
|  | b3n_sum | .354 | .230 | .059 | 1.536 | .125 |
|  | c1n_sum | -.225 | .236 | -.036 | -.957 | .339 |
|  | c2n_sum | 1.374 | .219 | .236 | 6.274 | .000 |
|  | c3n_sum | .710 | .214 | .127 | 3.313 | .001 |
|  | d1n_sum | .164 | .232 | .027 | .708 | .479 |
|  | d2n_sum | .214 | .247 | .035 | .867 | .386 |
|  | d3n_sum | -.488 | .207 | -.082 | -2.354 | .019 |
|  | a1p_sum | -.567 | .230 | -.085 | -2.463 | .014 |
|  | a2p_sum | .032 | .251 | .005 | .126 | .900 |
|  | a3p_sum | -.357 | .200 | -.059 | -1.780 | .075 |
|  | b2p_sum | -.191 | .232 | -.029 | -.826 | .409 |
|  | b3p_sum | .050 | .239 | .008 | .208 | .835 |
|  | c1p_sum | -.550 | .228 | -.088 | -2.409 | .016 |
|  | c2p_sum | .239 | .237 | .037 | 1.006 | .315 |
|  | c3p_sum | -.376 | .208 | -.063 | -1.805 | .071 |
|  | d1p_sum | -.050 | .228 | -.008 | -.219 | .827 |
|  | d2p_sum | -.676 | .207 | -.111 | -3.267 | .001 |
|  | d3p_sum | .452 | .221 | .072 | 2.049 | .041 |
| a. Dependent Variable: Depression | | | | | | |

| **Excluded Variables^a^** | | | | | | |
| --- | --- | --- | --- | --- | --- | --- |
| Model | | Beta In | t | Sig. | Partial Correlation | Collinearity Statistics |
|  |  |  |  |  |  | Tolerance |
| 1 | b1n_sum | .^b^ | . | . | . | .000 |
|  | b1p_sum | .^b^ | . | . | . | .000 |
| a. Dependent Variable: Depression | | | | | | |
| b. Predictors in the Model: (Constant), d3p_sum, b2n_sum, d2p_sum, d3n_sum, a3p_sum, c3p_sum, a1p_sum, c1n_sum, d1p_sum, a2p_sum, b2p_sum, b3n_sum, c1p_sum, c2n_sum, a2n_sum, b3p_sum, c2p_sum, a3n_sum, c3n_sum, d1n_sum, d2n_sum, a1n_sum | | | | | | |

**Supplementary Material Table 8.7**

**Regression**

**DV: Self-Rated Work Performance (IWPQ)**

**IV: Image-based measures (summed by motive)**

| **Notes** | | |
| --- | --- | --- |
| Output Created | | 11-AUG-2023 10:19:04 |
| Comments | |  |
| Input | Data | C:\Users\jpincus\Desktop\Usefuls\LIS\LIS Perspectives\Workforce Listening Surveys\Wave 6\WFL6.merged.sav |
|  | Active Dataset | DataSet2 |
|  | Filter | <none> |
|  | Weight | weight1 |
|  | Split File | <none> |
|  | N of Rows in Working Data File | 976 |
| Missing Value Handling | Definition of Missing | User-defined missing values are treated as missing. |
|  | Cases Used | Statistics are based on cases with no missing values for any variable used. |
| Syntax | | REGRESSION  /MISSING LISTWISE  /STATISTICS COEFF OUTS R ANOVA  /CRITERIA=PIN(.05) POUT(.10)  /NOORIGIN  /DEPENDENT IWPQ  /METHOD=ENTER a1n_sum a2n_sum a3n_sum b1n_sum b2n_sum b3n_sum c1n_sum c2n_sum c3n_sum d1n_sum  d2n_sum d3n_sum a1p_sum a2p_sum a3p_sum b1p_sum b2p_sum b3p_sum c1p_sum c2p_sum c3p_sum d1p_sum  d2p_sum d3p_sum. |
| Resources | Processor Time | 00:00:00.08 |
|  | Elapsed Time | 00:00:00.08 |
|  | Memory Required | 61072 bytes |
|  | Additional Memory Required for Residual Plots | 0 bytes |

| **Variables Entered/Removed^a^** | | | |
| --- | --- | --- | --- |
| Model | Variables Entered | Variables Removed | Method |
| 1 | d3p_sum, b2n_sum, d2p_sum, d3n_sum, a3p_sum, c3p_sum, a1p_sum, c1n_sum, d1p_sum, a2p_sum, b2p_sum, b3n_sum, c1p_sum, c2n_sum, a2n_sum, b3p_sum, c2p_sum, a3n_sum, c3n_sum, d1n_sum, d2n_sum, a1n_sum^b^ | . | Enter |
| a. Dependent Variable: IWPQ | | | |
| b. Tolerance = .000 limit reached. | | | |

| **Model Summary** | | | | |
| --- | --- | --- | --- | --- |
| Model | R | R Square | Adjusted R Square | Std. Error of the Estimate |
| 1 | .223^a^ | .050 | .028 | .71919 |
| a. Predictors: (Constant), d3p_sum, b2n_sum, d2p_sum, d3n_sum, a3p_sum, c3p_sum, a1p_sum, c1n_sum, d1p_sum, a2p_sum, b2p_sum, b3n_sum, c1p_sum, c2n_sum, a2n_sum, b3p_sum, c2p_sum, a3n_sum, c3n_sum, d1n_sum, d2n_sum, a1n_sum | | | | |

| **ANOVA^a^** | | | | | | |
| --- | --- | --- | --- | --- | --- | --- |
| Model | | Sum of Squares | df | Mean Square | F | Sig. |
| 1 | Regression | 26.154 | 22 | 1.189 | 2.298 | .001^b^ |
|  | Residual | 498.208 | 963 | .517 |  |  |
|  | Total | 524.362 | 985 |  |  |  |
| a. Dependent Variable: IWPQ | | | | | | |
| b. Predictors: (Constant), d3p_sum, b2n_sum, d2p_sum, d3n_sum, a3p_sum, c3p_sum, a1p_sum, c1n_sum, d1p_sum, a2p_sum, b2p_sum, b3n_sum, c1p_sum, c2n_sum, a2n_sum, b3p_sum, c2p_sum, a3n_sum, c3n_sum, d1n_sum, d2n_sum, a1n_sum | | | | | | |

| **Coefficients^a^** | | | | | | |
| --- | --- | --- | --- | --- | --- | --- |
| Model | | Unstandardized Coefficients | | Standardized Coefficients | t | Sig. |
|  |  | B | Std. Error | Beta |  |  |
| 1 | (Constant) | 2.883 | .060 |  | 48.447 | .000 |
|  | a1n_sum | -.008 | .031 | -.011 | -.247 | .805 |
|  | a2n_sum | -.069 | .029 | -.093 | -2.360 | .018 |
|  | a3n_sum | .022 | .029 | .031 | .755 | .450 |
|  | b2n_sum | -.009 | .025 | -.014 | -.354 | .723 |
|  | b3n_sum | .031 | .027 | .047 | 1.131 | .258 |
|  | c1n_sum | -.059 | .028 | -.087 | -2.111 | .035 |
|  | c2n_sum | .015 | .026 | .023 | .560 | .575 |
|  | c3n_sum | -.056 | .026 | -.090 | -2.191 | .029 |
|  | d1n_sum | .002 | .028 | .003 | .071 | .943 |
|  | d2n_sum | -.020 | .029 | -.030 | -.694 | .488 |
|  | d3n_sum | .049 | .025 | .074 | 1.976 | .048 |
|  | a1p_sum | .039 | .027 | .053 | 1.431 | .153 |
|  | a2p_sum | -.018 | .030 | -.024 | -.613 | .540 |
|  | a3p_sum | .058 | .024 | .087 | 2.432 | .015 |
|  | b2p_sum | -.049 | .028 | -.067 | -1.757 | .079 |
|  | b3p_sum | .005 | .029 | .007 | .186 | .853 |
|  | c1p_sum | .038 | .027 | .055 | 1.400 | .162 |
|  | c2p_sum | -.051 | .028 | -.071 | -1.802 | .072 |
|  | c3p_sum | .023 | .025 | .035 | .925 | .355 |
|  | d1p_sum | .038 | .027 | .054 | 1.398 | .163 |
|  | d2p_sum | .032 | .025 | .048 | 1.301 | .194 |
|  | d3p_sum | -.018 | .026 | -.026 | -.678 | .498 |
| a. Dependent Variable: IWPQ | | | | | | |

| **Excluded Variables^a^** | | | | | | |
| --- | --- | --- | --- | --- | --- | --- |
| Model | | Beta In | t | Sig. | Partial Correlation | Collinearity Statistics |
|  |  |  |  |  |  | Tolerance |
| 1 | b1n_sum | .^b^ | . | . | . | .000 |
|  | b1p_sum | .^b^ | . | . | . | .000 |
| a. Dependent Variable: IWPQ | | | | | | |
| b. Predictors in the Model: (Constant), d3p_sum, b2n_sum, d2p_sum, d3n_sum, a3p_sum, c3p_sum, a1p_sum, c1n_sum, d1p_sum, a2p_sum, b2p_sum, b3n_sum, c1p_sum, c2n_sum, a2n_sum, b3p_sum, c2p_sum, a3n_sum, c3n_sum, d1n_sum, d2n_sum, a1n_sum | | | | | | |
