# Supplementary material for: The structure of human motivation
Source: BMC Psychol. 2023 Oct 6;11:308. doi: 10.1186/s40359-023-01346-5 (PMC10557177; doi:10.1186/s40359-023-01346-5)
Supplement: Supplementary file 3 — Additional file 3: SM Table 8. Regression models: Image-based measures to predict subject characteristics: Full output. [file 40359_2023_1346_MOESM3_ESM.zip › Tables.8.31.2023R5.docx]

Table 1. A unified model of human motivation (Pincus, 2023a).

| Three Levels of Attainment | Four Life Domains | | | |
| --- | --- | --- | --- | --- |
|  | Self (A) | Material (B) | Social (C) | Spiritual (D) |
| Aspirational (3) (*Having)* | Fulfilling Potential (A3P) & Failure to Thrive (A3N) | Success (B3P) & Failure (B3N) | Recognition (C3P) & Scorn (C3N) | Higher Purpose (D3P) & Materialism (D3N) |
| Experiential (2) (*Doing*) | Authenticity (A2P) & Conformity (A2N) | Immersion (B2P) & Boredom (B2N) | Caring (C2P) & Uncaring (C2N) | Ethics (D2P) & Wrongdoing (D2N) |
| Foundational (1) (*Being*) | Safety (A1P) & Anxiety (A1N) | Autonomy (B1P) & Disempowerment (B1N) | Inclusion (C1P) & Exclusion (C1N) | Justice (D1P) & Injustice (D1N) |

Table 2. Sample Characteristics.

|  | Bureau of Labor Statistics^1^ | March 2021 | May 2022 | June 2023 |
| --- | --- | --- | --- | --- |
| Sample size | 60,000 | 932 | 810 | 986 |
| Margin of error at 95% CI | +/-<1% | +/-3% | +/-3% | +/-3% |
| Response rate | 71% | 35% | 38% | 26% |
|  |  |  |  |  |
| Sex |  |  |  |  |
| Male | 56.3% | 57.0% | 55.4% | 56.6% |
| Female | 43.7% | 43.0% | 44.6% | 43.4% |
| Age |  |  |  |  |
| 18-19 | 1.0% | 0.4% | 1.0% | 0.8% |
| 20-24 | 7.2% | 4.7% | 7.0% | 7.4% |
| 25-34 | 25.1% | 23.9% | 25.6% | 23.9% |
| 35-44 | 24.4% | 39.8% | 24.9% | 24.4% |
| 45-54 | 23.4% | 19.0% | 23.4% | 23.8% |
| 55-64 | 19.0% | 12.0% | 18.1% | 19.6% |
| Race |  |  |  |  |
| Asian (non-Hispanic) | 6.6% | 6.4% | 6.9% | 6.9% |
| Black (non-Hispanic) | 11.8% | 12.3% | 12.9% | 12.9% |
| Hispanic/Latino | 18.5% | 13.6% | 17.8% | 19.5% |
| White (non-Hispanic) | 63.0% | 70.6% | 70.2% | 68.5% |

^1^U. S. Bureau of Labor Statistics (2023). Labor Force Statistics from the Current Population Survey. Demographic characteristics of U.S. workers, employed, usually work full-time, by age, sex, and race.

Table 3. Coefficient alpha values using KR20 for life domains and cells of the model.

|  | **Self**  **0.93** | **Material**  **0.93** | **Social 0.93** | **Spiritual 0.93** |
| --- | --- | --- | --- | --- |
| **Aspirational** | Fulfilling Potential & Failure to Thrive  0.82 | Success &  Failure  0.81 | Recognition &  Scorn  0.83 | Higher Purpose & Materialism  0.81 |
| **Experiential** | Authenticity & Conformity  0.81 | Immersion & Boredom  0.80 | Caring &  Uncaring  0.83 | Ethics &  Wrongdoing  0.83 |
| **Foundational** | Safety &  Anxiety  0.81 | Autonomy & Disempowerment  0.82 | Inclusion &  Exclusion  0.80 | Justice &  Injustice  0.82 |

Table 4. 14-Month test-retest correlations (n = 60).

| Domains | *r* | *p* |
| --- | --- | --- |
| Overall Positive | 0.75 | <.01 |
| Overall Negative | 0.67 | <.01 |
| Self | 0.72 | <.01 |
| Material | 0.71 | <.01 |
| Social | 0.78 | <.01 |
| Spiritual | 0.67 | <.01 |

Table 5. Summary of regression models: Image-based and established measures as predictors of *burnout* and *intent to quit*.

| Dependent variable: Burnout | R | R-squared | Cohen’s f | Effect size | Dependent variable: Intent to Quit | R | R-squared | Cohen’s f | Effect size |
| --- | --- | --- | --- | --- | --- | --- | --- | --- | --- |
|  |  |  |  |  |  |  |  |  |  |
| *Independent variables* |  |  |  |  | *Independent variables* |  |  |  |  |
| Image-based method | 0.36 | 0.13 | 0.386 | L | Image-based method | 0.35 | 0.12 | 0.374 | L |
| BFI-15 | 0.24 | 0.06 | 0.247 | M | BFI-15 | 0.16 | 0.03 *ns* | 0.162 | M |
| Image-based method + BFI-15 | 0.41 | 0.17 | 0.450 | L | Image-based method + BFI-15 | 0.38 | 0.14 | 0.411 | L |
|  |  |  |  |  |  |  |  |  |  |
| Image-based method | 0.36 | 0.13 | 0.386 | L | Image-based method | 0.35 | 0.12 | 0.374 | L |
| PSS-10 | 0.10 | 0.01 *ns* | 0.101 | S | PSS-10 | 0.19 | 0.04 | 0.194 | M |
| Image-based method + PSS-10 | 0.41 | 0.17 | 0.450 | L | Image-based method + PSS-10 | 0.37 | 0.14 | 0.398 | L |
|  |  |  |  |  |  |  |  |  |  |
| Image-based method | 0.36 | 0.13 | 0.386 | L | Image-based method | 0.35 | 0.12 | 0.374 | L |
| Brief COPE-28 | 0.22 | 0.05 | 0.226 | M | Brief COPE-28 | 0.16 | 0.03 *ns* | 0.162 | M |
| Image-based method + Brief COPE-28 | 0.41 | 0.17 | 0.450 | L | Image-based method + Brief COPE-28 | 0.39 | 0.15 | 0.424 | L |
|  |  |  |  |  |  |  |  |  |  |
| Image-based method | 0.36 | 0.13 | 0.386 | L | Image-based method | 0.35 | 0.12 | 0.374 | L |
| RVS-18 | 0.17 | 0.03 *ns* | 0.173 | M | RVS-18 | 0.16 | 0.03 *ns* | 0.162 | M |
| Image-based method + RVS-18 | 0.39 | 0.15 | 0.424 | L | Image-based method + RVS-18 | 0.38 | 0.14 | 0.411 | L |
|  |  |  |  |  |  |  |  |  |  |
| Image-based method | 0.36 | 0.13 | 0.386 | L | Image-based method | 0.35 | 0.12 | 0.374 | L |
| BFI-15 | 0.24 | 0.06 | .0247 | S | BFI-15 | 0.16 | 0.03 | 0.162 | M |
| PSS-10 | 0.10 | 0.01 *ns* | 0.101 | S | PSS-10 | 0.19 | 0.04 | 0.194 | M |
| Brief COPE-28 | 0.22 | 0.05 | 0.226 | M | Brief COPE-28 | 0.16 | 0.03 *ns* | 0.162 | M |
| RVS-18 | 0.17 | 0.03 *ns* | 0.173 | M | RVS-18 | 0.17 | 0.03 *ns* | 0.173 | M |
| Image-based method + BFI-15 + PSS-10 + Brief COPE-28 + RVS-18 | 0.44 | 0.20 | 0.493 | L | Image-based method + BFI-15 + PSS-10 + Brief COPE-28 + RVS-18 | 0.44 | 0.20 | .493 | L |
|  |  |  |  |  |  |  |  |  |  |

Note: all models are significant at <0.01 unless noted *ns* (non-significant)

Cohen’s f interpretation of effect sizes: S (small) = .01-.14; M (medium) = .150-.34; L (large) = .35 or larger

Cohen J. E. (1988). Statistical Power Analysis for the Behavioral Sciences. Hillsdale, NJ: Lawrence Erlbaum Associates, Inc

Table 6. Discriminant validity of image-based method vs. BFI, RVS, and social support items of B-COPE (November 2021).

| Category | Measure | M (SD) | A1 | A2 | A3 | B1 | B2 | B3 | C1 | C2 | C3 | D1 | D2 | D3 |
| --- | --- | --- | --- | --- | --- | --- | --- | --- | --- | --- | --- | --- | --- | --- |
| Personality trait | Conscientiousness | 3.82 (0.71) | 0.063 | -0.058 | .108** | 0.057 | -0.006 | .102** | 0.062 | 0.03 | .084* | 0.073 | 0.035 | 0.018 |
| Personality trait | Extraversion | 3.15 (0.78) | -0.071 | -.078* | -0.065 | -.078* | -0.033 | -0.019 | 0.024 | -0.02 | 0.028 | -0.042 | -0.008 | -0.054 |
| Personality trait | Neuroticism | 2.98 (0.85) | 0.026 | 0.068 | 0.041 | 0.029 | .137** | 0.01 | 0.033 | 0.072 | 0.067 | -0.002 | 0.047 | 0.025 |
| Personality trait | Openness | 3.84 (0.76) | -0.006 | .115** | -0.035 | 0.039 | 0.007 | 0.05 | 0.016 | 0.042 | 0.04 | 0.004 | 0.046 | 0.031 |
| Personality trait | Agreeableness | 3.77 (0.75) | 0.07 | 0.021 | 0.044 | 0.056 | 0.016 | .124** | .095* | 0.05 | 0.062 | 0.061 | 0.003 | .077* |
| Terminal value | True Friendship | 4.50 (3.57) | 0.038 | 0.042 | 0.061 | 0.065 | -0.022 | 0.069 | -0.011 | -0.003 | 0.01 | 0.013 | -0.062 | 0.021 |
| Terminal value | Mature Love | 4.69 (3.63) | -0.011 | -0.031 | 0.036 | 0.023 | 0.041 | 0.029 | 0.025 | 0.012 | 0.039 | 0.012 | -0.042 | 0.019 |
| Terminal value | Self-respect | 4.20 (2.89) | -0.071 | -0.058 | -0.01 | 0.025 | -.121** | 0.012 | 0.013 | -0.047 | 0.026 | -0.025 | -.131** | -0.053 |
| Terminal value | Happiness | 4.13 (2.69) | 0.004 | 0.014 | -0.018 | -0.011 | 0.016 | 0.002 | -0.041 | -0.049 | 0.034 | -0.053 | 0.005 | 0.008 |
| Terminal value | Inner Harmony | 6.13 (2.97) | -0.016 | -.097* | -0.013 | 0.016 | -.081* | -0.05 | -0.016 | -0.024 | -0.03 | -0.044 | -0.032 | -0.035 |
| Terminal value | Equality | 7.03 (3.04) | .138** | .086* | **.182**** | .125** | 0.057 | .104** | .095* | 0.063 | 0.073 | 0.017 | 0.051 | 0.041 |
| Terminal value | Freedom | 7.15 (3.18) | 0.01 | 0.042 | .084* | 0.027 | -0.043 | .089* | 0.005 | -0.022 | 0.014 | 0.044 | -0.025 | 0.03 |
| Terminal value | Pleasure | 8.99 (3.11) | 0.022 | 0.035 | 0.043 | 0.023 | -0.02 | 0.045 | -0.008 | -0.036 | -0.029 | 0.027 | -0.029 | 0.021 |
| Terminal value | Social Recognition | 11.00 (3.58) | 0.035 | -0.063 | 0.042 | .082* | -0.001 | -0.053 | 0.016 | 0.016 | -0.048 | -0.035 | -0.03 | -0.028 |
| Terminal value | Wisdom | 9.71 (3.39) | 0.063 | 0.049 | 0.027 | 0.048 | 0.066 | 0.034 | 0.029 | 0.038 | 0.044 | 0.058 | 0.027 | 0.043 |
| Terminal value | Salvation | 11.86 (3.63) | 0.034 | 0.032 | 0.022 | 0.043 | 0.031 | 0.051 | 0.066 | 0.038 | 0.019 | .091* | -0.033 | 0.068 |
| Terminal value | Family Security | 9.16 (4.76) | -0.041 | -0.013 | -0.052 | -0.041 | 0.016 | -0.069 | -0.002 | -0.006 | 0.002 | -0.012 | 0.067 | -0.036 |
| Terminal value | National Security | 13.12 (3.27) | 0.056 | -0.023 | 0.039 | 0.032 | -0.025 | 0.03 | .094* | 0.002 | 0.009 | 0.019 | 0.045 | 0.002 |
| Terminal value | A Sense of Accomplishment | 12.59 (3.59) | 0.019 | -0.028 | -0.04 | -0.068 | 0.047 | -0.074 | -0.011 | 0.046 | 0.004 | -0.028 | 0.026 | -0.006 |
| Terminal value | A World of Beauty | 14.87 (2.53) | 0.06 | 0.07 | 0.046 | 0.07 | 0.061 | 0.068 | .078* | .080* | .077* | 0.073 | .129** | 0.074 |
| Terminal value | A World at Peace | 14.26 (3.90) | 0 | 0.045 | -0.056 | -0.037 | 0.021 | 0.035 | -0.062 | 0.003 | -0.006 | -0.011 | 0.024 | -0.026 |
| Terminal value | A Comfortable Life | 12.46 (5.61) | -.092* | 0.01 | -.134** | -.104** | 0.018 | -.108** | -0.074 | 0.022 | -0.034 | -0.036 | 0.058 | -0.023 |
| Terminal value | An Exciting Life | 15.15 (4.18) | -.125** | -.084* | -.093* | -.162** | -.078* | -0.072 | -.095* | -.121** | -.122** | -0.064 | -.078* | -0.062 |
| Coping style | Social Support | 9.84 (3.36) | -0.025 | -0.001 | -.143** | -.087* | 0.039 | -.134** | -0.056 | -0.013 | -0.069 | -.112** | -0.031 | -0.067 |

** Correlation is significant at the 0.01 level (2-tailed).

* Correlation is significant at the 0.05 level (2-tailed).

Table 7. Discriminant validity of image-based method vs. BFI, RVS, and social support items of B-COPE (March 2022).

| Category | Measure | M (SD) | A1 | A2 | A3 | B1 | B2 | B3 | C1 | C2 | C3 | D1 | D2 | D3 |
| --- | --- | --- | --- | --- | --- | --- | --- | --- | --- | --- | --- | --- | --- | --- |
| Personality trait | Conscientiousness | 3.85 (0.77) | 0.041 | -0.009 | .115** | -0.014 | .072* | -0.014 | 0.064 | 0.058 | .092** | 0.02 | 0.043 | 0.038 |
| Personality trait | Extraversion | 3.14 (0.84) | -0.041 | 0.067 | -0.029 | -0.02 | -0.013 | -0.02 | 0.025 | 0.036 | 0.019 | 0 | 0.023 | -0.013 |
| Personality trait | Neuroticism | 2.86 (0.99) | -0.021 | -.074* | -0.016 | -0.031 | -0.023 | -0.031 | -.099** | -0.067 | -.083* | -0.012 | -0.056 | -0.043 |
| Personality trait | Openness | 3.84 (0.76) | 0.003 | 0.069 | 0.013 | -0.007 | 0.058 | -0.007 | 0.057 | -0.017 | 0.002 | -0.002 | 0.011 | 0.027 |
| Personality trait | Agreeableness | 3.77 (0.75) | 0.057 | 0.013 | .114** | 0.033 | .105** | 0.033 | .134** | .104** | .095** | .105** | .077* | .080* |
| Terminal value | True Friendship | 3.65 (3.22) | 0.031 | -.076* | .095** | -0.011 | -0.036 | -0.011 | -0.051 | 0.049 | -0.037 | 0.025 | -0.04 | -0.039 |
| Terminal value | Mature Love | 4.38 (3.47) | .081* | 0.013 | .117** | 0.066 | 0.028 | 0.066 | 0.015 | .111** | 0.049 | 0.058 | 0.029 | -0.021 |
| Terminal value | Self-respect | 3.87 (2.43) | -0.031 | -.085* | 0.008 | -0.05 | -0.009 | -0.05 | -0.045 | -0.025 | -0.013 | -0.06 | -0.059 | 0.011 |
| Terminal value | Happiness | 4.32 (2.40) | -0.057 | -0.056 | 0.008 | -0.061 | -0.012 | -0.061 | -0.016 | 0.031 | -0.02 | -0.05 | 0 | -0.028 |
| Terminal value | Inner Harmony | 6.08 (2.69) | -0.037 | -0.028 | -0.042 | -0.03 | -0.018 | -0.03 | 0.008 | -0.03 | -0.026 | -0.002 | -0.059 | -0.021 |
| Terminal value | Equality | 7.58 (3.35) | 0.013 | -.121** | .085* | -0.004 | -0.035 | -0.004 | -0.06 | 0.038 | -0.015 | -0.044 | -0.035 | -0.056 |
| Terminal value | Freedom | 7.06 (2.91) | 0.058 | 0.052 | 0.019 | 0.001 | 0.014 | 0.001 | 0.062 | .101** | 0.025 | .090* | 0.059 | 0.018 |
| Terminal value | Pleasure | 8.94 (2.89) | 0.032 | -0.032 | .098** | 0.033 | 0.042 | 0.033 | 0.029 | .074* | 0.018 | -0.017 | 0.011 | 0.026 |
| Terminal value | Social Recognition | 11.16 (3.76) | .086* | -.072* | .133** | 0.056 | 0.021 | 0.056 | -0.009 | **.148**** | 0.034 | 0.02 | 0.018 | 0.029 |
| Terminal value | Wisdom | 9.23 (3.32) | 0.012 | 0.027 | -0.042 | 0.024 | 0 | 0.024 | 0.041 | -0.03 | 0.021 | 0.039 | -0.007 | 0.001 |
| Terminal value | Salvation | 10.95 (3.96) | -0.018 | -0.044 | -0.043 | -0.026 | -0.042 | -0.026 | -0.012 | -0.06 | 0.013 | 0.03 | -0.049 | -0.002 |
| Terminal value | Family Security | 9.46 (4.45) | -.081* | 0.05 | -.107** | -0.004 | -0.049 | -0.004 | -0.057 | -.139** | -.070* | -.076* | -0.04 | -0.057 |
| Terminal value | National Security | 13.17 (2.77) | -0.016 | 0.008 | 0.029 | -0.002 | -0.009 | -0.002 | -0.004 | -0.034 | -0.035 | 0.006 | -0.035 | 0.016 |
| Terminal value | A Sense of Accomplishment | 12.87 (3.28) | 0.021 | 0.058 | -0.054 | -0.021 | .089* | -0.021 | 0.046 | -0.005 | 0.016 | 0.022 | 0.063 | 0.064 |
| Terminal value | A World of Beauty | 14.71 (2.48) | -0.047 | 0.003 | -0.021 | -0.022 | -0.004 | -0.022 | -0.06 | -0.045 | 0.02 | -0.047 | -0.064 | -0.012 |
| Terminal value | A World at Peace | 14.02 (4.12) | -0.033 | 0.046 | -.073* | 0.028 | -0.025 | 0.028 | -0.008 | -0.067 | 0.011 | -0.057 | 0.002 | -0.026 |
| Terminal value | A Comfortable Life | 13.64 (4.94) | 0.004 | .111** | -0.057 | 0.011 | 0.042 | 0.011 | .073* | -0.025 | 0.025 | 0.026 | .107** | 0.037 |
| Terminal value | An Exciting Life | 15.9 (3.57) | -0.027 | 0.038 | -0.067 | -0.04 | 0.005 | -0.04 | 0.014 | -0.025 | -0.026 | 0.02 | 0.023 | 0.056 |
| Coping style | Social Support | 9.27 (3.19) | -0.033 | 0.038 | -0.035 | -0.052 | 0.019 | -0.052 | 0.015 | -0.006 | -0.043 | -0.014 | 0.004 | 0.006 |

** Correlation is significant at the 0.01 level (2-tailed).* Correlation is significant at the 0.05 level (2-tailed).

Table 8. Summary of regression analyses employing image selection data to predict subject characteristics.

| **Hypothesis** | **ANOVA** | **Significant predictors (linear regression)** |
| --- | --- | --- |
| CV-1. Openness to Experience 🡪 Authenticity (A2P) * & Immersion (B2P) † | F = 2.684, 22 df, p = .000 | Authenticity (A2P; β = 2.096, p = 0.036) *  Success (B3P; β = 2.644, p = 0.008)  Exclusion (C1N; β = -2.266, p = 0.024)  Wrongdoing (D2N; β = 2.154, p = 0.032) |
| CV-2. Extraversion 🡪 Inclusion (C1P) * | F = 3.231, 22 df, p = .000 | Inclusion (C1P; β = 3.428, p = 0.001) *  Recognition (C3P; β = 4.044, p = 0.000)  Uncaring (C2N; β = -3.910, p = 0.000) Anxiety (A1N; β = 2.379, p = 0.018) |
| CV-3. Agreeableness 🡪 Caring (C2P) * & Ethics (D2P) * | F = 1.716, 22 df, p = .021 | Ethics (D2P; β = 2.561, p = 0.011) * Caring (C2P; (β = -2.097, p = 0.036) *  Materialism (D3N; β = 2.860, p = 0.004) |
| CV-4. Conscientiousness 🡪 Potential (A3P) * | F = 2.451, 22 df, p = .000 | Potential (A3P; β = 2.583, p = 0.010) *  Limitation (A3N; β = 1.988, p = 0.047)  Stagnation (B2N; β = -1.968, p = 0.049) Inclusion (C1P; β = 2.420, p = 0.016) Recognition (C3P; β = 2.223, p = 0.026) Purpose (D3P; β = -2.174, p = 0.030) |
| CV-5. Neuroticism 🡪 Anxiety (A1N) † & Uncaring (C2N) * | F = 5.237, 22 df, p = .000 | Uncaring (C2N; β = 4.759, p = 0.000) *  Caring (C2P; β = 2.875, p = 0.004)  Inclusion (C1P; β = -2.044, p = 0.041)  Recognition (C3P; β = -2.010, p = 0.045) Ethics (D2P; β = -2.868, p = 0.004)  Stagnation (B2N; β = 2.370, p = 0.018)  Scorn (C3N; β = 3.404, p = 0.001)  Materialism (D3N; β = -2.021, p = 0.044) |
| CV-6. Depression 🡪 Anxiety (A1N) * & Uncaring (C2N) * | F = 9.904, 22 df, p = .000 | Anxiety (A1N; β = 2.991, p = 0.003) *  Uncaring (C2N; β = 6.274, p = 0.000) *  Scorn (C3N; β = 3.313, p = 0.001)  Materialism (D3N; β = -2.354, p = 0.019) Safety (A1P; β = -2.463, p = 0.014)  Inclusion (C1P; β = -2.409, p = 0.016)  Ethics (D2P; β = -3.267, p = 0.001)  Purpose (D3P; β = 2.049, p = 0.041) |
| CV-7. Work Performance 🡪 Potential (A3P) * | F = 2.298, 22 df, p = .001 | Potential (A3P; β = 2.432, p = 0.015) * Conformity (A2N; β = -2.360, p = 0.018) Exclusion (C1N; β = -2.111, p = 0.035)  Scorn (C3N; β = -2.191, p = 0.029)  Materialism (D3N; β = 1.976, p = 0.048) |

* = Findings support hypothesis; † = Findings do not support hypothesis

Table 9. Confirmatory factor models for the four life domains.

|  | Promotion motivation | | | | | Prevention motivation | | | | |
| --- | --- | --- | --- | --- | --- | --- | --- | --- | --- | --- |
|  | Overall | Self | Material | Social | Spiritual | Overall | Self | Material | Social | Spiritual |
| Chi-square | 1946.318, 588 *df*,  p < .001 | 66.375, 24 *df*,  p < .001 | 41.291, 24 *df*,  p = 0.015 | 30.063, 24 *df*,  p = 0.026 | 50.010, *df* 24,  p < 0.001 | 1338.450, 588 *df*,  p < .001 | 40.735, 24 *df*,  p < .001 | 31.052, 24 *df*,  p = 0.152 | 28.684, 24 *df*,  p = 0.232 | 35.711, 24 *df*,  p = 0.059 |
| TLI | 0.900 | 0.863 | 0.935 | 0.962 | 0.938 | 0.950 | 0.929 | 0.987 | 0.995 | 0.981 |
| RMSEA | 0.047 | 0.041 | 0.026 | 0.027 | 0.032 | 0.035 | 0.037 | 0.017 | 0.014 | 0.022 |
| SRMR | 0.040 | 0.036 | 0.027 | 0.024 | 0.031 | 0.027 | 0.033 | 0.021 | 0.019 | 0.023 |
| Minimum acceptance fit thresholds exceeded | 3 | 2 | 3 | 3 | 3 | 3 | 3 | 4 | 4 | 4 |

Table 10. Confirmatory factor models for the three levels of attainment.

|  | Promotion motivation | | | | Prevention motivation | | | |
| --- | --- | --- | --- | --- | --- | --- | --- | --- |
|  | Overall | Foundational | Experiential | Aspirational | Overall | Foundational | Experiential | Aspirational |
| Chi-square | 1818.986, 557 *df*,  p < .001 | 87.709, 50 *df*,  p < .001 | 120.171, 50 *df*, p < 0.001 | 83.479, 50 *df*,  p < .001 | 1330.930, 557 *df*,  p < .001 | 74.505, 50 *df*,  p = .014 | 97.702, 50 *df*,  p < .001 | 100.733, 50 *df*,  p < .001 |
| TLI | 0.900 | 0.925 | 0.889 | 0.926 | 0.950 | 0.976 | 0.946 | 0.952 |
| RMSEA | 0.047 | 0.027 | 0.037 | 0.032 | 0.037 | 0.022 | 0.030 | 0.031 |
| SRMR | 0.040 | 0.030 | 0.036 | 0.032 | 0.028 | 0.026 | 0.036 | 0.032 |
| Minimum acceptance fit thresholds exceeded | 3 | 3 | 2 | 3 | 3 | 3 | 3 | 3 |

Table 11. Confirmatory factor models for adjacent and antipodal* life domains.

|  | Promotion motivation | | | | | | Prevention motivation | | | | | |
| --- | --- | --- | --- | --- | --- | --- | --- | --- | --- | --- | --- | --- |
|  | Self-Material | Material-Social | Social-Spiritual | Spiritual-Self | Self-Social* | Material-Spiritual* | Self-Material | Material-Social | Social-Spiritual | Spiritual-Self | Self-Social* | Material-Spiritual* |
| Chi-square  (all 133 *df*,  p < 0.001) | 462.225 | 406.902 | 502.737 | 575.644 | 398.411 | 465.046 | 365.692 | 370.828 | 470.785 | 407.218 | 467.834 | 296.125 |
| TLI | 0.683 | 0.799 | 0.754 | 0.630 | 0.776 | 0.708 | 0.875 | 0.893 | 0.855 | 0.857 | 0.852 | 0.912 |
| RMSEA | 0.049 | 0.045 | 0.052 | 0.057 | 0.044 | 0.049 | 0.041 | 0.042 | 0.050 | 0.045 | 0.049 | 0.034 |
| SRMR | 0.049 | 0.045 | 0.049 | 0.054 | 0.046 | 0.047 | 0.040 | 0.039 | 0.046 | 0.041 | 0.044 | 0.035 |
| Minimum acceptance fit thresholds exceeded | 2 | 2 | 1 | 0 | 2 | 2 | 2 | 2 | 1 | 2 | 2 | 3 |

Table 12. Standardized canonical function coefficients for each construct pair.

| Set 1 | Set 2 | Canonical Correlation (all *p* < .000) | Average shared variance | Predicted (P), non-predicted (N) | Same domain (S), Adjacent (A), Antipodal (L) |
| --- | --- | --- | --- | --- | --- |
|  |  |  |  |  |  |
| Safety | Authenticity | 0.576 | 13.5% | P | S |
| Safety | Potential | 0.649 | 16.0% | P | S |
| Safety | Autonomy | 0.654 | 17.5% | P | A |
| Safety | Immersion | 0.569 | 13.0% | N | A |
| Safety | Success | 0.633 | 16.5% | N | A |
| Safety | Inclusion | 0.611 | 15.5% | N | L |
| Safety | Caring | 0.635 | 16.0% | N | L |
| Safety | Recognition | 0.627 | 16.5% | N | L |
| Safety | Justice | 0.633 | 17.5% | P | A |
| Safety | Ethics | 0.564 | 13.5% | N | A |
| Safety | Transcendence | 0.628 | 15.5% | N | A |
|  |  |  |  |  |  |
| Authenticity | Potential | 0.539 | 11.0% | P | S |
| Authenticity | Autonomy | 0.526 | 11.5% | N | A |
| Authenticity | Immersion | 0.484 | 9.5% | P | A |
| Authenticity | Success | 0.52 | 11.5% | N | A |
| Authenticity | Inclusion | 0.513 | 11.0% | N | L |
| Authenticity | Caring | 0.534 | 11.5% | N | L |
| Authenticity | Recognition | 0.501 | 10.5% | N | L |
| Authenticity | Justice | 0.532 | 11.5% | N | A |
| Authenticity | Ethics | 0.622 | 14.5% | P | A |
| Authenticity | Transcendence | 0.626 | 14.5% | N | A |
|  |  |  |  |  |  |
| Potential | Autonomy | 0.703 | 19.5% | N | A |
| Potential | Immersion | 0.628 | 15.0% | N | A |
| Potential | Success | 0.63 | 16.0% | P | A |
| Potential | Inclusion | 0.567 | 13.0% | N | L |
| Potential | Caring | 0.613 | 50.5% | N | L |
| Potential | Recognition | 0.594 | 52.5% | N | L |
| Potential | Justice | 0.562 | 41.0% | N | A |
| Potential | Ethics | 0.507 | 43.5% | N | A |
| Potential | Transcendence | 0.636 | 38.5% | P | A |
|  |  |  |  |  |  |
| Autonomy | Immersion | 0.684 | 33.5% | P | S |
| Autonomy | Success | 0.685 | 29.0% | P | S |
| Autonomy | Inclusion | 0.625 | 43.0% | P | A |
| Autonomy | Caring | 0.645 | 45.5% | N | A |
| Autonomy | Recognition | 0.65 | 44.5% | N | A |
| Autonomy | Justice | 0.582 | 33.0% | N | L |
| Autonomy | Ethics | 0.54 | 39.5% | N | L |
| Autonomy | Transcendence | 0.609 | 28.5% | N | L |
|  |  |  |  |  |  |
| Immersion | Success | 0.682 | 40.0% | P | S |
| Immersion | Inclusion | 0.643 | 29.5% | N | A |
| Immersion | Caring | 0.628 | 26.0% | P | A |
| Immersion | Recognition | 0.661 | 28.0% | N | A |
| Immersion | Justice | 0.543 | 26.5% | N | L |
| Immersion | Ethics | 0.564 | 27.0% | N | L |
| Immersion | Transcendence | 0.549 | 27.0% | N | L |
|  |  |  |  |  |  |
| Success | Inclusion | 0.64 | 38.5% | N | A |
| Success | Caring | 0.634 | 28.0% | N | A |
| Success | Recognition | 0.691 | 34.0% | P | A |
| Success | Justice | 0.564 | 30.5% | N | L |
| Success | Ethics | 0.572 | 27.0% | N | L |
| Success | Transcendence | 0.571 | 36.5% | N | L |
|  |  |  |  |  |  |
| Inclusion | Caring | 0.671 | 56.5% | P | S |
| Inclusion | Recognition | 0.686 | 55.5% | P | S |
| Inclusion | Justice | 0.612 | 45.0% | P | A |
| Inclusion | Ethics | 0.558 | 44.5% | N | A |
| Inclusion | Transcendence | 0.532 | 44.0% | N | A |
|  |  |  |  |  |  |
| Caring | Recognition | 0.698 | 60.0% | P | S |
| Caring | Justice | 0.595 | 42.0% | N | A |
| Caring | Ethics | 0.619 | 49.0% | P | A |
| Caring | Transcendence | 0.619 | 36.0% | N | A |
|  |  |  |  |  |  |
| Recognition | Justice | 0.605 | 39.5% | N | A |
| Recognition | Ethics | 0.641 | 44.5% | N | A |
| Recognition | Transcendence | 0.574 | 37.5% | P | A |
|  |  |  |  |  |  |
| Justice | Ethics | 0.571 | 54.0% | P | S |
| Justice | Transcendence | 0.573 | 42.5% | P | S |
|  |  |  |  |  |  |
| Ethics | Transcendence | 0.616 | 43.0% | P | S |

Table 13. Prevalence of predicted pairings by correlation strength bins.

| Correlation strength rank bins | % of observed correlations predicted by theoretical model |
| --- | --- |
| Top 10 | 80% |
| 11 to 20 | 20% |
| 21 to 30 | 50% |
| 31 to 40 | 20% |
| 41 to 50 | 10% |
| 51 to 66 | 0% |
