# Supplementary material for: The structure of human motivation
Source: BMC Psychol. 2023 Oct 6;11:308. doi: 10.1186/s40359-023-01346-5 (PMC10557177; doi:10.1186/s40359-023-01346-5)
Supplement: Supplementary file 4 — Additional file 4: SM Table 9. Confirmatory factor models for the four life domains: Full output. [file 40359_2023_1346_MOESM4_ESM.zip › Table 9.1 CFA.life.domains.PositiveR5.docx]

**Table 9.1 CFA-Domains-Promotion**

**Table 9.1.1 CFA Overall promotion motivation (“positive”)**

**Model fit**

| **Chi-square test** | | | | | | | |
| --- | --- | --- | --- | --- | --- | --- | --- |
| **Model** | | **Χ²** | | **df** | | **p** | |
| Baseline model |  | 14439.004 |  | 630 |  |  |  |
| Factor model |  | 1946.318 |  | 588 |  | < .001 |  |
|  | | | | | | | |

**Additional fit measures**

| **Fit indices** | | | |
| --- | --- | --- | --- |
| **Index** | | **Value** | |
| Comparative Fit Index (CFI) |  | 0.902 |  |
| Tucker-Lewis Index (TLI) |  | 0.895 |  |
| Bentler-Bonett Non-normed Fit Index (NNFI) |  | 0.895 |  |
| Bentler-Bonett Normed Fit Index (NFI) |  | 0.865 |  |
| Parsimony Normed Fit Index (PNFI) |  | 0.808 |  |
| Bollen's Relative Fit Index (RFI) |  | 0.856 |  |
| Bollen's Incremental Fit Index (IFI) |  | 0.902 |  |
| Relative Noncentrality Index (RNI) |  | 0.902 |  |
|  | | | |

| **Information criteria** | | | |
| --- | --- | --- | --- |
|  | | **Value** | |
| Log-likelihood |  | -266626.935 |  |
| Number of free parameters |  | 78.000 |  |
| Akaike (AIC) |  | 533409.870 |  |
| Bayesian (BIC) |  | 533795.057 |  |
| Sample-size adjusted Bayesian (SSABIC) |  | 533547.320 |  |
|  | | | |

| **Other fit measures** | | | |
| --- | --- | --- | --- |
| **Metric** | | **Value** | |
| Root mean square error of approximation (RMSEA) |  | 0.047 |  |
| RMSEA 90% CI lower bound |  | 0.045 |  |
| RMSEA 90% CI upper bound |  | 0.050 |  |
| RMSEA p-value |  | 0.970 |  |
| Standardized root mean square residual (SRMR) |  | 0.040 |  |
| Hoelter's critical N (α = .05) |  | 342.944 |  |
| Hoelter's critical N (α = .01) |  | 356.285 |  |
| Goodness of fit index (GFI) |  | 0.890 |  |
| McDonald fit index (MFI) |  | 0.518 |  |
| Expected cross validation index (ECVI) |  | 2.039 |  |
|  | | | |

| **R-Squared** | | | |
| --- | --- | --- | --- |
|  | | **R²** | |
| A1Px |  | 0.281 |  |
| A1Py |  | 0.390 |  |
| A1Pz |  | 0.257 |  |
| A2Px |  | 0.383 |  |
| A2Py |  | 0.392 |  |
| A2Pz |  | 0.337 |  |
| A3Px |  | 0.255 |  |
| A3Py |  | 0.241 |  |
| A3Pz |  | 0.222 |  |
| B1Px |  | 0.389 |  |
| B1Py |  | 0.390 |  |
| B1Pz |  | 0.352 |  |
| B2Px |  | 0.379 |  |
| B2Py |  | 0.403 |  |
| B2Pz |  | 0.404 |  |
| B3Px |  | 0.278 |  |
| B3Py |  | 0.270 |  |
| B3Pz |  | 0.297 |  |
| C1Px |  | 0.378 |  |
| C1Py |  | 0.386 |  |
| C1Pz |  | 0.414 |  |
| C2Px |  | 0.392 |  |
| C2Py |  | 0.361 |  |
| C2Pz |  | 0.432 |  |
| C3Px |  | 0.302 |  |
| C3Py |  | 0.400 |  |
| C3Pz |  | 0.379 |  |
| D1Px |  | 0.371 |  |
| D1Py |  | 0.412 |  |
| D1Pz |  | 0.299 |  |
| D2Px |  | 0.343 |  |
| D2Py |  | 0.398 |  |
| D2Pz |  | 0.395 |  |
| D3Px |  | 0.390 |  |
| D3Py |  | 0.340 |  |
| D3Pz |  | 0.355 |  |
|  | | | |

**Parameter estimates**

| **Factor loadings** | | | | | | | | | | | | | | | | | |
| --- | --- | --- | --- | --- | --- | --- | --- | --- | --- | --- | --- | --- | --- | --- | --- | --- | --- |
|  | | | | | | | | | | | | | | **95% Confidence Interval** | | | |
| **Factor** | | **Indicator** | | **Symbol** | | **Estimate** | | **Std. Error** | | **z-value** | | **p** | | **Lower** | | **Upper** | |
| Self |  | A1Px |  | λ11 |  | 204.953 |  | 11.569 |  | 17.715 |  | < .001 |  | 182.278 |  | 227.628 |  |
|  |  | A1Py |  | λ12 |  | 227.905 |  | 10.550 |  | 21.603 |  | < .001 |  | 207.228 |  | 248.582 |  |
|  |  | A1Pz |  | λ13 |  | 187.208 |  | 11.130 |  | 16.820 |  | < .001 |  | 165.393 |  | 209.023 |  |
|  |  | A2Px |  | λ14 |  | 222.083 |  | 10.401 |  | 21.353 |  | < .001 |  | 201.698 |  | 242.468 |  |
|  |  | A2Py |  | λ15 |  | 211.838 |  | 9.782 |  | 21.655 |  | < .001 |  | 192.665 |  | 231.011 |  |
|  |  | A2Pz |  | λ16 |  | 217.566 |  | 11.018 |  | 19.747 |  | < .001 |  | 195.971 |  | 239.161 |  |
|  |  | A3Px |  | λ17 |  | 197.385 |  | 11.791 |  | 16.741 |  | < .001 |  | 174.275 |  | 220.494 |  |
|  |  | A3Py |  | λ18 |  | 194.313 |  | 11.983 |  | 16.216 |  | < .001 |  | 170.827 |  | 217.799 |  |
|  |  | A3Pz |  | λ19 |  | 186.821 |  | 12.052 |  | 15.501 |  | < .001 |  | 163.199 |  | 210.443 |  |
| Material |  | B1Px |  | λ21 |  | 222.587 |  | 10.258 |  | 21.699 |  | < .001 |  | 202.482 |  | 242.692 |  |
|  |  | B1Py |  | λ22 |  | 221.268 |  | 10.188 |  | 21.718 |  | < .001 |  | 201.299 |  | 241.236 |  |
|  |  | B1Pz |  | λ23 |  | 220.833 |  | 10.827 |  | 20.396 |  | < .001 |  | 199.611 |  | 242.054 |  |
|  |  | B2Px |  | λ24 |  | 231.438 |  | 10.846 |  | 21.338 |  | < .001 |  | 210.180 |  | 252.696 |  |
|  |  | B2Py |  | λ25 |  | 237.415 |  | 10.710 |  | 22.168 |  | < .001 |  | 216.424 |  | 258.406 |  |
|  |  | B2Pz |  | λ26 |  | 242.553 |  | 10.917 |  | 22.217 |  | < .001 |  | 221.155 |  | 263.951 |  |
|  |  | B3Px |  | λ27 |  | 201.037 |  | 11.339 |  | 17.729 |  | < .001 |  | 178.812 |  | 223.261 |  |
|  |  | B3Py |  | λ28 |  | 198.380 |  | 11.391 |  | 17.415 |  | < .001 |  | 176.053 |  | 220.706 |  |
|  |  | B3Pz |  | λ29 |  | 208.727 |  | 11.324 |  | 18.433 |  | < .001 |  | 186.532 |  | 230.921 |  |
| Social |  | C1Px |  | λ31 |  | 248.196 |  | 11.778 |  | 21.073 |  | < .001 |  | 225.111 |  | 271.280 |  |
|  |  | C1Py |  | λ32 |  | 241.364 |  | 11.301 |  | 21.358 |  | < .001 |  | 219.214 |  | 263.514 |  |
|  |  | C1Pz |  | λ33 |  | 242.619 |  | 10.868 |  | 22.324 |  | < .001 |  | 221.318 |  | 263.920 |  |
|  |  | C2Px |  | λ34 |  | 238.773 |  | 11.075 |  | 21.560 |  | < .001 |  | 217.067 |  | 260.480 |  |
|  |  | C2Py |  | λ35 |  | 225.501 |  | 11.009 |  | 20.483 |  | < .001 |  | 203.923 |  | 247.078 |  |
|  |  | C2Pz |  | λ36 |  | 261.093 |  | 11.387 |  | 22.928 |  | < .001 |  | 238.775 |  | 283.412 |  |
|  |  | C3Px |  | λ37 |  | 219.174 |  | 11.910 |  | 18.402 |  | < .001 |  | 195.831 |  | 242.518 |  |
|  |  | C3Py |  | λ38 |  | 242.141 |  | 11.094 |  | 21.827 |  | < .001 |  | 220.398 |  | 263.884 |  |
|  |  | C3Pz |  | λ39 |  | 238.164 |  | 11.284 |  | 21.106 |  | < .001 |  | 216.047 |  | 260.281 |  |
| Spiritual |  | D1Px |  | λ41 |  | 221.876 |  | 10.594 |  | 20.944 |  | < .001 |  | 201.113 |  | 242.639 |  |
|  |  | D1Py |  | λ42 |  | 252.870 |  | 11.313 |  | 22.352 |  | < .001 |  | 230.697 |  | 275.043 |  |
|  |  | D1Pz |  | λ43 |  | 198.454 |  | 10.794 |  | 18.386 |  | < .001 |  | 177.299 |  | 219.609 |  |
|  |  | D2Px |  | λ44 |  | 212.599 |  | 10.650 |  | 19.963 |  | < .001 |  | 191.726 |  | 233.472 |  |
|  |  | D2Py |  | λ45 |  | 224.462 |  | 10.261 |  | 21.875 |  | < .001 |  | 204.351 |  | 244.573 |  |
|  |  | D2Pz |  | λ46 |  | 228.910 |  | 10.519 |  | 21.762 |  | < .001 |  | 208.293 |  | 249.527 |  |
|  |  | D3Px |  | λ47 |  | 239.389 |  | 11.085 |  | 21.596 |  | < .001 |  | 217.664 |  | 261.115 |  |
|  |  | D3Py |  | λ48 |  | 222.625 |  | 11.218 |  | 19.844 |  | < .001 |  | 200.637 |  | 244.613 |  |
|  |  | D3Pz |  | λ49 |  | 234.613 |  | 11.513 |  | 20.378 |  | < .001 |  | 212.048 |  | 257.178 |  |
|  | | | | | | | | | | | | | | | | | |

| **Factor variances** | | | | | | | | | | | | | |
| --- | --- | --- | --- | --- | --- | --- | --- | --- | --- | --- | --- | --- | --- |
|  | | | | | | | | | | **95% Confidence Interval** | | | |
| **Factor** | | **Estimate** | | **Std. Error** | | **z-value** | | **p** | | **Lower** | | **Upper** | |
| Self |  | 1.000 |  | 0.000 |  |  |  |  |  | 1.000 |  | 1.000 |  |
| Material |  | 1.000 |  | 0.000 |  |  |  |  |  | 1.000 |  | 1.000 |  |
| Social |  | 1.000 |  | 0.000 |  |  |  |  |  | 1.000 |  | 1.000 |  |
| Spiritual |  | 1.000 |  | 0.000 |  |  |  |  |  | 1.000 |  | 1.000 |  |
|  | | | | | | | | | | | | | |

| **Factor Covariances** | | | | | | | | | | | | | | | | | |
| --- | --- | --- | --- | --- | --- | --- | --- | --- | --- | --- | --- | --- | --- | --- | --- | --- | --- |
|  | | | | | | | | | | | | | | **95% Confidence Interval** | | | |
|  | |  | |  | | **Estimate** | | **Std. Error** | | **z-value** | | **p** | | **Lower** | | **Upper** | |
| Self |  | ↔ |  | Material |  | 0.989 |  | 0.011 |  | 93.302 |  | < .001 |  | 0.968 |  | 1.009 |  |
| Self |  | ↔ |  | Social |  | 0.921 |  | 0.013 |  | 70.811 |  | < .001 |  | 0.896 |  | 0.947 |  |
| Self |  | ↔ |  | Spiritual |  | 0.967 |  | 0.011 |  | 86.143 |  | < .001 |  | 0.945 |  | 0.989 |  |
| Material |  | ↔ |  | Social |  | 0.951 |  | 0.011 |  | 88.843 |  | < .001 |  | 0.930 |  | 0.972 |  |
| Material |  | ↔ |  | Spiritual |  | 0.966 |  | 0.010 |  | 93.521 |  | < .001 |  | 0.945 |  | 0.986 |  |
| Social |  | ↔ |  | Spiritual |  | 0.925 |  | 0.012 |  | 79.085 |  | < .001 |  | 0.902 |  | 0.948 |  |
|  | | | | | | | | | | | | | | | | | |

| **Residual variances** | | | | | | | | | | | | | |
| --- | --- | --- | --- | --- | --- | --- | --- | --- | --- | --- | --- | --- | --- |
|  | | | | | | | | | | **95% Confidence Interval** | | | |
| **Indicator** | | **Estimate** | | **Std. Error** | | **z-value** | | **p** | | **Lower** | | **Upper** | |
| A1Px |  | 107640.977 |  | 4899.006 |  | 21.972 |  | < .001 |  | 98039.101 |  | 117242.852 |  |
| A1Py |  | 81122.081 |  | 3782.679 |  | 21.446 |  | < .001 |  | 73708.166 |  | 88535.996 |  |
| A1Pz |  | 101506.386 |  | 4600.992 |  | 22.062 |  | < .001 |  | 92488.608 |  | 110524.164 |  |
| A2Px |  | 79417.145 |  | 3695.915 |  | 21.488 |  | < .001 |  | 72173.284 |  | 86661.006 |  |
| A2Py |  | 69639.215 |  | 3248.611 |  | 21.437 |  | < .001 |  | 63272.056 |  | 76006.375 |  |
| A2Pz |  | 93083.661 |  | 4283.898 |  | 21.729 |  | < .001 |  | 84687.376 |  | 101479.946 |  |
| A3Px |  | 114096.536 |  | 5169.905 |  | 22.069 |  | < .001 |  | 103963.708 |  | 124229.364 |  |
| A3Py |  | 119057.807 |  | 5383.012 |  | 22.117 |  | < .001 |  | 108507.297 |  | 129608.318 |  |
| A3Pz |  | 122056.622 |  | 5503.461 |  | 22.178 |  | < .001 |  | 111270.036 |  | 132843.208 |  |
| B1Px |  | 77809.863 |  | 3588.708 |  | 21.682 |  | < .001 |  | 70776.125 |  | 84843.601 |  |
| B1Py |  | 76716.944 |  | 3538.714 |  | 21.679 |  | < .001 |  | 69781.192 |  | 83652.696 |  |
| B1Pz |  | 89841.137 |  | 4112.682 |  | 21.845 |  | < .001 |  | 81780.428 |  | 97901.845 |  |
| B2Px |  | 87886.655 |  | 4044.548 |  | 21.730 |  | < .001 |  | 79959.487 |  | 95813.823 |  |
| B2Py |  | 83664.262 |  | 3870.391 |  | 21.616 |  | < .001 |  | 76078.435 |  | 91250.089 |  |
| B2Pz |  | 86813.787 |  | 4017.401 |  | 21.609 |  | < .001 |  | 78939.826 |  | 94687.749 |  |
| B3Px |  | 104918.156 |  | 4745.536 |  | 22.109 |  | < .001 |  | 95617.078 |  | 114219.235 |  |
| B3Py |  | 106582.960 |  | 4815.155 |  | 22.135 |  | < .001 |  | 97145.429 |  | 116020.491 |  |
| B3Pz |  | 103041.862 |  | 4673.748 |  | 22.047 |  | < .001 |  | 93881.485 |  | 112202.239 |  |
| C1Px |  | 101370.722 |  | 4746.331 |  | 21.358 |  | < .001 |  | 92068.084 |  | 110673.360 |  |
| C1Py |  | 92589.870 |  | 4345.138 |  | 21.309 |  | < .001 |  | 84073.555 |  | 101106.184 |  |
| C1Pz |  | 83250.152 |  | 3939.678 |  | 21.131 |  | < .001 |  | 75528.526 |  | 90971.778 |  |
| C2Px |  | 88414.008 |  | 4156.112 |  | 21.273 |  | < .001 |  | 80268.178 |  | 96559.838 |  |
| C2Py |  | 89983.730 |  | 4194.233 |  | 21.454 |  | < .001 |  | 81763.184 |  | 98204.277 |  |
| C2Pz |  | 89701.919 |  | 4269.503 |  | 21.010 |  | < .001 |  | 81333.848 |  | 98069.990 |  |
| C3Px |  | 110771.222 |  | 5092.958 |  | 21.750 |  | < .001 |  | 100789.208 |  | 120753.236 |  |
| C3Py |  | 88031.552 |  | 4147.552 |  | 21.225 |  | < .001 |  | 79902.500 |  | 96160.604 |  |
| C3Pz |  | 92964.417 |  | 4353.877 |  | 21.352 |  | < .001 |  | 84430.974 |  | 101497.860 |  |
| D1Px |  | 83361.344 |  | 3867.307 |  | 21.555 |  | < .001 |  | 75781.561 |  | 90941.127 |  |
| D1Py |  | 91312.323 |  | 4280.849 |  | 21.330 |  | < .001 |  | 82922.012 |  | 99702.633 |  |
| D1Pz |  | 92176.426 |  | 4212.181 |  | 21.883 |  | < .001 |  | 83920.703 |  | 100432.148 |  |
| D2Px |  | 86437.933 |  | 3984.740 |  | 21.692 |  | < .001 |  | 78627.987 |  | 94247.879 |  |
| D2Py |  | 76186.657 |  | 3558.338 |  | 21.411 |  | < .001 |  | 69212.442 |  | 83160.873 |  |
| D2Pz |  | 80332.737 |  | 3748.737 |  | 21.429 |  | < .001 |  | 72985.347 |  | 87680.127 |  |
| D3Px |  | 89629.110 |  | 4177.396 |  | 21.456 |  | < .001 |  | 81441.563 |  | 97816.656 |  |
| D3Py |  | 96202.854 |  | 4431.726 |  | 21.708 |  | < .001 |  | 87516.831 |  | 104888.878 |  |
| D3Pz |  | 99953.363 |  | 4619.730 |  | 21.636 |  | < .001 |  | 90898.859 |  | 109007.867 |  |
|  | | | | | | | | | | | | | |

**Table 9.1.2 CFA Self domain promotion motivation (“A positive”)**

**Model fit**

| **Chi-square test** | | | | | | | |
| --- | --- | --- | --- | --- | --- | --- | --- |
| **Model** | | **Χ²** | | **df** | | **p** | |
| Baseline model |  | 500.099 |  | 36 |  |  |  |
| Factor model |  | 66.375 |  | 24 |  | < .001 |  |
|  | | | | | | | |

**Additional fit measures**

| **Fit indices** | | | |
| --- | --- | --- | --- |
| **Index** | | **Value** | |
| Comparative Fit Index (CFI) |  | 0.909 |  |
| Tucker-Lewis Index (TLI) |  | 0.863 |  |
| Bentler-Bonett Non-normed Fit Index (NNFI) |  | 0.863 |  |
| Bentler-Bonett Normed Fit Index (NFI) |  | 0.867 |  |
| Parsimony Normed Fit Index (PNFI) |  | 0.578 |  |
| Bollen's Relative Fit Index (RFI) |  | 0.801 |  |
| Bollen's Incremental Fit Index (IFI) |  | 0.911 |  |
| Relative Noncentrality Index (RNI) |  | 0.909 |  |
|  | | | |

| **Information criteria** | | | |
| --- | --- | --- | --- |
|  | | **Value** | |
| Log-likelihood |  | -70190.448 |  |
| Number of free parameters |  | 21.000 |  |
| Akaike (AIC) |  | 140422.895 |  |
| Bayesian (BIC) |  | 140526.599 |  |
| Sample-size adjusted Bayesian (SSABIC) |  | 140459.901 |  |
|  | | | |

| **Other fit measures** | | | |
| --- | --- | --- | --- |
| **Metric** | | **Value** | |
| Root mean square error of approximation (RMSEA) |  | 0.041 |  |
| RMSEA 90% CI lower bound |  | 0.030 |  |
| RMSEA 90% CI upper bound |  | 0.053 |  |
| RMSEA p-value |  | 0.876 |  |
| Standardized root mean square residual (SRMR) |  | 0.036 |  |
| Hoelter's critical N (α = .05) |  | 566.633 |  |
| Hoelter's critical N (α = .01) |  | 668.604 |  |
| Goodness of fit index (GFI) |  | 0.986 |  |
| McDonald fit index (MFI) |  | 0.980 |  |
| Expected cross validation index (ECVI) |  | 0.105 |  |
|  | | | |

| **R-Squared** | | | |
| --- | --- | --- | --- |
|  | | **R²** | |
| A1Px |  | 0.131 |  |
| A1Py |  | 0.204 |  |
| A1Pz |  | 0.102 |  |
| A2Py |  | 0.134 |  |
| A2Pz |  | 0.162 |  |
| A2Px |  | 0.143 |  |
| A3Py |  | 0.244 |  |
| A3Px |  | 0.310 |  |
| A3Pz |  | 0.306 |  |
| Factor 1 |  | 1.000 |  |
| Factor 2 |  | 0.363 |  |
| Factor 3 |  | 0.177 |  |
|  | | | |

**Parameter estimates**

| **Factor loadings** | | | | | | | | | | | | | | | | | |
| --- | --- | --- | --- | --- | --- | --- | --- | --- | --- | --- | --- | --- | --- | --- | --- | --- | --- |
|  | | | | | | | | | | | | | | **95% Confidence Interval** | | | |
| **Factor** | | **Indicator** | | **Symbol** | | **Estimate** | | **Std. Error** | | **z-value** | | **p** | | **Lower** | | **Upper** | |
| Factor 1 |  | A1Px |  | λ11 |  | 0.127 |  |  |  |  |  |  |  |  |  |  |  |
|  |  | A1Py |  | λ12 |  | 0.148 |  |  |  |  |  |  |  |  |  |  |  |
|  |  | A1Pz |  | λ13 |  | 0.117 |  |  |  |  |  |  |  |  |  |  |  |
| Factor 2 |  | A2Py |  | λ21 |  | 127.929 |  |  |  |  |  |  |  |  |  |  |  |
|  |  | A2Pz |  | λ22 |  | 165.817 |  |  |  |  |  |  |  |  |  |  |  |
|  |  | A2Px |  | λ23 |  | 127.950 |  |  |  |  |  |  |  |  |  |  |  |
| Factor 3 |  | A3Py |  | λ31 |  | 214.688 |  |  |  |  |  |  |  |  |  |  |  |
|  |  | A3Px |  | λ32 |  | 233.447 |  |  |  |  |  |  |  |  |  |  |  |
|  |  | A3Pz |  | λ33 |  | 235.635 |  |  |  |  |  |  |  |  |  |  |  |
|  | | | | | | | | | | | | | | | | | |

| **Second-order factor loadings** | | | | | | | | | | | | | | | | | |
| --- | --- | --- | --- | --- | --- | --- | --- | --- | --- | --- | --- | --- | --- | --- | --- | --- | --- |
|  | | | | | | | | | | | | | | **95% Confidence Interval** | | | |
| **Factor** | | **Indicator** | | **Symbol** | | **Estimate** | | **Std. Error** | | **z-value** | | **p** | | **Lower** | | **Upper** | |
| SecondOrder |  | Factor 1 |  | γ11 |  | 1459.173 |  |  |  |  |  |  |  |  |  |  |  |
|  |  | Factor 2 |  | γ12 |  | 0.755 |  |  |  |  |  |  |  |  |  |  |  |
|  |  | Factor 3 |  | γ13 |  | 0.464 |  |  |  |  |  |  |  |  |  |  |  |
|  | | | | | | | | | | | | | | | | | |

| **Factor variances** | | | | | | | | | | | | | |
| --- | --- | --- | --- | --- | --- | --- | --- | --- | --- | --- | --- | --- | --- |
|  | | | | | | | | | | **95% Confidence Interval** | | | |
| **Factor** | | **Estimate** | | **Std. Error** | | **z-value** | | **p** | | **Lower** | | **Upper** | |
| Factor 1 |  | 1.000 |  | 0.000 |  |  |  |  |  | 1.000 |  | 1.000 |  |
| Factor 2 |  | 1.000 |  | 0.000 |  |  |  |  |  | 1.000 |  | 1.000 |  |
| Factor 3 |  | 1.000 |  | 0.000 |  |  |  |  |  | 1.000 |  | 1.000 |  |
| Second-Order |  | 1.000 |  | 0.000 |  |  |  |  |  | 1.000 |  | 1.000 |  |
|  | | | | | | | | | | | | | |

| **Residual variances** | | | | | | | | | | | | | |
| --- | --- | --- | --- | --- | --- | --- | --- | --- | --- | --- | --- | --- | --- |
|  | | | | | | | | | | **95% Confidence Interval** | | | |
| **Indicator** | | **Estimate** | | **Std. Error** | | **z-value** | | **p** | | **Lower** | | **Upper** | |
| A1Px |  | 228090.735 |  |  |  |  |  |  |  |  |  |  |  |
| A1Py |  | 181299.930 |  |  |  |  |  |  |  |  |  |  |  |
| A1Pz |  | 255407.247 |  |  |  |  |  |  |  |  |  |  |  |
| A2Py |  | 165379.998 |  |  |  |  |  |  |  |  |  |  |  |
| A2Pz |  | 222833.853 |  |  |  |  |  |  |  |  |  |  |  |
| A2Px |  | 153698.472 |  |  |  |  |  |  |  |  |  |  |  |
| A3Py |  | 173128.279 |  |  |  |  |  |  |  |  |  |  |  |
| A3Px |  | 147673.463 |  |  |  |  |  |  |  |  |  |  |  |
| A3Pz |  | 153202.539 |  |  |  |  |  |  |  |  |  |  |  |
|  | | | | | | | | | | | | | |

## Table 9.1.3 CFA Material domain promotion motivation (“B positive”)

**Model fit**

| **Chi-square test** | | | | | | | |
| --- | --- | --- | --- | --- | --- | --- | --- |
| **Model** | | **Χ²** | | **df** | | **p** | |
| Baseline model |  | 432.602 |  | 36 |  |  |  |
| Factor model |  | 41.291 |  | 24 |  | 0.015 |  |
|  | | | | | | | |

**Additional fit measures**

| **Fit indices** | | | |
| --- | --- | --- | --- |
| **Index** | | **Value** | |
| Comparative Fit Index (CFI) |  | 0.956 |  |
| Tucker-Lewis Index (TLI) |  | 0.935 |  |
| Bentler-Bonett Non-normed Fit Index (NNFI) |  | 0.935 |  |
| Bentler-Bonett Normed Fit Index (NFI) |  | 0.905 |  |
| Parsimony Normed Fit Index (PNFI) |  | 0.603 |  |
| Bollen's Relative Fit Index (RFI) |  | 0.857 |  |
| Bollen's Incremental Fit Index (IFI) |  | 0.958 |  |
| Relative Noncentrality Index (RNI) |  | 0.956 |  |
|  | | | |

| **Information criteria** | | | |
| --- | --- | --- | --- |
|  | | **Value** | |
| Log-likelihood |  | -70646.204 |  |
| Number of free parameters |  | 21.000 |  |
| Akaike (AIC) |  | 141334.408 |  |
| Bayesian (BIC) |  | 141438.112 |  |
| Sample-size adjusted Bayesian (SSABIC) |  | 141371.413 |  |
|  | | | |

| **Other fit measures** | | | |
| --- | --- | --- | --- |
| **Metric** | | **Value** | |
| Root mean square error of approximation (RMSEA) |  | 0.026 |  |
| RMSEA 90% CI lower bound |  | 0.012 |  |
| RMSEA 90% CI upper bound |  | 0.040 |  |
| RMSEA p-value |  | 0.999 |  |
| Standardized root mean square residual (SRMR) |  | 0.027 |  |
| Hoelter's critical N (α = .05) |  | 910.244 |  |
| Hoelter's critical N (α = .01) |  | 1074.159 |  |
| Goodness of fit index (GFI) |  | 0.991 |  |
| McDonald fit index (MFI) |  | 0.992 |  |
| Expected cross validation index (ECVI) |  | 0.081 |  |
|  | | | |

| **R-Squared** | | | |
| --- | --- | --- | --- |
|  | | **R²** | |
| B1Px |  | 0.094 |  |
| B1Py |  | 0.140 |  |
| B1Pz |  | 0.169 |  |
| B2Px |  | 0.221 |  |
| B2Py |  | 0.117 |  |
| B2Pz |  | 0.266 |  |
| B3Px |  | 0.186 |  |
| B3Py |  | 0.217 |  |
| B3Pz |  | 0.188 |  |
| Factor 1 |  | 0.851 |  |
| Factor 2 |  | 0.389 |  |
| Factor 3 |  | 0.428 |  |
|  | | | |

**Parameter estimates**

| **Factor loadings** | | | | | | | | | | | | | | | | | |
| --- | --- | --- | --- | --- | --- | --- | --- | --- | --- | --- | --- | --- | --- | --- | --- | --- | --- |
|  | | | | | | | | | | | | | | **95% Confidence Interval** | | | |
| **Factor** | | **Indicator** | | **Symbol** | | **Estimate** | | **Std. Error** | | **z-value** | | **p** | | **Lower** | | **Upper** | |
| Factor 1 |  | B1Px |  | λ11 |  | 64.014 |  | 47.583 |  | 1.345 |  | 0.179 |  | -29.248 |  | 157.276 |  |
|  |  | B1Py |  | λ12 |  | 73.411 |  | 54.592 |  | 1.345 |  | 0.179 |  | -33.588 |  | 180.409 |  |
|  |  | B1Pz |  | λ13 |  | 77.940 |  | 58.255 |  | 1.338 |  | 0.181 |  | -36.237 |  | 192.118 |  |
| Factor 2 |  | B2Px |  | λ21 |  | 183.285 |  | 24.906 |  | 7.359 |  | < .001 |  | 134.469 |  | 232.100 |  |
|  |  | B2Py |  | λ22 |  | 117.989 |  | 18.523 |  | 6.370 |  | < .001 |  | 81.686 |  | 154.293 |  |
|  |  | B2Pz |  | λ23 |  | 198.296 |  | 26.884 |  | 7.376 |  | < .001 |  | 145.605 |  | 250.987 |  |
| Factor 3 |  | B3Px |  | λ31 |  | 167.328 |  | 25.020 |  | 6.688 |  | < .001 |  | 118.290 |  | 216.366 |  |
|  |  | B3Py |  | λ32 |  | 178.299 |  | 26.213 |  | 6.802 |  | < .001 |  | 126.922 |  | 229.675 |  |
|  |  | B3Pz |  | λ33 |  | 170.905 |  | 25.521 |  | 6.697 |  | < .001 |  | 120.885 |  | 220.925 |  |
|  | | | | | | | | | | | | | | | | | |

| **Second-order factor loadings** | | | | | | | | | | | | | | | | | |
| --- | --- | --- | --- | --- | --- | --- | --- | --- | --- | --- | --- | --- | --- | --- | --- | --- | --- |
|  | | | | | | | | | | | | | | **95% Confidence Interval** | | | |
| **Factor** | | **Indicator** | | **Symbol** | | **Estimate** | | **Std. Error** | | **z-value** | | **p** | | **Lower** | | **Upper** | |
| SecondOrder |  | Factor 1 |  | γ11 |  | 2.388 |  | 1.981 |  | 1.206 |  | 0.228 |  | -1.494 |  | 6.270 |  |
|  |  | Factor 2 |  | γ12 |  | 0.797 |  | 0.163 |  | 4.881 |  | < .001 |  | 0.477 |  | 1.117 |  |
|  |  | Factor 3 |  | γ13 |  | 0.864 |  | 0.187 |  | 4.628 |  | < .001 |  | 0.498 |  | 1.230 |  |
|  | | | | | | | | | | | | | | | | | |

| **Factor variances** | | | | | | | | | | | | | |
| --- | --- | --- | --- | --- | --- | --- | --- | --- | --- | --- | --- | --- | --- |
|  | | | | | | | | | | **95% Confidence Interval** | | | |
| **Factor** | | **Estimate** | | **Std. Error** | | **z-value** | | **p** | | **Lower** | | **Upper** | |
| Factor 1 |  | 1.000 |  | 0.000 |  |  |  |  |  | 1.000 |  | 1.000 |  |
| Factor 2 |  | 1.000 |  | 0.000 |  |  |  |  |  | 1.000 |  | 1.000 |  |
| Factor 3 |  | 1.000 |  | 0.000 |  |  |  |  |  | 1.000 |  | 1.000 |  |
| Second-Order |  | 1.000 |  | 0.000 |  |  |  |  |  | 1.000 |  | 1.000 |  |
|  | | | | | | | | | | | | | |

| **Residual variances** | | | | | | | | | | | | | |
| --- | --- | --- | --- | --- | --- | --- | --- | --- | --- | --- | --- | --- | --- |
|  | | | | | | | | | | **95% Confidence Interval** | | | |
| **Indicator** | | **Estimate** | | **Std. Error** | | **z-value** | | **p** | | **Lower** | | **Upper** | |
| B1Px |  | 265602.259 |  | 13491.029 |  | 19.687 |  | < .001 |  | 239160.328 |  | 292044.190 |  |
| B1Py |  | 222309.280 |  | 12467.410 |  | 17.831 |  | < .001 |  | 197873.605 |  | 246744.955 |  |
| B1Pz |  | 200184.947 |  | 12149.765 |  | 16.476 |  | < .001 |  | 176371.846 |  | 223998.049 |  |
| B2Px |  | 193142.040 |  | 12631.345 |  | 15.291 |  | < .001 |  | 168385.060 |  | 217899.021 |  |
| B2Py |  | 171149.733 |  | 8822.776 |  | 19.399 |  | < .001 |  | 153857.411 |  | 188442.056 |  |
| B2Pz |  | 177748.842 |  | 13250.969 |  | 13.414 |  | < .001 |  | 151777.419 |  | 203720.264 |  |
| B3Px |  | 214115.994 |  | 12741.506 |  | 16.805 |  | < .001 |  | 189143.101 |  | 239088.887 |  |
| B3Py |  | 200447.187 |  | 12908.322 |  | 15.529 |  | < .001 |  | 175147.341 |  | 225747.032 |  |
| B3Pz |  | 220934.176 |  | 13200.040 |  | 16.737 |  | < .001 |  | 195062.573 |  | 246805.779 |  |
|  | | | | | | | | | | | | | |

**Table 9.1.4 CFA Social domain promotion motivation (“C positive”)**

**Model fit**

| **Chi-square test** | | | | | | | |
| --- | --- | --- | --- | --- | --- | --- | --- |
| **Model** | | **Χ²** | | **df** | | **p** | |
| Baseline model |  | 594.901 |  | 36 |  |  |  |
| Factor model |  | 40.063 |  | 24 |  | 0.026 |  |
|  | | | | | | | |

**Additional fit measures**

| **Fit indices** | | | |
| --- | --- | --- | --- |
| **Index** | | **Value** | |
| Comparative Fit Index (CFI) |  | 0.977 |  |
| Tucker-Lewis Index (TLI) |  | 0.962 |  |
| Bentler-Bonett Non-normed Fit Index (NNFI) |  | 0.962 |  |
| Bentler-Bonett Normed Fit Index (NFI) |  | 0.949 |  |
| Parsimony Normed Fit Index (PNFI) |  | 0.576 |  |
| Bollen's Relative Fit Index (RFI) |  | 0.917 |  |
| Bollen's Incremental Fit Index (IFI) |  | 0.977 |  |
| Relative Noncentrality Index (RNI) |  | 0.977 |  |
|  | | | |

| **Information criteria** | | | |
| --- | --- | --- | --- |
|  | | **Value** | |
| Log-likelihood |  | -62711.006 |  |
| Number of free parameters |  | 19.000 |  |
| Akaike (AIC) |  | 125460.013 |  |
| Bayesian (BIC) |  | 125553.840 |  |
| Sample-size adjusted Bayesian (SSABIC) |  | 125493.494 |  |
|  | | | |

| **Other fit measures** | | | |
| --- | --- | --- | --- |
| **Metric** | | **Value** | |
| Root mean square error of approximation (RMSEA) |  | 0.027 |  |
| RMSEA 90% CI lower bound |  | 0.009 |  |
| RMSEA 90% CI upper bound |  | 0.043 |  |
| RMSEA p-value |  | 0.993 |  |
| Standardized root mean square residual (SRMR) |  | 0.024 |  |
| Hoelter's critical N (α = .05) |  | 947.101 |  |
| Hoelter's critical N (α = .01) |  | 1146.751 |  |
| Goodness of fit index (GFI) |  | 0.993 |  |
| McDonald fit index (MFI) |  | 0.994 |  |
| Expected cross validation index (ECVI) |  | 0.066 |  |
|  | | | |

| **R-Squared** | | | |
| --- | --- | --- | --- |
|  | | **R²** | |
| C1Px |  | 0.240 |  |
| C1Py |  | 0.256 |  |
| C1Pz |  | 0.143 |  |
| C2Px |  | 0.217 |  |
| C2Py |  | 0.032 |  |
| C2Pz |  | 0.314 |  |
| C3Px |  | 0.316 |  |
| C3Py |  | 0.254 |  |
| C3Pz |  | 0.215 |  |
| Factor 1 |  | 1.000 |  |
| Factor 2 |  | 0.714 |  |
| Factor 3 |  | 0.588 |  |
|  | | | |

**Parameter estimates**

| **Factor loadings** | | | | | | | | | | | | | | | | | |
| --- | --- | --- | --- | --- | --- | --- | --- | --- | --- | --- | --- | --- | --- | --- | --- | --- | --- |
|  | | | | | | | | | | | | | | **95% Confidence Interval** | | | |
| **Factor** | | **Indicator** | | **Symbol** | | **Estimate** | | **Std. Error** | | **z-value** | | **p** | | **Lower** | | **Upper** | |
| Factor 1 |  | C1Px |  | λ11 |  | 0.166 |  |  |  |  |  |  |  |  |  |  |  |
|  |  | C1Py |  | λ12 |  | 0.173 |  |  |  |  |  |  |  |  |  |  |  |
|  |  | C1Pz |  | λ13 |  | 0.128 |  |  |  |  |  |  |  |  |  |  |  |
| Factor 2 |  | C2Px |  | λ21 |  | 125.618 |  |  |  |  |  |  |  |  |  |  |  |
|  |  | C2Py |  | λ22 |  | 49.560 |  |  |  |  |  |  |  |  |  |  |  |
|  |  | C2Pz |  | λ23 |  | 145.318 |  |  |  |  |  |  |  |  |  |  |  |
| Factor 3 |  | C3Px |  | λ31 |  | 150.312 |  |  |  |  |  |  |  |  |  |  |  |
|  |  | C3Py |  | λ32 |  | 163.011 |  |  |  |  |  |  |  |  |  |  |  |
|  |  | C3Pz |  | λ33 |  | 147.199 |  |  |  |  |  |  |  |  |  |  |  |
|  | | | | | | | | | | | | | | | | | |

| **Second-order factor loadings** | | | | | | | | | | | | | | | | | |
| --- | --- | --- | --- | --- | --- | --- | --- | --- | --- | --- | --- | --- | --- | --- | --- | --- | --- |
|  | | | | | | | | | | | | | | **95% Confidence Interval** | | | |
| **Factor** | | **Indicator** | | **Symbol** | | **Estimate** | | **Std. Error** | | **z-value** | | **p** | | **Lower** | | **Upper** | |
| SecondOrder |  | Factor 1 |  | γ11 |  | 1483.038 |  |  |  |  |  |  |  |  |  |  |  |
|  |  | Factor 2 |  | γ12 |  | 1.581 |  |  |  |  |  |  |  |  |  |  |  |
|  |  | Factor 3 |  | γ13 |  | 1.194 |  |  |  |  |  |  |  |  |  |  |  |
|  | | | | | | | | | | | | | | | | | |

| **Factor variances** | | | | | | | | | | | | | |
| --- | --- | --- | --- | --- | --- | --- | --- | --- | --- | --- | --- | --- | --- |
|  | | | | | | | | | | **95% Confidence Interval** | | | |
| **Factor** | | **Estimate** | | **Std. Error** | | **z-value** | | **p** | | **Lower** | | **Upper** | |
| Factor 1 |  | 1.000 |  | 0.000 |  |  |  |  |  | 1.000 |  | 1.000 |  |
| Factor 2 |  | 1.000 |  | 0.000 |  |  |  |  |  | 1.000 |  | 1.000 |  |
| Factor 3 |  | 1.000 |  | 0.000 |  |  |  |  |  | 1.000 |  | 1.000 |  |
| Second-Order |  | 1.000 |  | 0.000 |  |  |  |  |  | 1.000 |  | 1.000 |  |
|  | | | | | | | | | | | | | |

| **Residual variances** | | | | | | | | | | | | | |
| --- | --- | --- | --- | --- | --- | --- | --- | --- | --- | --- | --- | --- | --- |
|  | | | | | | | | | | **95% Confidence Interval** | | | |
| **Indicator** | | **Estimate** | | **Std. Error** | | **z-value** | | **p** | | **Lower** | | **Upper** | |
| C1Px |  | 191295.891 |  |  |  |  |  |  |  |  |  |  |  |
| C1Py |  | 192540.199 |  |  |  |  |  |  |  |  |  |  |  |
| C1Pz |  | 214654.234 |  |  |  |  |  |  |  |  |  |  |  |
| C2Px |  | 199120.305 |  |  |  |  |  |  |  |  |  |  |  |
| C2Py |  | 259572.774 |  |  |  |  |  |  |  |  |  |  |  |
| C2Pz |  | 161466.390 |  |  |  |  |  |  |  |  |  |  |  |
| C3Px |  | 108699.401 |  |  |  |  |  |  |  |  |  |  |  |
| C3Py |  | 189402.048 |  |  |  |  |  |  |  |  |  |  |  |
| C3Pz |  | 191582.173 |  |  |  |  |  |  |  |  |  |  |  |
|  | | | | | | | | | | | | | |

**Table 9.1.5 CFA Spiritual domain promotion motivation (“D positive”)**

**Model fit**

| **Chi-square test** | | | | | | | |
| --- | --- | --- | --- | --- | --- | --- | --- |
| **Model** | | **Χ²** | | **df** | | **p** | |
| Baseline model |  | 667.588 |  | 36 |  |  |  |
| Factor model |  | 50.010 |  | 24 |  | 0.001 |  |
|  | | | | | | | |

**Additional fit measures**

| **Fit indices** | | | |
| --- | --- | --- | --- |
| **Index** | | **Value** | |
| Comparative Fit Index (CFI) |  | 0.959 |  |
| Tucker-Lewis Index (TLI) |  | 0.938 |  |
| Bentler-Bonett Non-normed Fit Index (NNFI) |  | 0.938 |  |
| Bentler-Bonett Normed Fit Index (NFI) |  | 0.925 |  |
| Parsimony Normed Fit Index (PNFI) |  | 0.617 |  |
| Bollen's Relative Fit Index (RFI) |  | 0.888 |  |
| Bollen's Incremental Fit Index (IFI) |  | 0.960 |  |
| Relative Noncentrality Index (RNI) |  | 0.959 |  |
|  | | | |

| **Information criteria** | | | |
| --- | --- | --- | --- |
|  | | **Value** | |
| Log-likelihood |  | -70626.967 |  |
| Number of free parameters |  | 21.000 |  |
| Akaike (AIC) |  | 141295.934 |  |
| Bayesian (BIC) |  | 141399.638 |  |
| Sample-size adjusted Bayesian (SSABIC) |  | 141332.939 |  |
|  | | | |

| **Other fit measures** | | | |
| --- | --- | --- | --- |
| **Metric** | | **Value** | |
| Root mean square error of approximation (RMSEA) |  | 0.032 |  |
| RMSEA 90% CI lower bound |  | 0.020 |  |
| RMSEA 90% CI upper bound |  | 0.045 |  |
| RMSEA p-value |  | 0.990 |  |
| Standardized root mean square residual (SRMR) |  | 0.031 |  |
| Hoelter's critical N (α = .05) |  | 751.721 |  |
| Hoelter's critical N (α = .01) |  | 887.058 |  |
| Goodness of fit index (GFI) |  | 0.989 |  |
| McDonald fit index (MFI) |  | 0.987 |  |
| Expected cross validation index (ECVI) |  | 0.089 |  |
|  | | | |

| **R-Squared** | | | |
| --- | --- | --- | --- |
|  | | **R²** | |
| D1Px |  | 0.376 |  |
| D1Py |  | 0.116 |  |
| D1Pz |  | 0.184 |  |
| D2Px |  | 0.310 |  |
| D2Py |  | 0.237 |  |
| D2Pz |  | 0.312 |  |
| D3Px |  | 0.200 |  |
| D3Py |  | 0.205 |  |
| D3Pz |  | 0.271 |  |
| Factor 1 |  | 0.449 |  |
| Factor 2 |  | 0.425 |  |
| Factor 3 |  | 0.351 |  |
|  | | | |

**Parameter estimates**

| **Factor loadings** | | | | | | | | | | | | | | | | | |
| --- | --- | --- | --- | --- | --- | --- | --- | --- | --- | --- | --- | --- | --- | --- | --- | --- | --- |
|  | | | | | | | | | | | | | | **95% Confidence Interval** | | | |
| **Factor** | | **Indicator** | | **Symbol** | | **Estimate** | | **Std. Error** | | **z-value** | | **p** | | **Lower** | | **Upper** | |
| Factor 1 |  | D1Px |  | λ11 |  | 244.328 |  | 35.676 |  | 6.848 |  | < .001 |  | 174.404 |  | 314.252 |  |
|  |  | D1Py |  | λ12 |  | 125.613 |  | 20.070 |  | 6.259 |  | < .001 |  | 86.277 |  | 164.950 |  |
|  |  | D1Pz |  | λ13 |  | 162.884 |  | 23.221 |  | 7.015 |  | < .001 |  | 117.373 |  | 208.396 |  |
| Factor 2 |  | D2Px |  | λ21 |  | 211.223 |  | 24.803 |  | 8.516 |  | < .001 |  | 162.609 |  | 259.837 |  |
|  |  | D2Py |  | λ22 |  | 186.956 |  | 22.734 |  | 8.224 |  | < .001 |  | 142.398 |  | 231.514 |  |
|  |  | D2Pz |  | λ23 |  | 220.808 |  | 25.918 |  | 8.519 |  | < .001 |  | 170.009 |  | 271.607 |  |
| Factor 3 |  | D3Px |  | λ31 |  | 179.129 |  | 22.440 |  | 7.982 |  | < .001 |  | 135.147 |  | 223.112 |  |
|  |  | D3Py |  | λ32 |  | 181.040 |  | 22.569 |  | 8.022 |  | < .001 |  | 136.806 |  | 225.274 |  |
|  |  | D3Pz |  | λ33 |  | 203.553 |  | 24.490 |  | 8.312 |  | < .001 |  | 155.553 |  | 251.552 |  |
|  | | | | | | | | | | | | | | | | | |

| **Second-order factor loadings** | | | | | | | | | | | | | | | | | |
| --- | --- | --- | --- | --- | --- | --- | --- | --- | --- | --- | --- | --- | --- | --- | --- | --- | --- |
|  | | | | | | | | | | | | | | **95% Confidence Interval** | | | |
| **Factor** | | **Indicator** | | **Symbol** | | **Estimate** | | **Std. Error** | | **z-value** | | **p** | | **Lower** | | **Upper** | |
| SecondOrder |  | Factor 1 |  | γ11 |  | 0.904 |  | 0.199 |  | 4.540 |  | < .001 |  | 0.513 |  | 1.294 |  |
|  |  | Factor 2 |  | γ12 |  | 0.859 |  | 0.174 |  | 4.928 |  | < .001 |  | 0.517 |  | 1.200 |  |
|  |  | Factor 3 |  | γ13 |  | 0.735 |  | 0.141 |  | 5.216 |  | < .001 |  | 0.459 |  | 1.011 |  |
|  | | | | | | | | | | | | | | | | | |

| **Factor variances** | | | | | | | | | | | | | |
| --- | --- | --- | --- | --- | --- | --- | --- | --- | --- | --- | --- | --- | --- |
|  | | | | | | | | | | **95% Confidence Interval** | | | |
| **Factor** | | **Estimate** | | **Std. Error** | | **z-value** | | **p** | | **Lower** | | **Upper** | |
| Factor 1 |  | 1.000 |  | 0.000 |  |  |  |  |  | 1.000 |  | 1.000 |  |
| Factor 2 |  | 1.000 |  | 0.000 |  |  |  |  |  | 1.000 |  | 1.000 |  |
| Factor 3 |  | 1.000 |  | 0.000 |  |  |  |  |  | 1.000 |  | 1.000 |  |
| Second-Order |  | 1.000 |  | 0.000 |  |  |  |  |  | 1.000 |  | 1.000 |  |
|  | | | | | | | | | | | | | |

| **Residual variances** | | | | | | | | | | | | | |
| --- | --- | --- | --- | --- | --- | --- | --- | --- | --- | --- | --- | --- | --- |
|  | | | | | | | | | | **95% Confidence Interval** | | | |
| **Indicator** | | **Estimate** | | **Std. Error** | | **z-value** | | **p** | | **Lower** | | **Upper** | |
| D1Px |  | 179794.998 |  | 18155.150 |  | 9.903 |  | < .001 |  | 144211.557 |  | 215378.439 |  |
| D1Py |  | 217986.749 |  | 10980.484 |  | 19.852 |  | < .001 |  | 196465.397 |  | 239508.101 |  |
| D1Pz |  | 214316.047 |  | 12261.158 |  | 17.479 |  | < .001 |  | 190284.620 |  | 238347.475 |  |
| D2Px |  | 172667.010 |  | 11726.823 |  | 14.724 |  | < .001 |  | 149682.860 |  | 195651.161 |  |
| D2Py |  | 195074.342 |  | 11304.681 |  | 17.256 |  | < .001 |  | 172917.574 |  | 217231.110 |  |
| D2Pz |  | 186904.976 |  | 12757.572 |  | 14.651 |  | < .001 |  | 161900.594 |  | 211909.359 |  |
| D3Px |  | 197823.793 |  | 11750.682 |  | 16.835 |  | < .001 |  | 174792.879 |  | 220854.706 |  |
| D3Py |  | 196238.938 |  | 11785.941 |  | 16.650 |  | < .001 |  | 173138.917 |  | 219338.959 |  |
| D3Pz |  | 172058.334 |  | 12289.640 |  | 14.000 |  | < .001 |  | 147971.082 |  | 196145.585 |  |
|  | | | | | | | | | | | | | |
