# Supplementary material for: The structure of human motivation
Source: BMC Psychol. 2023 Oct 6;11:308. doi: 10.1186/s40359-023-01346-5 (PMC10557177; doi:10.1186/s40359-023-01346-5)
Supplement: Supplementary file 4 — Additional file 4: SM Table 9. Confirmatory factor models for the four life domains: Full output. [file 40359_2023_1346_MOESM4_ESM.zip › Table 9.2 CFA.life.domains.NegativeR5.docx]

## Table 9.2 CFA-Domains-Prevention

**Table 9.2.1 CFA Overall prevention motivation (“negative”)**

### Model fit

| **Chi-square test** | | | | | | | |
| --- | --- | --- | --- | --- | --- | --- | --- |
| **Model** | | **Χ²** | | **df** | | **p** | |
| Baseline model |  | 17392.013 |  | 630 |  |  |  |
| Factor model |  | 1338.450 |  | 588 |  | < .001 |  |
|  | | | | | | | |

#### Additional fit measures

| **Fit indices** | | | |
| --- | --- | --- | --- |
| **Index** | | **Value** | |
| Comparative Fit Index (CFI) |  | 0.955 |  |
| Tucker-Lewis Index (TLI) |  | 0.952 |  |
| Bentler-Bonett Non-normed Fit Index (NNFI) |  | 0.952 |  |
| Bentler-Bonett Normed Fit Index (NFI) |  | 0.923 |  |
| Parsimony Normed Fit Index (PNFI) |  | 0.862 |  |
| Bollen's Relative Fit Index (RFI) |  | 0.918 |  |
| Bollen's Incremental Fit Index (IFI) |  | 0.955 |  |
| Relative Noncentrality Index (RNI) |  | 0.955 |  |
|  | | | |

| **Information criteria** | | | |
| --- | --- | --- | --- |
|  | | **Value** | |
| Log-likelihood |  | -266224.363 |  |
| Number of free parameters |  | 78.000 |  |
| Akaike (AIC) |  | 532604.727 |  |
| Bayesian (BIC) |  | 532989.913 |  |
| Sample-size adjusted Bayesian (SSABIC) |  | 532742.176 |  |
|  | | | |

| **Other fit measures** | | | |
| --- | --- | --- | --- |
| **Metric** | | **Value** | |
| Root mean square error of approximation (RMSEA) |  | 0.035 |  |
| RMSEA 90% CI lower bound |  | 0.033 |  |
| RMSEA 90% CI upper bound |  | 0.038 |  |
| RMSEA p-value |  | 1.000 |  |
| Standardized root mean square residual (SRMR) |  | 0.027 |  |
| Hoelter's critical N (α = .05) |  | 498.241 |  |
| Hoelter's critical N (α = .01) |  | 517.641 |  |
| Goodness of fit index (GFI) |  | 0.930 |  |
| McDonald fit index (MFI) |  | 0.695 |  |
| Expected cross validation index (ECVI) |  | 1.450 |  |
|  | | | |

| **R-Squared** | | | |
| --- | --- | --- | --- |
|  | | **R²** | |
| A1Nx |  | 0.430 |  |
| A1Ny |  | 0.424 |  |
| A1Nz |  | 0.419 |  |
| A2Nx |  | 0.422 |  |
| A2Ny |  | 0.351 |  |
| A2Nz |  | 0.367 |  |
| A3Nx |  | 0.401 |  |
| A3Ny |  | 0.406 |  |
| A3Nz |  | 0.326 |  |
| B1Nx |  | 0.359 |  |
| B1Ny |  | 0.397 |  |
| B1Nz |  | 0.430 |  |
| B2Nx |  | 0.383 |  |
| B2Ny |  | 0.404 |  |
| B2Nz |  | 0.414 |  |
| B3Nx |  | 0.487 |  |
| B3Ny |  | 0.473 |  |
| B3Nz |  | 0.401 |  |
| C1Nx |  | 0.444 |  |
| C1Ny |  | 0.420 |  |
| C1Nz |  | 0.442 |  |
| C2Nx |  | 0.426 |  |
| C2Ny |  | 0.379 |  |
| C2Nz |  | 0.409 |  |
| C3Nx |  | 0.463 |  |
| C3Ny |  | 0.500 |  |
| C3Nz |  | 0.449 |  |
| D1Nx |  | 0.465 |  |
| D1Ny |  | 0.375 |  |
| D1Nz |  | 0.436 |  |
| D2Nx |  | 0.444 |  |
| D2Ny |  | 0.446 |  |
| D2Nz |  | 0.467 |  |
| D3Nx |  | 0.423 |  |
| D3Ny |  | 0.339 |  |
| D3Nz |  | 0.393 |  |
|  | | | |

### Parameter estimates

| **Factor loadings** | | | | | | | | | | | | | | | | | |
| --- | --- | --- | --- | --- | --- | --- | --- | --- | --- | --- | --- | --- | --- | --- | --- | --- | --- |
|  | | | | | | | | | | | | | | **95% Confidence Interval** | | | |
| **Factor** | | **Indicator** | | **Symbol** | | **Estimate** | | **Std. Error** | | **z-value** | | **p** | | **Lower** | | **Upper** | |
| Self |  | A1Nx |  | λ11 |  | 258.439 |  | 11.101 |  | 23.282 |  | < .001 |  | 236.683 |  | 280.196 |  |
|  |  | A1Ny |  | λ12 |  | 248.399 |  | 10.756 |  | 23.095 |  | < .001 |  | 227.319 |  | 269.480 |  |
|  |  | A1Nz |  | λ13 |  | 263.124 |  | 11.474 |  | 22.933 |  | < .001 |  | 240.636 |  | 285.612 |  |
|  |  | A2Nx |  | λ14 |  | 243.268 |  | 10.561 |  | 23.035 |  | < .001 |  | 222.569 |  | 263.967 |  |
|  |  | A2Ny |  | λ15 |  | 236.187 |  | 11.497 |  | 20.544 |  | < .001 |  | 213.654 |  | 258.720 |  |
|  |  | A2Nz |  | λ16 |  | 222.942 |  | 10.567 |  | 21.099 |  | < .001 |  | 202.232 |  | 243.652 |  |
|  |  | A3Nx |  | λ17 |  | 240.952 |  | 10.803 |  | 22.304 |  | < .001 |  | 219.779 |  | 262.126 |  |
|  |  | A3Ny |  | λ18 |  | 248.904 |  | 11.087 |  | 22.449 |  | < .001 |  | 227.173 |  | 270.634 |  |
|  |  | A3Nz |  | λ19 |  | 223.842 |  | 11.399 |  | 19.637 |  | < .001 |  | 201.501 |  | 246.183 |  |
| Material |  | B1Nx |  | λ21 |  | 232.481 |  | 11.231 |  | 20.701 |  | < .001 |  | 210.469 |  | 254.493 |  |
|  |  | B1Ny |  | λ22 |  | 249.583 |  | 11.334 |  | 22.021 |  | < .001 |  | 227.370 |  | 271.797 |  |
|  |  | B1Nz |  | λ23 |  | 252.008 |  | 10.883 |  | 23.156 |  | < .001 |  | 230.678 |  | 273.338 |  |
|  |  | B2Nx |  | λ24 |  | 247.735 |  | 11.502 |  | 21.539 |  | < .001 |  | 225.192 |  | 270.278 |  |
|  |  | B2Ny |  | λ25 |  | 260.337 |  | 11.690 |  | 22.270 |  | < .001 |  | 237.425 |  | 283.250 |  |
|  |  | B2Nz |  | λ26 |  | 252.294 |  | 11.154 |  | 22.619 |  | < .001 |  | 230.433 |  | 274.155 |  |
|  |  | B3Nx |  | λ27 |  | 274.441 |  | 10.921 |  | 25.130 |  | < .001 |  | 253.036 |  | 295.846 |  |
|  |  | B3Ny |  | λ28 |  | 273.842 |  | 11.106 |  | 24.657 |  | < .001 |  | 252.075 |  | 295.609 |  |
|  |  | B3Nz |  | λ29 |  | 237.092 |  | 10.701 |  | 22.156 |  | < .001 |  | 216.118 |  | 258.065 |  |
| Social |  | C1Nx |  | λ31 |  | 250.143 |  | 10.615 |  | 23.564 |  | < .001 |  | 229.337 |  | 270.949 |  |
|  |  | C1Ny |  | λ32 |  | 243.457 |  | 10.706 |  | 22.741 |  | < .001 |  | 222.474 |  | 264.440 |  |
|  |  | C1Nz |  | λ33 |  | 240.215 |  | 10.227 |  | 23.489 |  | < .001 |  | 220.172 |  | 260.259 |  |
|  |  | C2Nx |  | λ34 |  | 270.414 |  | 11.788 |  | 22.940 |  | < .001 |  | 247.310 |  | 293.519 |  |
|  |  | C2Ny |  | λ35 |  | 243.450 |  | 11.430 |  | 21.298 |  | < .001 |  | 221.047 |  | 265.854 |  |
|  |  | C2Nz |  | λ36 |  | 260.165 |  | 11.632 |  | 22.367 |  | < .001 |  | 237.367 |  | 282.963 |  |
|  |  | C3Nx |  | λ37 |  | 282.849 |  | 11.676 |  | 24.224 |  | < .001 |  | 259.964 |  | 305.734 |  |
|  |  | C3Ny |  | λ38 |  | 289.342 |  | 11.339 |  | 25.517 |  | < .001 |  | 267.118 |  | 311.567 |  |
|  |  | C3Nz |  | λ39 |  | 280.569 |  | 11.820 |  | 23.737 |  | < .001 |  | 257.403 |  | 303.736 |  |
| Spiritual |  | D1Nx |  | λ41 |  | 268.496 |  | 11.025 |  | 24.354 |  | < .001 |  | 246.888 |  | 290.103 |  |
|  |  | D1Ny |  | λ42 |  | 232.484 |  | 10.957 |  | 21.218 |  | < .001 |  | 211.008 |  | 253.959 |  |
|  |  | D1Nz |  | λ43 |  | 255.653 |  | 10.946 |  | 23.357 |  | < .001 |  | 234.200 |  | 277.106 |  |
|  |  | D2Nx |  | λ44 |  | 268.863 |  | 11.391 |  | 23.603 |  | < .001 |  | 246.537 |  | 291.189 |  |
|  |  | D2Ny |  | λ45 |  | 253.450 |  | 10.706 |  | 23.674 |  | < .001 |  | 232.467 |  | 274.433 |  |
|  |  | D2Nz |  | λ46 |  | 257.321 |  | 10.539 |  | 24.416 |  | < .001 |  | 236.666 |  | 277.977 |  |
|  |  | D3Nx |  | λ47 |  | 254.383 |  | 11.118 |  | 22.881 |  | < .001 |  | 232.593 |  | 276.173 |  |
|  |  | D3Ny |  | λ48 |  | 228.372 |  | 11.455 |  | 19.937 |  | < .001 |  | 205.921 |  | 250.822 |  |
|  |  | D3Nz |  | λ49 |  | 254.030 |  | 11.629 |  | 21.844 |  | < .001 |  | 231.237 |  | 276.822 |  |
|  | | | | | | | | | | | | | | | | | |

| **Factor variances** | | | | | | | | | | | | | |
| --- | --- | --- | --- | --- | --- | --- | --- | --- | --- | --- | --- | --- | --- |
|  | | | | | | | | | | **95% Confidence Interval** | | | |
| **Factor** | | **Estimate** | | **Std. Error** | | **z-value** | | **p** | | **Lower** | | **Upper** | |
| Self |  | 1.000 |  | 0.000 |  |  |  |  |  | 1.000 |  | 1.000 |  |
| Material |  | 1.000 |  | 0.000 |  |  |  |  |  | 1.000 |  | 1.000 |  |
| Social |  | 1.000 |  | 0.000 |  |  |  |  |  | 1.000 |  | 1.000 |  |
| Spiritual |  | 1.000 |  | 0.000 |  |  |  |  |  | 1.000 |  | 1.000 |  |
|  | | | | | | | | | | | | | |

| **Factor Covariances** | | | | | | | | | | | | | | | | | |
| --- | --- | --- | --- | --- | --- | --- | --- | --- | --- | --- | --- | --- | --- | --- | --- | --- | --- |
|  | | | | | | | | | | | | | | **95% Confidence Interval** | | | |
|  | |  | |  | | **Estimate** | | **Std. Error** | | **z-value** | | **p** | | **Lower** | | **Upper** | |
| Self |  | ↔ |  | Material |  | 0.986 |  | 0.008 |  | 124.338 |  | < .001 |  | 0.970 |  | 1.001 |  |
| Self |  | ↔ |  | Social |  | 0.962 |  | 0.009 |  | 110.612 |  | < .001 |  | 0.945 |  | 0.979 |  |
| Self |  | ↔ |  | Spiritual |  | 0.979 |  | 0.008 |  | 120.334 |  | < .001 |  | 0.963 |  | 0.995 |  |
| Material |  | ↔ |  | Social |  | 0.946 |  | 0.009 |  | 103.590 |  | < .001 |  | 0.928 |  | 0.964 |  |
| Material |  | ↔ |  | Spiritual |  | 0.954 |  | 0.009 |  | 106.480 |  | < .001 |  | 0.936 |  | 0.972 |  |
| Social |  | ↔ |  | Spiritual |  | 0.937 |  | 0.010 |  | 98.104 |  | < .001 |  | 0.918 |  | 0.955 |  |
|  | | | | | | | | | | | | | | | | | |

| **Residual variances** | | | | | | | | | | | | | |
| --- | --- | --- | --- | --- | --- | --- | --- | --- | --- | --- | --- | --- | --- |
|  | | | | | | | | | | **95% Confidence Interval** | | | |
| **Indicator** | | **Estimate** | | **Std. Error** | | **z-value** | | **p** | | **Lower** | | **Upper** | |
| A1Nx |  | 88703.268 |  | 4067.447 |  | 21.808 |  | < .001 |  | 80731.219 |  | 96675.317 |  |
| A1Ny |  | 83773.206 |  | 3837.532 |  | 21.830 |  | < .001 |  | 76251.782 |  | 91294.629 |  |
| A1Nz |  | 95811.731 |  | 4385.290 |  | 21.848 |  | < .001 |  | 87216.722 |  | 104406.741 |  |
| A2Nx |  | 80913.333 |  | 3705.365 |  | 21.837 |  | < .001 |  | 73650.951 |  | 88175.715 |  |
| A2Ny |  | 102939.991 |  | 4661.898 |  | 22.081 |  | < .001 |  | 93802.839 |  | 112077.143 |  |
| A2Nz |  | 85692.497 |  | 3889.259 |  | 22.033 |  | < .001 |  | 78069.689 |  | 93315.306 |  |
| A3Nx |  | 86567.632 |  | 3949.862 |  | 21.917 |  | < .001 |  | 78826.046 |  | 94309.219 |  |
| A3Ny |  | 90795.584 |  | 4145.652 |  | 21.901 |  | < .001 |  | 82670.255 |  | 98920.913 |  |
| A3Nz |  | 103514.914 |  | 4672.727 |  | 22.153 |  | < .001 |  | 94356.537 |  | 112673.290 |  |
| B1Nx |  | 96381.823 |  | 4405.786 |  | 21.876 |  | < .001 |  | 87746.641 |  | 105017.004 |  |
| B1Ny |  | 94662.892 |  | 4357.157 |  | 21.726 |  | < .001 |  | 86123.021 |  | 103202.764 |  |
| B1Nz |  | 84350.811 |  | 3909.253 |  | 21.577 |  | < .001 |  | 76688.815 |  | 92012.806 |  |
| B2Nx |  | 98832.278 |  | 4537.054 |  | 21.783 |  | < .001 |  | 89939.817 |  | 107724.740 |  |
| B2Ny |  | 99985.863 |  | 4608.713 |  | 21.695 |  | < .001 |  | 90952.950 |  | 109018.775 |  |
| B2Nz |  | 90079.200 |  | 4160.708 |  | 21.650 |  | < .001 |  | 81924.362 |  | 98234.039 |  |
| B3Nx |  | 79441.829 |  | 3735.782 |  | 21.265 |  | < .001 |  | 72119.831 |  | 86763.827 |  |
| B3Ny |  | 83561.054 |  | 3914.424 |  | 21.347 |  | < .001 |  | 75888.924 |  | 91233.183 |  |
| B3Nz |  | 84057.219 |  | 3871.968 |  | 21.709 |  | < .001 |  | 76468.301 |  | 91646.137 |  |
| C1Nx |  | 78437.066 |  | 3674.574 |  | 21.346 |  | < .001 |  | 71235.032 |  | 85639.100 |  |
| C1Ny |  | 81867.727 |  | 3812.277 |  | 21.475 |  | < .001 |  | 74395.800 |  | 89339.653 |  |
| C1Nz |  | 72970.885 |  | 3416.548 |  | 21.358 |  | < .001 |  | 66274.575 |  | 79667.195 |  |
| C2Nx |  | 98656.434 |  | 4600.495 |  | 21.445 |  | < .001 |  | 89639.629 |  | 107673.238 |  |
| C2Ny |  | 97296.886 |  | 4489.095 |  | 21.674 |  | < .001 |  | 88498.421 |  | 106095.352 |  |
| C2Nz |  | 97735.659 |  | 4539.616 |  | 21.530 |  | < .001 |  | 88838.176 |  | 106633.143 |  |
| C3Nx |  | 92838.174 |  | 4372.264 |  | 21.233 |  | < .001 |  | 84268.694 |  | 101407.653 |  |
| C3Ny |  | 83591.771 |  | 3983.211 |  | 20.986 |  | < .001 |  | 75784.821 |  | 91398.722 |  |
| C3Nz |  | 96702.154 |  | 4536.323 |  | 21.317 |  | < .001 |  | 87811.124 |  | 105593.185 |  |
| D1Nx |  | 82815.676 |  | 3886.867 |  | 21.307 |  | < .001 |  | 75197.556 |  | 90433.795 |  |
| D1Ny |  | 90063.753 |  | 4139.882 |  | 21.755 |  | < .001 |  | 81949.734 |  | 98177.773 |  |
| D1Nz |  | 84382.453 |  | 3930.746 |  | 21.467 |  | < .001 |  | 76678.332 |  | 92086.575 |  |
| D2Nx |  | 90666.045 |  | 4230.931 |  | 21.429 |  | < .001 |  | 82373.572 |  | 98958.519 |  |
| D2Ny |  | 79901.264 |  | 3730.530 |  | 21.418 |  | < .001 |  | 72589.559 |  | 87212.968 |  |
| D2Nz |  | 75517.059 |  | 3546.089 |  | 21.296 |  | < .001 |  | 68566.853 |  | 82467.265 |  |
| D3Nx |  | 88363.145 |  | 4102.761 |  | 21.537 |  | < .001 |  | 80321.881 |  | 96404.410 |  |
| D3Ny |  | 101745.840 |  | 4646.458 |  | 21.898 |  | < .001 |  | 92638.951 |  | 110852.730 |  |
| D3Nz |  | 99695.763 |  | 4598.965 |  | 21.678 |  | < .001 |  | 90681.958 |  | 108709.569 |  |
|  | | | | | | | | | | | | | |

**Table 9.2.2 CFA Self domain prevention motivation (“A negative”)**

**Model fit**

| **Chi-square test** | | | | | | | |
| --- | --- | --- | --- | --- | --- | --- | --- |
| **Model** | | **Χ²** | | **df** | | **p** | |
| Baseline model |  | 581.959 |  | 28 |  |  |  |
| Factor model |  | 40.735 |  | 17 |  | 0.001 |  |
|  | | | | | | | |

**Additional fit measures**

| **Fit indices** | | | |
| --- | --- | --- | --- |
| **Index** | | **Value** | |
| Comparative Fit Index (CFI) |  | 0.957 |  |
| Tucker-Lewis Index (TLI) |  | 0.929 |  |
| Bentler-Bonett Non-normed Fit Index (NNFI) |  | 0.929 |  |
| Bentler-Bonett Normed Fit Index (NFI) |  | 0.930 |  |
| Parsimony Normed Fit Index (PNFI) |  | 0.565 |  |
| Bollen's Relative Fit Index (RFI) |  | 0.885 |  |
| Bollen's Incremental Fit Index (IFI) |  | 0.958 |  |
| Relative Noncentrality Index (RNI) |  | 0.957 |  |
|  | | | |

| **Information criteria** | | | |
| --- | --- | --- | --- |
|  | | **Value** | |
| Log-likelihood |  | -62478.639 |  |
| Number of free parameters |  | 19.000 |  |
| Akaike (AIC) |  | 124995.278 |  |
| Bayesian (BIC) |  | 125089.106 |  |
| Sample-size adjusted Bayesian (SSABIC) |  | 125028.759 |  |
|  | | | |

| **Other fit measures** | | | |
| --- | --- | --- | --- |
| **Metric** | | **Value** | |
| Root mean square error of approximation (RMSEA) |  | 0.037 |  |
| RMSEA 90% CI lower bound |  | 0.022 |  |
| RMSEA 90% CI upper bound |  | 0.051 |  |
| RMSEA p-value |  | 0.929 |  |
| Standardized root mean square residual (SRMR) |  | 0.033 |  |
| Hoelter's critical N (α = .05) |  | 699.233 |  |
| Hoelter's critical N (α = .01) |  | 846.577 |  |
| Goodness of fit index (GFI) |  | 0.990 |  |
| McDonald fit index (MFI) |  | 0.989 |  |
| Expected cross validation index (ECVI) |  | 0.076 |  |
|  | | | |

| **R-Squared** | | | |
| --- | --- | --- | --- |
|  | | **R²** | |
| A1Nx |  | 0.232 |  |
| A1Ny |  | 0.181 |  |
| A1Nz |  | 0.253 |  |
| A2Nx |  | 0.387 |  |
| A2Ny |  | 0.070 |  |
| A2Nz |  | 0.263 |  |
| A3Ny |  | 0.156 |  |
| A3Nz |  | 0.145 |  |
| Factor 1 |  | 1.000 |  |
| Factor 2 |  | 0.322 |  |
| Factor 3 |  | 1.000 |  |
|  | | | |

**Parameter estimates**

| **Factor loadings** | | | | | | | | | | | | | | | | | |
| --- | --- | --- | --- | --- | --- | --- | --- | --- | --- | --- | --- | --- | --- | --- | --- | --- | --- |
|  | | | | | | | | | | | | | | **95% Confidence Interval** | | | |
| **Factor** | | **Indicator** | | **Symbol** | | **Estimate** | | **Std. Error** | | **z-value** | | **p** | | **Lower** | | **Upper** | |
| Factor 1 |  | A1Nx |  | λ11 |  | 0.161 |  |  |  |  |  |  |  |  |  |  |  |
|  |  | A1Ny |  | λ12 |  | 0.142 |  |  |  |  |  |  |  |  |  |  |  |
|  |  | A1Nz |  | λ13 |  | 0.165 |  |  |  |  |  |  |  |  |  |  |  |
| Factor 2 |  | A2Nx |  | λ21 |  | 231.962 |  |  |  |  |  |  |  |  |  |  |  |
|  |  | A2Ny |  | λ22 |  | 101.758 |  |  |  |  |  |  |  |  |  |  |  |
|  |  | A2Nz |  | λ23 |  | 195.268 |  |  |  |  |  |  |  |  |  |  |  |
| Factor 3 |  | A3Ny |  | λ31 |  | 0.153 |  |  |  |  |  |  |  |  |  |  |  |
|  |  | A3Nz |  | λ32 |  | 0.147 |  |  |  |  |  |  |  |  |  |  |  |
|  | | | | | | | | | | | | | | | | | |

| **Second-order factor loadings** | | | | | | | | | | | | | | | | | |
| --- | --- | --- | --- | --- | --- | --- | --- | --- | --- | --- | --- | --- | --- | --- | --- | --- | --- |
|  | | | | | | | | | | | | | | **95% Confidence Interval** | | | |
| **Factor** | | **Indicator** | | **Symbol** | | **Estimate** | | **Std. Error** | | **z-value** | | **p** | | **Lower** | | **Upper** | |
| SecondOrder |  | Factor 1 |  | γ11 |  | 1522.019 |  |  |  |  |  |  |  |  |  |  |  |
|  |  | Factor 2 |  | γ12 |  | 0.689 |  |  |  |  |  |  |  |  |  |  |  |
|  |  | Factor 3 |  | γ13 |  | 1306.648 |  |  |  |  |  |  |  |  |  |  |  |
|  | | | | | | | | | | | | | | | | | |

| **Factor variances** | | | | | | | | | | | | | |
| --- | --- | --- | --- | --- | --- | --- | --- | --- | --- | --- | --- | --- | --- |
|  | | | | | | | | | | **95% Confidence Interval** | | | |
| **Factor** | | **Estimate** | | **Std. Error** | | **z-value** | | **p** | | **Lower** | | **Upper** | |
| Factor 1 |  | 1.000 |  | 0.000 |  |  |  |  |  | 1.000 |  | 1.000 |  |
| Factor 2 |  | 1.000 |  | 0.000 |  |  |  |  |  | 1.000 |  | 1.000 |  |
| Factor 3 |  | 1.000 |  | 0.000 |  |  |  |  |  | 1.000 |  | 1.000 |  |
| Second-Order |  | 1.000 |  | 0.000 |  |  |  |  |  | 1.000 |  | 1.000 |  |
|  | | | | | | | | | | | | | |

| **Residual variances** | | | | | | | | | | | | | |
| --- | --- | --- | --- | --- | --- | --- | --- | --- | --- | --- | --- | --- | --- |
|  | | | | | | | | | | **95% Confidence Interval** | | | |
| **Indicator** | | **Estimate** | | **Std. Error** | | **z-value** | | **p** | | **Lower** | | **Upper** | |
| A1Nx |  | 199328.625 |  |  |  |  |  |  |  |  |  |  |  |
| A1Ny |  | 210467.800 |  |  |  |  |  |  |  |  |  |  |  |
| A1Nz |  | 186253.089 |  |  |  |  |  |  |  |  |  |  |  |
| A2Nx |  | 125534.461 |  |  |  |  |  |  |  |  |  |  |  |
| A2Ny |  | 201654.851 |  |  |  |  |  |  |  |  |  |  |  |
| A2Nz |  | 157133.467 |  |  |  |  |  |  |  |  |  |  |  |
| A3Ny |  | 214415.459 |  |  |  |  |  |  |  |  |  |  |  |
| A3Nz |  | 215595.503 |  |  |  |  |  |  |  |  |  |  |  |
|  | | | | | | | | | | | | | |

**Table 9.2.3 CFA Material domain prevention motivation (“B negative”)**

**Model fit**

| **Chi-square test** | | | | | | | |
| --- | --- | --- | --- | --- | --- | --- | --- |
| **Model** | | **Χ²** | | **df** | | **p** | |
| Baseline model |  | 822.641 |  | 36 |  |  |  |
| Factor model |  | 31.052 |  | 24 |  | 0.152 |  |
|  | | | | | | | |

**Additional fit measures**

| **Fit indices** | | | |
| --- | --- | --- | --- |
| **Index** | | **Value** | |
| Comparative Fit Index (CFI) |  | 0.991 |  |
| Tucker-Lewis Index (TLI) |  | 0.987 |  |
| Bentler-Bonett Non-normed Fit Index (NNFI) |  | 0.987 |  |
| Bentler-Bonett Normed Fit Index (NFI) |  | 0.962 |  |
| Parsimony Normed Fit Index (PNFI) |  | 0.642 |  |
| Bollen's Relative Fit Index (RFI) |  | 0.943 |  |
| Bollen's Incremental Fit Index (IFI) |  | 0.991 |  |
| Relative Noncentrality Index (RNI) |  | 0.991 |  |
|  | | | |

| **Information criteria** | | | |
| --- | --- | --- | --- |
|  | | **Value** | |
| Log-likelihood |  | -70436.802 |  |
| Number of free parameters |  | 21.000 |  |
| Akaike (AIC) |  | 140915.604 |  |
| Bayesian (BIC) |  | 141019.308 |  |
| Sample-size adjusted Bayesian (SSABIC) |  | 140952.610 |  |
|  | | | |

| **Other fit measures** | | | |
| --- | --- | --- | --- |
| **Metric** | | **Value** | |
| Root mean square error of approximation (RMSEA) |  | 0.017 |  |
| RMSEA 90% CI lower bound |  | 0.000 |  |
| RMSEA 90% CI upper bound |  | 0.032 |  |
| RMSEA p-value |  | 1.000 |  |
| Standardized root mean square residual (SRMR) |  | 0.021 |  |
| Hoelter's critical N (α = .05) |  | 1210.062 |  |
| Hoelter's critical N (α = .01) |  | 1428.028 |  |
| Goodness of fit index (GFI) |  | 0.993 |  |
| McDonald fit index (MFI) |  | 0.997 |  |
| Expected cross validation index (ECVI) |  | 0.071 |  |
|  | | | |

| **R-Squared** | | | |
| --- | --- | --- | --- |
|  | | **R²** | |
| B1Nx |  | 0.199 |  |
| B1Ny |  | 0.197 |  |
| B1Nz |  | 0.322 |  |
| B2Nx |  | 0.256 |  |
| B2Ny |  | 0.239 |  |
| B2Nz |  | 0.245 |  |
| B3Nx |  | 0.294 |  |
| B3Ny |  | 0.128 |  |
| B3Nz |  | 0.184 |  |
| Factor 1 |  | 0.902 |  |
| Factor 2 |  | 0.579 |  |
| Factor 3 |  | 0.771 |  |
|  | | | |

**Table 9.2.4 CFA Social domain prevention motivation (“C negative”)**

**Model fit**

| **Chi-square test** | | | | | | | |
| --- | --- | --- | --- | --- | --- | --- | --- |
| **Model** | | **Χ²** | | **df** | | **p** | |
| Baseline model |  | 1344.779 |  | 36 |  |  |  |
| Factor model |  | 28.684 |  | 24 |  | 0.232 |  |
|  | | | | | | | |

**Additional fit measures**

| **Fit indices** | | | |
| --- | --- | --- | --- |
| **Index** | | **Value** | |
| Comparative Fit Index (CFI) |  | 0.996 |  |
| Tucker-Lewis Index (TLI) |  | 0.995 |  |
| Bentler-Bonett Non-normed Fit Index (NNFI) |  | 0.995 |  |
| Bentler-Bonett Normed Fit Index (NFI) |  | 0.979 |  |
| Parsimony Normed Fit Index (PNFI) |  | 0.652 |  |
| Bollen's Relative Fit Index (RFI) |  | 0.968 |  |
| Bollen's Incremental Fit Index (IFI) |  | 0.996 |  |
| Relative Noncentrality Index (RNI) |  | 0.996 |  |
|  | | | |

| **Information criteria** | | | |
| --- | --- | --- | --- |
|  | | **Value** | |
| Log-likelihood |  | -69947.586 |  |
| Number of free parameters |  | 21.000 |  |
| Akaike (AIC) |  | 139937.172 |  |
| Bayesian (BIC) |  | 140040.876 |  |
| Sample-size adjusted Bayesian (SSABIC) |  | 139974.178 |  |
|  | | | |

| **Other fit measures** | | | |
| --- | --- | --- | --- |
| **Metric** | | **Value** | |
| Root mean square error of approximation (RMSEA) |  | 0.014 |  |
| RMSEA 90% CI lower bound |  | 0.000 |  |
| RMSEA 90% CI upper bound |  | 0.030 |  |
| RMSEA p-value |  | 1.000 |  |
| Standardized root mean square residual (SRMR) |  | 0.019 |  |
| Hoelter's critical N (α = .05) |  | 1309.880 |  |
| Hoelter's critical N (α = .01) |  | 1545.841 |  |
| Goodness of fit index (GFI) |  | 0.994 |  |
| McDonald fit index (MFI) |  | 0.998 |  |
| Expected cross validation index (ECVI) |  | 0.069 |  |
|  | | | |

| **R-Squared** | | | |
| --- | --- | --- | --- |
|  | | **R²** | |
| C1Nx |  | 0.364 |  |
| C1Ny |  | 0.324 |  |
| C1Nz |  | 0.254 |  |
| C2Nx |  | 0.424 |  |
| C2Ny |  | 0.233 |  |
| C2Nz |  | 0.220 |  |
| C3Nx |  | 0.322 |  |
| C3Ny |  | 0.421 |  |
| C3Nz |  | 0.408 |  |
| Factor 1 |  | 0.578 |  |
| Factor 2 |  | 0.542 |  |
| Factor 3 |  | 0.768 |  |
|  | | | |

**Parameter estimates**

| **Factor loadings** | | | | | | | | | | | | | | | | | |
| --- | --- | --- | --- | --- | --- | --- | --- | --- | --- | --- | --- | --- | --- | --- | --- | --- | --- |
|  | | | | | | | | | | | | | | **95% Confidence Interval** | | | |
| **Factor** | | **Indicator** | | **Symbol** | | **Estimate** | | **Std. Error** | | **z-value** | | **p** | | **Lower** | | **Upper** | |
| Factor 1 |  | C1Nx |  | λ11 |  | 193.065 |  | 19.982 |  | 9.662 |  | < .001 |  | 153.902 |  | 232.228 |  |
|  |  | C1Ny |  | λ12 |  | 185.459 |  | 19.357 |  | 9.581 |  | < .001 |  | 147.520 |  | 223.398 |  |
|  |  | C1Nz |  | λ13 |  | 156.823 |  | 17.041 |  | 9.203 |  | < .001 |  | 123.423 |  | 190.222 |  |
| Factor 2 |  | C2Nx |  | λ21 |  | 219.175 |  | 22.139 |  | 9.900 |  | < .001 |  | 175.783 |  | 262.567 |  |
|  |  | C2Ny |  | λ22 |  | 166.273 |  | 17.664 |  | 9.413 |  | < .001 |  | 131.651 |  | 200.894 |  |
|  |  | C2Nz |  | λ23 |  | 152.909 |  | 16.494 |  | 9.271 |  | < .001 |  | 120.582 |  | 185.236 |  |
| Factor 3 |  | C3Nx |  | λ31 |  | 132.158 |  | 23.493 |  | 5.626 |  | < .001 |  | 86.114 |  | 178.203 |  |
|  |  | C3Ny |  | λ32 |  | 149.588 |  | 26.425 |  | 5.661 |  | < .001 |  | 97.796 |  | 201.379 |  |
|  |  | C3Nz |  | λ33 |  | 144.431 |  | 25.508 |  | 5.662 |  | < .001 |  | 94.437 |  | 194.426 |  |
|  | | | | | | | | | | | | | | | | | |

| **Second-order factor loadings** | | | | | | | | | | | | | | | | | |
| --- | --- | --- | --- | --- | --- | --- | --- | --- | --- | --- | --- | --- | --- | --- | --- | --- | --- |
|  | | | | | | | | | | | | | | **95% Confidence Interval** | | | |
| **Factor** | | **Indicator** | | **Symbol** | | **Estimate** | | **Std. Error** | | **z-value** | | **p** | | **Lower** | | **Upper** | |
| SecondOrder |  | Factor 1 |  | γ11 |  | 1.170 |  | 0.159 |  | 7.371 |  | < .001 |  | 0.859 |  | 1.481 |  |
|  |  | Factor 2 |  | γ12 |  | 1.089 |  | 0.143 |  | 7.597 |  | < .001 |  | 0.808 |  | 1.370 |  |
|  |  | Factor 3 |  | γ13 |  | 1.820 |  | 0.393 |  | 4.625 |  | < .001 |  | 1.049 |  | 2.591 |  |
|  | | | | | | | | | | | | | | | | | |

| **Factor variances** | | | | | | | | | | | | | |
| --- | --- | --- | --- | --- | --- | --- | --- | --- | --- | --- | --- | --- | --- |
|  | | | | | | | | | | **95% Confidence Interval** | | | |
| **Factor** | | **Estimate** | | **Std. Error** | | **z-value** | | **p** | | **Lower** | | **Upper** | |
| Factor 1 |  | 1.000 |  | 0.000 |  |  |  |  |  | 1.000 |  | 1.000 |  |
| Factor 2 |  | 1.000 |  | 0.000 |  |  |  |  |  | 1.000 |  | 1.000 |  |
| Factor 3 |  | 1.000 |  | 0.000 |  |  |  |  |  | 1.000 |  | 1.000 |  |
| Second-Order |  | 1.000 |  | 0.000 |  |  |  |  |  | 1.000 |  | 1.000 |  |
|  | | | | | | | | | | | | | |

| **Residual variances** | | | | | | | | | | | | | |
| --- | --- | --- | --- | --- | --- | --- | --- | --- | --- | --- | --- | --- | --- |
|  | | | | | | | | | | **95% Confidence Interval** | | | |
| **Indicator** | | **Estimate** | | **Std. Error** | | **z-value** | | **p** | | **Lower** | | **Upper** | |
| C1Nx |  | 154257.304 |  | 9923.345 |  | 15.545 |  | < .001 |  | 134807.906 |  | 173706.702 |  |
| C1Ny |  | 169993.174 |  | 10161.537 |  | 16.729 |  | < .001 |  | 150076.927 |  | 189909.421 |  |
| C1Nz |  | 170674.030 |  | 9222.391 |  | 18.506 |  | < .001 |  | 152598.477 |  | 188749.584 |  |
| C2Nx |  | 142771.255 |  | 11110.242 |  | 12.850 |  | < .001 |  | 120995.580 |  | 164546.929 |  |
| C2Ny |  | 198617.461 |  | 10632.315 |  | 18.681 |  | < .001 |  | 177778.507 |  | 219456.415 |  |
| C2Nz |  | 181172.474 |  | 9540.684 |  | 18.989 |  | < .001 |  | 162473.077 |  | 199871.871 |  |
| C3Nx |  | 158201.591 |  | 8694.974 |  | 18.195 |  | < .001 |  | 141159.756 |  | 175243.426 |  |
| C3Ny |  | 132937.884 |  | 8448.564 |  | 15.735 |  | < .001 |  | 116379.002 |  | 149496.766 |  |
| C3Nz |  | 130733.648 |  | 8120.046 |  | 16.100 |  | < .001 |  | 114818.649 |  | 146648.646 |  |
|  | | | | | | | | | | | | | |

**Table 9.2.5 CFA Spiritual domain prevention motivation (“D negative”)**

**Model fit**

| **Chi-square test** | | | | | | | |
| --- | --- | --- | --- | --- | --- | --- | --- |
| **Model** | | **Χ²** | | **df** | | **p** | |
| Baseline model |  | 961.011 |  | 36 |  |  |  |
| Factor model |  | 35.711 |  | 24 |  | 0.059 |  |
|  | | | | | | | |

**Additional fit measures**

| **Fit indices** | | | |
| --- | --- | --- | --- |
| **Index** | | **Value** | |
| Comparative Fit Index (CFI) |  | 0.987 |  |
| Tucker-Lewis Index (TLI) |  | 0.981 |  |
| Bentler-Bonett Non-normed Fit Index (NNFI) |  | 0.981 |  |
| Bentler-Bonett Normed Fit Index (NFI) |  | 0.963 |  |
| Parsimony Normed Fit Index (PNFI) |  | 0.642 |  |
| Bollen's Relative Fit Index (RFI) |  | 0.944 |  |
| Bollen's Incremental Fit Index (IFI) |  | 0.988 |  |
| Relative Noncentrality Index (RNI) |  | 0.987 |  |
|  | | | |

| **Information criteria** | | | |
| --- | --- | --- | --- |
|  | | **Value** | |
| Log-likelihood |  | -70535.527 |  |
| Number of free parameters |  | 21.000 |  |
| Akaike (AIC) |  | 141113.054 |  |
| Bayesian (BIC) |  | 141216.758 |  |
| Sample-size adjusted Bayesian (SSABIC) |  | 141150.059 |  |
|  | | | |

| **Other fit measures** | | | |
| --- | --- | --- | --- |
| **Metric** | | **Value** | |
| Root mean square error of approximation (RMSEA) |  | 0.022 |  |
| RMSEA 90% CI lower bound |  | 0.000 |  |
| RMSEA 90% CI upper bound |  | 0.036 |  |
| RMSEA p-value |  | 1.000 |  |
| Standardized root mean square residual (SRMR) |  | 0.023 |  |
| Hoelter's critical N (α = .05) |  | 1052.324 |  |
| Hoelter's critical N (α = .01) |  | 1241.854 |  |
| Goodness of fit index (GFI) |  | 0.992 |  |
| McDonald fit index (MFI) |  | 0.994 |  |
| Expected cross validation index (ECVI) |  | 0.075 |  |
|  | | | |

| **R-Squared** | | | |
| --- | --- | --- | --- |
|  | | **R²** | |
| D1Nx |  | 0.241 |  |
| D1Ny |  | 0.324 |  |
| D1Nz |  | 0.193 |  |
| D2Nx |  | 0.304 |  |
| D2Ny |  | 0.308 |  |
| D2Nz |  | 0.162 |  |
| D3Nx |  | 0.340 |  |
| D3Ny |  | 0.204 |  |
| D3Nz |  | 0.282 |  |
| Factor 1 |  | 0.864 |  |
| Factor 2 |  | 0.627 |  |
| Factor 3 |  | 0.582 |  |
|  | | | |

**Parameter estimates**

| **Factor loadings** | | | | | | | | | | | | | | | | | |
| --- | --- | --- | --- | --- | --- | --- | --- | --- | --- | --- | --- | --- | --- | --- | --- | --- | --- |
|  | | | | | | | | | | | | | | **95% Confidence Interval** | | | |
| **Factor** | | **Indicator** | | **Symbol** | | **Estimate** | | **Std. Error** | | **z-value** | | **p** | | **Lower** | | **Upper** | |
| Factor 1 |  | D1Nx |  | λ11 |  | 92.120 |  | 35.396 |  | 2.603 |  | 0.009 |  | 22.746 |  | 161.495 |  |
|  |  | D1Ny |  | λ12 |  | 110.471 |  | 42.713 |  | 2.586 |  | 0.010 |  | 26.754 |  | 194.187 |  |
|  |  | D1Nz |  | λ13 |  | 81.344 |  | 31.309 |  | 2.598 |  | 0.009 |  | 19.980 |  | 142.708 |  |
| Factor 2 |  | D2Nx |  | λ21 |  | 163.079 |  | 21.823 |  | 7.473 |  | < .001 |  | 120.308 |  | 205.851 |  |
|  |  | D2Ny |  | λ22 |  | 171.278 |  | 22.921 |  | 7.473 |  | < .001 |  | 126.354 |  | 216.202 |  |
|  |  | D2Nz |  | λ23 |  | 119.606 |  | 17.388 |  | 6.879 |  | < .001 |  | 85.526 |  | 153.685 |  |
| Factor 3 |  | D3Nx |  | λ31 |  | 198.091 |  | 23.100 |  | 8.575 |  | < .001 |  | 152.817 |  | 243.366 |  |
|  |  | D3Ny |  | λ32 |  | 156.883 |  | 19.495 |  | 8.047 |  | < .001 |  | 118.673 |  | 195.092 |  |
|  |  | D3Nz |  | λ33 |  | 174.385 |  | 20.495 |  | 8.509 |  | < .001 |  | 134.216 |  | 214.555 |  |
|  | | | | | | | | | | | | | | | | | |

| **Second-order factor loadings** | | | | | | | | | | | | | | | | | |
| --- | --- | --- | --- | --- | --- | --- | --- | --- | --- | --- | --- | --- | --- | --- | --- | --- | --- |
|  | | | | | | | | | | | | | | **95% Confidence Interval** | | | |
| **Factor** | | **Indicator** | | **Symbol** | | **Estimate** | | **Std. Error** | | **z-value** | | **p** | | **Lower** | | **Upper** | |
| SecondOrder |  | Factor 1 |  | γ11 |  | 2.521 |  | 1.069 |  | 2.357 |  | 0.018 |  | 0.425 |  | 4.617 |  |
|  |  | Factor 2 |  | γ12 |  | 1.297 |  | 0.216 |  | 6.008 |  | < .001 |  | 0.874 |  | 1.720 |  |
|  |  | Factor 3 |  | γ13 |  | 1.180 |  | 0.176 |  | 6.702 |  | < .001 |  | 0.835 |  | 1.525 |  |
|  | | | | | | | | | | | | | | | | | |

| **Factor variances** | | | | | | | | | | | | | |
| --- | --- | --- | --- | --- | --- | --- | --- | --- | --- | --- | --- | --- | --- |
|  | | | | | | | | | | **95% Confidence Interval** | | | |
| **Factor** | | **Estimate** | | **Std. Error** | | **z-value** | | **p** | | **Lower** | | **Upper** | |
| Factor 1 |  | 1.000 |  | 0.000 |  |  |  |  |  | 1.000 |  | 1.000 |  |
| Factor 2 |  | 1.000 |  | 0.000 |  |  |  |  |  | 1.000 |  | 1.000 |  |
| Factor 3 |  | 1.000 |  | 0.000 |  |  |  |  |  | 1.000 |  | 1.000 |  |
| Second-Order |  | 1.000 |  | 0.000 |  |  |  |  |  | 1.000 |  | 1.000 |  |
|  | | | | | | | | | | | | | |

| **Residual variances** | | | | | | | | | | | | | |
| --- | --- | --- | --- | --- | --- | --- | --- | --- | --- | --- | --- | --- | --- |
|  | | | | | | | | | | **95% Confidence Interval** | | | |
| **Indicator** | | **Estimate** | | **Std. Error** | | **z-value** | | **p** | | **Lower** | | **Upper** | |
| D1Nx |  | 196924.229 |  | 10727.100 |  | 18.358 |  | < .001 |  | 175899.499 |  | 217948.960 |  |
| D1Ny |  | 187629.067 |  | 11791.199 |  | 15.913 |  | < .001 |  | 164518.742 |  | 210739.392 |  |
| D1Nz |  | 203697.711 |  | 10457.427 |  | 19.479 |  | < .001 |  | 183201.531 |  | 224193.891 |  |
| D2Nx |  | 163081.036 |  | 10106.470 |  | 16.136 |  | < .001 |  | 143272.719 |  | 182889.353 |  |
| D2Ny |  | 176560.211 |  | 11030.124 |  | 16.007 |  | < .001 |  | 154941.565 |  | 198178.857 |  |
| D2Nz |  | 197666.453 |  | 9937.648 |  | 19.891 |  | < .001 |  | 178189.020 |  | 217143.885 |  |
| D3Nx |  | 182199.632 |  | 12008.307 |  | 15.173 |  | < .001 |  | 158663.782 |  | 205735.481 |  |
| D3Ny |  | 230201.463 |  | 12078.992 |  | 19.058 |  | < .001 |  | 206527.073 |  | 253875.853 |  |
| D3Nz |  | 184800.068 |  | 10878.636 |  | 16.987 |  | < .001 |  | 163478.333 |  | 206121.802 |  |
|  | | | | | | | | | | | | | |
