# Supplementary material for: The structure of human motivation
Source: BMC Psychol. 2023 Oct 6;11:308. doi: 10.1186/s40359-023-01346-5 (PMC10557177; doi:10.1186/s40359-023-01346-5)
Supplement: Supplementary file 5 — Additional file 5: SM Table 10. Confirmatory factor models for the three levels of attainment: Full output. [file 40359_2023_1346_MOESM5_ESM.zip › Table 10.2.CFA.levels of attainment.negativeR5.docx]

**Table 10.2 Level of attainment (negative)**

**Table 10.2.1 CFA Overall prevention motivation model (levels of attainment)**

### Model fit

| **Chi-square test** | | | | | | | |
| --- | --- | --- | --- | --- | --- | --- | --- |
| **Model** | | **Χ²** | | **df** | | **p** | |
| Baseline model |  | 16790.248 |  | 595 |  |  |  |
| Factor model |  | 1330.930 |  | 557 |  | < .001 |  |
|  | | | | | | | |

#### Additional fit measures

| **Fit indices** | | | |
| --- | --- | --- | --- |
| **Index** | | **Value** | |
| Comparative Fit Index (CFI) |  | 0.952 |  |
| Tucker-Lewis Index (TLI) |  | 0.949 |  |
| Bentler-Bonett Non-normed Fit Index (NNFI) |  | 0.949 |  |
| Bentler-Bonett Normed Fit Index (NFI) |  | 0.921 |  |
| Parsimony Normed Fit Index (PNFI) |  | 0.862 |  |
| Bollen's Relative Fit Index (RFI) |  | 0.915 |  |
| Bollen's Incremental Fit Index (IFI) |  | 0.952 |  |
| Relative Noncentrality Index (RNI) |  | 0.952 |  |
|  | | | |

| **Information criteria** | | | |
| --- | --- | --- | --- |
|  | | **Value** | |
| Log-likelihood |  | -258904.172 |  |
| Number of free parameters |  | 73.000 |  |
| Akaike (AIC) |  | 517954.345 |  |
| Bayesian (BIC) |  | 518314.840 |  |
| Sample-size adjusted Bayesian (SSABIC) |  | 518082.983 |  |
|  | | | |

| **Other fit measures** | | | |
| --- | --- | --- | --- |
| **Metric** | | **Value** | |
| Root mean square error of approximation (RMSEA) |  | 0.037 |  |
| RMSEA 90% CI lower bound |  | 0.034 |  |
| RMSEA 90% CI upper bound |  | 0.039 |  |
| RMSEA p-value |  | 1.000 |  |
| Standardized root mean square residual (SRMR) |  | 0.028 |  |
| Hoelter's critical N (α = .05) |  | 475.868 |  |
| Hoelter's critical N (α = .01) |  | 494.894 |  |
| Goodness of fit index (GFI) |  | 0.927 |  |
| McDonald fit index (MFI) |  | 0.687 |  |
| Expected cross validation index (ECVI) |  | 1.433 |  |
|  | | | |

| **R-Squared** | | | |
| --- | --- | --- | --- |
|  | | **R²** | |
| A1Nx |  | 0.426 |  |
| A1Ny |  | 0.426 |  |
| A1Nz |  | 0.420 |  |
| B1Nx |  | 0.353 |  |
| B1Ny |  | 0.385 |  |
| B1Nz |  | 0.421 |  |
| C1Nx |  | 0.435 |  |
| C1Ny |  | 0.404 |  |
| C1Nz |  | 0.441 |  |
| D1Nx |  | 0.445 |  |
| D1Ny |  | 0.359 |  |
| D1Nz |  | 0.423 |  |
| A2Nx |  | 0.425 |  |
| A2Ny |  | 0.351 |  |
| A2Nz |  | 0.367 |  |
| B2Nx |  | 0.370 |  |
| B2Ny |  | 0.393 |  |
| B2Nz |  | 0.407 |  |
| C2Nx |  | 0.404 |  |
| C2Ny |  | 0.378 |  |
| C2Nz |  | 0.393 |  |
| D2Nx |  | 0.448 |  |
| D2Ny |  | 0.435 |  |
| D2Nz |  | 0.461 |  |
| A3Nx |  | 0.400 |  |
| A3Ny |  | 0.403 |  |
| A3Nz |  | 0.326 |  |
| B3Nx |  | 0.475 |  |
| B3Ny |  | 0.465 |  |
| B3Nz |  | 0.397 |  |
| C3Nx |  | 0.430 |  |
| C3Ny |  | 0.472 |  |
| C3Nz |  | 0.421 |  |
| D3Ny |  | 0.316 |  |
| D3Nz |  | 0.364 |  |
|  | | | |

### Parameter estimates

| **Factor loadings** | | | | | | | | | | | | | | | | | |
| --- | --- | --- | --- | --- | --- | --- | --- | --- | --- | --- | --- | --- | --- | --- | --- | --- | --- |
|  | | | | | | | | | | | | | | **95% Confidence Interval** | | | |
| **Factor** | | **Indicator** | | **Symbol** | | **Estimate** | | **Std. Error** | | **z-value** | | **p** | | **Lower** | | **Upper** | |
| Foundational |  | A1Nx |  | λ11 |  | 257.327 |  | 11.107 |  | 23.169 |  | < .001 |  | 235.558 |  | 279.095 |  |
|  |  | A1Ny |  | λ12 |  | 249.056 |  | 10.741 |  | 23.188 |  | < .001 |  | 228.005 |  | 270.108 |  |
|  |  | A1Nz |  | λ13 |  | 263.247 |  | 11.465 |  | 22.961 |  | < .001 |  | 240.775 |  | 285.718 |  |
|  |  | B1Nx |  | λ14 |  | 230.284 |  | 11.186 |  | 20.587 |  | < .001 |  | 208.360 |  | 252.209 |  |
|  |  | B1Ny |  | λ15 |  | 245.683 |  | 11.310 |  | 21.723 |  | < .001 |  | 223.516 |  | 267.850 |  |
|  |  | B1Nz |  | λ16 |  | 249.471 |  | 10.848 |  | 22.997 |  | < .001 |  | 228.209 |  | 270.733 |  |
|  |  | C1Nx |  | λ17 |  | 247.538 |  | 10.546 |  | 23.472 |  | < .001 |  | 226.868 |  | 268.208 |  |
|  |  | C1Ny |  | λ18 |  | 238.794 |  | 10.658 |  | 22.406 |  | < .001 |  | 217.906 |  | 259.683 |  |
|  |  | C1Nz |  | λ19 |  | 240.190 |  | 10.129 |  | 23.714 |  | < .001 |  | 220.338 |  | 260.043 |  |
|  |  | D1Nx |  | λ110 |  | 262.648 |  | 11.014 |  | 23.847 |  | < .001 |  | 241.061 |  | 284.235 |  |
|  |  | D1Ny |  | λ111 |  | 227.482 |  | 10.926 |  | 20.820 |  | < .001 |  | 206.067 |  | 248.897 |  |
|  |  | D1Nz |  | λ112 |  | 251.815 |  | 10.908 |  | 23.086 |  | < .001 |  | 230.436 |  | 273.194 |  |
| Experiential |  | A2Nx |  | λ21 |  | 244.078 |  | 10.548 |  | 23.140 |  | < .001 |  | 223.405 |  | 264.751 |  |
|  |  | A2Ny |  | λ22 |  | 236.174 |  | 11.498 |  | 20.540 |  | < .001 |  | 213.638 |  | 258.710 |  |
|  |  | A2Nz |  | λ23 |  | 222.937 |  | 10.567 |  | 21.098 |  | < .001 |  | 202.226 |  | 243.648 |  |
|  |  | B2Nx |  | λ24 |  | 243.459 |  | 11.484 |  | 21.200 |  | < .001 |  | 220.952 |  | 265.967 |  |
|  |  | B2Ny |  | λ25 |  | 256.829 |  | 11.664 |  | 22.019 |  | < .001 |  | 233.967 |  | 279.690 |  |
|  |  | B2Nz |  | λ26 |  | 250.015 |  | 11.117 |  | 22.489 |  | < .001 |  | 228.226 |  | 271.804 |  |
|  |  | C2Nx |  | λ27 |  | 263.552 |  | 11.760 |  | 22.410 |  | < .001 |  | 240.502 |  | 286.602 |  |
|  |  | C2Ny |  | λ28 |  | 243.292 |  | 11.323 |  | 21.486 |  | < .001 |  | 221.099 |  | 265.485 |  |
|  |  | C2Nz |  | λ29 |  | 254.851 |  | 11.585 |  | 21.999 |  | < .001 |  | 232.145 |  | 277.556 |  |
|  |  | D2Nx |  | λ210 |  | 270.092 |  | 11.291 |  | 23.921 |  | < .001 |  | 247.962 |  | 292.222 |  |
|  |  | D2Ny |  | λ211 |  | 250.293 |  | 10.666 |  | 23.467 |  | < .001 |  | 229.389 |  | 271.198 |  |
|  |  | D2Nz |  | λ212 |  | 255.484 |  | 10.485 |  | 24.367 |  | < .001 |  | 234.934 |  | 276.034 |  |
| Aspirational |  | A3Nx |  | λ31 |  | 240.659 |  | 10.778 |  | 22.328 |  | < .001 |  | 219.534 |  | 261.784 |  |
|  |  | A3Ny |  | λ32 |  | 248.026 |  | 11.069 |  | 22.408 |  | < .001 |  | 226.331 |  | 269.720 |  |
|  |  | A3Nz |  | λ33 |  | 223.722 |  | 11.373 |  | 19.672 |  | < .001 |  | 201.432 |  | 246.012 |  |
|  |  | B3Nx |  | λ34 |  | 271.112 |  | 10.879 |  | 24.922 |  | < .001 |  | 249.791 |  | 292.434 |  |
|  |  | B3Ny |  | λ35 |  | 271.659 |  | 11.046 |  | 24.593 |  | < .001 |  | 250.008 |  | 293.309 |  |
|  |  | B3Nz |  | λ36 |  | 235.894 |  | 10.628 |  | 22.197 |  | < .001 |  | 215.065 |  | 256.724 |  |
|  |  | C3Nx |  | λ37 |  | 272.598 |  | 11.670 |  | 23.358 |  | < .001 |  | 249.724 |  | 295.471 |  |
|  |  | C3Ny |  | λ38 |  | 281.075 |  | 11.322 |  | 24.826 |  | < .001 |  | 258.885 |  | 303.265 |  |
|  |  | C3Nz |  | λ39 |  | 271.617 |  | 11.793 |  | 23.032 |  | < .001 |  | 248.503 |  | 294.732 |  |
|  |  | D3Ny |  | λ310 |  | 220.652 |  | 11.416 |  | 19.328 |  | < .001 |  | 198.277 |  | 243.027 |  |
|  |  | D3Nz |  | λ311 |  | 244.575 |  | 11.619 |  | 21.050 |  | < .001 |  | 221.803 |  | 267.348 |  |
|  | | | | | | | | | | | | | | | | | |

| **Factor variances** | | | | | | | | | | | | | |
| --- | --- | --- | --- | --- | --- | --- | --- | --- | --- | --- | --- | --- | --- |
|  | | | | | | | | | | **95% Confidence Interval** | | | |
| **Factor** | | **Estimate** | | **Std. Error** | | **z-value** | | **p** | | **Lower** | | **Upper** | |
| Foundational |  | 1.000 |  | 0.000 |  |  |  |  |  | 1.000 |  | 1.000 |  |
| Experiential |  | 1.000 |  | 0.000 |  |  |  |  |  | 1.000 |  | 1.000 |  |
| Aspirational |  | 1.000 |  | 0.000 |  |  |  |  |  | 1.000 |  | 1.000 |  |
|  | | | | | | | | | | | | | |

| **Factor Covariances** | | | | | | | | | | | | | | | | | |
| --- | --- | --- | --- | --- | --- | --- | --- | --- | --- | --- | --- | --- | --- | --- | --- | --- | --- |
|  | | | | | | | | | | | | | | **95% Confidence Interval** | | | |
|  | |  | |  | | **Estimate** | | **Std. Error** | | **z-value** | | **p** | | **Lower** | | **Upper** | |
| Foundational |  | ↔ |  | Experiential |  | 0.984 |  | 0.006 |  | 160.658 |  | < .001 |  | 0.972 |  | 0.996 |  |
| Foundational |  | ↔ |  | Aspirational |  | 0.995 |  | 0.006 |  | 171.141 |  | < .001 |  | 0.984 |  | 1.007 |  |
| Experiential |  | ↔ |  | Aspirational |  | 0.995 |  | 0.006 |  | 167.785 |  | < .001 |  | 0.983 |  | 1.006 |  |
|  | | | | | | | | | | | | | | | | | |

| **Residual variances** | | | | | | | | | | | | | |
| --- | --- | --- | --- | --- | --- | --- | --- | --- | --- | --- | --- | --- | --- |
|  | | | | | | | | | | **95% Confidence Interval** | | | |
| **Indicator** | | **Estimate** | | **Std. Error** | | **z-value** | | **p** | | **Lower** | | **Upper** | |
| A1Nx |  | 89276.981 |  | 4085.700 |  | 21.851 |  | < .001 |  | 81269.157 |  | 97284.805 |  |
| A1Ny |  | 83446.340 |  | 3819.206 |  | 21.849 |  | < .001 |  | 75960.833 |  | 90931.847 |  |
| A1Nz |  | 95747.153 |  | 4377.512 |  | 21.873 |  | < .001 |  | 87167.387 |  | 104326.920 |  |
| B1Nx |  | 97398.594 |  | 4410.285 |  | 22.084 |  | < .001 |  | 88754.594 |  | 106042.594 |  |
| B1Ny |  | 96594.452 |  | 4392.680 |  | 21.990 |  | < .001 |  | 87984.956 |  | 105203.947 |  |
| B1Nz |  | 85623.089 |  | 3915.305 |  | 21.869 |  | < .001 |  | 77949.232 |  | 93296.945 |  |
| C1Nx |  | 79733.502 |  | 3654.312 |  | 21.819 |  | < .001 |  | 72571.181 |  | 86895.822 |  |
| C1Ny |  | 84116.281 |  | 3836.181 |  | 21.927 |  | < .001 |  | 76597.504 |  | 91635.057 |  |
| C1Nz |  | 72983.100 |  | 3348.978 |  | 21.793 |  | < .001 |  | 66419.224 |  | 79546.976 |  |
| D1Nx |  | 85921.560 |  | 3945.383 |  | 21.778 |  | < .001 |  | 78188.752 |  | 93654.368 |  |
| D1Ny |  | 92364.648 |  | 4185.851 |  | 22.066 |  | < .001 |  | 84160.531 |  | 100568.766 |  |
| D1Nz |  | 86330.073 |  | 3949.284 |  | 21.860 |  | < .001 |  | 78589.617 |  | 94070.528 |  |
| A2Nx |  | 80518.558 |  | 3686.846 |  | 21.839 |  | < .001 |  | 73292.473 |  | 87744.644 |  |
| A2Ny |  | 102946.410 |  | 4662.834 |  | 22.078 |  | < .001 |  | 93807.422 |  | 112085.397 |  |
| A2Nz |  | 85694.668 |  | 3889.465 |  | 22.033 |  | < .001 |  | 78071.457 |  | 93317.879 |  |
| B2Nx |  | 100933.115 |  | 4582.910 |  | 22.024 |  | < .001 |  | 91950.777 |  | 109915.453 |  |
| B2Ny |  | 101800.323 |  | 4637.647 |  | 21.951 |  | < .001 |  | 92710.702 |  | 110889.944 |  |
| B2Nz |  | 91224.142 |  | 4164.378 |  | 21.906 |  | < .001 |  | 83062.112 |  | 99386.173 |  |
| C2Nx |  | 102320.873 |  | 4669.301 |  | 21.914 |  | < .001 |  | 93169.212 |  | 111472.534 |  |
| C2Ny |  | 97374.112 |  | 4426.283 |  | 21.999 |  | < .001 |  | 88698.757 |  | 106049.468 |  |
| C2Nz |  | 100472.830 |  | 4576.791 |  | 21.953 |  | < .001 |  | 91502.484 |  | 109443.175 |  |
| D2Nx |  | 90003.585 |  | 4137.526 |  | 21.753 |  | < .001 |  | 81894.183 |  | 98112.988 |  |
| D2Ny |  | 81491.204 |  | 3737.413 |  | 21.804 |  | < .001 |  | 74166.008 |  | 88816.399 |  |
| D2Nz |  | 76459.209 |  | 3523.490 |  | 21.700 |  | < .001 |  | 69553.295 |  | 83365.124 |  |
| A3Nx |  | 86708.890 |  | 3937.908 |  | 22.019 |  | < .001 |  | 78990.733 |  | 94427.047 |  |
| A3Ny |  | 91231.740 |  | 4144.617 |  | 22.012 |  | < .001 |  | 83108.440 |  | 99355.040 |  |
| A3Nz |  | 103568.368 |  | 4661.669 |  | 22.217 |  | < .001 |  | 94431.665 |  | 112705.070 |  |
| B3Nx |  | 81257.754 |  | 3735.025 |  | 21.756 |  | < .001 |  | 73937.239 |  | 88578.269 |  |
| B3Ny |  | 84752.131 |  | 3888.798 |  | 21.794 |  | < .001 |  | 77130.227 |  | 92374.035 |  |
| B3Nz |  | 84623.375 |  | 3841.223 |  | 22.030 |  | < .001 |  | 77094.716 |  | 92152.034 |  |
| C3Nx |  | 98532.439 |  | 4494.208 |  | 21.924 |  | < .001 |  | 89723.954 |  | 107340.923 |  |
| C3Ny |  | 88308.002 |  | 4056.989 |  | 21.767 |  | < .001 |  | 80356.449 |  | 96259.555 |  |
| C3Nz |  | 101645.336 |  | 4629.583 |  | 21.956 |  | < .001 |  | 92571.521 |  | 110719.152 |  |
| D3Ny |  | 105212.109 |  | 4731.056 |  | 22.239 |  | < .001 |  | 95939.409 |  | 114484.809 |  |
| D3Nz |  | 104409.808 |  | 4719.783 |  | 22.122 |  | < .001 |  | 95159.203 |  | 113660.412 |  |
|  | | | | | | | | | | | | | |

**Table 10.2.2 Foundational negative**

**Model fit**

| **Chi-square test** | | | | | | | |
| --- | --- | --- | --- | --- | --- | --- | --- |
| **Model** | | **Χ²** | | **df** | | **p** | |
| Baseline model |  | 1429.370 |  | 66 |  |  |  |
| Factor model |  | 74.505 |  | 50 |  | 0.014 |  |
|  | | | | | | | |

**Additional fit measures**

| **Fit indices** | | | |
| --- | --- | --- | --- |
| **Index** | | **Value** | |
| Comparative Fit Index (CFI) |  | 0.982 |  |
| Tucker-Lewis Index (TLI) |  | 0.976 |  |
| Bentler-Bonett Non-normed Fit Index (NNFI) |  | 0.976 |  |
| Bentler-Bonett Normed Fit Index (NFI) |  | 0.948 |  |
| Parsimony Normed Fit Index (PNFI) |  | 0.718 |  |
| Bollen's Relative Fit Index (RFI) |  | 0.931 |  |
| Bollen's Incremental Fit Index (IFI) |  | 0.982 |  |
| Relative Noncentrality Index (RNI) |  | 0.982 |  |
|  | | | |

| **Information criteria** | | | |
| --- | --- | --- | --- |
|  | | **Value** | |
| Log-likelihood |  | -93886.637 |  |
| Number of free parameters |  | 28.000 |  |
| Akaike (AIC) |  | 187829.274 |  |
| Bayesian (BIC) |  | 187967.546 |  |
| Sample-size adjusted Bayesian (SSABIC) |  | 187878.615 |  |
|  | | | |

| **Other fit measures** | | | |
| --- | --- | --- | --- |
| **Metric** | | **Value** | |
| Root mean square error of approximation (RMSEA) |  | 0.022 |  |
| RMSEA 90% CI lower bound |  | 0.010 |  |
| RMSEA 90% CI upper bound |  | 0.032 |  |
| RMSEA p-value |  | 1.000 |  |
| Standardized root mean square residual (SRMR) |  | 0.026 |  |
| Hoelter's critical N (α = .05) |  | 935.126 |  |
| Hoelter's critical N (α = .01) |  | 1054.812 |  |
| Goodness of fit index (GFI) |  | 0.988 |  |
| McDonald fit index (MFI) |  | 0.988 |  |
| Expected cross validation index (ECVI) |  | 0.127 |  |
|  | | | |

| **R-Squared** | | | |
| --- | --- | --- | --- |
|  | | **R²** | |
| D1Nx |  | 0.283 |  |
| D1Ny |  | 0.238 |  |
| D1Nz |  | 0.221 |  |
| C1Nx |  | 0.334 |  |
| C1Ny |  | 0.312 |  |
| C1Nz |  | 0.295 |  |
| B1Nx |  | 0.212 |  |
| B1Ny |  | 0.207 |  |
| B1Nz |  | 0.295 |  |
| A1Nx |  | 0.206 |  |
| A1Ny |  | 0.222 |  |
| A1Nz |  | 0.226 |  |
| Factor 1 |  | 0.638 |  |
| Factor 2 |  | 0.585 |  |
| Factor 3 |  | 0.727 |  |
|  | | | |

**Parameter estimates**

| **Factor loadings** | | | | | | | | | | | | | | | | | |
| --- | --- | --- | --- | --- | --- | --- | --- | --- | --- | --- | --- | --- | --- | --- | --- | --- | --- |
|  | | | | | | | | | | | | | | **95% Confidence Interval** | | | |
| **Factor** | | **Indicator** | | **Symbol** | | **Estimate** | | **Std. Error** | | **z-value** | | **p** | | **Lower** | | **Upper** | |
| Factor 1 |  | D1Nx |  | λ11 |  | 163.068 |  | 21.127 |  | 7.718 |  | < .001 |  | 121.659 |  | 204.476 |  |
|  |  | D1Ny |  | λ12 |  | 154.623 |  | 20.300 |  | 7.617 |  | < .001 |  | 114.836 |  | 194.411 |  |
|  |  | D1Nz |  | λ13 |  | 142.063 |  | 18.833 |  | 7.543 |  | < .001 |  | 105.150 |  | 178.975 |  |
| Factor 2 |  | C1Nx |  | λ21 |  | 183.293 |  | 18.052 |  | 10.153 |  | < .001 |  | 147.911 |  | 218.675 |  |
|  |  | C1Ny |  | λ22 |  | 180.394 |  | 17.920 |  | 10.067 |  | < .001 |  | 145.272 |  | 215.516 |  |
|  |  | C1Nz |  | λ23 |  | 167.366 |  | 16.776 |  | 9.977 |  | < .001 |  | 134.486 |  | 200.246 |  |
| Factor 3 |  | B1Nx |  | λ31 |  | 124.111 |  | 21.095 |  | 5.883 |  | < .001 |  | 82.765 |  | 165.457 |  |
|  |  | B1Ny |  | λ32 |  | 120.843 |  | 20.582 |  | 5.871 |  | < .001 |  | 80.503 |  | 161.184 |  |
|  |  | B1Nz |  | λ33 |  | 144.293 |  | 24.296 |  | 5.939 |  | < .001 |  | 96.675 |  | 191.912 |  |
| Factor 4 |  | A1Nx |  | λ41 |  | 231.412 |  | 19.143 |  | 12.089 |  | < .001 |  | 193.893 |  | 268.931 |  |
|  |  | A1Ny |  | λ42 |  | 238.834 |  | 19.095 |  | 12.508 |  | < .001 |  | 201.409 |  | 276.260 |  |
|  |  | A1Nz |  | λ43 |  | 237.554 |  | 18.826 |  | 12.618 |  | < .001 |  | 200.656 |  | 274.453 |  |
|  | | | | | | | | | | | | | | | | | |

| **Second-order factor loadings** | | | | | | | | | | | | | | | | | |
| --- | --- | --- | --- | --- | --- | --- | --- | --- | --- | --- | --- | --- | --- | --- | --- | --- | --- |
|  | | | | | | | | | | | | | | **95% Confidence Interval** | | | |
| **Factor** | | **Indicator** | | **Symbol** | | **Estimate** | | **Std. Error** | | **z-value** | | **p** | | **Lower** | | **Upper** | |
| SecondOrder |  | Factor 1 |  | γ11 |  | 1.328 |  | 0.198 |  | 6.714 |  | < .001 |  | 0.940 |  | 1.716 |  |
|  |  | Factor 2 |  | γ12 |  | 1.188 |  | 0.143 |  | 8.280 |  | < .001 |  | 0.906 |  | 1.469 |  |
|  |  | Factor 3 |  | γ13 |  | 1.633 |  | 0.307 |  | 5.318 |  | < .001 |  | 1.031 |  | 2.235 |  |
|  | | | | | | | | | | | | | | | | | |

| **Factor variances** | | | | | | | | | | | | | |
| --- | --- | --- | --- | --- | --- | --- | --- | --- | --- | --- | --- | --- | --- |
|  | | | | | | | | | | **95% Confidence Interval** | | | |
| **Factor** | | **Estimate** | | **Std. Error** | | **z-value** | | **p** | | **Lower** | | **Upper** | |
| Factor 1 |  | 1.000 |  | 0.000 |  |  |  |  |  | 1.000 |  | 1.000 |  |
| Factor 2 |  | 1.000 |  | 0.000 |  |  |  |  |  | 1.000 |  | 1.000 |  |
| Factor 3 |  | 1.000 |  | 0.000 |  |  |  |  |  | 1.000 |  | 1.000 |  |
| Factor 4 |  | 1.000 |  | 0.000 |  |  |  |  |  | 1.000 |  | 1.000 |  |
| Second-Order |  | 1.000 |  | 0.000 |  |  |  |  |  | 1.000 |  | 1.000 |  |
|  | | | | | | | | | | | | | |

| **Residual variances** | | | | | | | | | | | | | |
| --- | --- | --- | --- | --- | --- | --- | --- | --- | --- | --- | --- | --- | --- |
|  | | | | | | | | | | **95% Confidence Interval** | | | |
| **Indicator** | | **Estimate** | | **Std. Error** | | **z-value** | | **p** | | **Lower** | | **Upper** | |
| D1Nx |  | 185865.795 |  | 10963.813 |  | 16.953 |  | < .001 |  | 164377.116 |  | 207354.474 |  |
| D1Ny |  | 211330.407 |  | 11587.288 |  | 18.238 |  | < .001 |  | 188619.740 |  | 234041.075 |  |
| D1Nz |  | 196600.154 |  | 10526.516 |  | 18.677 |  | < .001 |  | 175968.562 |  | 217231.745 |  |
| C1Nx |  | 161579.805 |  | 9719.639 |  | 16.624 |  | < .001 |  | 142529.663 |  | 180629.947 |  |
| C1Ny |  | 173036.778 |  | 10046.041 |  | 17.224 |  | < .001 |  | 153346.899 |  | 192726.656 |  |
| C1Nz |  | 161418.792 |  | 9138.419 |  | 17.664 |  | < .001 |  | 143507.819 |  | 179329.765 |  |
| B1Nx |  | 210132.058 |  | 11024.311 |  | 19.061 |  | < .001 |  | 188524.806 |  | 231739.311 |  |
| B1Ny |  | 205660.362 |  | 10720.575 |  | 19.184 |  | < .001 |  | 184648.422 |  | 226672.303 |  |
| B1Nz |  | 182651.429 |  | 10898.756 |  | 16.759 |  | < .001 |  | 161290.260 |  | 204012.598 |  |
| A1Nx |  | 205987.437 |  | 10603.944 |  | 19.426 |  | < .001 |  | 185204.089 |  | 226770.785 |  |
| A1Ny |  | 199947.396 |  | 10496.982 |  | 19.048 |  | < .001 |  | 179373.689 |  | 220521.102 |  |
| A1Nz |  | 192961.409 |  | 10188.558 |  | 18.939 |  | < .001 |  | 172992.202 |  | 212930.615 |  |
|  | | | | | | | | | | | | | |

**Table 10.2.3 Experiential negative**

**Model fit**

| **Chi-square test** | | | | | | | |
| --- | --- | --- | --- | --- | --- | --- | --- |
| **Model** | | **Χ²** | | **df** | | **p** | |
| Baseline model |  | 1241.246 |  | 66 |  |  |  |
| Factor model |  | 97.702 |  | 50 |  | < .001 |  |
|  | | | | | | | |

**Additional fit measures**

| **Fit indices** | | | |
| --- | --- | --- | --- |
| **Index** | | **Value** | |
| Comparative Fit Index (CFI) |  | 0.959 |  |
| Tucker-Lewis Index (TLI) |  | 0.946 |  |
| Bentler-Bonett Non-normed Fit Index (NNFI) |  | 0.946 |  |
| Bentler-Bonett Normed Fit Index (NFI) |  | 0.921 |  |
| Parsimony Normed Fit Index (PNFI) |  | 0.698 |  |
| Bollen's Relative Fit Index (RFI) |  | 0.896 |  |
| Bollen's Incremental Fit Index (IFI) |  | 0.960 |  |
| Relative Noncentrality Index (RNI) |  | 0.959 |  |
|  | | | |

| **Information criteria** | | | |
| --- | --- | --- | --- |
|  | | **Value** | |
| Log-likelihood |  | -93592.513 |  |
| Number of free parameters |  | 28.000 |  |
| Akaike (AIC) |  | 187241.027 |  |
| Bayesian (BIC) |  | 187379.298 |  |
| Sample-size adjusted Bayesian (SSABIC) |  | 187290.367 |  |
|  | | | |

| **Other fit measures** | | | |
| --- | --- | --- | --- |
| **Metric** | | **Value** | |
| Root mean square error of approximation (RMSEA) |  | 0.030 |  |
| RMSEA 90% CI lower bound |  | 0.021 |  |
| RMSEA 90% CI upper bound |  | 0.039 |  |
| RMSEA p-value |  | 1.000 |  |
| Standardized root mean square residual (SRMR) |  | 0.036 |  |
| Hoelter's critical N (α = .05) |  | 713.347 |  |
| Hoelter's critical N (α = .01) |  | 804.617 |  |
| Goodness of fit index (GFI) |  | 0.984 |  |
| McDonald fit index (MFI) |  | 0.977 |  |
| Expected cross validation index (ECVI) |  | 0.149 |  |
|  | | | |

| **R-Squared** | | | |
| --- | --- | --- | --- |
|  | | **R²** | |
| D2Nx |  | 0.365 |  |
| D2Ny |  | 0.274 |  |
| D2Nz |  | 0.147 |  |
| C2Nx |  | 0.364 |  |
| C2Ny |  | 0.269 |  |
| C2Nz |  | 0.233 |  |
| B2Nx |  | 0.249 |  |
| B2Ny |  | 0.268 |  |
| B2Nz |  | 0.228 |  |
| A2Nx |  | 0.366 |  |
| A2Ny |  | 0.080 |  |
| A2Nz |  | 0.264 |  |
| Factor 1 |  | 0.788 |  |
| Factor 2 |  | 0.586 |  |
| Factor 3 |  | 0.535 |  |
|  | | | |

**Parameter estimates**

| **Factor loadings** | | | | | | | | | | | | | | | | | |
| --- | --- | --- | --- | --- | --- | --- | --- | --- | --- | --- | --- | --- | --- | --- | --- | --- | --- |
|  | | | | | | | | | | | | | | **95% Confidence Interval** | | | |
| **Factor** | | **Indicator** | | **Symbol** | | **Estimate** | | **Std. Error** | | **z-value** | | **p** | | **Lower** | | **Upper** | |
| Factor 1 |  | D2Nx |  | λ11 |  | 134.694 |  | 30.750 |  | 4.380 |  | < .001 |  | 74.424 |  | 194.963 |  |
|  |  | D2Ny |  | λ12 |  | 121.919 |  | 27.476 |  | 4.437 |  | < .001 |  | 68.067 |  | 175.771 |  |
|  |  | D2Nz |  | λ13 |  | 85.798 |  | 19.985 |  | 4.293 |  | < .001 |  | 46.628 |  | 124.969 |  |
| Factor 2 |  | C2Nx |  | λ21 |  | 193.221 |  | 21.796 |  | 8.865 |  | < .001 |  | 150.501 |  | 235.940 |  |
|  |  | C2Ny |  | λ22 |  | 169.919 |  | 19.526 |  | 8.702 |  | < .001 |  | 131.648 |  | 208.190 |  |
|  |  | C2Nz |  | λ23 |  | 149.705 |  | 17.647 |  | 8.483 |  | < .001 |  | 115.117 |  | 184.292 |  |
| Factor 3 |  | B2Nx |  | λ31 |  | 170.016 |  | 19.872 |  | 8.556 |  | < .001 |  | 131.068 |  | 208.964 |  |
|  |  | B2Ny |  | λ32 |  | 180.726 |  | 20.931 |  | 8.634 |  | < .001 |  | 139.702 |  | 221.751 |  |
|  |  | B2Nz |  | λ33 |  | 168.256 |  | 19.952 |  | 8.433 |  | < .001 |  | 129.152 |  | 207.360 |  |
| Factor 4 |  | A2Nx |  | λ41 |  | 273.962 |  | 22.384 |  | 12.239 |  | < .001 |  | 230.091 |  | 317.833 |  |
|  |  | A2Ny |  | λ42 |  | 132.088 |  | 19.524 |  | 6.765 |  | < .001 |  | 93.821 |  | 170.356 |  |
|  |  | A2Nz |  | λ43 |  | 237.209 |  | 21.113 |  | 11.235 |  | < .001 |  | 195.828 |  | 278.591 |  |
|  | | | | | | | | | | | | | | | | | |

| **Second-order factor loadings** | | | | | | | | | | | | | | | | | |
| --- | --- | --- | --- | --- | --- | --- | --- | --- | --- | --- | --- | --- | --- | --- | --- | --- | --- |
|  | | | | | | | | | | | | | | **95% Confidence Interval** | | | |
| **Factor** | | **Indicator** | | **Symbol** | | **Estimate** | | **Std. Error** | | **z-value** | | **p** | | **Lower** | | **Upper** | |
| SecondOrder |  | Factor 1 |  | γ11 |  | 1.926 |  | 0.503 |  | 3.830 |  | < .001 |  | 0.941 |  | 2.912 |  |
|  |  | Factor 2 |  | γ12 |  | 1.189 |  | 0.172 |  | 6.913 |  | < .001 |  | 0.852 |  | 1.526 |  |
|  |  | Factor 3 |  | γ13 |  | 1.072 |  | 0.153 |  | 7.000 |  | < .001 |  | 0.772 |  | 1.372 |  |
|  | | | | | | | | | | | | | | | | | |

| **Factor variances** | | | | | | | | | | | | | |
| --- | --- | --- | --- | --- | --- | --- | --- | --- | --- | --- | --- | --- | --- |
|  | | | | | | | | | | **95% Confidence Interval** | | | |
| **Factor** | | **Estimate** | | **Std. Error** | | **z-value** | | **p** | | **Lower** | | **Upper** | |
| Factor 1 |  | 1.000 |  | 0.000 |  |  |  |  |  | 1.000 |  | 1.000 |  |
| Factor 2 |  | 1.000 |  | 0.000 |  |  |  |  |  | 1.000 |  | 1.000 |  |
| Factor 3 |  | 1.000 |  | 0.000 |  |  |  |  |  | 1.000 |  | 1.000 |  |
| Factor 4 |  | 1.000 |  | 0.000 |  |  |  |  |  | 1.000 |  | 1.000 |  |
| Second-Order |  | 1.000 |  | 0.000 |  |  |  |  |  | 1.000 |  | 1.000 |  |
|  | | | | | | | | | | | | | |

| **Residual variances** | | | | | | | | | | | | | |
| --- | --- | --- | --- | --- | --- | --- | --- | --- | --- | --- | --- | --- | --- |
|  | | | | | | | | | | **95% Confidence Interval** | | | |
| **Indicator** | | **Estimate** | | **Std. Error** | | **z-value** | | **p** | | **Lower** | | **Upper** | |
| D2Nx |  | 148923.451 |  | 10110.607 |  | 14.729 |  | < .001 |  | 129107.026 |  | 168739.877 |  |
| D2Ny |  | 185192.962 |  | 10522.722 |  | 17.599 |  | < .001 |  | 164568.806 |  | 205817.118 |  |
| D2Nz |  | 201344.057 |  | 9841.679 |  | 20.458 |  | < .001 |  | 182054.721 |  | 220633.393 |  |
| C2Nx |  | 157616.519 |  | 10688.503 |  | 14.746 |  | < .001 |  | 136667.437 |  | 178565.600 |  |
| C2Ny |  | 189329.550 |  | 10738.580 |  | 17.631 |  | < .001 |  | 168282.320 |  | 210376.780 |  |
| C2Nz |  | 178161.271 |  | 9601.691 |  | 18.555 |  | < .001 |  | 159342.302 |  | 196980.241 |  |
| B2Nx |  | 187567.097 |  | 10879.587 |  | 17.240 |  | < .001 |  | 166243.498 |  | 208890.697 |  |
| B2Ny |  | 191259.976 |  | 11503.847 |  | 16.626 |  | < .001 |  | 168712.850 |  | 213807.102 |  |
| B2Nz |  | 206110.074 |  | 11542.112 |  | 17.857 |  | < .001 |  | 183487.949 |  | 228732.198 |  |
| A2Nx |  | 129797.517 |  | 11592.055 |  | 11.197 |  | < .001 |  | 107077.506 |  | 152517.528 |  |
| A2Ny |  | 199471.749 |  | 9470.813 |  | 21.062 |  | < .001 |  | 180909.295 |  | 218034.202 |  |
| A2Nz |  | 157073.536 |  | 10380.740 |  | 15.131 |  | < .001 |  | 136727.658 |  | 177419.413 |  |
|  | | | | | | | | | | | | | |

**Table 10.2.4 Aspirational negative**

**Model fit**

| **Chi-square test** | | | | | | | |
| --- | --- | --- | --- | --- | --- | --- | --- |
| **Model** | | **Χ²** | | **df** | | **p** | |
| Baseline model |  | 1464.756 |  | 66 |  |  |  |
| Factor model |  | 100.733 |  | 50 |  | < .001 |  |
|  | | | | | | | |

**Additional fit measures**

| **Fit indices** | | | |
| --- | --- | --- | --- |
| **Index** | | **Value** | |
| Comparative Fit Index (CFI) |  | 0.964 |  |
| Tucker-Lewis Index (TLI) |  | 0.952 |  |
| Bentler-Bonett Non-normed Fit Index (NNFI) |  | 0.952 |  |
| Bentler-Bonett Normed Fit Index (NFI) |  | 0.931 |  |
| Parsimony Normed Fit Index (PNFI) |  | 0.705 |  |
| Bollen's Relative Fit Index (RFI) |  | 0.909 |  |
| Bollen's Incremental Fit Index (IFI) |  | 0.964 |  |
| Relative Noncentrality Index (RNI) |  | 0.964 |  |
|  | | | |

| **Information criteria** | | | |
| --- | --- | --- | --- |
|  | | **Value** | |
| Log-likelihood |  | -93711.766 |  |
| Number of free parameters |  | 28.000 |  |
| Akaike (AIC) |  | 187479.531 |  |
| Bayesian (BIC) |  | 187617.803 |  |
| Sample-size adjusted Bayesian (SSABIC) |  | 187528.872 |  |
|  | | | |

| **Other fit measures** | | | |
| --- | --- | --- | --- |
| **Metric** | | **Value** | |
| Root mean square error of approximation (RMSEA) |  | 0.031 |  |
| RMSEA 90% CI lower bound |  | 0.022 |  |
| RMSEA 90% CI upper bound |  | 0.040 |  |
| RMSEA p-value |  | 1.000 |  |
| Standardized root mean square residual (SRMR) |  | 0.032 |  |
| Hoelter's critical N (α = .05) |  | 691.909 |  |
| Hoelter's critical N (α = .01) |  | 780.432 |  |
| Goodness of fit index (GFI) |  | 0.984 |  |
| McDonald fit index (MFI) |  | 0.976 |  |
| Expected cross validation index (ECVI) |  | 0.152 |  |
|  | | | |

| **R-Squared** | | | |
| --- | --- | --- | --- |
|  | | **R²** | |
| D3Nx |  | 0.343 |  |
| D3Ny |  | 0.215 |  |
| D3Nz |  | 0.268 |  |
| C3Nx |  | 0.332 |  |
| C3Ny |  | 0.407 |  |
| C3Nz |  | 0.412 |  |
| B3Nx |  | 0.276 |  |
| B3Ny |  | 0.147 |  |
| B3Nz |  | 0.182 |  |
| A3Nx |  | 0.261 |  |
| A3Ny |  | 0.138 |  |
| A3Nz |  | 0.280 |  |
| Factor 1 |  | 0.395 |  |
| Factor 2 |  | 0.416 |  |
| Factor 3 |  | 0.964 |  |
|  | | | |

**Parameter estimates**

| **Factor loadings** | | | | | | | | | | | | | | | | | |
| --- | --- | --- | --- | --- | --- | --- | --- | --- | --- | --- | --- | --- | --- | --- | --- | --- | --- |
|  | | | | | | | | | | | | | | **95% Confidence Interval** | | | |
| **Factor** | | **Indicator** | | **Symbol** | | **Estimate** | | **Std. Error** | | **z-value** | | **p** | | **Lower** | | **Upper** | |
| Factor 1 |  | D3Nx |  | λ11 |  | 239.346 |  | 21.254 |  | 11.261 |  | < .001 |  | 197.689 |  | 281.003 |  |
|  |  | D3Ny |  | λ12 |  | 193.916 |  | 19.159 |  | 10.121 |  | < .001 |  | 156.364 |  | 231.467 |  |
|  |  | D3Nz |  | λ13 |  | 204.547 |  | 18.899 |  | 10.823 |  | < .001 |  | 167.506 |  | 241.588 |  |
| Factor 2 |  | C3Nx |  | λ21 |  | 212.688 |  | 15.818 |  | 13.446 |  | < .001 |  | 181.684 |  | 243.691 |  |
|  |  | C3Ny |  | λ22 |  | 233.531 |  | 16.428 |  | 14.215 |  | < .001 |  | 201.332 |  | 265.729 |  |
|  |  | C3Nz |  | λ23 |  | 230.453 |  | 16.172 |  | 14.250 |  | < .001 |  | 198.757 |  | 262.150 |  |
| Factor 3 |  | B3Nx |  | λ31 |  | 50.673 |  | 75.681 |  | 0.670 |  | 0.503 |  | -97.660 |  | 199.005 |  |
|  |  | B3Ny |  | λ32 |  | 34.450 |  | 51.251 |  | 0.672 |  | 0.501 |  | -66.001 |  | 134.900 |  |
|  |  | B3Nz |  | λ33 |  | 37.213 |  | 55.389 |  | 0.672 |  | 0.502 |  | -71.349 |  | 145.774 |  |
| Factor 4 |  | A3Nx |  | λ41 |  | 272.234 |  | 20.875 |  | 13.041 |  | < .001 |  | 231.320 |  | 313.147 |  |
|  |  | A3Ny |  | λ42 |  | 187.532 |  | 19.509 |  | 9.613 |  | < .001 |  | 149.296 |  | 225.768 |  |
|  |  | A3Nz |  | λ43 |  | 265.936 |  | 19.794 |  | 13.435 |  | < .001 |  | 227.140 |  | 304.732 |  |
|  | | | | | | | | | | | | | | | | | |

| **Second-order factor loadings** | | | | | | | | | | | | | | | | | |
| --- | --- | --- | --- | --- | --- | --- | --- | --- | --- | --- | --- | --- | --- | --- | --- | --- | --- |
|  | | | | | | | | | | | | | | **95% Confidence Interval** | | | |
| **Factor** | | **Indicator** | | **Symbol** | | **Estimate** | | **Std. Error** | | **z-value** | | **p** | | **Lower** | | **Upper** | |
| SecondOrder |  | Factor 1 |  | γ11 |  | 0.808 |  | 0.096 |  | 8.406 |  | < .001 |  | 0.619 |  | 0.996 |  |
|  |  | Factor 2 |  | γ12 |  | 0.843 |  | 0.088 |  | 9.589 |  | < .001 |  | 0.671 |  | 1.016 |  |
|  |  | Factor 3 |  | γ13 |  | 5.174 |  | 7.862 |  | 0.658 |  | 0.510 |  | -10.234 |  | 20.582 |  |
|  | | | | | | | | | | | | | | | | | |

| **Factor variances** | | | | | | | | | | | | | |
| --- | --- | --- | --- | --- | --- | --- | --- | --- | --- | --- | --- | --- | --- |
|  | | | | | | | | | | **95% Confidence Interval** | | | |
| **Factor** | | **Estimate** | | **Std. Error** | | **z-value** | | **p** | | **Lower** | | **Upper** | |
| Factor 1 |  | 1.000 |  | 0.000 |  |  |  |  |  | 1.000 |  | 1.000 |  |
| Factor 2 |  | 1.000 |  | 0.000 |  |  |  |  |  | 1.000 |  | 1.000 |  |
| Factor 3 |  | 1.000 |  | 0.000 |  |  |  |  |  | 1.000 |  | 1.000 |  |
| Factor 4 |  | 1.000 |  | 0.000 |  |  |  |  |  | 1.000 |  | 1.000 |  |
| Second-Order |  | 1.000 |  | 0.000 |  |  |  |  |  | 1.000 |  | 1.000 |  |
|  | | | | | | | | | | | | | |

| **Residual variances** | | | | | | | | | | | | | |
| --- | --- | --- | --- | --- | --- | --- | --- | --- | --- | --- | --- | --- | --- |
|  | | | | | | | | | | **95% Confidence Interval** | | | |
| **Indicator** | | **Estimate** | | **Std. Error** | | **z-value** | | **p** | | **Lower** | | **Upper** | |
| D3Nx |  | 181438.317 |  | 12675.640 |  | 14.314 |  | < .001 |  | 156594.520 |  | 206282.115 |  |
| D3Ny |  | 226961.828 |  | 12363.705 |  | 18.357 |  | < .001 |  | 202729.412 |  | 251194.245 |  |
| D3Nz |  | 188433.717 |  | 11221.230 |  | 16.793 |  | < .001 |  | 166440.511 |  | 210426.923 |  |
| C3Nx |  | 156089.268 |  | 8981.302 |  | 17.379 |  | < .001 |  | 138486.239 |  | 173692.297 |  |
| C3Ny |  | 136080.494 |  | 8921.032 |  | 15.254 |  | < .001 |  | 118595.592 |  | 153565.396 |  |
| C3Nz |  | 129783.813 |  | 8594.962 |  | 15.100 |  | < .001 |  | 112937.997 |  | 146629.628 |  |
| B3Nx |  | 186902.117 |  | 11068.548 |  | 16.886 |  | < .001 |  | 165208.161 |  | 208596.074 |  |
| B3Ny |  | 191699.497 |  | 9384.974 |  | 20.426 |  | < .001 |  | 173305.286 |  | 210093.708 |  |
| B3Nz |  | 172741.938 |  | 8788.179 |  | 19.656 |  | < .001 |  | 155517.422 |  | 189966.453 |  |
| A3Nx |  | 210193.843 |  | 11991.252 |  | 17.529 |  | < .001 |  | 186691.421 |  | 233696.264 |  |
| A3Ny |  | 218954.043 |  | 10689.372 |  | 20.483 |  | < .001 |  | 198003.260 |  | 239904.827 |  |
| A3Nz |  | 181539.276 |  | 10735.773 |  | 16.910 |  | < .001 |  | 160497.548 |  | 202581.004 |  |
|  | | | | | | | | | | | | | |
