# Supplementary material for: The structure of human motivation
Source: BMC Psychol. 2023 Oct 6;11:308. doi: 10.1186/s40359-023-01346-5 (PMC10557177; doi:10.1186/s40359-023-01346-5)
Supplement: Supplementary file 5 — Additional file 5: SM Table 10. Confirmatory factor models for the three levels of attainment: Full output. [file 40359_2023_1346_MOESM5_ESM.zip › Tables 10.1.CFA.levels of attainment.positiveR5.docx]

**Table 10.1 Level of attainment (positive)**

**Table 10.1.1 CFA Overall promotion motivation model (levels of attainment)**

### Model fit

| **Chi-square test** | | | | | | | |
| --- | --- | --- | --- | --- | --- | --- | --- |
| **Model** | | **Χ²** | | **df** | | **p** | |
| Baseline model |  | 13954.656 |  | 595 |  |  |  |
| Factor model |  | 1818.986 |  | 557 |  | < .001 |  |
|  | | | | | | | |

#### Additional fit measures

| **Fit indices** | | | |
| --- | --- | --- | --- |
| **Index** | | **Value** | |
| Comparative Fit Index (CFI) |  | 0.906 |  |
| Tucker-Lewis Index (TLI) |  | 0.899 |  |
| Bentler-Bonett Non-normed Fit Index (NNFI) |  | 0.899 |  |
| Bentler-Bonett Normed Fit Index (NFI) |  | 0.870 |  |
| Parsimony Normed Fit Index (PNFI) |  | 0.814 |  |
| Bollen's Relative Fit Index (RFI) |  | 0.861 |  |
| Bollen's Incremental Fit Index (IFI) |  | 0.906 |  |
| Relative Noncentrality Index (RNI) |  | 0.906 |  |
|  | | | |

| **Information criteria** | | | |
| --- | --- | --- | --- |
|  | | **Value** | |
| Log-likelihood |  | -259155.831 |  |
| Number of free parameters |  | 73.000 |  |
| Akaike (AIC) |  | 518457.663 |  |
| Bayesian (BIC) |  | 518818.157 |  |
| Sample-size adjusted Bayesian (SSABIC) |  | 518586.301 |  |
|  | | | |

| **Other fit measures** | | | |
| --- | --- | --- | --- |
| **Metric** | | **Value** | |
| Root mean square error of approximation (RMSEA) |  | 0.047 |  |
| RMSEA 90% CI lower bound |  | 0.044 |  |
| RMSEA 90% CI upper bound |  | 0.049 |  |
| RMSEA p-value |  | 0.984 |  |
| Standardized root mean square residual (SRMR) |  | 0.040 |  |
| Hoelter's critical N (α = .05) |  | 348.455 |  |
| Hoelter's critical N (α = .01) |  | 362.376 |  |
| Goodness of fit index (GFI) |  | 0.896 |  |
| McDonald fit index (MFI) |  | 0.542 |  |
| Expected cross validation index (ECVI) |  | 1.906 |  |
|  | | | |

| **R-Squared** | | | |
| --- | --- | --- | --- |
|  | | **R²** | |
| A1Px |  | 0.285 |  |
| A1Py |  | 0.384 |  |
| A1Pz |  | 0.260 |  |
| B1Px |  | 0.390 |  |
| B1Py |  | 0.395 |  |
| B1Pz |  | 0.347 |  |
| C1Py |  | 0.358 |  |
| C1Pz |  | 0.405 |  |
| D1Px |  | 0.370 |  |
| D1Py |  | 0.403 |  |
| D1Pz |  | 0.298 |  |
| A2Px |  | 0.385 |  |
| A2Py |  | 0.396 |  |
| A2Pz |  | 0.338 |  |
| B2Px |  | 0.381 |  |
| B2Py |  | 0.407 |  |
| B2Pz |  | 0.411 |  |
| C2Px |  | 0.366 |  |
| C2Py |  | 0.384 |  |
| C2Pz |  | 0.413 |  |
| D2Px |  | 0.354 |  |
| D2Py |  | 0.416 |  |
| D2Pz |  | 0.398 |  |
| A3Px |  | 0.292 |  |
| A3Py |  | 0.274 |  |
| A3Pz |  | 0.251 |  |
| B3Px |  | 0.324 |  |
| B3Py |  | 0.305 |  |
| B3Pz |  | 0.333 |  |
| C3Px |  | 0.316 |  |
| C3Py |  | 0.385 |  |
| C3Pz |  | 0.363 |  |
| D3Px |  | 0.365 |  |
| D3Py |  | 0.307 |  |
| D3Pz |  | 0.360 |  |
|  | | | |

### Parameter estimates

| **Factor loadings** | | | | | | | | | | | | | | | | | |
| --- | --- | --- | --- | --- | --- | --- | --- | --- | --- | --- | --- | --- | --- | --- | --- | --- | --- |
|  | | | | | | | | | | | | | | **95% Confidence Interval** | | | |
| **Factor** | | **Indicator** | | **Symbol** | | **Estimate** | | **Std. Error** | | **z-value** | | **p** | | **Lower** | | **Upper** | |
| Foundational |  | A1Px |  | λ11 |  | 206.608 |  | 11.496 |  | 17.973 |  | < .001 |  | 184.077 |  | 229.140 |  |
|  |  | A1Py |  | λ12 |  | 226.005 |  | 10.509 |  | 21.505 |  | < .001 |  | 205.407 |  | 246.603 |  |
|  |  | A1Pz |  | λ13 |  | 188.366 |  | 11.066 |  | 17.022 |  | < .001 |  | 166.677 |  | 210.055 |  |
|  |  | B1Px |  | λ14 |  | 222.808 |  | 10.262 |  | 21.713 |  | < .001 |  | 202.696 |  | 242.921 |  |
|  |  | B1Py |  | λ15 |  | 222.729 |  | 10.177 |  | 21.885 |  | < .001 |  | 202.782 |  | 242.677 |  |
|  |  | B1Pz |  | λ16 |  | 219.298 |  | 10.853 |  | 20.205 |  | < .001 |  | 198.025 |  | 240.570 |  |
|  |  | C1Py |  | λ17 |  | 232.270 |  | 11.284 |  | 20.584 |  | < .001 |  | 210.153 |  | 254.386 |  |
|  |  | C1Pz |  | λ18 |  | 239.895 |  | 10.786 |  | 22.240 |  | < .001 |  | 218.754 |  | 261.036 |  |
|  |  | D1Px |  | λ19 |  | 221.481 |  | 10.538 |  | 21.018 |  | < .001 |  | 200.827 |  | 242.134 |  |
|  |  | D1Py |  | λ110 |  | 250.044 |  | 11.282 |  | 22.162 |  | < .001 |  | 227.931 |  | 272.157 |  |
|  |  | D1Pz |  | λ111 |  | 197.845 |  | 10.738 |  | 18.424 |  | < .001 |  | 176.799 |  | 218.892 |  |
| Experiential |  | A2Px |  | λ21 |  | 222.591 |  | 10.362 |  | 21.482 |  | < .001 |  | 202.282 |  | 242.900 |  |
|  |  | A2Py |  | λ22 |  | 212.821 |  | 9.739 |  | 21.853 |  | < .001 |  | 193.734 |  | 231.908 |  |
|  |  | A2Pz |  | λ23 |  | 217.924 |  | 10.986 |  | 19.836 |  | < .001 |  | 196.391 |  | 239.457 |  |
|  |  | B2Px |  | λ24 |  | 232.293 |  | 10.874 |  | 21.363 |  | < .001 |  | 210.981 |  | 253.604 |  |
|  |  | B2Py |  | λ25 |  | 238.770 |  | 10.728 |  | 22.258 |  | < .001 |  | 217.744 |  | 259.796 |  |
|  |  | B2Pz |  | λ26 |  | 244.778 |  | 10.925 |  | 22.405 |  | < .001 |  | 223.365 |  | 266.190 |  |
|  |  | C2Px |  | λ27 |  | 230.652 |  | 11.082 |  | 20.814 |  | < .001 |  | 208.932 |  | 252.372 |  |
|  |  | C2Py |  | λ28 |  | 232.687 |  | 10.839 |  | 21.467 |  | < .001 |  | 211.443 |  | 253.932 |  |
|  |  | C2Pz |  | λ29 |  | 255.409 |  | 11.367 |  | 22.469 |  | < .001 |  | 233.129 |  | 277.689 |  |
|  |  | D2Px |  | λ210 |  | 215.798 |  | 10.584 |  | 20.388 |  | < .001 |  | 195.053 |  | 236.543 |  |
|  |  | D2Py |  | λ211 |  | 229.334 |  | 10.170 |  | 22.549 |  | < .001 |  | 209.401 |  | 249.268 |  |
|  |  | D2Pz |  | λ212 |  | 229.976 |  | 10.474 |  | 21.956 |  | < .001 |  | 209.447 |  | 250.506 |  |
| Aspirational |  | A3Px |  | λ31 |  | 211.325 |  | 11.775 |  | 17.946 |  | < .001 |  | 188.246 |  | 234.404 |  |
|  |  | A3Py |  | λ32 |  | 207.430 |  | 11.983 |  | 17.310 |  | < .001 |  | 183.943 |  | 230.918 |  |
|  |  | A3Pz |  | λ33 |  | 198.355 |  | 12.076 |  | 16.425 |  | < .001 |  | 174.686 |  | 222.024 |  |
|  |  | B3Px |  | λ34 |  | 217.014 |  | 11.358 |  | 19.106 |  | < .001 |  | 194.752 |  | 239.276 |  |
|  |  | B3Py |  | λ35 |  | 210.893 |  | 11.452 |  | 18.416 |  | < .001 |  | 188.448 |  | 233.337 |  |
|  |  | B3Pz |  | λ36 |  | 221.036 |  | 11.375 |  | 19.432 |  | < .001 |  | 198.742 |  | 243.330 |  |
|  |  | C3Px |  | λ37 |  | 223.851 |  | 11.905 |  | 18.802 |  | < .001 |  | 200.517 |  | 247.185 |  |
|  |  | C3Py |  | λ38 |  | 237.534 |  | 11.189 |  | 21.230 |  | < .001 |  | 215.604 |  | 259.464 |  |
|  |  | C3Pz |  | λ39 |  | 233.248 |  | 11.382 |  | 20.492 |  | < .001 |  | 210.939 |  | 255.557 |  |
|  |  | D3Px |  | λ310 |  | 231.551 |  | 11.272 |  | 20.542 |  | < .001 |  | 209.458 |  | 253.644 |  |
|  |  | D3Py |  | λ311 |  | 211.514 |  | 11.437 |  | 18.494 |  | < .001 |  | 189.097 |  | 233.930 |  |
|  |  | D3Pz |  | λ312 |  | 236.081 |  | 11.597 |  | 20.357 |  | < .001 |  | 213.352 |  | 258.811 |  |
|  | | | | | | | | | | | | | | | | | |

| **Factor variances** | | | | | | | | | | | | | |
| --- | --- | --- | --- | --- | --- | --- | --- | --- | --- | --- | --- | --- | --- |
|  | | | | | | | | | | **95% Confidence Interval** | | | |
| **Factor** | | **Estimate** | | **Std. Error** | | **z-value** | | **p** | | **Lower** | | **Upper** | |
| Foundational |  | 1.000 |  | 0.000 |  |  |  |  |  | 1.000 |  | 1.000 |  |
| Experiential |  | 1.000 |  | 0.000 |  |  |  |  |  | 1.000 |  | 1.000 |  |
| Aspirational |  | 1.000 |  | 0.000 |  |  |  |  |  | 1.000 |  | 1.000 |  |
|  | | | | | | | | | | | | | |

| **Factor Covariances** | | | | | | | | | | | | | | | | | |
| --- | --- | --- | --- | --- | --- | --- | --- | --- | --- | --- | --- | --- | --- | --- | --- | --- | --- |
|  | | | | | | | | | | | | | | **95% Confidence Interval** | | | |
|  | |  | |  | | **Estimate** | | **Std. Error** | | **z-value** | | **p** | | **Lower** | | **Upper** | |
| Foundational |  | ↔ |  | Experiential |  | 0.970 |  | 0.008 |  | 122.017 |  | < .001 |  | 0.954 |  | 0.986 |  |
| Foundational |  | ↔ |  | Aspirational |  | 0.935 |  | 0.011 |  | 88.959 |  | < .001 |  | 0.914 |  | 0.955 |  |
| Experiential |  | ↔ |  | Aspirational |  | 0.893 |  | 0.012 |  | 75.189 |  | < .001 |  | 0.870 |  | 0.917 |  |
|  | | | | | | | | | | | | | | | | | |

| **Residual variances** | | | | | | | | | | | | | |
| --- | --- | --- | --- | --- | --- | --- | --- | --- | --- | --- | --- | --- | --- |
|  | | | | | | | | | | **95% Confidence Interval** | | | |
| **Indicator** | | **Estimate** | | **Std. Error** | | **z-value** | | **p** | | **Lower** | | **Upper** | |
| A1Px |  | 106959.363 |  | 4848.555 |  | 22.060 |  | < .001 |  | 97456.369 |  | 116462.356 |  |
| A1Py |  | 81984.622 |  | 3781.023 |  | 21.683 |  | < .001 |  | 74573.952 |  | 89395.291 |  |
| A1Pz |  | 101071.314 |  | 4565.215 |  | 22.139 |  | < .001 |  | 92123.656 |  | 110018.972 |  |
| B1Px |  | 77711.104 |  | 3588.407 |  | 21.656 |  | < .001 |  | 70677.955 |  | 84744.252 |  |
| B1Py |  | 76068.081 |  | 3516.248 |  | 21.633 |  | < .001 |  | 69176.361 |  | 82959.801 |  |
| B1Pz |  | 90516.466 |  | 4144.732 |  | 21.839 |  | < .001 |  | 82392.941 |  | 98639.991 |  |
| C1Py |  | 96897.321 |  | 4445.672 |  | 21.796 |  | < .001 |  | 88183.963 |  | 105610.679 |  |
| C1Pz |  | 84564.185 |  | 3917.816 |  | 21.585 |  | < .001 |  | 76885.406 |  | 92242.964 |  |
| D1Px |  | 83536.472 |  | 3841.780 |  | 21.744 |  | < .001 |  | 76006.722 |  | 91066.222 |  |
| D1Py |  | 92733.433 |  | 4294.125 |  | 21.595 |  | < .001 |  | 84317.102 |  | 101149.763 |  |
| D1Pz |  | 92417.587 |  | 4197.088 |  | 22.019 |  | < .001 |  | 84191.445 |  | 100643.728 |  |
| A2Px |  | 79191.313 |  | 3668.765 |  | 21.585 |  | < .001 |  | 72000.666 |  | 86381.961 |  |
| A2Py |  | 69221.720 |  | 3214.632 |  | 21.533 |  | < .001 |  | 62921.157 |  | 75522.283 |  |
| A2Pz |  | 92927.588 |  | 4264.215 |  | 21.792 |  | < .001 |  | 84569.881 |  | 101285.296 |  |
| B2Px |  | 87490.408 |  | 4050.217 |  | 21.601 |  | < .001 |  | 79552.128 |  | 95428.688 |  |
| B2Py |  | 83019.026 |  | 3865.936 |  | 21.474 |  | < .001 |  | 75441.931 |  | 90596.121 |  |
| B2Pz |  | 85729.602 |  | 3996.274 |  | 21.452 |  | < .001 |  | 77897.048 |  | 93562.156 |  |
| C2Px |  | 92226.212 |  | 4255.228 |  | 21.674 |  | < .001 |  | 83886.118 |  | 100566.306 |  |
| C2Py |  | 86690.657 |  | 4015.823 |  | 21.587 |  | < .001 |  | 78819.788 |  | 94561.527 |  |
| C2Pz |  | 92637.734 |  | 4320.231 |  | 21.443 |  | < .001 |  | 84170.237 |  | 101105.232 |  |
| D2Px |  | 85067.229 |  | 3915.319 |  | 21.727 |  | < .001 |  | 77393.345 |  | 92741.113 |  |
| D2Py |  | 73975.508 |  | 3451.890 |  | 21.430 |  | < .001 |  | 67209.927 |  | 80741.088 |  |
| D2Pz |  | 79843.470 |  | 3710.433 |  | 21.519 |  | < .001 |  | 72571.155 |  | 87115.785 |  |
| A3Px |  | 108399.270 |  | 4991.283 |  | 21.718 |  | < .001 |  | 98616.535 |  | 118182.005 |  |
| A3Py |  | 113787.982 |  | 5219.740 |  | 21.800 |  | < .001 |  | 103557.480 |  | 124018.484 |  |
| A3Pz |  | 117613.863 |  | 5369.320 |  | 21.905 |  | < .001 |  | 107090.189 |  | 128137.538 |  |
| B3Px |  | 98238.782 |  | 4557.738 |  | 21.554 |  | < .001 |  | 89305.779 |  | 107171.785 |  |
| B3Py |  | 101461.890 |  | 4685.626 |  | 21.654 |  | < .001 |  | 92278.231 |  | 110645.548 |  |
| B3Pz |  | 97751.644 |  | 4545.598 |  | 21.505 |  | < .001 |  | 88842.436 |  | 106660.852 |  |
| C3Px |  | 108699.414 |  | 5032.622 |  | 21.599 |  | < .001 |  | 98835.656 |  | 118563.172 |  |
| C3Py |  | 90241.456 |  | 4256.927 |  | 21.199 |  | < .001 |  | 81898.033 |  | 98584.880 |  |
| C3Pz |  | 95281.963 |  | 4466.765 |  | 21.331 |  | < .001 |  | 86527.265 |  | 104036.662 |  |
| D3Px |  | 93320.436 |  | 4376.587 |  | 21.323 |  | < .001 |  | 84742.483 |  | 101898.389 |  |
| D3Py |  | 101026.331 |  | 4667.855 |  | 21.643 |  | < .001 |  | 91877.503 |  | 110175.160 |  |
| D3Pz |  | 99262.045 |  | 4648.299 |  | 21.354 |  | < .001 |  | 90151.546 |  | 108372.544 |  |
|  | | | | | | | | | | | | | |

**Table 10.1.2 Foundational positive**

**Model fit**

| **Chi-square test** | | | | | | | |
| --- | --- | --- | --- | --- | --- | --- | --- |
| **Model** | | **Χ²** | | **df** | | **p** | |
| Baseline model |  | 730.472 |  | 66 |  |  |  |
| Factor model |  | 87.709 |  | 50 |  | < .001 |  |
|  | | | | | | | |

**Additional fit measures**

| **Fit indices** | | | |
| --- | --- | --- | --- |
| **Index** | | **Value** | |
| Comparative Fit Index (CFI) |  | 0.943 |  |
| Tucker-Lewis Index (TLI) |  | 0.925 |  |
| Bentler-Bonett Non-normed Fit Index (NNFI) |  | 0.925 |  |
| Bentler-Bonett Normed Fit Index (NFI) |  | 0.880 |  |
| Parsimony Normed Fit Index (PNFI) |  | 0.667 |  |
| Bollen's Relative Fit Index (RFI) |  | 0.842 |  |
| Bollen's Incremental Fit Index (IFI) |  | 0.945 |  |
| Relative Noncentrality Index (RNI) |  | 0.943 |  |
|  | | | |

| **Information criteria** | | | |
| --- | --- | --- | --- |
|  | | **Value** | |
| Log-likelihood |  | -94357.654 |  |
| Number of free parameters |  | 28.000 |  |
| Akaike (AIC) |  | 188771.308 |  |
| Bayesian (BIC) |  | 188909.580 |  |
| Sample-size adjusted Bayesian (SSABIC) |  | 188820.649 |  |
|  | | | |

| **Other fit measures** | | | |
| --- | --- | --- | --- |
| **Metric** | | **Value** | |
| Root mean square error of approximation (RMSEA) |  | 0.027 |  |
| RMSEA 90% CI lower bound |  | 0.017 |  |
| RMSEA 90% CI upper bound |  | 0.036 |  |
| RMSEA p-value |  | 1.000 |  |
| Standardized root mean square residual (SRMR) |  | 0.030 |  |
| Hoelter's critical N (α = .05) |  | 794.505 |  |
| Hoelter's critical N (α = .01) |  | 896.173 |  |
| Goodness of fit index (GFI) |  | 0.986 |  |
| McDonald fit index (MFI) |  | 0.982 |  |
| Expected cross validation index (ECVI) |  | 0.139 |  |
|  | | | |

| **R-Squared** | | | |
| --- | --- | --- | --- |
|  | | **R²** | |
| D1Px |  | 0.276 |  |
| D1Py |  | 0.132 |  |
| D1Pz |  | 0.234 |  |
| C1Px |  | 0.209 |  |
| C1Py |  | 0.159 |  |
| C1Pz |  | 0.237 |  |
| B1Px |  | 0.136 |  |
| B1Py |  | 0.170 |  |
| B1Pz |  | 0.099 |  |
| A1Px |  | 0.197 |  |
| A1Py |  | 0.106 |  |
| A1Pz |  | 0.119 |  |
| Factor 1 |  | 0.641 |  |
| Factor 2 |  | 0.714 |  |
| Factor 3 |  | 0.705 |  |
|  | | | |

**Parameter estimates**

| **Factor loadings** | | | | | | | | | | | | | | | | | |
| --- | --- | --- | --- | --- | --- | --- | --- | --- | --- | --- | --- | --- | --- | --- | --- | --- | --- |
|  | | | | | | | | | | | | | | **95% Confidence Interval** | | | |
| **Factor** | | **Indicator** | | **Symbol** | | **Estimate** | | **Std. Error** | | **z-value** | | **p** | | **Lower** | | **Upper** | |
| Factor 1 |  | D1Px |  | λ11 |  | 169.232 |  | 28.693 |  | 5.898 |  | < .001 |  | 112.996 |  | 225.469 |  |
|  |  | D1Py |  | λ12 |  | 108.251 |  | 19.563 |  | 5.534 |  | < .001 |  | 69.909 |  | 146.593 |  |
|  |  | D1Pz |  | λ13 |  | 148.602 |  | 25.097 |  | 5.921 |  | < .001 |  | 99.412 |  | 197.791 |  |
| Factor 2 |  | C1Px |  | λ21 |  | 122.786 |  | 26.922 |  | 4.561 |  | < .001 |  | 70.020 |  | 175.551 |  |
|  |  | C1Py |  | λ22 |  | 108.447 |  | 24.106 |  | 4.499 |  | < .001 |  | 61.200 |  | 155.694 |  |
|  |  | C1Pz |  | λ23 |  | 130.354 |  | 28.608 |  | 4.557 |  | < .001 |  | 74.283 |  | 186.426 |  |
| Factor 3 |  | B1Px |  | λ31 |  | 108.423 |  | 30.432 |  | 3.563 |  | < .001 |  | 48.778 |  | 168.068 |  |
|  |  | B1Py |  | λ32 |  | 113.610 |  | 31.913 |  | 3.560 |  | < .001 |  | 51.061 |  | 176.159 |  |
|  |  | B1Pz |  | λ33 |  | 83.654 |  | 23.932 |  | 3.496 |  | < .001 |  | 36.748 |  | 130.559 |  |
| Factor 4 |  | A1Px |  | λ41 |  | 227.284 |  | 27.286 |  | 8.330 |  | < .001 |  | 173.804 |  | 280.763 |  |
|  |  | A1Py |  | λ42 |  | 155.237 |  | 23.163 |  | 6.702 |  | < .001 |  | 109.839 |  | 200.635 |  |
|  |  | A1Pz |  | λ43 |  | 184.257 |  | 26.131 |  | 7.051 |  | < .001 |  | 133.042 |  | 235.472 |  |
|  | | | | | | | | | | | | | | | | | |

| **Second-order factor loadings** | | | | | | | | | | | | | | | | | |
| --- | --- | --- | --- | --- | --- | --- | --- | --- | --- | --- | --- | --- | --- | --- | --- | --- | --- |
|  | | | | | | | | | | | | | | **95% Confidence Interval** | | | |
| **Factor** | | **Indicator** | | **Symbol** | | **Estimate** | | **Std. Error** | | **z-value** | | **p** | | **Lower** | | **Upper** | |
| SecondOrder |  | Factor 1 |  | γ11 |  | 1.335 |  | 0.277 |  | 4.825 |  | < .001 |  | 0.793 |  | 1.877 |  |
|  |  | Factor 2 |  | γ12 |  | 1.581 |  | 0.407 |  | 3.884 |  | < .001 |  | 0.783 |  | 2.378 |  |
|  |  | Factor 3 |  | γ13 |  | 1.548 |  | 0.481 |  | 3.220 |  | 0.001 |  | 0.605 |  | 2.490 |  |
|  | | | | | | | | | | | | | | | | | |

| **Factor variances** | | | | | | | | | | | | | |
| --- | --- | --- | --- | --- | --- | --- | --- | --- | --- | --- | --- | --- | --- |
|  | | | | | | | | | | **95% Confidence Interval** | | | |
| **Factor** | | **Estimate** | | **Std. Error** | | **z-value** | | **p** | | **Lower** | | **Upper** | |
| Factor 1 |  | 1.000 |  | 0.000 |  |  |  |  |  | 1.000 |  | 1.000 |  |
| Factor 2 |  | 1.000 |  | 0.000 |  |  |  |  |  | 1.000 |  | 1.000 |  |
| Factor 3 |  | 1.000 |  | 0.000 |  |  |  |  |  | 1.000 |  | 1.000 |  |
| Factor 4 |  | 1.000 |  | 0.000 |  |  |  |  |  | 1.000 |  | 1.000 |  |
| Second-Order |  | 1.000 |  | 0.000 |  |  |  |  |  | 1.000 |  | 1.000 |  |
|  | | | | | | | | | | | | | |

| **Residual variances** | | | | | | | | | | | | | |
| --- | --- | --- | --- | --- | --- | --- | --- | --- | --- | --- | --- | --- | --- |
|  | | | | | | | | | | **95% Confidence Interval** | | | |
| **Indicator** | | **Estimate** | | **Std. Error** | | **z-value** | | **p** | | **Lower** | | **Upper** | |
| D1Px |  | 208563.753 |  | 13512.262 |  | 15.435 |  | < .001 |  | 182080.205 |  | 235047.301 |  |
| D1Py |  | 214051.194 |  | 10741.885 |  | 19.927 |  | < .001 |  | 192997.488 |  | 235104.901 |  |
| D1Pz |  | 201082.648 |  | 11858.530 |  | 16.957 |  | < .001 |  | 177840.357 |  | 224324.939 |  |
| C1Px |  | 199035.190 |  | 11275.701 |  | 17.652 |  | < .001 |  | 176935.223 |  | 221135.158 |  |
| C1Py |  | 217527.587 |  | 11348.908 |  | 19.167 |  | < .001 |  | 195284.136 |  | 239771.038 |  |
| C1Pz |  | 191022.669 |  | 11439.350 |  | 16.699 |  | < .001 |  | 168601.955 |  | 213443.383 |  |
| B1Px |  | 253158.929 |  | 13481.236 |  | 18.779 |  | < .001 |  | 226736.193 |  | 279581.666 |  |
| B1Py |  | 214611.139 |  | 12334.466 |  | 17.399 |  | < .001 |  | 190436.030 |  | 238786.248 |  |
| B1Pz |  | 217142.831 |  | 10809.612 |  | 20.088 |  | < .001 |  | 195956.381 |  | 238329.282 |  |
| A1Px |  | 210668.196 |  | 13875.796 |  | 15.182 |  | < .001 |  | 183472.137 |  | 237864.256 |  |
| A1Py |  | 203553.014 |  | 10579.889 |  | 19.240 |  | < .001 |  | 182816.812 |  | 224289.216 |  |
| A1Pz |  | 250523.167 |  | 13388.101 |  | 18.712 |  | < .001 |  | 224282.972 |  | 276763.362 |  |
|  | | | | | | | | | | | | | |

**Table 10.1.3 Experiential positive**

**Model fit**

| **Chi-square test** | | | | | | | |
| --- | --- | --- | --- | --- | --- | --- | --- |
| **Model** | | **Χ²** | | **df** | | **p** | |
| Baseline model |  | 901.663 |  | 66 |  |  |  |
| Factor model |  | 120.171 |  | 50 |  | < .001 |  |
|  | | | | | | | |

**Additional fit measures**

| **Fit indices** | | | |
| --- | --- | --- | --- |
| **Index** | | **Value** | |
| Comparative Fit Index (CFI) |  | 0.916 |  |
| Tucker-Lewis Index (TLI) |  | 0.889 |  |
| Bentler-Bonett Non-normed Fit Index (NNFI) |  | 0.889 |  |
| Bentler-Bonett Normed Fit Index (NFI) |  | 0.867 |  |
| Parsimony Normed Fit Index (PNFI) |  | 0.657 |  |
| Bollen's Relative Fit Index (RFI) |  | 0.824 |  |
| Bollen's Incremental Fit Index (IFI) |  | 0.918 |  |
| Relative Noncentrality Index (RNI) |  | 0.916 |  |
|  | | | |

| **Information criteria** | | | |
| --- | --- | --- | --- |
|  | | **Value** | |
| Log-likelihood |  | -93691.283 |  |
| Number of free parameters |  | 28.000 |  |
| Akaike (AIC) |  | 187438.567 |  |
| Bayesian (BIC) |  | 187576.839 |  |
| Sample-size adjusted Bayesian (SSABIC) |  | 187487.907 |  |
|  | | | |

| **Other fit measures** | | | |
| --- | --- | --- | --- |
| **Metric** | | **Value** | |
| Root mean square error of approximation (RMSEA) |  | 0.037 |  |
| RMSEA 90% CI lower bound |  | 0.028 |  |
| RMSEA 90% CI upper bound |  | 0.045 |  |
| RMSEA p-value |  | 0.995 |  |
| Standardized root mean square residual (SRMR) |  | 0.036 |  |
| Hoelter's critical N (α = .05) |  | 580.153 |  |
| Hoelter's critical N (α = .01) |  | 654.357 |  |
| Goodness of fit index (GFI) |  | 0.981 |  |
| McDonald fit index (MFI) |  | 0.967 |  |
| Expected cross validation index (ECVI) |  | 0.171 |  |
|  | | | |

| **R-Squared** | | | |
| --- | --- | --- | --- |
|  | | **R²** | |
| D2Px |  | 0.291 |  |
| D2Py |  | 0.251 |  |
| D2Pz |  | 0.315 |  |
| C2Px |  | 0.149 |  |
| C2Py |  | 0.101 |  |
| C2Pz |  | 0.240 |  |
| B2Px |  | 0.214 |  |
| B2Py |  | 0.106 |  |
| B2Pz |  | 0.294 |  |
| A2Px |  | 0.160 |  |
| A2Py |  | 0.156 |  |
| A2Pz |  | 0.125 |  |
| Factor 1 |  | 0.663 |  |
| Factor 2 |  | 0.777 |  |
| Factor 3 |  | 0.480 |  |
|  | | | |

**Parameter estimates**

| **Factor loadings** | | | | | | | | | | | | | | | | | |
| --- | --- | --- | --- | --- | --- | --- | --- | --- | --- | --- | --- | --- | --- | --- | --- | --- | --- |
|  | | | | | | | | | | | | | | **95% Confidence Interval** | | | |
| **Factor** | | **Indicator** | | **Symbol** | | **Estimate** | | **Std. Error** | | **z-value** | | **p** | | **Lower** | | **Upper** | |
| Factor 1 |  | D2Px |  | λ11 |  | 156.722 |  | 23.906 |  | 6.556 |  | < .001 |  | 109.867 |  | 203.578 |  |
|  |  | D2Py |  | λ12 |  | 147.171 |  | 22.669 |  | 6.492 |  | < .001 |  | 102.740 |  | 191.602 |  |
|  |  | D2Pz |  | λ13 |  | 169.828 |  | 25.863 |  | 6.566 |  | < .001 |  | 119.138 |  | 220.519 |  |
| Factor 2 |  | C2Px |  | λ21 |  | 91.910 |  | 29.111 |  | 3.157 |  | 0.002 |  | 34.852 |  | 148.967 |  |
|  |  | C2Py |  | λ22 |  | 77.799 |  | 25.093 |  | 3.100 |  | 0.002 |  | 28.619 |  | 126.980 |  |
|  |  | C2Pz |  | λ23 |  | 112.243 |  | 36.007 |  | 3.117 |  | 0.002 |  | 41.672 |  | 182.814 |  |
| Factor 3 |  | B2Px |  | λ31 |  | 166.027 |  | 21.342 |  | 7.779 |  | < .001 |  | 124.197 |  | 207.856 |  |
|  |  | B2Py |  | λ32 |  | 103.303 |  | 16.026 |  | 6.446 |  | < .001 |  | 71.893 |  | 134.713 |  |
|  |  | B2Pz |  | λ33 |  | 192.366 |  | 24.651 |  | 7.804 |  | < .001 |  | 144.052 |  | 240.681 |  |
| Factor 4 |  | A2Px |  | λ41 |  | 169.438 |  | 21.642 |  | 7.829 |  | < .001 |  | 127.021 |  | 211.855 |  |
|  |  | A2Py |  | λ42 |  | 172.842 |  | 22.253 |  | 7.767 |  | < .001 |  | 129.228 |  | 216.456 |  |
|  |  | A2Pz |  | λ43 |  | 182.318 |  | 25.550 |  | 7.136 |  | < .001 |  | 132.240 |  | 232.396 |  |
|  | | | | | | | | | | | | | | | | | |

| **Second-order factor loadings** | | | | | | | | | | | | | | | | | |
| --- | --- | --- | --- | --- | --- | --- | --- | --- | --- | --- | --- | --- | --- | --- | --- | --- | --- |
|  | | | | | | | | | | | | | | **95% Confidence Interval** | | | |
| **Factor** | | **Indicator** | | **Symbol** | | **Estimate** | | **Std. Error** | | **z-value** | | **p** | | **Lower** | | **Upper** | |
| SecondOrder |  | Factor 1 |  | γ11 |  | 1.401 |  | 0.273 |  | 5.134 |  | < .001 |  | 0.866 |  | 1.936 |  |
|  |  | Factor 2 |  | γ12 |  | 1.865 |  | 0.660 |  | 2.827 |  | 0.005 |  | 0.572 |  | 3.159 |  |
|  |  | Factor 3 |  | γ13 |  | 0.960 |  | 0.155 |  | 6.195 |  | < .001 |  | 0.656 |  | 1.264 |  |
|  | | | | | | | | | | | | | | | | | |

| **Factor variances** | | | | | | | | | | | | | |
| --- | --- | --- | --- | --- | --- | --- | --- | --- | --- | --- | --- | --- | --- |
|  | | | | | | | | | | **95% Confidence Interval** | | | |
| **Factor** | | **Estimate** | | **Std. Error** | | **z-value** | | **p** | | **Lower** | | **Upper** | |
| Factor 1 |  | 1.000 |  | 0.000 |  |  |  |  |  | 1.000 |  | 1.000 |  |
| Factor 2 |  | 1.000 |  | 0.000 |  |  |  |  |  | 1.000 |  | 1.000 |  |
| Factor 3 |  | 1.000 |  | 0.000 |  |  |  |  |  | 1.000 |  | 1.000 |  |
| Factor 4 |  | 1.000 |  | 0.000 |  |  |  |  |  | 1.000 |  | 1.000 |  |
| Second-Order |  | 1.000 |  | 0.000 |  |  |  |  |  | 1.000 |  | 1.000 |  |
|  | | | | | | | | | | | | | |

| **Residual variances** | | | | | | | | | | | | | |
| --- | --- | --- | --- | --- | --- | --- | --- | --- | --- | --- | --- | --- | --- |
|  | | | | | | | | | | **95% Confidence Interval** | | | |
| **Indicator** | | **Estimate** | | **Std. Error** | | **z-value** | | **p** | | **Lower** | | **Upper** | |
| D2Px |  | 177394.971 |  | 10599.636 |  | 16.736 |  | < .001 |  | 156620.065 |  | 198169.876 |  |
| D2Py |  | 191615.419 |  | 10724.191 |  | 17.868 |  | < .001 |  | 170596.391 |  | 212634.448 |  |
| D2Pz |  | 186144.431 |  | 11626.947 |  | 16.010 |  | < .001 |  | 163356.034 |  | 208932.828 |  |
| C2Px |  | 216516.935 |  | 11282.311 |  | 19.191 |  | < .001 |  | 194404.012 |  | 238629.858 |  |
| C2Py |  | 241054.970 |  | 11748.649 |  | 20.518 |  | < .001 |  | 218028.041 |  | 264081.899 |  |
| C2Pz |  | 178951.056 |  | 11351.879 |  | 15.764 |  | < .001 |  | 156701.782 |  | 201200.331 |  |
| B2Px |  | 195116.843 |  | 11490.600 |  | 16.981 |  | < .001 |  | 172595.681 |  | 217638.004 |  |
| B2Py |  | 173412.665 |  | 8541.008 |  | 20.304 |  | < .001 |  | 156672.597 |  | 190152.734 |  |
| B2Pz |  | 170951.470 |  | 12329.728 |  | 13.865 |  | < .001 |  | 146785.646 |  | 195117.293 |  |
| A2Px |  | 150679.935 |  | 8913.332 |  | 16.905 |  | < .001 |  | 133210.125 |  | 168149.744 |  |
| A2Py |  | 161187.794 |  | 9442.411 |  | 17.071 |  | < .001 |  | 142681.008 |  | 179694.581 |  |
| A2Pz |  | 232741.326 |  | 12638.046 |  | 18.416 |  | < .001 |  | 207971.211 |  | 257511.441 |  |
|  | | | | | | | | | | | | | |

**Table 10.1.4 Aspirational positive**

**Model fit**

| **Chi-square test** | | | | | | | |
| --- | --- | --- | --- | --- | --- | --- | --- |
| **Model** | | **Χ²** | | **df** | | **p** | |
| Baseline model |  | 865.688 |  | 55 |  |  |  |
| Factor model |  | 83.479 |  | 50 |  | < .001 |  |
|  | | | | | | | |

**Additional fit measures**

| **Fit indices** | | | |
| --- | --- | --- | --- |
| **Index** | | **Value** | |
| Comparative Fit Index (CFI) |  | 0.946 |  |
| Tucker-Lewis Index (TLI) |  | 0.926 |  |
| Bentler-Bonett Non-normed Fit Index (NNFI) |  | 0.926 |  |
| Bentler-Bonett Normed Fit Index (NFI) |  | 0.904 |  |
| Parsimony Normed Fit Index (PNFI) |  | 0.657 |  |
| Bollen's Relative Fit Index (RFI) |  | 0.867 |  |
| Bollen's Incremental Fit Index (IFI) |  | 0.947 |  |
| Relative Noncentrality Index (RNI) |  | 0.946 |  |
|  | | | |

| **Information criteria** | | | |
| --- | --- | --- | --- |
|  | | **Value** | |
| Log-likelihood |  | -86138.761 |  |
| Number of free parameters |  | 26.000 |  |
| Akaike (AIC) |  | 172329.521 |  |
| Bayesian (BIC) |  | 172457.917 |  |
| Sample-size adjusted Bayesian (SSABIC) |  | 172375.338 |  |
|  | | | |

| **Other fit measures** | | | |
| --- | --- | --- | --- |
| **Metric** | | **Value** | |
| Root mean square error of approximation (RMSEA) |  | 0.032 |  |
| RMSEA 90% CI lower bound |  | 0.023 |  |
| RMSEA 90% CI upper bound |  | 0.042 |  |
| RMSEA p-value |  | 0.999 |  |
| Standardized root mean square residual (SRMR) |  | 0.032 |  |
| Hoelter's critical N (α = .05) |  | 689.640 |  |
| Hoelter's critical N (α = .01) |  | 787.606 |  |
| Goodness of fit index (GFI) |  | 0.986 |  |
| McDonald fit index (MFI) |  | 0.979 |  |
| Expected cross validation index (ECVI) |  | 0.131 |  |
|  | | | |

| **R-Squared** | | | |
| --- | --- | --- | --- |
|  | | **R²** | |
| D3Px |  | 0.196 |  |
| D3Py |  | 0.194 |  |
| D3Pz |  | 0.284 |  |
| C3Px |  | 0.331 |  |
| C3Py |  | 0.287 |  |
| C3Pz |  | 0.191 |  |
| B3Px |  | 0.253 |  |
| B3Py |  | 0.176 |  |
| B3Pz |  | 0.170 |  |
| A3Px |  | 0.053 |  |
| A3Py |  | 0.284 |  |
| A3Pz |  | 0.262 |  |
| Factor 1 |  | 0.160 |  |
| Factor 2 |  | 0.822 |  |
| Factor 3 |  | 0.986 |  |
|  | | | |

**Parameter estimates**

| **Factor loadings** | | | | | | | | | | | | | | | | | |
| --- | --- | --- | --- | --- | --- | --- | --- | --- | --- | --- | --- | --- | --- | --- | --- | --- | --- |
|  | | | | | | | | | | | | | | **95% Confidence Interval** | | | |
| **Factor** | | **Indicator** | | **Symbol** | | **Estimate** | | **Std. Error** | | **z-value** | | **p** | | **Lower** | | **Upper** | |
| Factor 1 |  | D3Px |  | λ11 |  | 201.930 |  | 22.285 |  | 9.061 |  | < .001 |  | 158.252 |  | 245.607 |  |
|  |  | D3Py |  | λ12 |  | 200.653 |  | 22.214 |  | 9.033 |  | < .001 |  | 157.115 |  | 244.191 |  |
|  |  | D3Pz |  | λ13 |  | 237.031 |  | 24.143 |  | 9.818 |  | < .001 |  | 189.711 |  | 284.351 |  |
| Factor 2 |  | C3Px |  | λ21 |  | 117.543 |  | 39.792 |  | 2.954 |  | 0.003 |  | 39.553 |  | 195.534 |  |
|  |  | C3Py |  | λ22 |  | 113.893 |  | 38.448 |  | 2.962 |  | 0.003 |  | 38.537 |  | 189.249 |  |
|  |  | C3Pz |  | λ23 |  | 91.173 |  | 30.930 |  | 2.948 |  | 0.003 |  | 30.551 |  | 151.794 |  |
| Factor 3 |  | B3Px |  | λ31 |  | 30.391 |  | 159.305 |  | 0.191 |  | 0.849 |  | -281.841 |  | 342.624 |  |
|  |  | B3Py |  | λ32 |  | 24.992 |  | 130.910 |  | 0.191 |  | 0.849 |  | -231.587 |  | 281.572 |  |
|  |  | B3Pz |  | λ33 |  | 25.346 |  | 132.757 |  | 0.191 |  | 0.849 |  | -234.854 |  | 285.546 |  |
| Factor 4 |  | A3Px |  | λ41 |  | 117.602 |  | 26.876 |  | 4.376 |  | < .001 |  | 64.926 |  | 170.278 |  |
|  |  | A3Py |  | λ42 |  | 207.43 |  | 11.983 |  | 17.31 |  | < .001 |  | 183.943 |  | 230.918 |  |
|  |  | A3Pz |  | λ43 |  | 240.652 |  | 46.731 |  | 5.150 |  | < .001 |  | 149.061 |  | 332.244 |  |
|  | | | | | | | | | | | | | | | | | |

| **Second-order factor loadings** | | | | | | | | | | | | | | | | | |
| --- | --- | --- | --- | --- | --- | --- | --- | --- | --- | --- | --- | --- | --- | --- | --- | --- | --- |
|  | | | | | | | | | | | | | | **95% Confidence Interval** | | | |
| **Factor** | | **Indicator** | | **Symbol** | | **Estimate** | | **Std. Error** | | **z-value** | | **p** | | **Lower** | | **Upper** | |
| SecondOrder |  | Factor 1 |  | γ11 |  | 0.437 |  | 0.071 |  | 6.116 |  | < .001 |  | 0.297 |  | 0.577 |  |
|  |  | Factor 2 |  | γ12 |  | 2.150 |  | 0.845 |  | 2.545 |  | 0.011 |  | 0.494 |  | 3.805 |  |
|  |  | Factor 3 |  | γ13 |  | 8.423 |  | 44.594 |  | 0.189 |  | 0.850 |  | -78.980 |  | 95.825 |  |
|  | | | | | | | | | | | | | | | | | |

| **Factor variances** | | | | | | | | | | | | | |
| --- | --- | --- | --- | --- | --- | --- | --- | --- | --- | --- | --- | --- | --- |
|  | | | | | | | | | | **95% Confidence Interval** | | | |
| **Factor** | | **Estimate** | | **Std. Error** | | **z-value** | | **p** | | **Lower** | | **Upper** | |
| Factor 1 |  | 1.000 |  | 0.000 |  |  |  |  |  | 1.000 |  | 1.000 |  |
| Factor 2 |  | 1.000 |  | 0.000 |  |  |  |  |  | 1.000 |  | 1.000 |  |
| Factor 3 |  | 1.000 |  | 0.000 |  |  |  |  |  | 1.000 |  | 1.000 |  |
| Factor 4 |  | 1.000 |  | 0.000 |  |  |  |  |  | 1.000 |  | 1.000 |  |
| Second-Order |  | 1.000 |  | 0.000 |  |  |  |  |  | 1.000 |  | 1.000 |  |
|  | | | | | | | | | | | | | |

| **Residual variances** | | | | | | | | | | | | | |
| --- | --- | --- | --- | --- | --- | --- | --- | --- | --- | --- | --- | --- | --- |
|  | | | | | | | | | | **95% Confidence Interval** | | | |
| **Indicator** | | **Estimate** | | **Std. Error** | | **z-value** | | **p** | | **Lower** | | **Upper** | |
| D3Px |  | 198689.524 |  | 11925.816 |  | 16.660 |  | < .001 |  | 175315.355 |  | 222063.693 |  |
| D3Py |  | 198777.045 |  | 11870.166 |  | 16.746 |  | < .001 |  | 175511.947 |  | 222042.142 |  |
| D3Pz |  | 168972.225 |  | 12982.893 |  | 13.015 |  | < .001 |  | 143526.223 |  | 194418.228 |  |
| C3Px |  | 157062.292 |  | 9855.056 |  | 15.937 |  | < .001 |  | 137746.736 |  | 176377.847 |  |
| C3Py |  | 180945.267 |  | 10484.576 |  | 17.258 |  | < .001 |  | 160395.875 |  | 201494.659 |  |
| C3Pz |  | 197417.577 |  | 10079.287 |  | 19.586 |  | < .001 |  | 177662.537 |  | 217172.617 |  |
| B3Px |  | 196583.364 |  | 11394.910 |  | 17.252 |  | < .001 |  | 174249.752 |  | 218916.977 |  |
| B3Py |  | 211049.699 |  | 10817.414 |  | 19.510 |  | < .001 |  | 189847.957 |  | 232251.442 |  |
| B3Pz |  | 225745.803 |  | 11490.302 |  | 19.647 |  | < .001 |  | 203225.225 |  | 248266.382 |  |
| A3Px |  | 245899.344 |  | 11987.121 |  | 20.514 |  | < .001 |  | 222405.019 |  | 269393.669 |  |
| A3Py |  | 164041.112 |  |  |  |  |  | < .001 |  |  |  |  |  |
| A3Pz |  | 162744.375 |  | 22675.868 |  | 7.177 |  | < .001 |  | 118300.491 |  | 207188.260 |  |
|  | | | | | | | | | | | | | |
