# Supplementary material for: The structure of human motivation
Source: BMC Psychol. 2023 Oct 6;11:308. doi: 10.1186/s40359-023-01346-5 (PMC10557177; doi:10.1186/s40359-023-01346-5)
Supplement: Supplementary file 6 — Additional file 6: SM Table 11. Confirmatory factor models for adjacent and antipodal life domains: Full output. [file 40359_2023_1346_MOESM6_ESM.zip › Table 11.1 CFA.adjacencies and antipodes.positiveR5.docx]

**Table 11.1.1 Adjacent positive A-B**

**Model fit**

| **Chi-square test** | | | | | | | |
| --- | --- | --- | --- | --- | --- | --- | --- |
| **Model** | | **Χ²** | | **df** | | **p** | |
| Baseline model |  | 1348.300 |  | 153 |  |  |  |
| Factor model |  | 462.225 |  | 133 |  | < .001 |  |
|  | | | | | | | |

**Additional fit measures**

| **Fit indices** | | | |
| --- | --- | --- | --- |
| **Index** | | **Value** | |
| Comparative Fit Index (CFI) |  | 0.725 |  |
| Tucker-Lewis Index (TLI) |  | 0.683 |  |
| Bentler-Bonett Non-normed Fit Index (NNFI) |  | 0.683 |  |
| Bentler-Bonett Normed Fit Index (NFI) |  | 0.657 |  |
| Parsimony Normed Fit Index (PNFI) |  | 0.571 |  |
| Bollen's Relative Fit Index (RFI) |  | 0.606 |  |
| Bollen's Incremental Fit Index (IFI) |  | 0.729 |  |
| Relative Noncentrality Index (RNI) |  | 0.725 |  |
|  | | | |

| **Information criteria** | | | |
| --- | --- | --- | --- |
|  | | **Value** | |
| Log-likelihood |  | -140806.131 |  |
| Number of free parameters |  | 38.000 |  |
| Akaike (AIC) |  | 281688.262 |  |
| Bayesian (BIC) |  | 281875.917 |  |
| Sample-size adjusted Bayesian (SSABIC) |  | 281755.225 |  |
|  | | | |

| **Other fit measures** | | | |
| --- | --- | --- | --- |
| **Metric** | | **Value** | |
| Root mean square error of approximation (RMSEA) |  | 0.049 |  |
| RMSEA 90% CI lower bound |  | 0.044 |  |
| RMSEA 90% CI upper bound |  | 0.054 |  |
| RMSEA p-value |  | 0.624 |  |
| Standardized root mean square residual (SRMR) |  | 0.049 |  |
| Hoelter's critical N (α = .05) |  | 359.923 |  |
| Hoelter's critical N (α = .01) |  | 388.784 |  |
| Goodness of fit index (GFI) |  | 0.945 |  |
| McDonald fit index (MFI) |  | 0.852 |  |
| Expected cross validation index (ECVI) |  | 0.522 |  |
|  | | | |

| **R-Squared** | | | |
| --- | --- | --- | --- |
|  | | **R²** | |
| B1Px |  | 0.113 |  |
| B1Py |  | 0.080 |  |
| B1Pz |  | 0.100 |  |
| B2Px |  | 0.141 |  |
| B2Py |  | 0.076 |  |
| B2Pz |  | 0.140 |  |
| B3Px |  | 0.090 |  |
| B3Py |  | 0.153 |  |
| B3Pz |  | 0.118 |  |
| A1Px |  | 0.094 |  |
| A1Py |  | 0.120 |  |
| A1Pz |  | 0.054 |  |
| A2Px |  | 0.056 |  |
| A2Py |  | 0.044 |  |
| A2Pz |  | 0.077 |  |
| A3Px |  | 0.212 |  |
| A3Py |  | 0.140 |  |
| A3Pz |  | 0.182 |  |
| Factor 1 |  | 1.000 |  |
|  | | | |

**Parameter estimates**

| **Factor loadings** | | | | | | | | | | | | | | | | | |
| --- | --- | --- | --- | --- | --- | --- | --- | --- | --- | --- | --- | --- | --- | --- | --- | --- | --- |
|  | | | | | | | | | | | | | | **95% Confidence Interval** | | | |
| **Factor** | | **Indicator** | | **Symbol** | | **Estimate** | | **Std. Error** | | **z-value** | | **p** | | **Lower** | | **Upper** | |
| Factor 1 |  | B1Px |  | λ11 |  | 8.777 |  |  |  |  |  |  |  |  |  |  |  |
|  |  | B1Py |  | λ12 |  | 6.900 |  |  |  |  |  |  |  |  |  |  |  |
|  |  | B1Pz |  | λ13 |  | 7.468 |  |  |  |  |  |  |  |  |  |  |  |
|  |  | B2Px |  | λ14 |  | 8.987 |  |  |  |  |  |  |  |  |  |  |  |
|  |  | B2Py |  | λ15 |  | 5.855 |  |  |  |  |  |  |  |  |  |  |  |
|  |  | B2Pz |  | λ16 |  | 8.871 |  |  |  |  |  |  |  |  |  |  |  |
|  |  | B3Px |  | λ17 |  | 7.411 |  |  |  |  |  |  |  |  |  |  |  |
|  |  | B3Py |  | λ18 |  | 9.520 |  |  |  |  |  |  |  |  |  |  |  |
|  |  | B3Pz |  | λ19 |  | 8.623 |  |  |  |  |  |  |  |  |  |  |  |
| Factor 2 |  | A1Px |  | λ21 |  | 157.221 |  |  |  |  |  |  |  |  |  |  |  |
|  |  | A1Py |  | λ22 |  | 165.462 |  |  |  |  |  |  |  |  |  |  |  |
|  |  | A1Pz |  | λ23 |  | 124.435 |  |  |  |  |  |  |  |  |  |  |  |
|  |  | A2Px |  | λ24 |  | 99.875 |  |  |  |  |  |  |  |  |  |  |  |
|  |  | A2Py |  | λ25 |  | 91.819 |  |  |  |  |  |  |  |  |  |  |  |
|  |  | A2Pz |  | λ26 |  | 143.380 |  |  |  |  |  |  |  |  |  |  |  |
|  |  | A3Px |  | λ27 |  | 213.016 |  |  |  |  |  |  |  |  |  |  |  |
|  |  | A3Py |  | λ28 |  | 178.941 |  |  |  |  |  |  |  |  |  |  |  |
|  |  | A3Pz |  | λ29 |  | 200.473 |  |  |  |  |  |  |  |  |  |  |  |
|  | | | | | | | | | | | | | | | | | |

| **Second-order factor loadings** | | | | | | | | | | | | | | | | | |
| --- | --- | --- | --- | --- | --- | --- | --- | --- | --- | --- | --- | --- | --- | --- | --- | --- | --- |
|  | | | | | | | | | | | | | | **95% Confidence Interval** | | | |
| **Factor** | | **Indicator** | | **Symbol** | | **Estimate** | | **Std. Error** | | **z-value** | | **p** | | **Lower** | | **Upper** | |
| SecondOrder |  | Factor 1 |  | γ11 |  | 20.775 |  |  |  |  |  |  |  |  |  |  |  |
|  | | | | | | | | | | | | | | | | | |

| **Factor variances** | | | | | | | | | | | | | |
| --- | --- | --- | --- | --- | --- | --- | --- | --- | --- | --- | --- | --- | --- |
|  | | | | | | | | | | **95% Confidence Interval** | | | |
| **Factor** | | **Estimate** | | **Std. Error** | | **z-value** | | **p** | | **Lower** | | **Upper** | |
| Factor 1 |  | 0.000 |  | 0.000 |  |  |  |  |  | 0.000 |  | 0.000 |  |
| Factor 2 |  | 1.000 |  | 0.000 |  |  |  |  |  | 1.000 |  | 1.000 |  |
| Second-Order |  | 1.000 |  | 0.000 |  |  |  |  |  | 1.000 |  | 1.000 |  |
|  | | | | | | | | | | | | | |

| **Residual variances** | | | | | | | | | | | | | |
| --- | --- | --- | --- | --- | --- | --- | --- | --- | --- | --- | --- | --- | --- |
|  | | | | | | | | | | **95% Confidence Interval** | | | |
| **Indicator** | | **Estimate** | | **Std. Error** | | **z-value** | | **p** | | **Lower** | | **Upper** | |
| B1Px |  | 259815.742 |  |  |  |  |  |  |  |  |  |  |  |
| B1Py |  | 237879.155 |  |  |  |  |  |  |  |  |  |  |  |
| B1Pz |  | 216832.644 |  |  |  |  |  |  |  |  |  |  |  |
| B2Px |  | 213228.850 |  |  |  |  |  |  |  |  |  |  |  |
| B2Py |  | 179124.107 |  |  |  |  |  |  |  |  |  |  |  |
| B2Pz |  | 208099.520 |  |  |  |  |  |  |  |  |  |  |  |
| B3Px |  | 239326.861 |  |  |  |  |  |  |  |  |  |  |  |
| B3Py |  | 216870.335 |  |  |  |  |  |  |  |  |  |  |  |
| B3Pz |  | 239866.154 |  |  |  |  |  |  |  |  |  |  |  |
| A1Px |  | 237607.560 |  |  |  |  |  |  |  |  |  |  |  |
| A1Py |  | 200274.056 |  |  |  |  |  |  |  |  |  |  |  |
| A1Pz |  | 268989.893 |  |  |  |  |  |  |  |  |  |  |  |
| A2Px |  | 169414.530 |  |  |  |  |  |  |  |  |  |  |  |
| A2Py |  | 182631.767 |  |  |  |  |  |  |  |  |  |  |  |
| A2Pz |  | 245423.541 |  |  |  |  |  |  |  |  |  |  |  |
| A3Px |  | 168505.935 |  |  |  |  |  |  |  |  |  |  |  |
| A3Py |  | 197103.449 |  |  |  |  |  |  |  |  |  |  |  |
| A3Pz |  | 180468.593 |  |  |  |  |  |  |  |  |  |  |  |
|  | | | | | | | | | | | | | |

**Table 11.1.2 Adjacent positive B-C**

**Model fit**

| **Chi-square test** | | | | | | | |
| --- | --- | --- | --- | --- | --- | --- | --- |
| **Model** | | **Χ²** | | **df** | | **p** | |
| Baseline model |  | 1718.024 |  | 153 |  |  |  |
| Factor model |  | 406.902 |  | 133 |  | < .001 |  |
|  | | | | | | | |

**Additional fit measures**

| **Fit indices** | | | |
| --- | --- | --- | --- |
| **Index** | | **Value** | |
| Comparative Fit Index (CFI) |  | 0.825 |  |
| Tucker-Lewis Index (TLI) |  | 0.799 |  |
| Bentler-Bonett Non-normed Fit Index (NNFI) |  | 0.799 |  |
| Bentler-Bonett Normed Fit Index (NFI) |  | 0.763 |  |
| Parsimony Normed Fit Index (PNFI) |  | 0.663 |  |
| Bollen's Relative Fit Index (RFI) |  | 0.728 |  |
| Bollen's Incremental Fit Index (IFI) |  | 0.827 |  |
| Relative Noncentrality Index (RNI) |  | 0.825 |  |
|  | | | |

| **Information criteria** | | | |
| --- | --- | --- | --- |
|  | | **Value** | |
| Log-likelihood |  | -141017.419 |  |
| Number of free parameters |  | 38.000 |  |
| Akaike (AIC) |  | 282110.838 |  |
| Bayesian (BIC) |  | 282298.493 |  |
| Sample-size adjusted Bayesian (SSABIC) |  | 282177.800 |  |
|  | | | |

| **Other fit measures** | | | |
| --- | --- | --- | --- |
| **Metric** | | **Value** | |
| Root mean square error of approximation (RMSEA) |  | 0.045 |  |
| RMSEA 90% CI lower bound |  | 0.040 |  |
| RMSEA 90% CI upper bound |  | 0.050 |  |
| RMSEA p-value |  | 0.960 |  |
| Standardized root mean square residual (SRMR) |  | 0.045 |  |
| Hoelter's critical N (α = .05) |  | 408.723 |  |
| Hoelter's critical N (α = .01) |  | 441.508 |  |
| Goodness of fit index (GFI) |  | 0.953 |  |
| McDonald fit index (MFI) |  | 0.876 |  |
| Expected cross validation index (ECVI) |  | 0.468 |  |
|  | | | |

| **R-Squared** | | | |
| --- | --- | --- | --- |
|  | | **R²** | |
| B1Px |  | 0.048 |  |
| B1Py |  | 0.103 |  |
| B1Pz |  | 0.103 |  |
| B2Px |  | 0.093 |  |
| B2Py |  | 0.059 |  |
| B2Pz |  | 0.112 |  |
| B3Px |  | 0.173 |  |
| B3Py |  | 0.148 |  |
| B3Pz |  | 0.165 |  |
| C1Px |  | 0.201 |  |
| C1Py |  | 0.206 |  |
| C1Pz |  | 0.143 |  |
| C2Px |  | 0.128 |  |
| C2Py |  | 0.052 |  |
| C2Pz |  | 0.166 |  |
| C3Px |  | 0.217 |  |
| C3Py |  | 0.226 |  |
| C3Pz |  | 0.202 |  |
| Factor 1 |  | 1.000 |  |
|  | | | |

**Parameter estimates**

| **Factor loadings** | | | | | | | | | | | | | | | | | |
| --- | --- | --- | --- | --- | --- | --- | --- | --- | --- | --- | --- | --- | --- | --- | --- | --- | --- |
|  | | | | | | | | | | | | | | **95% Confidence Interval** | | | |
| **Factor** | | **Indicator** | | **Symbol** | | **Estimate** | | **Std. Error** | | **z-value** | | **p** | | **Lower** | | **Upper** | |
| Factor 1 |  | B1Px |  | λ11 |  | 5.399 |  |  |  |  |  |  |  |  |  |  |  |
|  |  | B1Py |  | λ12 |  | 7.454 |  |  |  |  |  |  |  |  |  |  |  |
|  |  | B1Pz |  | λ13 |  | 7.184 |  |  |  |  |  |  |  |  |  |  |  |
|  |  | B2Px |  | λ14 |  | 6.935 |  |  |  |  |  |  |  |  |  |  |  |
|  |  | B2Py |  | λ15 |  | 4.892 |  |  |  |  |  |  |  |  |  |  |  |
|  |  | B2Pz |  | λ16 |  | 7.508 |  |  |  |  |  |  |  |  |  |  |  |
|  |  | B3Px |  | λ17 |  | 9.746 |  |  |  |  |  |  |  |  |  |  |  |
|  |  | B3Py |  | λ18 |  | 8.876 |  |  |  |  |  |  |  |  |  |  |  |
|  |  | B3Pz |  | λ19 |  | 9.661 |  |  |  |  |  |  |  |  |  |  |  |
| Factor 2 |  | C1Px |  | λ21 |  | 225.179 |  |  |  |  |  |  |  |  |  |  |  |
|  |  | C1Py |  | λ22 |  | 230.742 |  |  |  |  |  |  |  |  |  |  |  |
|  |  | C1Pz |  | λ23 |  | 189.251 |  |  |  |  |  |  |  |  |  |  |  |
|  |  | C2Px |  | λ24 |  | 180.137 |  |  |  |  |  |  |  |  |  |  |  |
|  |  | C2Py |  | λ25 |  | 118.104 |  |  |  |  |  |  |  |  |  |  |  |
|  |  | C2Pz |  | λ26 |  | 197.560 |  |  |  |  |  |  |  |  |  |  |  |
|  |  | C3Px |  | λ27 |  | 225.890 |  |  |  |  |  |  |  |  |  |  |  |
|  |  | C3Py |  | λ28 |  | 239.671 |  |  |  |  |  |  |  |  |  |  |  |
|  |  | C3Pz |  | λ29 |  | 221.931 |  |  |  |  |  |  |  |  |  |  |  |
|  | | | | | | | | | | | | | | | | | |

| **Second-order factor loadings** | | | | | | | | | | | | | | | | | |
| --- | --- | --- | --- | --- | --- | --- | --- | --- | --- | --- | --- | --- | --- | --- | --- | --- | --- |
|  | | | | | | | | | | | | | | **95% Confidence Interval** | | | |
| **Factor** | | **Indicator** | | **Symbol** | | **Estimate** | | **Std. Error** | | **z-value** | | **p** | | **Lower** | | **Upper** | |
| SecondOrder |  | Factor 1 |  | γ11 |  | 21.894 |  |  |  |  |  |  |  |  |  |  |  |
|  | | | | | | | | | | | | | | | | | |

| **Factor variances** | | | | | | | | | | | | | |
| --- | --- | --- | --- | --- | --- | --- | --- | --- | --- | --- | --- | --- | --- |
|  | | | | | | | | | | **95% Confidence Interval** | | | |
| **Factor** | | **Estimate** | | **Std. Error** | | **z-value** | | **p** | | **Lower** | | **Upper** | |
| Factor 1 |  | 0.000 |  | 0.000 |  |  |  |  |  | 0.000 |  | 0.000 |  |
| Factor 2 |  | 1.000 |  | 0.000 |  |  |  |  |  | 1.000 |  | 1.000 |  |
| Second-Order |  | 1.000 |  | 0.000 |  |  |  |  |  | 1.000 |  | 1.000 |  |
|  | | | | | | | | | | | | | |

| **Residual variances** | | | | | | | | | | | | | |
| --- | --- | --- | --- | --- | --- | --- | --- | --- | --- | --- | --- | --- | --- |
|  | | | | | | | | | | **95% Confidence Interval** | | | |
| **Indicator** | | **Estimate** | | **Std. Error** | | **z-value** | | **p** | | **Lower** | | **Upper** | |
| B1Px |  | 279094.231 |  |  |  |  |  |  |  |  |  |  |  |
| B1Py |  | 231796.188 |  |  |  |  |  |  |  |  |  |  |  |
| B1Pz |  | 216160.372 |  |  |  |  |  |  |  |  |  |  |  |
| B2Px |  | 225033.163 |  |  |  |  |  |  |  |  |  |  |  |
| B2Py |  | 182451.203 |  |  |  |  |  |  |  |  |  |  |  |
| B2Pz |  | 215043.530 |  |  |  |  |  |  |  |  |  |  |  |
| B3Px |  | 217502.098 |  |  |  |  |  |  |  |  |  |  |  |
| B3Py |  | 218222.725 |  |  |  |  |  |  |  |  |  |  |  |
| B3Pz |  | 227222.601 |  |  |  |  |  |  |  |  |  |  |  |
| C1Px |  | 201071.705 |  |  |  |  |  |  |  |  |  |  |  |
| C1Py |  | 205428.540 |  |  |  |  |  |  |  |  |  |  |  |
| C1Pz |  | 214651.727 |  |  |  |  |  |  |  |  |  |  |  |
| C2Px |  | 221912.703 |  |  |  |  |  |  |  |  |  |  |  |
| C2Py |  | 254222.716 |  |  |  |  |  |  |  |  |  |  |  |
| C2Pz |  | 196363.862 |  |  |  |  |  |  |  |  |  |  |  |
| C3Px |  | 183703.147 |  |  |  |  |  |  |  |  |  |  |  |
| C3Py |  | 196421.473 |  |  |  |  |  |  |  |  |  |  |  |
| C3Pz |  | 194891.364 |  |  |  |  |  |  |  |  |  |  |  |
|  | | | | | | | | | | | | | |

**Table 11.1.3 Adjacent positive C-D**

**Model fit**

| **Chi-square test** | | | | | | | |
| --- | --- | --- | --- | --- | --- | --- | --- |
| **Model** | | **Χ²** | | **df** | | **p** | |
| Baseline model |  | 1879.419 |  | 153 |  |  |  |
| Factor model |  | 502.737 |  | 133 |  | < .001 |  |
|  | | | | | | | |

**Additional fit measures**

| **Fit indices** | | | |
| --- | --- | --- | --- |
| **Index** | | **Value** | |
| Comparative Fit Index (CFI) |  | 0.786 |  |
| Tucker-Lewis Index (TLI) |  | 0.754 |  |
| Bentler-Bonett Non-normed Fit Index (NNFI) |  | 0.754 |  |
| Bentler-Bonett Normed Fit Index (NFI) |  | 0.733 |  |
| Parsimony Normed Fit Index (PNFI) |  | 0.637 |  |
| Bollen's Relative Fit Index (RFI) |  | 0.692 |  |
| Bollen's Incremental Fit Index (IFI) |  | 0.788 |  |
| Relative Noncentrality Index (RNI) |  | 0.786 |  |
|  | | | |

| **Information criteria** | | | |
| --- | --- | --- | --- |
|  | | **Value** | |
| Log-likelihood |  | -141078.536 |  |
| Number of free parameters |  | 38.000 |  |
| Akaike (AIC) |  | 282233.071 |  |
| Bayesian (BIC) |  | 282420.726 |  |
| Sample-size adjusted Bayesian (SSABIC) |  | 282300.034 |  |
|  | | | |

| **Other fit measures** | | | |
| --- | --- | --- | --- |
| **Metric** | | **Value** | |
| Root mean square error of approximation (RMSEA) |  | 0.052 |  |
| RMSEA 90% CI lower bound |  | 0.047 |  |
| RMSEA 90% CI upper bound |  | 0.057 |  |
| RMSEA p-value |  | 0.249 |  |
| Standardized root mean square residual (SRMR) |  | 0.049 |  |
| Hoelter's critical N (α = .05) |  | 331.000 |  |
| Hoelter's critical N (α = .01) |  | 357.535 |  |
| Goodness of fit index (GFI) |  | 0.944 |  |
| McDonald fit index (MFI) |  | 0.836 |  |
| Expected cross validation index (ECVI) |  | 0.561 |  |
|  | | | |

| **R-Squared** | | | |
| --- | --- | --- | --- |
|  | | **R²** | |
| D1Px |  | 0.175 |  |
| D1Py |  | 0.099 |  |
| D1Pz |  | 0.112 |  |
| D2Px |  | 0.176 |  |
| D2Py |  | 0.184 |  |
| D2Pz |  | 0.202 |  |
| D3Px |  | 0.075 |  |
| D3Py |  | 0.060 |  |
| D3Pz |  | 0.153 |  |
| C1Px |  | 0.203 |  |
| C1Py |  | 0.207 |  |
| C1Pz |  | 0.162 |  |
| C2Px |  | 0.156 |  |
| C2Py |  | 0.059 |  |
| C2Pz |  | 0.243 |  |
| C3Px |  | 0.160 |  |
| C3Py |  | 0.173 |  |
| C3Pz |  | 0.179 |  |
| Factor 1 |  | 1.000 |  |
|  | | | |

**Parameter estimates**

| **Factor loadings** | | | | | | | | | | | | | | | | | |
| --- | --- | --- | --- | --- | --- | --- | --- | --- | --- | --- | --- | --- | --- | --- | --- | --- | --- |
|  | | | | | | | | | | | | | | **95% Confidence Interval** | | | |
| **Factor** | | **Indicator** | | **Symbol** | | **Estimate** | | **Std. Error** | | **z-value** | | **p** | | **Lower** | | **Upper** | |
| Factor 1 |  | D1Px |  | λ11 |  | 9.737 |  |  |  |  |  |  |  |  |  |  |  |
|  |  | D1Py |  | λ12 |  | 6.760 |  |  |  |  |  |  |  |  |  |  |  |
|  |  | D1Pz |  | λ13 |  | 7.418 |  |  |  |  |  |  |  |  |  |  |  |
|  |  | D2Px |  | λ14 |  | 9.099 |  |  |  |  |  |  |  |  |  |  |  |
|  |  | D2Py |  | λ15 |  | 9.394 |  |  |  |  |  |  |  |  |  |  |  |
|  |  | D2Pz |  | λ16 |  | 10.139 |  |  |  |  |  |  |  |  |  |  |  |
|  |  | D3Px |  | λ17 |  | 5.907 |  |  |  |  |  |  |  |  |  |  |  |
|  |  | D3Py |  | λ18 |  | 5.250 |  |  |  |  |  |  |  |  |  |  |  |
|  |  | D3Pz |  | λ19 |  | 8.229 |  |  |  |  |  |  |  |  |  |  |  |
| Factor 2 |  | C1Px |  | λ21 |  | 226.001 |  |  |  |  |  |  |  |  |  |  |  |
|  |  | C1Py |  | λ22 |  | 231.252 |  |  |  |  |  |  |  |  |  |  |  |
|  |  | C1Pz |  | λ23 |  | 201.426 |  |  |  |  |  |  |  |  |  |  |  |
|  |  | C2Px |  | λ24 |  | 198.899 |  |  |  |  |  |  |  |  |  |  |  |
|  |  | C2Py |  | λ25 |  | 125.591 |  |  |  |  |  |  |  |  |  |  |  |
|  |  | C2Pz |  | λ26 |  | 239.187 |  |  |  |  |  |  |  |  |  |  |  |
|  |  | C3Px |  | λ27 |  | 193.816 |  |  |  |  |  |  |  |  |  |  |  |
|  |  | C3Py |  | λ28 |  | 209.811 |  |  |  |  |  |  |  |  |  |  |  |
|  |  | C3Pz |  | λ29 |  | 208.895 |  |  |  |  |  |  |  |  |  |  |  |
|  | | | | | | | | | | | | | | | | | |

| **Second-order factor loadings** | | | | | | | | | | | | | | | | | |
| --- | --- | --- | --- | --- | --- | --- | --- | --- | --- | --- | --- | --- | --- | --- | --- | --- | --- |
|  | | | | | | | | | | | | | | **95% Confidence Interval** | | | |
| **Factor** | | **Indicator** | | **Symbol** | | **Estimate** | | **Std. Error** | | **z-value** | | **p** | | **Lower** | | **Upper** | |
| SecondOrder |  | Factor 1 |  | γ11 |  | 23.094 |  |  |  |  |  |  |  |  |  |  |  |
|  | | | | | | | | | | | | | | | | | |

| **Factor variances** | | | | | | | | | | | | | |
| --- | --- | --- | --- | --- | --- | --- | --- | --- | --- | --- | --- | --- | --- |
|  | | | | | | | | | | **95% Confidence Interval** | | | |
| **Factor** | | **Estimate** | | **Std. Error** | | **z-value** | | **p** | | **Lower** | | **Upper** | |
| Factor 1 |  | 0.000 |  | 0.000 |  |  |  |  |  | 0.000 |  | 0.000 |  |
| Factor 2 |  | 1.000 |  | 0.000 |  |  |  |  |  | 1.000 |  | 1.000 |  |
| Second-Order |  | 1.000 |  | 0.000 |  |  |  |  |  | 1.000 |  | 1.000 |  |
|  | | | | | | | | | | | | | |

| **Residual variances** | | | | | | | | | | | | | |
| --- | --- | --- | --- | --- | --- | --- | --- | --- | --- | --- | --- | --- | --- |
|  | | | | | | | | | | **95% Confidence Interval** | | | |
| **Indicator** | | **Estimate** | | **Std. Error** | | **z-value** | | **p** | | **Lower** | | **Upper** | |
| D1Px |  | 237671.961 |  |  |  |  |  |  |  |  |  |  |  |
| D1Py |  | 222269.827 |  |  |  |  |  |  |  |  |  |  |  |
| D1Pz |  | 233163.814 |  |  |  |  |  |  |  |  |  |  |  |
| D2Px |  | 206032.660 |  |  |  |  |  |  |  |  |  |  |  |
| D2Py |  | 208746.141 |  |  |  |  |  |  |  |  |  |  |  |
| D2Pz |  | 216795.169 |  |  |  |  |  |  |  |  |  |  |  |
| D3Px |  | 228640.159 |  |  |  |  |  |  |  |  |  |  |  |
| D3Py |  | 232019.547 |  |  |  |  |  |  |  |  |  |  |  |
| D3Pz |  | 199759.488 |  |  |  |  |  |  |  |  |  |  |  |
| C1Px |  | 200701.123 |  |  |  |  |  |  |  |  |  |  |  |
| C1Py |  | 205192.717 |  |  |  |  |  |  |  |  |  |  |  |
| C1Pz |  | 209894.636 |  |  |  |  |  |  |  |  |  |  |  |
| C2Px |  | 214801.864 |  |  |  |  |  |  |  |  |  |  |  |
| C2Py |  | 252397.974 |  |  |  |  |  |  |  |  |  |  |  |
| C2Pz |  | 178183.239 |  |  |  |  |  |  |  |  |  |  |  |
| C3Px |  | 197164.727 |  |  |  |  |  |  |  |  |  |  |  |
| C3Py |  | 209842.589 |  |  |  |  |  |  |  |  |  |  |  |
| C3Pz |  | 200507.735 |  |  |  |  |  |  |  |  |  |  |  |
|  | | | | | | | | | | | | | |

**Table 11.1.4 Adjacent positive D-A**

**Model fit**

| **Chi-square test** | | | | | | | |
| --- | --- | --- | --- | --- | --- | --- | --- |
| **Model** | | **Χ²** | | **df** | | **p** | |
| Baseline model |  | 1529.301 |  | 153 |  |  |  |
| Factor model |  | 575.644 |  | 133 |  | < .001 |  |
|  | | | | | | | |

**Additional fit measures**

| **Fit indices** | | | |
| --- | --- | --- | --- |
| **Index** | | **Value** | |
| Comparative Fit Index (CFI) |  | 0.678 |  |
| Tucker-Lewis Index (TLI) |  | 0.630 |  |
| Bentler-Bonett Non-normed Fit Index (NNFI) |  | 0.630 |  |
| Bentler-Bonett Normed Fit Index (NFI) |  | 0.624 |  |
| Parsimony Normed Fit Index (PNFI) |  | 0.542 |  |
| Bollen's Relative Fit Index (RFI) |  | 0.567 |  |
| Bollen's Incremental Fit Index (IFI) |  | 0.683 |  |
| Relative Noncentrality Index (RNI) |  | 0.678 |  |
|  | | | |

| **Information criteria** | | | |
| --- | --- | --- | --- |
|  | | **Value** | |
| Log-likelihood |  | -140866.237 |  |
| Number of free parameters |  | 38.000 |  |
| Akaike (AIC) |  | 281808.474 |  |
| Bayesian (BIC) |  | 281996.129 |  |
| Sample-size adjusted Bayesian (SSABIC) |  | 281875.437 |  |
|  | | | |

| **Other fit measures** | | | |
| --- | --- | --- | --- |
| **Metric** | | **Value** | |
| Root mean square error of approximation (RMSEA) |  | 0.057 |  |
| RMSEA 90% CI lower bound |  | 0.052 |  |
| RMSEA 90% CI upper bound |  | 0.062 |  |
| RMSEA p-value |  | 0.009 |  |
| Standardized root mean square residual (SRMR) |  | 0.054 |  |
| Hoelter's critical N (α = .05) |  | 289.204 |  |
| Hoelter's critical N (α = .01) |  | 312.379 |  |
| Goodness of fit index (GFI) |  | 0.934 |  |
| McDonald fit index (MFI) |  | 0.807 |  |
| Expected cross validation index (ECVI) |  | 0.632 |  |
|  | | | |

| **R-Squared** | | | |
| --- | --- | --- | --- |
|  | | **R²** | |
| D1Px |  | 0.142 |  |
| D1Py |  | 0.121 |  |
| D1Pz |  | 0.082 |  |
| D2Px |  | 0.147 |  |
| D2Py |  | 0.160 |  |
| D2Pz |  | 0.160 |  |
| D3Px |  | 0.108 |  |
| D3Py |  | 0.122 |  |
| D3Pz |  | 0.188 |  |
| A1Px |  | 0.087 |  |
| A1Py |  | 0.146 |  |
| A1Pz |  | 0.066 |  |
| A2Px |  | 0.086 |  |
| A2Py |  | 0.060 |  |
| A2Pz |  | 0.082 |  |
| A3Px |  | 0.192 |  |
| A3Py |  | 0.091 |  |
| A3Pz |  | 0.149 |  |
| Factor 1 |  | 1.000 |  |
|  | | | |

**Parameter estimates**

| **Factor loadings** | | | | | | | | | | | | | | | | | |
| --- | --- | --- | --- | --- | --- | --- | --- | --- | --- | --- | --- | --- | --- | --- | --- | --- | --- |
|  | | | | | | | | | | | | | | **95% Confidence Interval** | | | |
| **Factor** | | **Indicator** | | **Symbol** | | **Estimate** | | **Std. Error** | | **z-value** | | **p** | | **Lower** | | **Upper** | |
| Factor 1 |  | D1Px |  | λ11 |  | 9.296 |  |  |  |  |  |  |  |  |  |  |  |
|  |  | D1Py |  | λ12 |  | 7.916 |  |  |  |  |  |  |  |  |  |  |  |
|  |  | D1Pz |  | λ13 |  | 6.745 |  |  |  |  |  |  |  |  |  |  |  |
|  |  | D2Px |  | λ14 |  | 8.790 |  |  |  |  |  |  |  |  |  |  |  |
|  |  | D2Py |  | λ15 |  | 9.289 |  |  |  |  |  |  |  |  |  |  |  |
|  |  | D2Pz |  | λ16 |  | 9.554 |  |  |  |  |  |  |  |  |  |  |  |
|  |  | D3Px |  | λ17 |  | 7.516 |  |  |  |  |  |  |  |  |  |  |  |
|  |  | D3Py |  | λ18 |  | 7.948 |  |  |  |  |  |  |  |  |  |  |  |
|  |  | D3Pz |  | λ19 |  | 9.675 |  |  |  |  |  |  |  |  |  |  |  |
| Factor 2 |  | A1Px |  | λ21 |  | 151.086 |  |  |  |  |  |  |  |  |  |  |  |
|  |  | A1Py |  | λ22 |  | 182.545 |  |  |  |  |  |  |  |  |  |  |  |
|  |  | A1Pz |  | λ23 |  | 137.417 |  |  |  |  |  |  |  |  |  |  |  |
|  |  | A2Px |  | λ24 |  | 124.317 |  |  |  |  |  |  |  |  |  |  |  |
|  |  | A2Py |  | λ25 |  | 106.836 |  |  |  |  |  |  |  |  |  |  |  |
|  |  | A2Pz |  | λ26 |  | 147.484 |  |  |  |  |  |  |  |  |  |  |  |
|  |  | A3Px |  | λ27 |  | 202.726 |  |  |  |  |  |  |  |  |  |  |  |
|  |  | A3Py |  | λ28 |  | 144.404 |  |  |  |  |  |  |  |  |  |  |  |
|  |  | A3Pz |  | λ29 |  | 181.129 |  |  |  |  |  |  |  |  |  |  |  |
|  | | | | | | | | | | | | | | | | | |

| **Second-order factor loadings** | | | | | | | | | | | | | | | | | |
| --- | --- | --- | --- | --- | --- | --- | --- | --- | --- | --- | --- | --- | --- | --- | --- | --- | --- |
|  | | | | | | | | | | | | | | **95% Confidence Interval** | | | |
| **Factor** | | **Indicator** | | **Symbol** | | **Estimate** | | **Std. Error** | | **z-value** | | **p** | | **Lower** | | **Upper** | |
| SecondOrder |  | Factor 1 |  | γ11 |  | 21.786 |  |  |  |  |  |  |  |  |  |  |  |
|  | | | | | | | | | | | | | | | | | |

| **Factor variances** | | | | | | | | | | | | | |
| --- | --- | --- | --- | --- | --- | --- | --- | --- | --- | --- | --- | --- | --- |
|  | | | | | | | | | | **95% Confidence Interval** | | | |
| **Factor** | | **Estimate** | | **Std. Error** | | **z-value** | | **p** | | **Lower** | | **Upper** | |
| Factor 1 |  | 0.000 |  | 0.000 |  |  |  |  |  | 0.000 |  | 0.000 |  |
| Factor 2 |  | 1.000 |  | 0.000 |  |  |  |  |  | 1.000 |  | 1.000 |  |
| Second-Order |  | 1.000 |  | 0.000 |  |  |  |  |  | 1.000 |  | 1.000 |  |
|  | | | | | | | | | | | | | |

| **Residual variances** | | | | | | | | | | | | | |
| --- | --- | --- | --- | --- | --- | --- | --- | --- | --- | --- | --- | --- | --- |
|  | | | | | | | | | | **95% Confidence Interval** | | | |
| **Indicator** | | **Estimate** | | **Std. Error** | | **z-value** | | **p** | | **Lower** | | **Upper** | |
| D1Px |  | 247207.264 |  |  |  |  |  |  |  |  |  |  |  |
| D1Py |  | 216906.239 |  |  |  |  |  |  |  |  |  |  |  |
| D1Pz |  | 240914.528 |  |  |  |  |  |  |  |  |  |  |  |
| D2Px |  | 213533.557 |  |  |  |  |  |  |  |  |  |  |  |
| D2Py |  | 214856.084 |  |  |  |  |  |  |  |  |  |  |  |
| D2Pz |  | 228297.912 |  |  |  |  |  |  |  |  |  |  |  |
| D3Px |  | 220437.188 |  |  |  |  |  |  |  |  |  |  |  |
| D3Py |  | 216737.753 |  |  |  |  |  |  |  |  |  |  |  |
| D3Pz |  | 191447.225 |  |  |  |  |  |  |  |  |  |  |  |
| A1Px |  | 239499.380 |  |  |  |  |  |  |  |  |  |  |  |
| A1Py |  | 194329.010 |  |  |  |  |  |  |  |  |  |  |  |
| A1Pz |  | 265590.028 |  |  |  |  |  |  |  |  |  |  |  |
| A2Px |  | 163935.095 |  |  |  |  |  |  |  |  |  |  |  |
| A2Py |  | 179648.428 |  |  |  |  |  |  |  |  |  |  |  |
| A2Pz |  | 244229.757 |  |  |  |  |  |  |  |  |  |  |  |
| A3Px |  | 172783.746 |  |  |  |  |  |  |  |  |  |  |  |
| A3Py |  | 208271.073 |  |  |  |  |  |  |  |  |  |  |  |
| A3Pz |  | 187850.272 |  |  |  |  |  |  |  |  |  |  |  |
|  | | | | | | | | | | | | | |

**Table 11.1.5 Antipodes positive A-C**

**Model fit**

| **Chi-square test** | | | | | | | |
| --- | --- | --- | --- | --- | --- | --- | --- |
| **Model** | | **Χ²** | | **df** | | **p** | |
| Baseline model |  | 1516.417 |  | 153 |  |  |  |
| Factor model |  | 398.411 |  | 133 |  | < .001 |  |
|  | | | | | | | |

**Additional fit measures**

| **Fit indices** | | | |
| --- | --- | --- | --- |
| **Index** | | **Value** | |
| Comparative Fit Index (CFI) |  | 0.805 |  |
| Tucker-Lewis Index (TLI) |  | 0.776 |  |
| Bentler-Bonett Non-normed Fit Index (NNFI) |  | 0.776 |  |
| Bentler-Bonett Normed Fit Index (NFI) |  | 0.737 |  |
| Parsimony Normed Fit Index (PNFI) |  | 0.641 |  |
| Bollen's Relative Fit Index (RFI) |  | 0.698 |  |
| Bollen's Incremental Fit Index (IFI) |  | 0.808 |  |
| Relative Noncentrality Index (RNI) |  | 0.805 |  |
|  | | | |

| **Information criteria** | | | |
| --- | --- | --- | --- |
|  | | **Value** | |
| Log-likelihood |  | -140679.428 |  |
| Number of free parameters |  | 38.000 |  |
| Akaike (AIC) |  | 281434.856 |  |
| Bayesian (BIC) |  | 281622.510 |  |
| Sample-size adjusted Bayesian (SSABIC) |  | 281501.818 |  |
|  | | | |

| **Other fit measures** | | | |
| --- | --- | --- | --- |
| **Metric** | | **Value** | |
| Root mean square error of approximation (RMSEA) |  | 0.044 |  |
| RMSEA 90% CI lower bound |  | 0.039 |  |
| RMSEA 90% CI upper bound |  | 0.049 |  |
| RMSEA p-value |  | 0.976 |  |
| Standardized root mean square residual (SRMR) |  | 0.046 |  |
| Hoelter's critical N (α = .05) |  | 417.412 |  |
| Hoelter's critical N (α = .01) |  | 450.895 |  |
| Goodness of fit index (GFI) |  | 0.954 |  |
| McDonald fit index (MFI) |  | 0.879 |  |
| Expected cross validation index (ECVI) |  | 0.460 |  |
|  | | | |

| **R-Squared** | | | |
| --- | --- | --- | --- |
|  | | **R²** | |
| C1Px |  | 0.210 |  |
| C1Py |  | 0.230 |  |
| C1Pz |  | 0.124 |  |
| C2Px |  | 0.155 |  |
| C2Py |  | 0.047 |  |
| C2Pz |  | 0.211 |  |
| C3Px |  | 0.186 |  |
| C3Py |  | 0.209 |  |
| C3Pz |  | 0.178 |  |
| A1Px |  | 0.088 |  |
| A1Py |  | 0.104 |  |
| A1Pz |  | 0.052 |  |
| A2Px |  | 0.036 |  |
| A2Py |  | 0.019 |  |
| A2Pz |  | 0.045 |  |
| A3Px |  | 0.269 |  |
| A3Py |  | 0.152 |  |
| A3Pz |  | 0.240 |  |
| Factor 1 |  | 1.000 |  |
|  | | | |

**Parameter estimates**

| **Factor loadings** | | | | | | | | | | | | | | | | | |
| --- | --- | --- | --- | --- | --- | --- | --- | --- | --- | --- | --- | --- | --- | --- | --- | --- | --- |
|  | | | | | | | | | | | | | | **95% Confidence Interval** | | | |
| **Factor** | | **Indicator** | | **Symbol** | | **Estimate** | | **Std. Error** | | **z-value** | | **p** | | **Lower** | | **Upper** | |
| Factor 1 |  | C1Px |  | λ11 |  | 9.794 |  |  |  |  |  |  |  |  |  |  |  |
|  |  | C1Py |  | λ12 |  | 10.384 |  |  |  |  |  |  |  |  |  |  |  |
|  |  | C1Pz |  | λ13 |  | 7.515 |  |  |  |  |  |  |  |  |  |  |  |
|  |  | C2Px |  | λ14 |  | 8.439 |  |  |  |  |  |  |  |  |  |  |  |
|  |  | C2Py |  | λ15 |  | 4.790 |  |  |  |  |  |  |  |  |  |  |  |
|  |  | C2Pz |  | λ16 |  | 9.475 |  |  |  |  |  |  |  |  |  |  |  |
|  |  | C3Px |  | λ17 |  | 8.891 |  |  |  |  |  |  |  |  |  |  |  |
|  |  | C3Py |  | λ18 |  | 9.807 |  |  |  |  |  |  |  |  |  |  |  |
|  |  | C3Pz |  | λ19 |  | 8.867 |  |  |  |  |  |  |  |  |  |  |  |
| Factor 2 |  | A1Px |  | λ21 |  | 152.104 |  |  |  |  |  |  |  |  |  |  |  |
|  |  | A1Py |  | λ22 |  | 154.233 |  |  |  |  |  |  |  |  |  |  |  |
|  |  | A1Pz |  | λ23 |  | 122.178 |  |  |  |  |  |  |  |  |  |  |  |
|  |  | A2Px |  | λ24 |  | 80.836 |  |  |  |  |  |  |  |  |  |  |  |
|  |  | A2Py |  | λ25 |  | 60.919 |  |  |  |  |  |  |  |  |  |  |  |
|  |  | A2Pz |  | λ26 |  | 109.813 |  |  |  |  |  |  |  |  |  |  |  |
|  |  | A3Px |  | λ27 |  | 239.969 |  |  |  |  |  |  |  |  |  |  |  |
|  |  | A3Py |  | λ28 |  | 186.664 |  |  |  |  |  |  |  |  |  |  |  |
|  |  | A3Pz |  | λ29 |  | 230.178 |  |  |  |  |  |  |  |  |  |  |  |
|  | | | | | | | | | | | | | | | | | |

| **Second-order factor loadings** | | | | | | | | | | | | | | | | | |
| --- | --- | --- | --- | --- | --- | --- | --- | --- | --- | --- | --- | --- | --- | --- | --- | --- | --- |
|  | | | | | | | | | | | | | | **95% Confidence Interval** | | | |
| **Factor** | | **Indicator** | | **Symbol** | | **Estimate** | | **Std. Error** | | **z-value** | | **p** | | **Lower** | | **Upper** | |
| SecondOrder |  | Factor 1 |  | γ11 |  | 23.495 |  |  |  |  |  |  |  |  |  |  |  |
|  | | | | | | | | | | | | | | | | | |

| **Factor variances** | | | | | | | | | | | | | |
| --- | --- | --- | --- | --- | --- | --- | --- | --- | --- | --- | --- | --- | --- |
|  | | | | | | | | | | **95% Confidence Interval** | | | |
| **Factor** | | **Estimate** | | **Std. Error** | | **z-value** | | **p** | | **Lower** | | **Upper** | |
| Factor 1 |  | 0.000 |  | 0.000 |  |  |  |  |  | 0.000 |  | 0.000 |  |
| Factor 2 |  | 1.000 |  | 0.000 |  |  |  |  |  | 1.000 |  | 1.000 |  |
| Second-Order |  | 1.000 |  | 0.000 |  |  |  |  |  | 1.000 |  | 1.000 |  |
|  | | | | | | | | | | | | | |

| **Residual variances** | | | | | | | | | | | | | |
| --- | --- | --- | --- | --- | --- | --- | --- | --- | --- | --- | --- | --- | --- |
|  | | | | | | | | | | **95% Confidence Interval** | | | |
| **Indicator** | | **Estimate** | | **Std. Error** | | **z-value** | | **p** | | **Lower** | | **Upper** | |
| C1Px |  | 198830.540 |  |  |  |  |  |  |  |  |  |  |  |
| C1Py |  | 199144.468 |  |  |  |  |  |  |  |  |  |  |  |
| C1Pz |  | 219289.868 |  |  |  |  |  |  |  |  |  |  |  |
| C2Px |  | 215059.014 |  |  |  |  |  |  |  |  |  |  |  |
| C2Py |  | 255508.347 |  |  |  |  |  |  |  |  |  |  |  |
| C2Pz |  | 185829.904 |  |  |  |  |  |  |  |  |  |  |  |
| C3Px |  | 191095.216 |  |  |  |  |  |  |  |  |  |  |  |
| C3Py |  | 200771.207 |  |  |  |  |  |  |  |  |  |  |  |
| C3Pz |  | 200750.779 |  |  |  |  |  |  |  |  |  |  |  |
| A1Px |  | 239190.612 |  |  |  |  |  |  |  |  |  |  |  |
| A1Py |  | 203863.586 |  |  |  |  |  |  |  |  |  |  |  |
| A1Pz |  | 269546.156 |  |  |  |  |  |  |  |  |  |  |  |
| A2Px |  | 172854.381 |  |  |  |  |  |  |  |  |  |  |  |
| A2Py |  | 187351.767 |  |  |  |  |  |  |  |  |  |  |  |
| A2Pz |  | 253923.102 |  |  |  |  |  |  |  |  |  |  |  |
| A3Px |  | 156296.348 |  |  |  |  |  |  |  |  |  |  |  |
| A3Py |  | 194279.904 |  |  |  |  |  |  |  |  |  |  |  |
| A3Pz |  | 167675.804 |  |  |  |  |  |  |  |  |  |  |  |
|  | | | | | | | | | | | | | |

**Table 11.1.6 Antipodes positive B-D**

**Model fit**

| **Chi-square test** | | | | | | | |
| --- | --- | --- | --- | --- | --- | --- | --- |
| **Model** | | **Χ²** | | **df** | | **p** | |
| Baseline model |  | 1461.868 |  | 153 |  |  |  |
| Factor model |  | 465.046 |  | 133 |  | < .001 |  |
|  | | | | | | | |

**Additional fit measures**

| **Fit indices** | | | |
| --- | --- | --- | --- |
| **Index** | | **Value** | |
| Comparative Fit Index (CFI) |  | 0.746 |  |
| Tucker-Lewis Index (TLI) |  | 0.708 |  |
| Bentler-Bonett Non-normed Fit Index (NNFI) |  | 0.708 |  |
| Bentler-Bonett Normed Fit Index (NFI) |  | 0.682 |  |
| Parsimony Normed Fit Index (PNFI) |  | 0.593 |  |
| Bollen's Relative Fit Index (RFI) |  | 0.634 |  |
| Bollen's Incremental Fit Index (IFI) |  | 0.750 |  |
| Relative Noncentrality Index (RNI) |  | 0.746 |  |
|  | | | |

| **Information criteria** | | | |
| --- | --- | --- | --- |
|  | | **Value** | |
| Log-likelihood |  | -141279.204 |  |
| Number of free parameters |  | 38.000 |  |
| Akaike (AIC) |  | 282634.408 |  |
| Bayesian (BIC) |  | 282822.063 |  |
| Sample-size adjusted Bayesian (SSABIC) |  | 282701.370 |  |
|  | | | |

| **Other fit measures** | | | |
| --- | --- | --- | --- |
| **Metric** | | **Value** | |
| Root mean square error of approximation (RMSEA) |  | 0.049 |  |
| RMSEA 90% CI lower bound |  | 0.044 |  |
| RMSEA 90% CI upper bound |  | 0.054 |  |
| RMSEA p-value |  | 0.596 |  |
| Standardized root mean square residual (SRMR) |  | 0.047 |  |
| Hoelter's critical N (α = .05) |  | 357.745 |  |
| Hoelter's critical N (α = .01) |  | 386.431 |  |
| Goodness of fit index (GFI) |  | 0.949 |  |
| McDonald fit index (MFI) |  | 0.851 |  |
| Expected cross validation index (ECVI) |  | 0.525 |  |
|  | | | |

| **R-Squared** | | | |
| --- | --- | --- | --- |
|  | | **R²** | |
| B1Px |  | 0.111 |  |
| B1Py |  | 0.101 |  |
| B1Pz |  | 0.095 |  |
| B2Px |  | 0.150 |  |
| B2Py |  | 0.100 |  |
| B2Pz |  | 0.176 |  |
| B3Px |  | 0.070 |  |
| B3Py |  | 0.104 |  |
| B3Pz |  | 0.103 |  |
| D1Px |  | 0.131 |  |
| D1Py |  | 0.114 |  |
| D1Pz |  | 0.087 |  |
| D2Px |  | 0.154 |  |
| D2Py |  | 0.164 |  |
| D2Pz |  | 0.182 |  |
| D3Px |  | 0.114 |  |
| D3Py |  | 0.101 |  |
| D3Pz |  | 0.184 |  |
| Factor 1 |  | 1.000 |  |
|  | | | |

**Parameter estimates**

| **Factor loadings** | | | | | | | | | | | | | | | | | |
| --- | --- | --- | --- | --- | --- | --- | --- | --- | --- | --- | --- | --- | --- | --- | --- | --- | --- |
|  | | | | | | | | | | | | | | **95% Confidence Interval** | | | |
| **Factor** | | **Indicator** | | **Symbol** | | **Estimate** | | **Std. Error** | | **z-value** | | **p** | | **Lower** | | **Upper** | |
| Factor 1 |  | B1Px |  | λ11 |  | 8.422 |  |  |  |  |  |  |  |  |  |  |  |
|  |  | B1Py |  | λ12 |  | 7.538 |  |  |  |  |  |  |  |  |  |  |  |
|  |  | B1Pz |  | λ13 |  | 7.075 |  |  |  |  |  |  |  |  |  |  |  |
|  |  | B2Px |  | λ14 |  | 9.032 |  |  |  |  |  |  |  |  |  |  |  |
|  |  | B2Py |  | λ15 |  | 6.496 |  |  |  |  |  |  |  |  |  |  |  |
|  |  | B2Pz |  | λ16 |  | 9.653 |  |  |  |  |  |  |  |  |  |  |  |
|  |  | B3Px |  | λ17 |  | 6.362 |  |  |  |  |  |  |  |  |  |  |  |
|  |  | B3Py |  | λ18 |  | 7.628 |  |  |  |  |  |  |  |  |  |  |  |
|  |  | B3Pz |  | λ19 |  | 7.829 |  |  |  |  |  |  |  |  |  |  |  |
| Factor 2 |  | D1Px |  | λ21 |  | 194.180 |  |  |  |  |  |  |  |  |  |  |  |
|  |  | D1Py |  | λ22 |  | 167.964 |  |  |  |  |  |  |  |  |  |  |  |
|  |  | D1Pz |  | λ23 |  | 151.336 |  |  |  |  |  |  |  |  |  |  |  |
|  |  | D2Px |  | λ24 |  | 196.427 |  |  |  |  |  |  |  |  |  |  |  |
|  |  | D2Py |  | λ25 |  | 204.957 |  |  |  |  |  |  |  |  |  |  |  |
|  |  | D2Pz |  | λ26 |  | 222.577 |  |  |  |  |  |  |  |  |  |  |  |
|  |  | D3Px |  | λ27 |  | 168.233 |  |  |  |  |  |  |  |  |  |  |  |
|  |  | D3Py |  | λ28 |  | 157.962 |  |  |  |  |  |  |  |  |  |  |  |
|  |  | D3Pz |  | λ29 |  | 208.301 |  |  |  |  |  |  |  |  |  |  |  |
|  | | | | | | | | | | | | | | | | | |

| **Second-order factor loadings** | | | | | | | | | | | | | | | | | |
| --- | --- | --- | --- | --- | --- | --- | --- | --- | --- | --- | --- | --- | --- | --- | --- | --- | --- |
|  | | | | | | | | | | | | | | **95% Confidence Interval** | | | |
| **Factor** | | **Indicator** | | **Symbol** | | **Estimate** | | **Std. Error** | | **z-value** | | **p** | | **Lower** | | **Upper** | |
| SecondOrder |  | Factor 1 |  | γ11 |  | 21.384 |  |  |  |  |  |  |  |  |  |  |  |
|  | | | | | | | | | | | | | | | | | |

| **Factor variances** | | | | | | | | | | | | | |
| --- | --- | --- | --- | --- | --- | --- | --- | --- | --- | --- | --- | --- | --- |
|  | | | | | | | | | | **95% Confidence Interval** | | | |
| **Factor** | | **Estimate** | | **Std. Error** | | **z-value** | | **p** | | **Lower** | | **Upper** | |
| Factor 1 |  | 0.000 |  | 0.000 |  |  |  |  |  | 0.000 |  | 0.000 |  |
| Factor 2 |  | 1.000 |  | 0.000 |  |  |  |  |  | 1.000 |  | 1.000 |  |
| Second-Order |  | 1.000 |  | 0.000 |  |  |  |  |  | 1.000 |  | 1.000 |  |
|  | | | | | | | | | | | | | |

| **Residual variances** | | | | | | | | | | | | | |
| --- | --- | --- | --- | --- | --- | --- | --- | --- | --- | --- | --- | --- | --- |
|  | | | | | | | | | | **95% Confidence Interval** | | | |
| **Indicator** | | **Estimate** | | **Std. Error** | | **z-value** | | **p** | | **Lower** | | **Upper** | |
| B1Px |  | 260633.377 |  |  |  |  |  |  |  |  |  |  |  |
| B1Py |  | 232450.074 |  |  |  |  |  |  |  |  |  |  |  |
| B1Pz |  | 218009.301 |  |  |  |  |  |  |  |  |  |  |  |
| B2Px |  | 210788.572 |  |  |  |  |  |  |  |  |  |  |  |
| B2Py |  | 174623.416 |  |  |  |  |  |  |  |  |  |  |  |
| B2Pz |  | 199450.217 |  |  |  |  |  |  |  |  |  |  |  |
| B3Px |  | 244521.132 |  |  |  |  |  |  |  |  |  |  |  |
| B3Py |  | 229377.565 |  |  |  |  |  |  |  |  |  |  |  |
| B3Pz |  | 243935.372 |  |  |  |  |  |  |  |  |  |  |  |
| D1Px |  | 250523.469 |  |  |  |  |  |  |  |  |  |  |  |
| D1Py |  | 218435.891 |  |  |  |  |  |  |  |  |  |  |  |
| D1Pz |  | 239605.416 |  |  |  |  |  |  |  |  |  |  |  |
| D2Px |  | 211610.655 |  |  |  |  |  |  |  |  |  |  |  |
| D2Py |  | 213804.211 |  |  |  |  |  |  |  |  |  |  |  |
| D2Pz |  | 222087.977 |  |  |  |  |  |  |  |  |  |  |  |
| D3Px |  | 218941.838 |  |  |  |  |  |  |  |  |  |  |  |
| D3Py |  | 221767.687 |  |  |  |  |  |  |  |  |  |  |  |
| D3Pz |  | 192485.135 |  |  |  |  |  |  |  |  |  |  |  |
|  | | | | | | | | | | | | | |
