# Supplementary material for: The structure of human motivation
Source: BMC Psychol. 2023 Oct 6;11:308. doi: 10.1186/s40359-023-01346-5 (PMC10557177; doi:10.1186/s40359-023-01346-5)
Supplement: Supplementary file 6 — Additional file 6: SM Table 11. Confirmatory factor models for adjacent and antipodal life domains: Full output. [file 40359_2023_1346_MOESM6_ESM.zip › Table 11.2 CFA.adjacencies and antipodes.negativeR5.docx]

**Table 11.2**

**Table 11.2.1 Adjacent negative A-B**

**Model fit**

| **Chi-square test** | | | | | | | |
| --- | --- | --- | --- | --- | --- | --- | --- |
| **Model** | | **Χ²** | | **df** | | **p** | |
| Baseline model |  | 2288.674 |  | 153 |  |  |  |
| Factor model |  | 365.692 |  | 133 |  | < .001 |  |
|  | | | | | | | |

**Additional fit measures**

| **Fit indices** | | | |
| --- | --- | --- | --- |
| **Index** | | **Value** | |
| Comparative Fit Index (CFI) |  | 0.891 |  |
| Tucker-Lewis Index (TLI) |  | 0.875 |  |
| Bentler-Bonett Non-normed Fit Index (NNFI) |  | 0.875 |  |
| Bentler-Bonett Normed Fit Index (NFI) |  | 0.840 |  |
| Parsimony Normed Fit Index (PNFI) |  | 0.730 |  |
| Bollen's Relative Fit Index (RFI) |  | 0.816 |  |
| Bollen's Incremental Fit Index (IFI) |  | 0.892 |  |
| Relative Noncentrality Index (RNI) |  | 0.891 |  |
|  | | | |

| **Information criteria** | | | |
| --- | --- | --- | --- |
|  | | **Value** | |
| Log-likelihood |  | -140556.830 |  |
| Number of free parameters |  | 38.000 |  |
| Akaike (AIC) |  | 281189.659 |  |
| Bayesian (BIC) |  | 281377.314 |  |
| Sample-size adjusted Bayesian (SSABIC) |  | 281256.622 |  |
|  | | | |

| **Other fit measures** | | | |
| --- | --- | --- | --- |
| **Metric** | | **Value** | |
| Root mean square error of approximation (RMSEA) |  | 0.041 |  |
| RMSEA 90% CI lower bound |  | 0.036 |  |
| RMSEA 90% CI upper bound |  | 0.046 |  |
| RMSEA p-value |  | 0.998 |  |
| Standardized root mean square residual (SRMR) |  | 0.040 |  |
| Hoelter's critical N (α = .05) |  | 454.669 |  |
| Hoelter's critical N (α = .01) |  | 491.149 |  |
| Goodness of fit index (GFI) |  | 0.960 |  |
| McDonald fit index (MFI) |  | 0.893 |  |
| Expected cross validation index (ECVI) |  | 0.428 |  |
|  | | | |

| **R-Squared** | | | |
| --- | --- | --- | --- |
|  | | **R²** | |
| B1Nx |  | 0.180 |  |
| B1Ny |  | 0.200 |  |
| B1Nz |  | 0.253 |  |
| B2Nx |  | 0.184 |  |
| B2Ny |  | 0.138 |  |
| B2Nz |  | 0.157 |  |
| B3Nx |  | 0.251 |  |
| B3Ny |  | 0.118 |  |
| B3Nz |  | 0.177 |  |
| A1Nx |  | 0.234 |  |
| A1Ny |  | 0.195 |  |
| A1Nz |  | 0.215 |  |
| A2Nx |  | 0.122 |  |
| A2Ny |  | 0.104 |  |
| A2Nz |  | 0.084 |  |
| A3Nx |  | 0.204 |  |
| A3Ny |  | 0.166 |  |
| A3Nz |  | 0.186 |  |
| Factor 1 |  | 1.000 |  |
|  | | | |

**Parameter estimates**

| **Factor loadings** | | | | | | | | | | | | | | | | | |
| --- | --- | --- | --- | --- | --- | --- | --- | --- | --- | --- | --- | --- | --- | --- | --- | --- | --- |
|  | | | | | | | | | | | | | | **95% Confidence Interval** | | | |
| **Factor** | | **Indicator** | | **Symbol** | | **Estimate** | | **Std. Error** | | **z-value** | | **p** | | **Lower** | | **Upper** | |
| Factor 1 |  | B1Nx |  | λ11 |  | 9.107 |  |  |  |  |  |  |  |  |  |  |  |
|  |  | B1Ny |  | λ12 |  | 9.454 |  |  |  |  |  |  |  |  |  |  |  |
|  |  | B1Nz |  | λ13 |  | 10.647 |  |  |  |  |  |  |  |  |  |  |  |
|  |  | B2Nx |  | λ14 |  | 8.898 |  |  |  |  |  |  |  |  |  |  |  |
|  |  | B2Ny |  | λ15 |  | 7.887 |  |  |  |  |  |  |  |  |  |  |  |
|  |  | B2Nz |  | λ16 |  | 8.497 |  |  |  |  |  |  |  |  |  |  |  |
|  |  | B3Nx |  | λ17 |  | 10.571 |  |  |  |  |  |  |  |  |  |  |  |
|  |  | B3Ny |  | λ18 |  | 6.779 |  |  |  |  |  |  |  |  |  |  |  |
|  |  | B3Nz |  | λ19 |  | 8.043 |  |  |  |  |  |  |  |  |  |  |  |
| Factor 2 |  | A1Nx |  | λ21 |  | 246.222 |  |  |  |  |  |  |  |  |  |  |  |
|  |  | A1Ny |  | λ22 |  | 224.030 |  |  |  |  |  |  |  |  |  |  |  |
|  |  | A1Nz |  | λ23 |  | 231.566 |  |  |  |  |  |  |  |  |  |  |  |
|  |  | A2Nx |  | λ24 |  | 158.301 |  |  |  |  |  |  |  |  |  |  |  |
|  |  | A2Ny |  | λ25 |  | 150.491 |  |  |  |  |  |  |  |  |  |  |  |
|  |  | A2Nz |  | λ26 |  | 133.599 |  |  |  |  |  |  |  |  |  |  |  |
|  |  | A3Nx |  | λ27 |  | 240.873 |  |  |  |  |  |  |  |  |  |  |  |
|  |  | A3Ny |  | λ28 |  | 205.610 |  |  |  |  |  |  |  |  |  |  |  |
|  |  | A3Nz |  | λ29 |  | 216.453 |  |  |  |  |  |  |  |  |  |  |  |
|  | | | | | | | | | | | | | | | | | |

| **Second-order factor loadings** | | | | | | | | | | | | | | | | | |
| --- | --- | --- | --- | --- | --- | --- | --- | --- | --- | --- | --- | --- | --- | --- | --- | --- | --- |
|  | | | | | | | | | | | | | | **95% Confidence Interval** | | | |
| **Factor** | | **Indicator** | | **Symbol** | | **Estimate** | | **Std. Error** | | **z-value** | | **p** | | **Lower** | | **Upper** | |
| SecondOrder |  | Factor 1 |  | γ11 |  | 24.063 |  |  |  |  |  |  |  |  |  |  |  |
|  | | | | | | | | | | | | | | | | | |

| **Factor variances** | | | | | | | | | | | | | |
| --- | --- | --- | --- | --- | --- | --- | --- | --- | --- | --- | --- | --- | --- |
|  | | | | | | | | | | **95% Confidence Interval** | | | |
| **Factor** | | **Estimate** | | **Std. Error** | | **z-value** | | **p** | | **Lower** | | **Upper** | |
| Factor 1 |  | 0.000 |  | 0.000 |  |  |  |  |  | 0.000 |  | 0.000 |  |
| Factor 2 |  | 1.000 |  | 0.000 |  |  |  |  |  | 1.000 |  | 1.000 |  |
| Second-Order |  | 1.000 |  | 0.000 |  |  |  |  |  | 1.000 |  | 1.000 |  |
|  | | | | | | | | | | | | | |

| **Residual variances** | | | | | | | | | | | | | |
| --- | --- | --- | --- | --- | --- | --- | --- | --- | --- | --- | --- | --- | --- |
|  | | | | | | | | | | **95% Confidence Interval** | | | |
| **Indicator** | | **Estimate** | | **Std. Error** | | **z-value** | | **p** | | **Lower** | | **Upper** | |
| B1Nx |  | 218599.167 |  |  |  |  |  |  |  |  |  |  |  |
| B1Ny |  | 207465.211 |  |  |  |  |  |  |  |  |  |  |  |
| B1Nz |  | 193380.067 |  |  |  |  |  |  |  |  |  |  |  |
| B2Nx |  | 203835.411 |  |  |  |  |  |  |  |  |  |  |  |
| B2Ny |  | 225421.486 |  |  |  |  |  |  |  |  |  |  |  |
| B2Nz |  | 225131.430 |  |  |  |  |  |  |  |  |  |  |  |
| B3Nx |  | 193504.638 |  |  |  |  |  |  |  |  |  |  |  |
| B3Ny |  | 198044.053 |  |  |  |  |  |  |  |  |  |  |  |
| B3Nz |  | 173743.782 |  |  |  |  |  |  |  |  |  |  |  |
| A1Nx |  | 198914.170 |  |  |  |  |  |  |  |  |  |  |  |
| A1Ny |  | 206799.412 |  |  |  |  |  |  |  |  |  |  |  |
| A1Nz |  | 195770.977 |  |  |  |  |  |  |  |  |  |  |  |
| A2Nx |  | 179793.629 |  |  |  |  |  |  |  |  |  |  |  |
| A2Ny |  | 194271.827 |  |  |  |  |  |  |  |  |  |  |  |
| A2Nz |  | 195493.272 |  |  |  |  |  |  |  |  |  |  |  |
| A3Nx |  | 226284.853 |  |  |  |  |  |  |  |  |  |  |  |
| A3Ny |  | 211846.878 |  |  |  |  |  |  |  |  |  |  |  |
| A3Nz |  | 205409.975 |  |  |  |  |  |  |  |  |  |  |  |
|  | | | | | | | | | | | | | |

**Table 11.2.2 Adjacent negative B-C**

**Model fit**

| **Chi-square test** | | | | | | | |
| --- | --- | --- | --- | --- | --- | --- | --- |
| **Model** | | **Χ²** | | **df** | | **p** | |
| Baseline model |  | 2699.460 |  | 153 |  |  |  |
| Factor model |  | 370.828 |  | 133 |  | < .001 |  |
|  | | | | | | | |

**Additional fit measures**

| **Fit indices** | | | |
| --- | --- | --- | --- |
| **Index** | | **Value** | |
| Comparative Fit Index (CFI) |  | 0.907 |  |
| Tucker-Lewis Index (TLI) |  | 0.893 |  |
| Bentler-Bonett Non-normed Fit Index (NNFI) |  | 0.893 |  |
| Bentler-Bonett Normed Fit Index (NFI) |  | 0.863 |  |
| Parsimony Normed Fit Index (PNFI) |  | 0.750 |  |
| Bollen's Relative Fit Index (RFI) |  | 0.842 |  |
| Bollen's Incremental Fit Index (IFI) |  | 0.907 |  |
| Relative Noncentrality Index (RNI) |  | 0.907 |  |
|  | | | |

| **Information criteria** | | | |
| --- | --- | --- | --- |
|  | | **Value** | |
| Log-likelihood |  | -140273.914 |  |
| Number of free parameters |  | 38.000 |  |
| Akaike (AIC) |  | 280623.829 |  |
| Bayesian (BIC) |  | 280811.484 |  |
| Sample-size adjusted Bayesian (SSABIC) |  | 280690.791 |  |
|  | | | |

| **Other fit measures** | | | |
| --- | --- | --- | --- |
| **Metric** | | **Value** | |
| Root mean square error of approximation (RMSEA) |  | 0.042 |  |
| RMSEA 90% CI lower bound |  | 0.037 |  |
| RMSEA 90% CI upper bound |  | 0.047 |  |
| RMSEA p-value |  | 0.997 |  |
| Standardized root mean square residual (SRMR) |  | 0.039 |  |
| Hoelter's critical N (α = .05) |  | 448.385 |  |
| Hoelter's critical N (α = .01) |  | 484.359 |  |
| Goodness of fit index (GFI) |  | 0.959 |  |
| McDonald fit index (MFI) |  | 0.891 |  |
| Expected cross validation index (ECVI) |  | 0.433 |  |
|  | | | |

| **R-Squared** | | | |
| --- | --- | --- | --- |
|  | | **R²** | |
| B1Nx |  | 0.156 |  |
| B1Ny |  | 0.194 |  |
| B1Nz |  | 0.261 |  |
| B2Nx |  | 0.185 |  |
| B2Ny |  | 0.155 |  |
| B2Nz |  | 0.154 |  |
| B3Nx |  | 0.250 |  |
| B3Ny |  | 0.124 |  |
| B3Nz |  | 0.178 |  |
| C1Nx |  | 0.243 |  |
| C1Ny |  | 0.227 |  |
| C1Nz |  | 0.170 |  |
| C2Nx |  | 0.275 |  |
| C2Ny |  | 0.175 |  |
| C2Nz |  | 0.151 |  |
| C3Nx |  | 0.273 |  |
| C3Ny |  | 0.334 |  |
| C3Nz |  | 0.304 |  |
| Factor 1 |  | 0.811 |  |
| Factor 2 |  | 0.821 |  |
|  | | | |

**Parameter estimates**

| **Factor loadings** | | | | | | | | | | | | | | | | | |
| --- | --- | --- | --- | --- | --- | --- | --- | --- | --- | --- | --- | --- | --- | --- | --- | --- | --- |
|  | | | | | | | | | | | | | | **95% Confidence Interval** | | | |
| **Factor** | | **Indicator** | | **Symbol** | | **Estimate** | | **Std. Error** | | **z-value** | | **p** | | **Lower** | | **Upper** | |
| Factor 1 |  | B1Nx |  | λ11 |  | 88.703 |  |  |  |  |  |  |  |  |  |  |  |
|  |  | B1Ny |  | λ12 |  | 97.304 |  |  |  |  |  |  |  |  |  |  |  |
|  |  | B1Nz |  | λ13 |  | 113.026 |  |  |  |  |  |  |  |  |  |  |  |
|  |  | B2Nx |  | λ14 |  | 93.414 |  |  |  |  |  |  |  |  |  |  |  |
|  |  | B2Ny |  | λ15 |  | 87.497 |  |  |  |  |  |  |  |  |  |  |  |
|  |  | B2Nz |  | λ16 |  | 88.194 |  |  |  |  |  |  |  |  |  |  |  |
|  |  | B3Nx |  | λ17 |  | 110.357 |  |  |  |  |  |  |  |  |  |  |  |
|  |  | B3Ny |  | λ18 |  | 72.535 |  |  |  |  |  |  |  |  |  |  |  |
|  |  | B3Nz |  | λ19 |  | 84.240 |  |  |  |  |  |  |  |  |  |  |  |
| Factor 2 |  | C1Nx |  | λ21 |  | 102.940 |  |  |  |  |  |  |  |  |  |  |  |
|  |  | C1Ny |  | λ22 |  | 101.185 |  |  |  |  |  |  |  |  |  |  |  |
|  |  | C1Nz |  | λ23 |  | 83.461 |  |  |  |  |  |  |  |  |  |  |  |
|  |  | C2Nx |  | λ24 |  | 110.595 |  |  |  |  |  |  |  |  |  |  |  |
|  |  | C2Ny |  | λ25 |  | 90.298 |  |  |  |  |  |  |  |  |  |  |  |
|  |  | C2Nz |  | λ26 |  | 79.470 |  |  |  |  |  |  |  |  |  |  |  |
|  |  | C3Nx |  | λ27 |  | 107.018 |  |  |  |  |  |  |  |  |  |  |  |
|  |  | C3Ny |  | λ28 |  | 117.347 |  |  |  |  |  |  |  |  |  |  |  |
|  |  | C3Nz |  | λ29 |  | 109.738 |  |  |  |  |  |  |  |  |  |  |  |
|  | | | | | | | | | | | | | | | | | |

| **Second-order factor loadings** | | | | | | | | | | | | | | | | | |
| --- | --- | --- | --- | --- | --- | --- | --- | --- | --- | --- | --- | --- | --- | --- | --- | --- | --- |
|  | | | | | | | | | | | | | | **95% Confidence Interval** | | | |
| **Factor** | | **Indicator** | | **Symbol** | | **Estimate** | | **Std. Error** | | **z-value** | | **p** | | **Lower** | | **Upper** | |
| SecondOrder |  | Factor 1 |  | γ11 |  | 2.073 |  |  |  |  |  |  |  |  |  |  |  |
|  |  | Factor 2 |  | γ12 |  | 2.138 |  |  |  |  |  |  |  |  |  |  |  |
|  | | | | | | | | | | | | | | | | | |

| **Factor variances** | | | | | | | | | | | | | |
| --- | --- | --- | --- | --- | --- | --- | --- | --- | --- | --- | --- | --- | --- |
|  | | | | | | | | | | **95% Confidence Interval** | | | |
| **Factor** | | **Estimate** | | **Std. Error** | | **z-value** | | **p** | | **Lower** | | **Upper** | |
| Factor 1 |  | 1.000 |  | 0.000 |  |  |  |  |  | 1.000 |  | 1.000 |  |
| Factor 2 |  | 1.000 |  | 0.000 |  |  |  |  |  | 1.000 |  | 1.000 |  |
| Second-Order |  | 1.000 |  | 0.000 |  |  |  |  |  | 1.000 |  | 1.000 |  |
|  | | | | | | | | | | | | | |

| **Residual variances** | | | | | | | | | | | | | |
| --- | --- | --- | --- | --- | --- | --- | --- | --- | --- | --- | --- | --- | --- |
|  | | | | | | | | | | **95% Confidence Interval** | | | |
| **Indicator** | | **Estimate** | | **Std. Error** | | **z-value** | | **p** | | **Lower** | | **Upper** | |
| B1Nx |  | 224932.930 |  |  |  |  |  |  |  |  |  |  |  |
| B1Ny |  | 209047.748 |  |  |  |  |  |  |  |  |  |  |  |
| B1Nz |  | 191320.295 |  |  |  |  |  |  |  |  |  |  |  |
| B2Nx |  | 203435.898 |  |  |  |  |  |  |  |  |  |  |  |
| B2Ny |  | 220873.069 |  |  |  |  |  |  |  |  |  |  |  |
| B2Nz |  | 225723.380 |  |  |  |  |  |  |  |  |  |  |  |
| B3Nx |  | 193673.586 |  |  |  |  |  |  |  |  |  |  |  |
| B3Ny |  | 196776.908 |  |  |  |  |  |  |  |  |  |  |  |
| B3Nz |  | 173593.150 |  |  |  |  |  |  |  |  |  |  |  |
| C1Nx |  | 183515.562 |  |  |  |  |  |  |  |  |  |  |  |
| C1Ny |  | 194427.335 |  |  |  |  |  |  |  |  |  |  |  |
| C1Nz |  | 190122.903 |  |  |  |  |  |  |  |  |  |  |  |
| C2Nx |  | 179606.439 |  |  |  |  |  |  |  |  |  |  |  |
| C2Ny |  | 213608.261 |  |  |  |  |  |  |  |  |  |  |  |
| C2Nz |  | 197083.100 |  |  |  |  |  |  |  |  |  |  |  |
| C3Nx |  | 169695.336 |  |  |  |  |  |  |  |  |  |  |  |
| C3Ny |  | 152691.907 |  |  |  |  |  |  |  |  |  |  |  |
| C3Nz |  | 153578.523 |  |  |  |  |  |  |  |  |  |  |  |
|  | | | | | | | | | | | | | |

**Table 11.2.3 Adjacent negative C-D**

**Model fit**

| **Chi-square test** | | | | | | | |
| --- | --- | --- | --- | --- | --- | --- | --- |
| **Model** | | **Χ²** | | **df** | | **p** | |
| Baseline model |  | 2832.212 |  | 153 |  |  |  |
| Factor model |  | 470.785 |  | 133 |  | < .001 |  |
|  | | | | | | | |

**Additional fit measures**

| **Fit indices** | | | |
| --- | --- | --- | --- |
| **Index** | | **Value** | |
| Comparative Fit Index (CFI) |  | 0.874 |  |
| Tucker-Lewis Index (TLI) |  | 0.855 |  |
| Bentler-Bonett Non-normed Fit Index (NNFI) |  | 0.855 |  |
| Bentler-Bonett Normed Fit Index (NFI) |  | 0.834 |  |
| Parsimony Normed Fit Index (PNFI) |  | 0.725 |  |
| Bollen's Relative Fit Index (RFI) |  | 0.809 |  |
| Bollen's Incremental Fit Index (IFI) |  | 0.875 |  |
| Relative Noncentrality Index (RNI) |  | 0.874 |  |
|  | | | |

| **Information criteria** | | | |
| --- | --- | --- | --- |
|  | | **Value** | |
| Log-likelihood |  | -140423.097 |  |
| Number of free parameters |  | 38.000 |  |
| Akaike (AIC) |  | 280922.194 |  |
| Bayesian (BIC) |  | 281109.849 |  |
| Sample-size adjusted Bayesian (SSABIC) |  | 280989.157 |  |
|  | | | |

| **Other fit measures** | | | |
| --- | --- | --- | --- |
| **Metric** | | **Value** | |
| Root mean square error of approximation (RMSEA) |  | 0.050 |  |
| RMSEA 90% CI lower bound |  | 0.045 |  |
| RMSEA 90% CI upper bound |  | 0.055 |  |
| RMSEA p-value |  | 0.540 |  |
| Standardized root mean square residual (SRMR) |  | 0.046 |  |
| Hoelter's critical N (α = .05) |  | 353.397 |  |
| Hoelter's critical N (α = .01) |  | 381.733 |  |
| Goodness of fit index (GFI) |  | 0.946 |  |
| McDonald fit index (MFI) |  | 0.849 |  |
| Expected cross validation index (ECVI) |  | 0.530 |  |
|  | | | |

| **R-Squared** | | | |
| --- | --- | --- | --- |
|  | | **R²** | |
| D3Nx |  | 0.218 |  |
| D3Ny |  | 0.107 |  |
| D3Nz |  | 0.152 |  |
| D1Nx |  | 0.199 |  |
| D1Ny |  | 0.210 |  |
| D1Nz |  | 0.210 |  |
| D2Nx |  | 0.269 |  |
| D2Ny |  | 0.243 |  |
| D2Nz |  | 0.167 |  |
| C3Nx |  | 0.265 |  |
| C3Ny |  | 0.308 |  |
| C3Nz |  | 0.299 |  |
| C1Nx |  | 0.269 |  |
| C1Ny |  | 0.240 |  |
| C1Nz |  | 0.181 |  |
| C2Nx |  | 0.261 |  |
| C2Ny |  | 0.176 |  |
| C2Nz |  | 0.154 |  |
| Factor 1 |  | 0.762 |  |
| Factor 2 |  | 0.759 |  |
|  | | | |

**Parameter estimates**

| **Factor loadings** | | | | | | | | | | | | | | | | | |
| --- | --- | --- | --- | --- | --- | --- | --- | --- | --- | --- | --- | --- | --- | --- | --- | --- | --- |
|  | | | | | | | | | | | | | | **95% Confidence Interval** | | | |
| **Factor** | | **Indicator** | | **Symbol** | | **Estimate** | | **Std. Error** | | **z-value** | | **p** | | **Lower** | | **Upper** | |
| Factor 1 |  | D3Nx |  | λ11 |  | 119.791 |  |  |  |  |  |  |  |  |  |  |  |
|  |  | D3Ny |  | λ12 |  | 85.893 |  |  |  |  |  |  |  |  |  |  |  |
|  |  | D3Nz |  | λ13 |  | 96.650 |  |  |  |  |  |  |  |  |  |  |  |
|  |  | D1Nx |  | λ14 |  | 110.860 |  |  |  |  |  |  |  |  |  |  |  |
|  |  | D1Ny |  | λ15 |  | 117.879 |  |  |  |  |  |  |  |  |  |  |  |
|  |  | D1Nz |  | λ16 |  | 112.295 |  |  |  |  |  |  |  |  |  |  |  |
|  |  | D2Nx |  | λ17 |  | 122.617 |  |  |  |  |  |  |  |  |  |  |  |
|  |  | D2Ny |  | λ18 |  | 121.617 |  |  |  |  |  |  |  |  |  |  |  |
|  |  | D2Nz |  | λ19 |  | 96.975 |  |  |  |  |  |  |  |  |  |  |  |
| Factor 2 |  | C3Nx |  | λ21 |  | 122.129 |  |  |  |  |  |  |  |  |  |  |  |
|  |  | C3Ny |  | λ22 |  | 130.535 |  |  |  |  |  |  |  |  |  |  |  |
|  |  | C3Nz |  | λ23 |  | 126.091 |  |  |  |  |  |  |  |  |  |  |  |
|  |  | C1Nx |  | λ24 |  | 125.436 |  |  |  |  |  |  |  |  |  |  |  |
|  |  | C1Ny |  | λ25 |  | 120.760 |  |  |  |  |  |  |  |  |  |  |  |
|  |  | C1Nz |  | λ26 |  | 100.036 |  |  |  |  |  |  |  |  |  |  |  |
|  |  | C2Nx |  | λ27 |  | 124.838 |  |  |  |  |  |  |  |  |  |  |  |
|  |  | C2Ny |  | λ28 |  | 104.711 |  |  |  |  |  |  |  |  |  |  |  |
|  |  | C2Nz |  | λ29 |  | 92.847 |  |  |  |  |  |  |  |  |  |  |  |
|  | | | | | | | | | | | | | | | | | |

| **Second-order factor loadings** | | | | | | | | | | | | | | | | | |
| --- | --- | --- | --- | --- | --- | --- | --- | --- | --- | --- | --- | --- | --- | --- | --- | --- | --- |
|  | | | | | | | | | | | | | | **95% Confidence Interval** | | | |
| **Factor** | | **Indicator** | | **Symbol** | | **Estimate** | | **Std. Error** | | **z-value** | | **p** | | **Lower** | | **Upper** | |
| SecondOrder |  | Factor 1 |  | γ11 |  | 1.787 |  |  |  |  |  |  |  |  |  |  |  |
|  |  | Factor 2 |  | γ12 |  | 1.774 |  |  |  |  |  |  |  |  |  |  |  |
|  | | | | | | | | | | | | | | | | | |

| **Factor variances** | | | | | | | | | | | | | |
| --- | --- | --- | --- | --- | --- | --- | --- | --- | --- | --- | --- | --- | --- |
|  | | | | | | | | | | **95% Confidence Interval** | | | |
| **Factor** | | **Estimate** | | **Std. Error** | | **z-value** | | **p** | | **Lower** | | **Upper** | |
| Factor 1 |  | 1.000 |  | 0.000 |  |  |  |  |  | 1.000 |  | 1.000 |  |
| Factor 2 |  | 1.000 |  | 0.000 |  |  |  |  |  | 1.000 |  | 1.000 |  |
| Second-Order |  | 1.000 |  | 0.000 |  |  |  |  |  | 1.000 |  | 1.000 |  |
|  | | | | | | | | | | | | | |

| **Residual variances** | | | | | | | | | | | | | |
| --- | --- | --- | --- | --- | --- | --- | --- | --- | --- | --- | --- | --- | --- |
|  | | | | | | | | | | **95% Confidence Interval** | | | |
| **Indicator** | | **Estimate** | | **Std. Error** | | **z-value** | | **p** | | **Lower** | | **Upper** | |
| D3Nx |  | 215905.946 |  |  |  |  |  |  |  |  |  |  |  |
| D3Ny |  | 258148.870 |  |  |  |  |  |  |  |  |  |  |  |
| D3Nz |  | 218384.399 |  |  |  |  |  |  |  |  |  |  |  |
| D1Nx |  | 207808.638 |  |  |  |  |  |  |  |  |  |  |  |
| D1Ny |  | 219126.622 |  |  |  |  |  |  |  |  |  |  |  |
| D1Nz |  | 199488.560 |  |  |  |  |  |  |  |  |  |  |  |
| D2Nx |  | 171331.374 |  |  |  |  |  |  |  |  |  |  |  |
| D2Ny |  | 193183.689 |  |  |  |  |  |  |  |  |  |  |  |
| D2Nz |  | 196582.339 |  |  |  |  |  |  |  |  |  |  |  |
| C3Nx |  | 171649.089 |  |  |  |  |  |  |  |  |  |  |  |
| C3Ny |  | 158749.762 |  |  |  |  |  |  |  |  |  |  |  |
| C3Nz |  | 154737.952 |  |  |  |  |  |  |  |  |  |  |  |
| C1Nx |  | 177304.348 |  |  |  |  |  |  |  |  |  |  |  |
| C1Ny |  | 190994.228 |  |  |  |  |  |  |  |  |  |  |  |
| C1Nz |  | 187431.534 |  |  |  |  |  |  |  |  |  |  |  |
| C2Nx |  | 183122.472 |  |  |  |  |  |  |  |  |  |  |  |
| C2Ny |  | 213566.777 |  |  |  |  |  |  |  |  |  |  |  |
| C2Nz |  | 196519.210 |  |  |  |  |  |  |  |  |  |  |  |
|  | | | | | | | | | | | | | |

**Table 11.2.4 Adjacent negative D-A**

**Model fit**

| **Chi-square test** | | | | | | | |
| --- | --- | --- | --- | --- | --- | --- | --- |
| **Model** | | **Χ²** | | **df** | | **p** | |
| Baseline model |  | 2366.706 |  | 153 |  |  |  |
| Factor model |  | 407.218 |  | 133 |  | < .001 |  |
|  | | | | | | | |

**Additional fit measures**

| **Fit indices** | | | |
| --- | --- | --- | --- |
| **Index** | | **Value** | |
| Comparative Fit Index (CFI) |  | 0.876 |  |
| Tucker-Lewis Index (TLI) |  | 0.857 |  |
| Bentler-Bonett Non-normed Fit Index (NNFI) |  | 0.857 |  |
| Bentler-Bonett Normed Fit Index (NFI) |  | 0.828 |  |
| Parsimony Normed Fit Index (PNFI) |  | 0.720 |  |
| Bollen's Relative Fit Index (RFI) |  | 0.802 |  |
| Bollen's Incremental Fit Index (IFI) |  | 0.877 |  |
| Relative Noncentrality Index (RNI) |  | 0.876 |  |
|  | | | |

| **Information criteria** | | | |
| --- | --- | --- | --- |
|  | | **Value** | |
| Log-likelihood |  | -140704.157 |  |
| Number of free parameters |  | 38.000 |  |
| Akaike (AIC) |  | 281484.314 |  |
| Bayesian (BIC) |  | 281671.969 |  |
| Sample-size adjusted Bayesian (SSABIC) |  | 281551.277 |  |
|  | | | |

| **Other fit measures** | | | |
| --- | --- | --- | --- |
| **Metric** | | **Value** | |
| Root mean square error of approximation (RMSEA) |  | 0.045 |  |
| RMSEA 90% CI lower bound |  | 0.040 |  |
| RMSEA 90% CI upper bound |  | 0.050 |  |
| RMSEA p-value |  | 0.959 |  |
| Standardized root mean square residual (SRMR) |  | 0.041 |  |
| Hoelter's critical N (α = .05) |  | 408.406 |  |
| Hoelter's critical N (α = .01) |  | 441.166 |  |
| Goodness of fit index (GFI) |  | 0.956 |  |
| McDonald fit index (MFI) |  | 0.875 |  |
| Expected cross validation index (ECVI) |  | 0.469 |  |
|  | | | |

| **R-Squared** | | | |
| --- | --- | --- | --- |
|  | | **R²** | |
| D1Nx |  | 0.184 |  |
| D1Ny |  | 0.198 |  |
| D1Nz |  | 0.180 |  |
| D2Nx |  | 0.257 |  |
| D2Ny |  | 0.258 |  |
| D2Nz |  | 0.154 |  |
| D3Nx |  | 0.251 |  |
| D3Ny |  | 0.131 |  |
| D3Nz |  | 0.169 |  |
| A1Nx |  | 0.200 |  |
| A1Ny |  | 0.204 |  |
| A1Nz |  | 0.245 |  |
| A2Nx |  | 0.121 |  |
| A2Ny |  | 0.097 |  |
| A2Nz |  | 0.099 |  |
| A3Nx |  | 0.222 |  |
| A3Ny |  | 0.125 |  |
| A3Nz |  | 0.198 |  |
| Factor 1 |  | 1.000 |  |
|  | | | |

**Parameter estimates**

| **Factor loadings** | | | | | | | | | | | | | | | | | |
| --- | --- | --- | --- | --- | --- | --- | --- | --- | --- | --- | --- | --- | --- | --- | --- | --- | --- |
|  | | | | | | | | | | | | | | **95% Confidence Interval** | | | |
| **Factor** | | **Indicator** | | **Symbol** | | **Estimate** | | **Std. Error** | | **z-value** | | **p** | | **Lower** | | **Upper** | |
| Factor 1 |  | D1Nx |  | λ11 |  | 8.778 |  |  |  |  |  |  |  |  |  |  |  |
|  |  | D1Ny |  | λ12 |  | 9.435 |  |  |  |  |  |  |  |  |  |  |  |
|  |  | D1Nz |  | λ13 |  | 8.569 |  |  |  |  |  |  |  |  |  |  |  |
|  |  | D2Nx |  | λ14 |  | 9.866 |  |  |  |  |  |  |  |  |  |  |  |
|  |  | D2Ny |  | λ15 |  | 10.332 |  |  |  |  |  |  |  |  |  |  |  |
|  |  | D2Nz |  | λ16 |  | 7.661 |  |  |  |  |  |  |  |  |  |  |  |
|  |  | D3Nx |  | λ17 |  | 10.593 |  |  |  |  |  |  |  |  |  |  |  |
|  |  | D3Ny |  | λ18 |  | 7.828 |  |  |  |  |  |  |  |  |  |  |  |
|  |  | D3Nz |  | λ19 |  | 8.396 |  |  |  |  |  |  |  |  |  |  |  |
| Factor 2 |  | A1Nx |  | λ21 |  | 228.082 |  |  |  |  |  |  |  |  |  |  |  |
|  |  | A1Ny |  | λ22 |  | 229.243 |  |  |  |  |  |  |  |  |  |  |  |
|  |  | A1Nz |  | λ23 |  | 246.999 |  |  |  |  |  |  |  |  |  |  |  |
|  |  | A2Nx |  | λ24 |  | 157.191 |  |  |  |  |  |  |  |  |  |  |  |
|  |  | A2Ny |  | λ25 |  | 144.761 |  |  |  |  |  |  |  |  |  |  |  |
|  |  | A2Nz |  | λ26 |  | 145.250 |  |  |  |  |  |  |  |  |  |  |  |
|  |  | A3Nx |  | λ27 |  | 251.157 |  |  |  |  |  |  |  |  |  |  |  |
|  |  | A3Ny |  | λ28 |  | 177.892 |  |  |  |  |  |  |  |  |  |  |  |
|  |  | A3Nz |  | λ29 |  | 223.513 |  |  |  |  |  |  |  |  |  |  |  |
|  | | | | | | | | | | | | | | | | | |

| **Second-order factor loadings** | | | | | | | | | | | | | | | | | |
| --- | --- | --- | --- | --- | --- | --- | --- | --- | --- | --- | --- | --- | --- | --- | --- | --- | --- |
|  | | | | | | | | | | | | | | **95% Confidence Interval** | | | |
| **Factor** | | **Indicator** | | **Symbol** | | **Estimate** | | **Std. Error** | | **z-value** | | **p** | | **Lower** | | **Upper** | |
| SecondOrder |  | Factor 1 |  | γ11 |  | 24.856 |  |  |  |  |  |  |  |  |  |  |  |
|  | | | | | | | | | | | | | | | | | |

| **Factor variances** | | | | | | | | | | | | | |
| --- | --- | --- | --- | --- | --- | --- | --- | --- | --- | --- | --- | --- | --- |
|  | | | | | | | | | | **95% Confidence Interval** | | | |
| **Factor** | | **Estimate** | | **Std. Error** | | **z-value** | | **p** | | **Lower** | | **Upper** | |
| Factor 1 |  | 0.000 |  | 0.000 |  |  |  |  |  | 0.000 |  | 0.000 |  |
| Factor 2 |  | 1.000 |  | 0.000 |  |  |  |  |  | 1.000 |  | 1.000 |  |
| Second-Order |  | 1.000 |  | 0.000 |  |  |  |  |  | 1.000 |  | 1.000 |  |
|  | | | | | | | | | | | | | |

| **Residual variances** | | | | | | | | | | | | | |
| --- | --- | --- | --- | --- | --- | --- | --- | --- | --- | --- | --- | --- | --- |
|  | | | | | | | | | | **95% Confidence Interval** | | | |
| **Indicator** | | **Estimate** | | **Std. Error** | | **z-value** | | **p** | | **Lower** | | **Upper** | |
| D1Nx |  | 211738.361 |  |  |  |  |  |  |  |  |  |  |  |
| D1Ny |  | 222395.380 |  |  |  |  |  |  |  |  |  |  |  |
| D1Nz |  | 207006.537 |  |  |  |  |  |  |  |  |  |  |  |
| D2Nx |  | 174242.515 |  |  |  |  |  |  |  |  |  |  |  |
| D2Ny |  | 189256.137 |  |  |  |  |  |  |  |  |  |  |  |
| D2Nz |  | 199756.953 |  |  |  |  |  |  |  |  |  |  |  |
| D3Nx |  | 206760.618 |  |  |  |  |  |  |  |  |  |  |  |
| D3Ny |  | 251227.764 |  |  |  |  |  |  |  |  |  |  |  |
| D3Nz |  | 214006.612 |  |  |  |  |  |  |  |  |  |  |  |
| A1Nx |  | 207517.798 |  |  |  |  |  |  |  |  |  |  |  |
| A1Ny |  | 204436.926 |  |  |  |  |  |  |  |  |  |  |  |
| A1Nz |  | 188385.209 |  |  |  |  |  |  |  |  |  |  |  |
| A2Nx |  | 180143.869 |  |  |  |  |  |  |  |  |  |  |  |
| A2Ny |  | 195963.510 |  |  |  |  |  |  |  |  |  |  |  |
| A2Nz |  | 192244.262 |  |  |  |  |  |  |  |  |  |  |  |
| A3Nx |  | 221225.228 |  |  |  |  |  |  |  |  |  |  |  |
| A3Ny |  | 222476.307 |  |  |  |  |  |  |  |  |  |  |  |
| A3Nz |  | 202303.392 |  |  |  |  |  |  |  |  |  |  |  |
|  | | | | | | | | | | | | | |

**Table 11.2.5 Antipodes negative A-C**

**Model fit**

| **Chi-square test** | | | | | | | |
| --- | --- | --- | --- | --- | --- | --- | --- |
| **Model** | | **Χ²** | | **df** | | **p** | |
| Baseline model |  | 2757.844 |  | 153 |  |  |  |
| Factor model |  | 467.834 |  | 133 |  | < .001 |  |
|  | | | | | | | |

**Additional fit measures**

| **Fit indices** | | | |
| --- | --- | --- | --- |
| **Index** | | **Value** | |
| Comparative Fit Index (CFI) |  | 0.871 |  |
| Tucker-Lewis Index (TLI) |  | 0.852 |  |
| Bentler-Bonett Non-normed Fit Index (NNFI) |  | 0.852 |  |
| Bentler-Bonett Normed Fit Index (NFI) |  | 0.830 |  |
| Parsimony Normed Fit Index (PNFI) |  | 0.722 |  |
| Bollen's Relative Fit Index (RFI) |  | 0.805 |  |
| Bollen's Incremental Fit Index (IFI) |  | 0.872 |  |
| Relative Noncentrality Index (RNI) |  | 0.871 |  |
|  | | | |

| **Information criteria** | | | |
| --- | --- | --- | --- |
|  | | **Value** | |
| Log-likelihood |  | -140146.353 |  |
| Number of free parameters |  | 38.000 |  |
| Akaike (AIC) |  | 280368.706 |  |
| Bayesian (BIC) |  | 280556.360 |  |
| Sample-size adjusted Bayesian (SSABIC) |  | 280435.668 |  |
|  | | | |

| **Other fit measures** | | | |
| --- | --- | --- | --- |
| **Metric** | | **Value** | |
| Root mean square error of approximation (RMSEA) |  | 0.049 |  |
| RMSEA 90% CI lower bound |  | 0.045 |  |
| RMSEA 90% CI upper bound |  | 0.054 |  |
| RMSEA p-value |  | 0.569 |  |
| Standardized root mean square residual (SRMR) |  | 0.044 |  |
| Hoelter's critical N (α = .05) |  | 355.619 |  |
| Hoelter's critical N (α = .01) |  | 384.134 |  |
| Goodness of fit index (GFI) |  | 0.948 |  |
| McDonald fit index (MFI) |  | 0.850 |  |
| Expected cross validation index (ECVI) |  | 0.527 |  |
|  | | | |

| **R-Squared** | | | |
| --- | --- | --- | --- |
|  | | **R²** | |
| C1Nx |  | 0.241 |  |
| C1Ny |  | 0.225 |  |
| C1Nz |  | 0.183 |  |
| C2Nx |  | 0.276 |  |
| C2Ny |  | 0.185 |  |
| C2Nz |  | 0.145 |  |
| C3Nx |  | 0.269 |  |
| C3Ny |  | 0.327 |  |
| C3Nz |  | 0.301 |  |
| A1Nx |  | 0.204 |  |
| A1Ny |  | 0.217 |  |
| A1Nz |  | 0.260 |  |
| A2Nx |  | 0.121 |  |
| A2Ny |  | 0.084 |  |
| A2Nz |  | 0.082 |  |
| A3Nx |  | 0.190 |  |
| A3Ny |  | 0.149 |  |
| A3Nz |  | 0.200 |  |
| Factor 1 |  | 1.000 |  |
|  | | | |

**Parameter estimates**

| **Factor loadings** | | | | | | | | | | | | | | | | | |
| --- | --- | --- | --- | --- | --- | --- | --- | --- | --- | --- | --- | --- | --- | --- | --- | --- | --- |
|  | | | | | | | | | | | | | | **95% Confidence Interval** | | | |
| **Factor** | | **Indicator** | | **Symbol** | | **Estimate** | | **Std. Error** | | **z-value** | | **p** | | **Lower** | | **Upper** | |
| Factor 1 |  | C1Nx |  | λ11 |  | 9.551 |  |  |  |  |  |  |  |  |  |  |  |
|  |  | C1Ny |  | λ12 |  | 9.394 |  |  |  |  |  |  |  |  |  |  |  |
|  |  | C1Nz |  | λ13 |  | 8.080 |  |  |  |  |  |  |  |  |  |  |  |
|  |  | C2Nx |  | λ14 |  | 10.324 |  |  |  |  |  |  |  |  |  |  |  |
|  |  | C2Ny |  | λ15 |  | 8.630 |  |  |  |  |  |  |  |  |  |  |  |
|  |  | C2Nz |  | λ16 |  | 7.243 |  |  |  |  |  |  |  |  |  |  |  |
|  |  | C3Nx |  | λ17 |  | 9.885 |  |  |  |  |  |  |  |  |  |  |  |
|  |  | C3Ny |  | λ18 |  | 10.815 |  |  |  |  |  |  |  |  |  |  |  |
|  |  | C3Nz |  | λ19 |  | 10.167 |  |  |  |  |  |  |  |  |  |  |  |
| Factor 2 |  | A1Nx |  | λ21 |  | 229.887 |  |  |  |  |  |  |  |  |  |  |  |
|  |  | A1Ny |  | λ22 |  | 236.003 |  |  |  |  |  |  |  |  |  |  |  |
|  |  | A1Nz |  | λ23 |  | 254.685 |  |  |  |  |  |  |  |  |  |  |  |
|  |  | A2Nx |  | λ24 |  | 157.357 |  |  |  |  |  |  |  |  |  |  |  |
|  |  | A2Ny |  | λ25 |  | 134.846 |  |  |  |  |  |  |  |  |  |  |  |
|  |  | A2Nz |  | λ26 |  | 132.639 |  |  |  |  |  |  |  |  |  |  |  |
|  |  | A3Nx |  | λ27 |  | 232.461 |  |  |  |  |  |  |  |  |  |  |  |
|  |  | A3Ny |  | λ28 |  | 194.331 |  |  |  |  |  |  |  |  |  |  |  |
|  |  | A3Nz |  | λ29 |  | 224.464 |  |  |  |  |  |  |  |  |  |  |  |
|  | | | | | | | | | | | | | | | | | |

| **Second-order factor loadings** | | | | | | | | | | | | | | | | | |
| --- | --- | --- | --- | --- | --- | --- | --- | --- | --- | --- | --- | --- | --- | --- | --- | --- | --- |
|  | | | | | | | | | | | | | | **95% Confidence Interval** | | | |
| **Factor** | | **Indicator** | | **Symbol** | | **Estimate** | | **Std. Error** | | **z-value** | | **p** | | **Lower** | | **Upper** | |
| SecondOrder |  | Factor 1 |  | γ11 |  | 25.340 |  |  |  |  |  |  |  |  |  |  |  |
|  | | | | | | | | | | | | | | | | | |

| **Factor variances** | | | | | | | | | | | | | |
| --- | --- | --- | --- | --- | --- | --- | --- | --- | --- | --- | --- | --- | --- |
|  | | | | | | | | | | **95% Confidence Interval** | | | |
| **Factor** | | **Estimate** | | **Std. Error** | | **z-value** | | **p** | | **Lower** | | **Upper** | |
| Factor 1 |  | 0.000 |  | 0.000 |  |  |  |  |  | 0.000 |  | 0.000 |  |
| Factor 2 |  | 1.000 |  | 0.000 |  |  |  |  |  | 1.000 |  | 1.000 |  |
| Second-Order |  | 1.000 |  | 0.000 |  |  |  |  |  | 1.000 |  | 1.000 |  |
|  | | | | | | | | | | | | | |

| **Residual variances** | | | | | | | | | | | | | |
| --- | --- | --- | --- | --- | --- | --- | --- | --- | --- | --- | --- | --- | --- |
|  | | | | | | | | | | **95% Confidence Interval** | | | |
| **Indicator** | | **Estimate** | | **Std. Error** | | **z-value** | | **p** | | **Lower** | | **Upper** | |
| C1Nx |  | 183977.987 |  |  |  |  |  |  |  |  |  |  |  |
| C1Ny |  | 194802.083 |  |  |  |  |  |  |  |  |  |  |  |
| C1Nz |  | 187013.097 |  |  |  |  |  |  |  |  |  |  |  |
| C2Nx |  | 179314.888 |  |  |  |  |  |  |  |  |  |  |  |
| C2Ny |  | 211217.582 |  |  |  |  |  |  |  |  |  |  |  |
| C2Nz |  | 198581.747 |  |  |  |  |  |  |  |  |  |  |  |
| C3Nx |  | 170767.708 |  |  |  |  |  |  |  |  |  |  |  |
| C3Ny |  | 154308.558 |  |  |  |  |  |  |  |  |  |  |  |
| C3Nz |  | 154293.961 |  |  |  |  |  |  |  |  |  |  |  |
| A1Nx |  | 206691.083 |  |  |  |  |  |  |  |  |  |  |  |
| A1Ny |  | 201291.763 |  |  |  |  |  |  |  |  |  |  |  |
| A1Nz |  | 184529.150 |  |  |  |  |  |  |  |  |  |  |  |
| A2Nx |  | 180091.639 |  |  |  |  |  |  |  |  |  |  |  |
| A2Ny |  | 198735.619 |  |  |  |  |  |  |  |  |  |  |  |
| A2Nz |  | 195748.639 |  |  |  |  |  |  |  |  |  |  |  |
| A3Nx |  | 230266.852 |  |  |  |  |  |  |  |  |  |  |  |
| A3Ny |  | 216357.615 |  |  |  |  |  |  |  |  |  |  |  |
| A3Nz |  | 201877.165 |  |  |  |  |  |  |  |  |  |  |  |
|  | | | | | | | | | | | | | |

**Table 11.2.6 Antipodes negative B-D**

**Model fit**

| **Chi-square test** | | | | | | | |
| --- | --- | --- | --- | --- | --- | --- | --- |
| **Model** | | **Χ²** | | **df** | | **p** | |
| Baseline model |  | 2290.485 |  | 153 |  |  |  |
| Factor model |  | 296.125 |  | 133 |  | < .001 |  |
|  | | | | | | | |

**Additional fit measures**

| **Fit indices** | | | |
| --- | --- | --- | --- |
| **Index** | | **Value** | |
| Comparative Fit Index (CFI) |  | 0.924 |  |
| Tucker-Lewis Index (TLI) |  | 0.912 |  |
| Bentler-Bonett Non-normed Fit Index (NNFI) |  | 0.912 |  |
| Bentler-Bonett Normed Fit Index (NFI) |  | 0.871 |  |
| Parsimony Normed Fit Index (PNFI) |  | 0.757 |  |
| Bollen's Relative Fit Index (RFI) |  | 0.851 |  |
| Bollen's Incremental Fit Index (IFI) |  | 0.924 |  |
| Relative Noncentrality Index (RNI) |  | 0.924 |  |
|  | | | |

| **Information criteria** | | | |
| --- | --- | --- | --- |
|  | | **Value** | |
| Log-likelihood |  | -140833.594 |  |
| Number of free parameters |  | 38.000 |  |
| Akaike (AIC) |  | 281743.188 |  |
| Bayesian (BIC) |  | 281930.842 |  |
| Sample-size adjusted Bayesian (SSABIC) |  | 281810.150 |  |
|  | | | |

| **Other fit measures** | | | |
| --- | --- | --- | --- |
| **Metric** | | **Value** | |
| Root mean square error of approximation (RMSEA) |  | 0.034 |  |
| RMSEA 90% CI lower bound |  | 0.029 |  |
| RMSEA 90% CI upper bound |  | 0.040 |  |
| RMSEA p-value |  | 1.000 |  |
| Standardized root mean square residual (SRMR) |  | 0.035 |  |
| Hoelter's critical N (α = .05) |  | 561.247 |  |
| Hoelter's critical N (α = .01) |  | 606.296 |  |
| Goodness of fit index (GFI) |  | 0.968 |  |
| McDonald fit index (MFI) |  | 0.924 |  |
| Expected cross validation index (ECVI) |  | 0.361 |  |
|  | | | |

| **R-Squared** | | | |
| --- | --- | --- | --- |
|  | | **R²** | |
| B1Nx |  | 0.166 |  |
| B1Ny |  | 0.199 |  |
| B1Nz |  | 0.265 |  |
| B2Nx |  | 0.183 |  |
| B2Ny |  | 0.143 |  |
| B2Nz |  | 0.151 |  |
| B3Nx |  | 0.266 |  |
| B3Ny |  | 0.115 |  |
| B3Nz |  | 0.169 |  |
| D1Nx |  | 0.191 |  |
| D1Ny |  | 0.228 |  |
| D1Nz |  | 0.179 |  |
| D2Nx |  | 0.265 |  |
| D2Ny |  | 0.241 |  |
| D2Nz |  | 0.156 |  |
| D3Nx |  | 0.224 |  |
| D3Ny |  | 0.125 |  |
| D3Nz |  | 0.175 |  |
| Factor 1 |  | 1.000 |  |
|  | | | |

**Parameter estimates**

| **Factor loadings** | | | | | | | | | | | | | | | | | |
| --- | --- | --- | --- | --- | --- | --- | --- | --- | --- | --- | --- | --- | --- | --- | --- | --- | --- |
|  | | | | | | | | | | | | | | **95% Confidence Interval** | | | |
| **Factor** | | **Indicator** | | **Symbol** | | **Estimate** | | **Std. Error** | | **z-value** | | **p** | | **Lower** | | **Upper** | |
| Factor 1 |  | B1Nx |  | λ11 |  | 8.652 |  |  |  |  |  |  |  |  |  |  |  |
|  |  | B1Ny |  | λ12 |  | 9.342 |  |  |  |  |  |  |  |  |  |  |  |
|  |  | B1Nz |  | λ13 |  | 10.783 |  |  |  |  |  |  |  |  |  |  |  |
|  |  | B2Nx |  | λ14 |  | 8.794 |  |  |  |  |  |  |  |  |  |  |  |
|  |  | B2Ny |  | λ15 |  | 7.955 |  |  |  |  |  |  |  |  |  |  |  |
|  |  | B2Nz |  | λ16 |  | 8.271 |  |  |  |  |  |  |  |  |  |  |  |
|  |  | B3Nx |  | λ17 |  | 10.782 |  |  |  |  |  |  |  |  |  |  |  |
|  |  | B3Ny |  | λ18 |  | 6.608 |  |  |  |  |  |  |  |  |  |  |  |
|  |  | B3Nz |  | λ19 |  | 7.780 |  |  |  |  |  |  |  |  |  |  |  |
| Factor 2 |  | D1Nx |  | λ21 |  | 222.772 |  |  |  |  |  |  |  |  |  |  |  |
|  |  | D1Ny |  | λ22 |  | 251.631 |  |  |  |  |  |  |  |  |  |  |  |
|  |  | D1Nz |  | λ23 |  | 212.763 |  |  |  |  |  |  |  |  |  |  |  |
|  |  | D2Nx |  | λ24 |  | 249.039 |  |  |  |  |  |  |  |  |  |  |  |
|  |  | D2Ny |  | λ25 |  | 248.016 |  |  |  |  |  |  |  |  |  |  |  |
|  |  | D2Nz |  | λ26 |  | 191.804 |  |  |  |  |  |  |  |  |  |  |  |
|  |  | D3Nx |  | λ27 |  | 248.606 |  |  |  |  |  |  |  |  |  |  |  |
|  |  | D3Ny |  | λ28 |  | 190.258 |  |  |  |  |  |  |  |  |  |  |  |
|  |  | D3Nz |  | λ29 |  | 212.581 |  |  |  |  |  |  |  |  |  |  |  |
|  | | | | | | | | | | | | | | | | | |

| **Second-order factor loadings** | | | | | | | | | | | | | | | | | |
| --- | --- | --- | --- | --- | --- | --- | --- | --- | --- | --- | --- | --- | --- | --- | --- | --- | --- |
|  | | | | | | | | | | | | | | **95% Confidence Interval** | | | |
| **Factor** | | **Indicator** | | **Symbol** | | **Estimate** | | **Std. Error** | | **z-value** | | **p** | | **Lower** | | **Upper** | |
| SecondOrder |  | Factor 1 |  | γ11 |  | 24.305 |  |  |  |  |  |  |  |  |  |  |  |
|  | | | | | | | | | | | | | | | | | |

| **Factor variances** | | | | | | | | | | | | | |
| --- | --- | --- | --- | --- | --- | --- | --- | --- | --- | --- | --- | --- | --- |
|  | | | | | | | | | | **95% Confidence Interval** | | | |
| **Factor** | | **Estimate** | | **Std. Error** | | **z-value** | | **p** | | **Lower** | | **Upper** | |
| Factor 1 |  | 0.000 |  | 0.000 |  |  |  |  |  | 0.000 |  | 0.000 |  |
| Factor 2 |  | 1.000 |  | 0.000 |  |  |  |  |  | 1.000 |  | 1.000 |  |
| Second-Order |  | 1.000 |  | 0.000 |  |  |  |  |  | 1.000 |  | 1.000 |  |
|  | | | | | | | | | | | | | |

| **Residual variances** | | | | | | | | | | | | | |
| --- | --- | --- | --- | --- | --- | --- | --- | --- | --- | --- | --- | --- | --- |
|  | | | | | | | | | | **95% Confidence Interval** | | | |
| **Indicator** | | **Estimate** | | **Std. Error** | | **z-value** | | **p** | | **Lower** | | **Upper** | |
| B1Nx |  | 222407.202 |  |  |  |  |  |  |  |  |  |  |  |
| B1Ny |  | 207666.416 |  |  |  |  |  |  |  |  |  |  |  |
| B1Nz |  | 190324.217 |  |  |  |  |  |  |  |  |  |  |  |
| B2Nx |  | 203985.651 |  |  |  |  |  |  |  |  |  |  |  |
| B2Ny |  | 224057.962 |  |  |  |  |  |  |  |  |  |  |  |
| B2Nz |  | 226527.084 |  |  |  |  |  |  |  |  |  |  |  |
| B3Nx |  | 189532.702 |  |  |  |  |  |  |  |  |  |  |  |
| B3Ny |  | 198861.435 |  |  |  |  |  |  |  |  |  |  |  |
| B3Nz |  | 175432.844 |  |  |  |  |  |  |  |  |  |  |  |
| D1Nx |  | 209720.138 |  |  |  |  |  |  |  |  |  |  |  |
| D1Ny |  | 214079.960 |  |  |  |  |  |  |  |  |  |  |  |
| D1Nz |  | 207101.868 |  |  |  |  |  |  |  |  |  |  |  |
| D2Nx |  | 172360.256 |  |  |  |  |  |  |  |  |  |  |  |
| D2Ny |  | 193696.672 |  |  |  |  |  |  |  |  |  |  |  |
| D2Nz |  | 199229.802 |  |  |  |  |  |  |  |  |  |  |  |
| D3Nx |  | 214278.348 |  |  |  |  |  |  |  |  |  |  |  |
| D3Ny |  | 252889.502 |  |  |  |  |  |  |  |  |  |  |  |
| D3Nz |  | 212366.709 |  |  |  |  |  |  |  |  |  |  |  |
|  | | | | | | | | | | | | | |
